# Effect of prenatal multiple micronutrient supplementation compared with iron and folic acid supplementation on size at birth and subsequent growth through 24 mo of age: a systematic review and meta-analysis

**DOI:** 10.1016/j.ajcnut.2025.04.022

**Published:** 2025-04-28

**Authors:** Filomena Gomes, Seth Adu-Afarwuah, Rina Agustina, Hasmot Ali, Amrita Arcot, Shams Arifeen, Charles D Arnold, Robert E Black, Parul Christian, Kathryn G Dewey, Wafaie W Fawzi, Lotta Hallamaa, John Hoddinott, Mihaela C Kissell, Klaus Kraemer, Carl Lachat, Sophie E Moore, Kenneth Maleta, Carolina Pereira, Dominique Roberfroid, Saijuddin Shaikh, Anuraj H Shankar, Emily R Smith, Inan Subarkah, Rahardjo Sunawang, Dongqing Wang, Lee S-F Wu, Martin N Mwangi

**Affiliations:** 1 Healthy Mothers Healthy Babies, The Micronutrient Forum, Washington, DC, United States; 2 NOVA Medical School, Universidade NOVA de Lisboa, Lisbon, Portugal; 3 Department of Nutrition and Food Science, University of Ghana, Legon, Accra, Ghana; 4 Department of Nutrition, Faculty of Medicine, Universitas Indonesia - Dr. Cipto Mangunkusumo General Hospital, Jakarta, Indonesia; 5 Human Nutrition Research Center, Indonesian Medical Education and Research Institute (HNRC-IMERI), Faculty of Medicine, Universitas Indonesia, Jakarta, Indonesia; 6 The JiVitA Project, Johns Hopkins University in Bangladesh, Gaibandha, Bangladesh; 7 International Centre for Diarrhoeal Diseases Research Bangladesh (ICDDR,B), Dhaka, Bangladesh; 8 Department of Nutrition and Institute for Global Nutrition, University of California-Davis, Davis, CA, United States; 9 Institute for International Programs, Department of International Health, Johns Hopkins Bloomberg School of Public Health, Baltimore, MD, United States; 10 Center for Human Nutrition, Department of International Health, Johns Hopkins Bloomberg School of Public Health, Baltimore, MD, United States; 11 Department of Global Health and Population, Harvard T.H. Chan School of Public Health, Boston, MA, United States; 12 Center for Child, Adolescent, and Maternal Health Research, Faculty of Medicine and Health Technology, Tampere University, Tampere, Finland; 13 Division of Nutritional Sciences, Cornell University, Ithaca, NY, United States; 14 Sight and Life Foundation, Basel, Switzerland; 15 Department of Food Technology, Safety and Health, Ghent University, Ghent, Belgium; 16 Department of Women & Children’s Health, King’s College London, London, United Kingdom; 17 MRC Unit The Gambia at the London School of Hygiene & Tropical Medicine, Fajara, The Gambia; 18 Kamuzu University of Health Sciences, Blantyre, Malawi; 19 Department of Medicine, Namur University, Namur, Belgium; 20 JiVitA Project, Rangpur, Bangladesh; 21 Oxford University Clinical Research Unit–Indonesia, Jakarta, Indonesia; 22 Nuffield Department of Medicine, University of Oxford, Oxford, United Kingdom; 23 Department of Global Health, Milken Institute School of Public Health, The George Washington University, Washington, DC, United States; 24 Center for Health Research, School of Public Health, University of Indonesia, Depok, Indonesia; 25 Department of Global and Community Health, College of Public Health, George Mason University, Fairfax, VA, United States; 26 Global Nutrition and Health Department, Wageningen University & Research, Wageningen, The Netherlands

**Keywords:** micronutrient supplements, pregnancy, length, birth size, infant growth

## Abstract

**Background:**

Prenatal multiple micronutrient supplementation (MMS), in comparison to iron and folic acid supplementation (IFA), improves pregnancy outcomes, but less is known about their effect on infant growth.

**Objectives:**

We conducted a systematic review of trials comparing maternal MMS to IFA and assessed the effect on infants’ anthropometric outcomes at birth, 3, 6, 12, 18, and 24 mo of age.

**Methods:**

We included trials from a Cochrane review and new studies identified through systematic literature searches in 3 databases. We calculated the pooled effect estimates with 95% confidence intervals (CIs) using a generic inverse variance method, with fixed (primary analysis) and random-effects, and assessed subgroup differences.

**Results:**

The 19 included trials showed that MMS, compared to IFA, led to significantly greater length and weight from birth to 6 mo, head circumference (HC) from birth to 12 mo, and mid-upper arm circumference (MUAC) through 3 mo. Infants born to pregnant women consuming MMS were longer at birth (mean difference: 0.05 cm; 95% CI: 0.02, 0.08 cm) and had higher length-for-age *z*-score at birth (0.09; 95% CI: 0.06, 0.12), 3 mo (0.09; 95% CI: 0.06, 0.12), and 6 mo (0.04; 95% CI: 0.01, 0.07) of age but not thereafter. MMS resulted in significantly higher weight-for-age *z*-score and HC-for-age *z*-score until 6 mo and higher weight-for-length *z*-score and MUAC-for-age *z*-score until 3 mo. MMS reduced risk of stunting (risk ratio [RR]: 0.86; 95% CI: 0.82, 0.91), underweight (RR: 0.86; 95% CI: 0.81, 0.90), small HC (RR: 0.84; 95% CI: 0.79, 0.90), and low MUAC (RR: 0.90; 95% CI: 0.82, 0.99) at 3 mo and wasting (RR: 0.90; 95% CI: 0.85, 0.96) at birth. For some outcomes, effects were greater when MMS was continued postpartum and in settings with higher prevalence of low birthweight.

**Conclusions:**

Prenatal MMS improves size at birth and subsequent infant growth through 6 mo of age but not thereafter. These results strengthen the evidence on MMS benefits beyond birth outcomes.

This study was registered in PROSPERO as CRD42024551864.

## Introduction

Pregnancy is characterized by major maternal physiological changes and rapid fetal development, both of which require optimal maternal micronutrient (vitamin and mineral) status [1,2]. Meeting micronutrient requirements during pregnancy is important for placentation, fetal organ development, fetal growth, and the delivery of healthy offspring [[3], [4], [5]]. Nearly 70% of nonpregnant women of reproductive age have a micronutrient deficiency, with a disproportionate fraction of these women living in sub-Saharan Africa and Asia [6], where anemia affects 31% to 52% of pregnant women [7]. The prevalence of micronutrient deficiencies in pregnancy is likely higher than that of women of reproductive age because nutritional requirements are higher to meet fetal demands [1,2] and because many pregnant women living in low- and middle-income countries (LMICs) have limited access and affordability for nutritionally adequate diets [8,9]. These high prevalences of micronutrient deficiencies lead to adverse pregnancy outcomes, such as low birthweight (LBW), prematurity, and small for gestational age (SGA; i.e. small vulnerable newborns), all of which increase neonatal mortality risk [10] and have long-term consequences from inadequate growth and poor cognitive and motor development to greater chronic disease risk [9,[11], [12], [13]].

Prenatal multiple micronutrient supplements (MMS) contain vitamins and minerals, including iron and folic acid (IFA), to address the gap between the typically low intakes of several micronutrients observed in LMICs and the increased requirements imposed by pregnancy and the growing fetus. The 2020 WHO recommendation on MMS [14] was based on the evidence generated by a Cochrane review, which evaluated the benefits of providing MMS compared to IFA (or iron alone) in 19 trials with 141,447 women conducted in LMICs [15]. In comparison with IFA, MMS has been shown to reduce risk of LBW, SGA births, preterm births, and stillbirths, with greater and additional benefits in anemic and underweight pregnant women [15,16]. The effect of MMS on other important anthropometric outcomes beyond weight (eg, length, head circumference [HC], or mid-upper arm circumference [MUAC]) at birth and during the first 2 y of life warrants further investigation. WHO [14] also called for the follow-up of infants whose mothers were provided MMS or IFA to assess if gains from MMS were sustained into childhood [14].

Thus, there is a critical gap in our understanding of prenatal MMS and its relationship to important infant anthropometric outcomes from birth to the second birthday. Our systematic review and meta-analysis aimed to comprehensively assess the effect of MMS compared to IFA on size at birth and subsequent growth status through 24 mo of age, including weight, length, height, HC, MUAC and associated *z*-scores, stunting, underweight, wasting, small HC, and low MUAC. The primary outcomes were birth length, length-for-age *z*-score (LAZ) at each prespecified age, and stunting at 24 mo, whereas the remaining outcomes were secondary.

## Methods

We conducted a systematic review and meta-analysis of trials that assessed the effect of daily prenatal MMS compared to IFA on an infant’s size at birth and/or anthropometric outcomes ≤24 mo of age. The review protocol was preregistered at PROSPERO (registration number CRD42024551864) on July 6, 2024 [17].

### Literature searches

We included the trials from Keats et al. [15], a 2019 Cochrane review that informed the 2020 WHO guideline on MMS [14], as well as newly published trials identified through the same search criteria and meeting the same inclusion criteria. Of the 19 trials included in the Cochrane review [15], which were conducted in LMICs and compared MMS with iron, with and without folic acid, 1 provided MMS twice weekly [18] and was, therefore, excluded from the current systematic review assessing the impact of daily supplementation.

Given our initial focus on the 18 trials included in the Cochrane review published in 2019 [15], there was a need to identify new trials and follow-up studies of the 18 included trials that conducted anthropometric assessments of the offspring ≤2 y of age. This was done in 3 steps. First, we conducted systematic literature searches in 3 electronic databases (MEDLINE [Ovid], Embase [Ovid], and Scopus) on July 2, 2024, without applying language restrictions, to identify new trials published since 2018 (the date of the literature searches for the Cochrane review) [15]. The search strategy employed in Embase (Ovid) is presented in Supplemental Appendix. Second, we conducted searches in MEDLINE (Ovid) using the trial identification number of each of the 18 trials included in the Keats et al. Cochrane review [15] to identify all the publications related to each trial. As a final step, we attempted to contact the corresponding author of each of the 18 trials included in the Cochrane review [15].

### Inclusion and exclusion criteria

Following the same inclusion criteria as the Cochrane review [15], we included only randomized controlled trials, and MMS was defined as a supplement containing ≥3 micronutrients, in addition to IFA. Trials with cointerventions (for example, food supplements) were considered eligible if the cointervention was similar in both intervention and control groups (see “Trials with multiple intervention groups” below). There was no exclusion related to the gestational age at the time of enrollment. Studies conducted specifically in HIV-infected pregnant women or only women with specific nutritional disorders (eg, anemic women) were excluded.

### Types of outcomes

We included anthropometric outcomes of eligible studies at birth and, if available, at the predefined follow-up periods of 3, 6, 12, 18, and 24 mo of age in the MMS and IFA arms. The prespecified outcomes at each of these times were the mean (SD) for the continuous outcomes, i.e., weight, length, HC, MUAC, LAZ, weight-for-age *z*-score (WAZ), weight-for-length *z*-score (WLZ), or birth body mass index-for-age *z*-score (BMIZ) if available, HC-for-age *z*-score (HCAZ), and MUAC-for-age *z*-score (MUACZ) (except at birth, which is not possible). Categorical outcomes included prevalence of stunting (LAZ < −2), underweight (WAZ < −2), wasting (WLZ < −2; whenever available, birth BMIZ < −2 was used instead, as WLZ excludes infant lengths < 45 cm), small HC (HCAZ < −2), and low MUAC (MUACZ <−2) (except at birth, which is not possible).

The primary outcomes were birth length, LAZ at each prespecified age, and stunting at 24 mo.

### Data extraction

Two independent reviewers (CP and FG) screened the abstracts retrieved in the systematic literature searches and reviewed the full text against the eligibility criteria. For all the included studies, the following data were extracted: first author and publication year, location, study design, parent trial and follow-up study, anthropometric assessments (reported as mean [SD] for continuous outcomes or prevalence for binary outcomes), duration of follow-up of anthropometric assessments ≤24 mo (predicted and actual), supplementation duration, prevalence of LBW in the control group, and growth standards used to produce the *z*-scores. Finally, information on the quality of anthropometric assessments was recorded, if available.

All extracted data on anthropometric outcomes were shared with the corresponding author and/or principal investigator of all included trials to confirm accuracy and to determine if additional unpublished data (as described above) were available. If the study authors decided to analyze and contribute unpublished data relevant to these analyses, they followed standardized methodology [19] to compute the *z*-scores at all-time points (using the 2006 WHO Growth Standards at all-time points) and used a common definition of the follow-up periods (±2 wk for all-time points until 24 mo and ±1 mo for 24 mo).

### Trials with multiple intervention groups

Three trials examined infant outcomes in 3 arms receiving 3 prenatal interventions: IFA compared with MMS compared with lipid-based nutrient supplements [[20], [21], [22]]. Only data related to the IFA and MMS arms were extracted for analyses. Kæstel et al. [23] had 3 study arms (IFA and MMS1 and MMS2 – containing 1 or 2 recommended dietary allowances of 15 micronutrients, respectively). The 2 MMS groups were combined by creating a single pairwise comparison, per the *Cochrane Handbook for Systematic Reviews of Interventions* [24]. The parent trial by Moore et al (ENID trial) and subsequent studies [[25], [26]] had 4 study arms (IFA, MMS, Protein and Energy supplements, Protein and Energy supplements paired with MMS) with additional randomization for infants to receive lipid-based nutrient supplements as a complementary food or placebo from 6 to 18 mo. Only data related to IFA and MMS contributed to the analysis until the initiation of lipid-based nutrient supplements, as this intervention could influence infant anthropometric outcomes. In this study, only birth weight data were included; the other anthropometric measures were taken at a later age of around 1 wk and were, therefore, excluded from the analysis.

Tofail et al. [[27], [28], [29]] had 6 study arms (3 × 2 factorial design) in which 3 arms (IFA with 30 mg iron, IFA with 60 mg iron, and MMS) received food supplementation at 9 wk of gestation (*early invitation to food supplementation*), and the other 3 arms (IFA with 30 mg iron, IFA with 60 mg iron, and MMS) received food supplementation at 20 wk of gestation (*usual invitation to food supplementation*). Only the intervention arms in which food supplementation began at 20 wk gestation *(usual invitation to food supplementation)* were included in the present analysis. For the current meta-analysis, the data from both IFA arms (30 mg iron and 60 mg iron) were merged into 1 group and compared with MMS.

### Overall analyses

We conducted meta-analyses to calculate the pooled mean differences with 95% confidence intervals (CIs) comparing MMS and IFA across multiple trials for all continuous anthropometric outcomes and the pooled risk ratios (RRs) with 95% CIs for the dichotomous anthropometric outcomes at all ages. We pooled data from individually and cluster-randomized trials using a generic inverse variance method after adjustment of estimates for cluster-randomized trials per the *Cochrane Handbook for Systematic Reviews of Interventions* [30]. From the 8 cluster-randomized trials included in the analyses, the trial publications from Bliznashka et al. [21], Shankar et al. [31], and Zagré et al. [32] provided the effect estimates adjusted for cluster design. For the remaining trials [[33], [34], [35], [36], [37]], we applied the design effect (described in Supplemental Table 1) to the number of events and sample sizes for dichotomous outcomes and to the sample sizes for continuous outcomes, to adjust the cluster-randomized trial data to their sample size. Fixed effects models were predefined as our primary analysis, and random-effects models were conducted as a sensitivity analysis. For inclusion in pooled analyses, studies were required to provide a sufficient sample size within each arm to estimate intervention effects (i.e., ≥30 per arm).

### Subgroup analyses

We conducted subgroup analyses for 2 potential effect modifiers: *1*) supplementation in pregnancy or both pregnancy and postpartum and *2*) trial baseline maternal health and nutritional status using LBW as a surrogate indicator, i.e., trials with a lower prevalence of LBW compared with trials with a higher prevalence of LBW (based on the median prevalence in the control groups).

For the subgroup analyses by MMS only in pregnancy or both pregnancy and postpartum, we excluded birth anthropometric assessments. We divided the studies into trials that discontinued MMS at birth [20,21,25,27] and trials that continued MMS postpartum, varying from 1 to 6 mo [20,22,35,36,38,39].

For the subgroup analyses by prevalence of LBW, we grouped the studies into trials with prevalence of LBW less than or equal to the median, i.e., “lower prevalence of LBW” [22,31,32,35,37,38,[40], [41], [42]], and trials with prevalence of LBW greater than the median, i.e., “higher prevalence of LBW” [20,23,25,27,33,34,36,39,43].

### Sensitivity analyses

We conducted 2 sensitivity analyses. First, we limited analysis to the 16 trials that contributed data to the updated 2020 WHO recommendation on MMS [14]. We excluded 2 trials that were included in the 2019 Cochrane review [15] but not used for the development of the 2020 WHO recommendation. One trial was excluded because it evaluated a supplement with 8 micronutrients plus IFA [38], and 1 trial was excluded because it did not provide folic acid to the control group [40]. In addition, to assess the influence of individual trials on the pooled estimates we also conducted sensitivity analyses with the leave-one-out method [44] by removing 1 study in each cycle and re-estimating the pooled mean difference or RR. We evaluated patterns in individual studies and their contribution to the pooled estimate for each outcome.

### Heterogeneity and publication bias assessment

We evaluated heterogeneity between studies using the χ^2^ test and *I*^2^ statistic and considered substantial heterogeneity when *I*^2^ > 50% [44]. Publication bias was visually inspected by the symmetry of effect sizes in a contour-enhanced funnel plot [45] and via Egger’s test [46]. Outcomes required a minimum of 10 trials for inclusion in the publication bias assessment to limit drawing conclusions from a small number of studies [46,47].

All data were analyzed in Review Manager (version 5.4) and R (version 4.3.3).

### Assessment of risk of bias in included studies

We assessed risk of bias (RoB) in all eligible trials with the Cochrane RoB 2 tool (individually randomized trials) and the RoB 2 for cluster-randomized trials [48]. Both reviewers (CP and AA) independently assessed all studies, followed by discussion to identify and resolve any disagreement in collaboration with a third reviewer (FG) when needed.

## Results

A total of 1123 abstracts were retrieved from the 3 databases. After removing duplicates, 575 abstracts remained for screening, and 1 new trial was identified as meeting the inclusion criteria [21] (Supplemental Figure 1). Table 1 describes the baseline characteristics of all the 19 included trials, of which 18 were sourced from the Keats et al. Cochrane review [20,22,23,25,27,[31], [32], [33], [34], [35], [36], [37], [38], [39], [40], [41], [42], [43]] and 1 [21] was identified from systematic literature searches. Trials were conducted in 14 countries and were published between 2003 and 2021. The majority (*n* = 11) of trials were individually randomized and controlled [20,22,23,25,27,[38], [39], [40], [41], [42], [43]]. Seven trials presented anthropometric outcomes at birth alone [23,[31], [32], [33], [34],^41^,^42^], whereas the remaining conducted follow-up studies that assessed anthropometric outcomes ≤24 mo of age. Of note, there were few trials that assessed anthropometric outcomes in the later predefined follow-up periods, particularly at 18 and 24 mo, resulting in very limited sample sizes contributing to the effect estimates at the later time points. All trials provided IFA to the control groups, except Ramakrishnan et al. [40], who provided only iron; this trial only contributed with birth anthropometric data to the present analyses. Half of all trials (*n* = 9) continued supplementation postpartum, with a range of 1 to 6 mo [20,22,31,[34], [35], [36], [37], [38], [39]]. Eight trials contributed unpublished outcome data for the meta-analysis [20,22,25,27,35,36,38,39], whereas for the other 11 trials, only published data were available and used in the analysis. The prevalence of LBW in the control group as reported in all studies (except 1 [21]) ranged from 2% to 43% with a median value of 11.9%.

**TABLE 1:** Baseline characteristics of included trials.

Author, year trial name (country)	Anthropometric measurements	All anthropometric follow-ups (≤ ∼24 mo) in the study design	Data contribution for current analyses and time points	Supplementation continued postpartum?	Prevalence of LBW (control group), %
Adu-Afarwuah, 2015 [22] iLiNS DYAD-Ghana (Ghana)	Weight, length, HC, MUAC	0, 3, 6, 12, and 18 mo	Contributed with unpublished data at 0, 3, 6, 12, and 18 mo	Yes, for 6 mo postpartum	10.1
Ashorn, 2015 [20] iLiNS-DYAD-M (Malawi)	Weight, length, HC, MUAC	0, 6, 12, 18, and 24 mo^1^	Contributed with unpublished data at 0, 6, 12, 18, and 24 mo	Yes, for 6 mo in the complete follow-up cohort. No, for the simplified follow-up cohort	12.7
Bhutta, 2009 [33] (Pakistan)	Weight, length, HC, MUAC	0 mo	Only published data at birth	No	17.7
Bliznashka, 2022 [21] (Niger)	Weight, length, MUAC	6–8 wk, 24 mo	Only published data at 24 mo	No	Unknown/not available in publication
Christian, 2003 [34] (Nepal)	Weight, length, HC	0 mo	Only published data at birth	Yes, for 12 wk after a live birth or 5 wk after a stillbirth or miscarriage	43.3
Fawzi, 2007 [38] (Tanzania)	Weight, length, HC	0, 3, 6, 12, and 18 mo	Contributed with unpublished data at 0, 3, 6, 12, and 18 mo	Yes, for 6 wk postpartum	9.4
Friis, 2004 [41] (Zimbabwe)	Weight, length, HC	0 mo	Only published data at birth	No	11.4
Kæstel, 2005 [23] (Guinea-Bissau)	Weight	0 mo	Only published data at birth	No	13.6
Liu, 2013 [42] (China)	Weight, length	0 mo	Only published data at birth	No	2.2
Moore, 2009 [25] ENID trial (The Gambia)	Weight, length, HC, MUAC	0, 3, 6, and 12 mo	Contributed with unpublished data at 0 and 6 mo	No	12.3
Osrin, 2005 [43] (Nepal)	Weight, length, HC, MUAC	0, 24–36 mo (mean 2.5 y - excluded)	Only published data at birth	No	25
Tofail, 2008 [27] MINIMat (Bangladesh)	Weight, length, HC	0, 1, 2, 3, 4, 5, 6, 9, 12, 18, and 24 mo	Contributed with unpublished data at 0, 3, 6, 12, 18, and 24 mo	No	30
Ramakrishnan, 2003 [40] (Mexico)	Weight, length, HC	0, 3, and 24 mo	Only published data at birth^2^	No	8.9
Roberfroid, 2008 [39] (Burkina Faso)	Weight, length, HC, MUAC, chest circumference	0 mo then monthly ≤12 mo; small cohort assessed at 18 and 24 mo	Contributed with unpublished data at 0, 3, 6, 12, 18, and 24 mo	Yes, for 3 mo postpartum	15.6
Shankar, 2008 [31] SUMMIT (Indonesia)	Weight	0 mo	Only published data at birth	Yes, for 90 d postpartum	11
Sunawang, 2009 [35] (Indonesia)	Weight, length, HC	0, then monthly ≤12 mo (in a subset)	Contributed with unpublished data at 0, 3, 6, and 12 months	Yes, for 30 d postpartum	7.3
West, 2014 [36] JiVitA (Bangladesh)	Weight, length, HC, MUAC, chest circumference	0, 3, 6, 12, and 24 mo (actual follow-up 2.9 y)	Contributed with unpublished data at 0, 3, 6, 12, and 24 mo	Yes, for 12 wk postpartum	40.2
Zagré, 2007 [32] (Niger)	Weight	0 mo	Only published data at birth	No	8.4
Zeng, 2008 [37] (China)	Weight, length, HC	Follow-up time period is not clear – “during the first 30 mo”	Only published data at birth	Yes, for 6 wk postpartum	4.5

Abbreviations: HC, head circumference; LBW, low birthweight; MMS, multiple micronutrient supplementation; MUAC, mid-upper arm circumference.

1 This trial included 2 groups with different interventions after birth and follow-up periods: *1*) Complete follow-up group: the intervention (MMS) continued for 6 mo postpartum, with the control group receiving a daily supplement of 200 mg calcium; anthropometric outcomes assessed at 6, 12, 18, and 24 mo. *2*) Simplified follow-up: the intervention (MMS) and control (iron and folic acid) were discontinued at delivery, and fewer follow-up assessments were conducted (6 and 18 mo).

2 Infants were randomized to additional supplements at 3 mo, and the 3 mo published data were not in the required format.

### Overall analyses

Regarding the primary outcomes, relative to IFA, MMS increased length at birth (mean difference: 0.05 cm; 95% CI: 0.02, 0.08 cm; 14 trials), and LAZ at birth (0.09; 95% CI: 0.06, 0.12; 8 trials), 3 mo (0.09; 95% CI: 0.06, 0.12; trials), and 6 mo (0.04; 95% CI: 0.01, 0.07; 9 trials), but not thereafter (Table 2; Supplemental Figure 2). MMS had no effect on stunting at 24 mo (RR: 1.03; 95% CI: 0.98, 1.07; 5 trials). Heterogeneity was low (*I*^2^ = 0–33%) for most primary outcomes, i.e., LAZ at birth to ≤12 months and stunting at 24 mo.

**TABLE 2:** Pooled effects of MMS compared with IFA on length, LAZ, stunting (fixed effects).

Effect of MMS vs. IFA	Length (cm)			LAZ score			Stunting (LAZ <−2)
	No. of studies	No. of infants	Mean difference (95% CI), fixed effects	No. of studies	No. of infants	Mean difference (95% CI), fixed effects	Risk ratio (95% CI), fixed effects
Birth	14	43,546	0.05 (0.02, 0.08)	8	25,664	0.09 (0.06, 0.12)	0.91 (0.88, 0.95)
3 mo	7	24,669	0.18 (0.12, 0.24)	7	24,625	0.09 (0.06, 0.12)	0.86 (0.82, 0.91)
6 mo	9	18,878	0.08 (0.01, 0.16)	9	18,850	0.04 (0.01, 0.07)	0.96 (0.91, 1.02)
12 mo	7	16,314	0.05 (−0.03, 0.14)	7	14,293	0.02 (−0.02, 0.06)	1.01 (0.97, 1.06)
18 mo	5	2937	0.20 (−0.03, 0.43)	5	2927	0.01 (−0.06, 0.09)	0.99 (0.90, 1.09)
24 mo	4	8450	−0.06 (−0.20, 0.08)	5	8693	−0.03 (−0.07, 0.02)	1.03 (0.98, 1.07)

The table shows the generic inverse variance weighted pooled mean differences or pooled risk ratios with their corresponding 95% CIs comparing MMS and IFA intervention groups. Number of infants were calculated from effective sample sizes.

Abbreviations: CI, confidence interval; IFA, iron and folic acid supplements; LAZ, length-for-age *z*-score; MMS, multiple micronutrient supplementation.

TABLE 2, TABLE 3, TABLE 4, TABLE 5, TABLE 6 summarize the overall effects of MMS compared to IFA on length (Table 2), HC (Table 3), weight (Table 4), MUAC (Table 6), and related outcome indicators (*z*-scores and malnutrition indicators); Table 5 shows the effects for WLZ and wasting.

**TABLE 3:** Pooled effects of MMS compared with IFA on for HC, HCAZ, and small HC (fixed effects).

Effect of MMS vs. IFA	HC (cm)			HCAZ score			Small HC (HCAZ <−2)
	No. of studies	No. of infants	Mean difference (95% CI), fixed effects	No. of studies	No. of infants	Mean difference (95% CI), fixed effects	Risk ratio (95% CI), fixed effects
Birth	12	31,852	0.14 (0.10, 0.17)	7	25,902	0.12 (0.09, 0.15)	0.88 (0.84, 0.92)
3 mo	6	24,765	0.12 (0.09, 0.16)	6	24,720	0.10 (0.07, 0.12)	0.84 (0.79, 0.90)
6 mo	8	18,759	0.06 (0.02, 0.10)	8	18,734	0.04 (0.01, 0.07)	0.98 (0.91, 1.06)
12 mo	6	16,170	0.05 (0.00, 0.09)	6	16,163	0.02 (−0.01, 0.05)	0.96 (0.91, 1.02)
18 mo	4	1488	−0.09 (−0.23, 0.05)	4	1488	0.04 (−0.06, 0.14)	0.89 (0.68, 1.16)
24 mo	2	7136	0.01 (−0.06, 0.08)	2	7136	0.00 (−0.04, 0.05)	1.00 (0.93, 1.06)

The table shows the generic inverse variance weighted pooled mean differences or pooled risk ratios with their corresponding 95% CIs comparing MMS and IFA intervention groups. Number of infants were calculated from effective sample sizes.

Abbreviations: CI, confidence interval; HC, head circumference; HCAZ, head circumference-for-age *z*-score; IFA, iron and folic acid supplementation; MMS, multiple micronutrient supplementation.

**TABLE 4:** Pooled effects of MMS compared with IFA on weight, WAZ, underweight (fixed effects).

Effect of MMS vs. IFA	Weight (kg)			WAZ score			Underweight (WAZ <−2)
	No. of studies	No. of infants	Mean difference (95% CI), fixed effects	No. of studies	No. of infants	Mean difference (95% CI), fixed effects	Risk ratio (95% CI), fixed effects
Birth	18	60,379	0.04 (0.03, 0.05)	9	28,444	0.12 (0.09, 0.14)	0.87 (0.84, 0.91)
3 mo	7	24,990	0.11 (0.09, 0.13)	7	24,969	0.10 (0.07, 0.13)	0.86 (0.81, 0.90)
6 mo	9	19,063	0.04 (0.01, 0.07)	9	19,041	0.04 (0.00, 0.07)	0.97 (0.91, 1.04)
12 mo	7	16,534	0.02 (−0.01, 0.05)	7	16,523	0.02 (−0.01, 0.06)	0.97 (0.92, 1.01)
18 mo	8	2941	0.09 (−0.00, 0.18)	5	2926	0.05 (−0.03, 0.13)	0.89 (0.78, 1.02)
24 mo	4	8718	0.01 (−0.04, 0.05)	5	10,073	0.00 (−0.04, 0.04)	1.01 (0.96, 1.05)

The table shows the generic inverse variance weighted pooled mean differences or pooled risk ratios with their corresponding 95% CIs comparing MMS and IFA intervention groups. Number of infants were calculated from effective sample sizes.

Abbreviations: CI, confidence interval; IFA, iron and folic acid supplementation; MMS, multiple micronutrient supplementation; WAZ, weight-for-age *z*-score.

**TABLE 5:** Pooled effects of MMS compared with IFA on WLZ and wasting (fixed effects).

Effect of MMS vs. IFA	WLZ score			Wasting (WLZ <−2)		
	No. of studies	No. of infants	Mean difference (95% CI), fixed effects	No. of studies	No. of infants	Risk ratio (95% CI), fixed effects
Birth^1^	8	22,884	0.08 (0.05, 0.11)	8	21,567	0.90 (0.85, 0.96)
3 mo	7	24,688	0.03 (0.00, 0.06)	7	24,688	0.94 (0.85, 1.04)
6 mo	9	18,868	0.00 (−0.03, 0.04)	9	18,780	1.05 (0.94, 1.16)
12 mo	7	16,297	0.03 (−0.01, 0.07)	7	16,297	0.97 (0.90, 1.04)
18 mo	5	2922	0.06 (−0.02, 0.14)	5	2922	0.90 (0.72, 1.14)
24 mo	5	9810	−0.00 (−0.04, 0.04)	5	9810	1.01 (0.92, 1.09)

The table shows the generic inverse variance weighted pooled mean differences or pooled risk ratios with their corresponding 95% CI comparing MMS and IFA intervention groups. Number of infants were calculated from effective sample sizes.

Abbreviations: BMIZ, body mass index *z*-score; CI, confidence interval; IFA, iron and folic acid supplementation; MMS, multiple micronutrient supplementation; WLZ, weight-for-length *z*-score.

1 BMIZ for 5 studies and WLZ for 3 studies.

**TABLE 6:** Pooled effects of MMS compared with IFA on MUAC, MUACZ, and low MUAC (fixed effects).

Effect of MMS vs. IFA	MUAC (cm)			MUACZ score			Low MUAC (MUACZ <−2)
	No. of studies	No. of infants	Mean difference (95% CI), fixed effects	No. of studies	No. of infants	Mean difference (95% CI), fixed effects	Risk ratio (95% CI), fixed effects
Birth	5	17781	0.11 (0.08, 0.13)	N.A.	N.A.	N.A,	N.A.
3 months	6	20937	0.08 (0.06, 0.11)	6	20733	0.07 (0.05, 0.10)	0.90 (0.82, 0.99)
6 months	8	15388	0.02 (-0.01, 0.05)	8	15382	0.02 (-0.01, 0.05)	0.99 (0.88, 1.11)
12 months	6	14788	0.02 (-0.01, 0.06)	6	14784	0.02 (-0.01, 0.05)	0.99 (0.90, 1.09)
18 months	4	1490	0.06 (-0.05, 0.17)	4	1490	0.06 (-0.03, 0.16)	0.91 (0.52, 1.59)
24 months	4	8659	-0.01 (-0.05, 0.03)	3	7299	-0.01 (-0.05, 0.03)	0.92 (0.83, 1.02)

The table shows the generic inverse variance weighted pooled mean differences or pooled risk ratios with their corresponding 95% CIs comparing MMS and IFA intervention groups. Number of infants were calculated from effective sample sizes.

Abbreviations: CI, confidence interval; IFA, iron and folic acid supplementation; MMS, multiple micronutrient supplementation; MUAC, mid-upper arm circumference; MUACZ, mid-upper arm circumference *z*-score; N.A., non applicable.

In comparison with IFA, MMS resulted in higher length and weight at each age from birth until 6 mo, higher HC at birth through 12 mo, and higher MUAC at birth and 3 mo (all *P* < 0.05; TABLE 2, TABLE 3, TABLE 4, TABLE 6). The associated *z*-scores reflect similar results, with MMS resulting in higher LAZ, WAZ, and HCAZ at each cross-sectionally examined age from birth through 6 mo and in higher WLZ and MUACZ until 3 mo (all *P* < 0.05; TABLE 2, TABLE 3, TABLE 4, TABLE 5, TABLE 6). Similarly, in comparison with IFA, MMS resulted in a lower risk of indicators of undernutrition through 3 mo but not thereafter, including stunting (with a risk reduction of 9% at birth [RR: 0.91; 95% CI: 0.88, 0.95] and 14% at 3 mo [RR: 0.86; 95% CI: 0.82, 0.91]), small HC (with a risk reduction of 12% at birth [RR: 0.88; 95% CI: 0.84, 0.92] and 16% at 3 mo (RR: 0.84; 95% CI: 0.79,0.90]), underweight (with a risk reduction of 13% at birth [RR: 0.87; 95% CI: 0.84, 0.91] and 14% at 3 mo [RR: 0.86; 95% CI: 0.81, 0.90]), and low MUAC (with a risk reduction of 10% at 3 mo, RR: 0.90; 95% CI: 0.82, 0.99]), and wasting (with a risk reduction of 10%, RR: 0.90; 95% CI: 0.85, 0.96) at birth.

Of note, the relatively small differences in mean HC between the groups at 3 mo (0.12 cm; Table 3) translated to a meaningful 16% reduction in risk of small HC (RR: 0.84; 95% CI: 0.79, 0.90; Table 3). In most cases, similar results were observed between the fixed effects and random-effects models (Supplemental Figure 2). Heterogeneity was generally low (*I*^2^ <40%) for nearly all outcomes.

### Subgroup analyses

The results for the subgroup analyses are shown in Supplemental Table 2 (Supplemental Tables 2.1–2.14). The effects of MMS compared with IFA on anthropometric outcomes were greater among trials that continued maternal supplementation postpartum than among those that discontinued supplementation at birth (*P* value for subgroup differences < 0.05) for length, weight, and HC at 3 mo; LAZ at 3, 6 and 18 mo; stunting at 3 and 6 mo; as well as small HC at 3 mo. No other subgroup differences were observed. In nearly all cases, similar results were observed between the fixed and random-effects models.

The median value of LBW prevalence in the control groups was 11.9%. The effects of MMS compared with IFA on anthropometric outcomes were greater among trials conducted in settings with higher prevalence of LBW than those conducted in settings with lower prevalence of LBW (*P* value for subgroup differences < 0.05) for length at birth, HC at birth and 3 mo, MUAC at 3 mo, WAZ at 3 mo, and HCAZ at birth and 3 mo. In trials conducted in settings with lower LBW prevalence, the effects of MMS compared with IFA on anthropometric outcomes were greater for weight at 3 mo and LAZ at 18 mo than in trials conducted in settings with higher LBW prevalence (*P* value for subgroup differences < 0.05).

### Sensitivity analyses limited to the 16 studies included in the WHO analyses

Sensitivity analyses limited to the 16 studies included in the WHO analyses are shown in Supplemental Table 3. The effect estimates were similar to the overall analyses with the 19 trials, i.e., with beneficial effects of MMS compared with IFA on size at birth and growth ≤3 or 6 mo of age, except for MUAC at 3 mo, where the effect of MMS became significant in the sensitivity analyses.

### Leave-one-out sensitivity analyses

Leave-one-out sensitivity analyses (Supplemental Figure 3) reported a 0.01 to 0.15 cm fixed mean difference in length at birth, with West et al. [36] and Liu et al. [42] contributing the most to the pooled estimate. The greatest contribution to the pooled estimates was West et al. [36] and to a lesser extent Fawzi et al. [38].

### RoB in included studies and quality of anthropometric measurements

The RoB was generally low for included individually and cluster-randomized studies (Supplemental Figure 4). All judgments in the individually randomized trials were characterized as low risk (Supplemental Figure 4.1 and 4.3). In cluster-randomized trials, 88% of the first 2 domains were characterized as low risk (Supplemental Figure 4.2 and 4.4) [[31], [32], [33], [34],^36^,^37^]. Missing outcome data (attrition bias, domain 3) raised some concerns in ∼40% of cluster-randomized trials [21,32,34]. The last 2 domains (measurement of outcomes and selection of reported results) were characterized as low risk for all studies.

The information on the quality of anthropometric measurements was extracted (data not shown) and considered in the RoB assessment. All studies followed standard methodology for the assessment of anthropometric measurements in infants. Regarding the timing of birth assessments, some studies measured infants immediately after delivery; all studies aimed to complete these assessments within 72 h, allowing the weighing of infants born at home. For the majority of infants, weights were successfully obtained shortly after birth. Four studies accounted for variations in assessment timing by modeling the later weights back to the time of birth [20,22,25,27].

### Publication bias

Three outcomes were eligible for publication bias assessment (≥10 trials): length, HC, and weight at birth. Visual inspection of contour-enhanced funnel plots did not suggest publication bias, which was confirmed with Egger’s test (*P* > 0.05 for all outcomes; Supplemental Figure 5).

## Discussion

Infants of pregnant women who received MMS had higher weight and length from birth until 6 mo, higher HC from birth until 12 mo, and higher MUAC from birth until 3 mo compared to those whose mothers received IFA. Similarly, MMS led to higher LAZ, WAZ, and HCAZ from birth until 6 mo of age; higher WLZ and MUACZ from birth until 3 mo; a lower risk of stunting, underweight, small HC, and low MUAC from birth until 3 mo; and a lower risk of wasting at birth. Overall, these results suggest that, in comparison with IFA, MMS results in improved birth size as well as growth ≤6 mo but not thereafter.

Benefits on growth within the first 3 to 6 mo after birth are important because infants are vulnerable to growth faltering during this age interval [49], and there are few options for interventions to prevent growth faltering apart from early detection and treatment of infections and promotion and support of exclusive breastfeeding. After 6 mo, the effects of MMS are attenuated, perhaps because other factors (eg, complementary feeding with associated variations in dietary intake and breastfeeding practices; frequent infections) have a stronger influence on growth outcomes, although limited follow-up data after 6 mo could also account for inability to demonstrate significant effects.

Prenatal supplementation continued postpartum may improve some infant anthropometric outcomes however, few studies contributed to this subgroup analysis, warranting caution in interpreting these results. The subgroup analyses by prevalence of LBW suggested greater benefits of MMS for several anthropometric outcomes ≤3 mo in areas of higher prevalence of LBW, although greater benefits of MMS were observed for only 2 outcomes at 2 time points in settings with lower prevalence of LBW. Finally, we found no differences in results based on the 16 trials included in the WHO analysis [14] and the 19 total trials included in this review.

### Length, LAZ, and stunting

Our findings suggest that maternal MMS can improve length-related outcomes in the first 6 mo of life. A previous systematic review of 12 trials [50] reported no differences between prenatal MMS and IFA in infants’ birth length, but our analyses included 7 additional trials. At 3 mo, the relatively small mean difference of 0.18 cm between MMS and IFA translates into a significant reduction of stunting by 14%, likely due to a shift in the left tail of the distribution.

Stunting in early childhood is directly associated with reduced adult stature, with implications for adverse pregnancy and obstetric outcomes [51]; it is also associated with lower educational attainment, test performance, future economic success [52,53], and child mortality [54].

### HC and HCAZ

We found that MMS resulted in higher HC from birth until 12 mo and a higher HCAZ from birth until 6 mo, which contrasts with previous systematic reviews that reported no overall effect on HC [50,55]. Fall et al. [50] exclusively examined outcomes at birth, and only 10 studies contributed to the estimates of HC, compared to 12 in our analyses (including the largest trial [36]).

The mean HCAZ scores observed in the included studies (Supplemental Figure 2.8) are mostly negative values. As such, a small shift may mean a large difference in the left tail of the distribution, namely at <−2 Z. This may explain why the relatively small differences in mean HC measurements between MMS compared with IFA, eg, of 0.12 cm at 3 mo, translate into a 16% statistically significant reduction of infants with small HC at 3 mo. As HC measurements reflect brain size, the observed significant reduction in small HC of 12% at birth and 16% at 3 mo could have implications in neurodevelopmental outcomes [52,56].

Several infant anthropometric measures, including HC, have been associated with cognitive outcomes in the first 24 mo of life [57]. A few trials assessed the effect of prenatal MMS compared with IFA on cognitive and motor domains in infants and children. Anemic or undernourished pregnant women in Indonesia given MMS had children with improved motor and cognitive (visual attention/spatial ability) outcomes at 42 mo of age [58], and a later follow-up showed that children (ages 9–12 y) had higher procedural memory [59]. Another trial demonstrated that MMS was associated with improvements in intellectual development among adolescents (ages 10–14 y) in China [60]. Similarly, MMS was associated with higher mean intelligence quotient in 12-y-old Nepalese girls [61]. Scaling up MMS to 90% coverage in LMICs has been projected to contribute to better educational attainment and economic success [62].

### Wasting, underweight, WAZ, WLZ, MUAC, and MUACZ

In line with previous evidence showing that MMS reduces risk of LBW, SGA, and preterm births [15,16], we found that MMS resulted in higher weight and WAZ at birth until 6 mo and lower risk of underweight from birth until 3 mo and wasting at birth. Additionally, we found a higher MUAC at birth (as opposed to Fall et al. [50], with fewer studies) and at 3 mo, a higher MUACZ, and a risk reduction of low MUAC until 3 mo. Our study restricted follow-up to 24 mo because this is a critical time of physical and cognitive development and because sparse data are available after that period [51,63]. Growth failure in early life is associated with mortality in the first 24 mo of life [64]. Notably, compared to IFA, MMS reduced 3-mo infant mortality by 18% in Indonesia [31] and 6-mo infant mortality by 29% among children of anemic women in an individual participant data meta-analysis [16].

### Strengths and limitations

The main strength of this systematic review and meta-analysis is that we examined the effect of MMS compared with IFA on infants’ anthropometric outcomes within the first 24 mo of life, at 6 prespecified ages, using published and unpublished data. We included high-quality trials that were previously in a Cochrane review [15] and conducted our own searches to include any new eligible trials. We conducted a rigorous review of all literature as per PRISMA guidelines and Cochrane methodology [65,66]. Additionally, we contacted all principal investigators from the included studies to check the accuracy of the extracted data and gain access to relevant unpublished data. Most primary outcomes, LAZ (from birth to ≤12 mo) and stunting at 24 mo, had low statistical heterogeneity (*I*^2^ = 0–33%), suggesting little variation among studies. Our primary inference was based on a fixed-effect pooling approach, which estimates a common effect across studies; however, we conducted all analyses with both fixed and random effect approaches and have presented and interpreted both sets of results for full transparency.

The subgroup analyses showed improved anthropometric outcomes for infants whose mothers continued MMS postpartum. Although no firm conclusions can be drawn because of the limited sample sizes and short duration of postpartum supplementation in a few trials [35,38], this limited evidence supports global maternal nutrition guidance stating that “In settings where MMS are provided during pregnancy, women can continue taking them during the postnatal period” [67]. Our analysis provides important insights into the benefit of MMS on infant size and growth covering a time period that had not been addressed in previous meta-analyses [50,55].

Our systematic review has limitations. First, all studies reported anthropometric outcomes at birth, but fewer assessed anthropometric outcomes at the predefined ages, with the data for the older ages being more limited, eg, only 2 to 5 trials contributed data for the outcomes assessed at 24 mo. This may have contributed to the lack of observed associations at the later follow-ups. Second, the availability of the most appropriate *z*-scores was suboptimal. Although birth BMIZ was preferably used in our review (*n* = 5), when this outcome was not available, we used WLZ (*n* = 3), which excludes infant lengths <45 cm from the analyses [68]. Third, sensitivity analysis indicated that West et al. [36] and to a lesser extent Fawzi et al. [38] and Liu et al. [42] contributed the most to pooled estimates, likely related to their larger sample sizes. Finally, subgroup analysis by the prevalence of LBW was defined by the median prevalence of LBW in the control group. Future analysis would benefit from other setting-specific proxy indicators for poor nutrition, such as the prevalence of micronutrient deficiencies, maternal stunting and low BMI, and prevalence of infections.

### Conclusion

In comparison to IFA, prenatal MMS improved size at birth (including weight, length, HC, and MUAC) and subsequent infant growth through 6 mo of age. This work supports the conclusion that MMS reduces the incidence of small vulnerable newborns [69], who have greater risk of neonatal mortality [70], and also reduces risk of several indicators of undernutrition—stunting, underweight, and small HC—in the critical first months of life. Our findings support the use of MMS for all women in LMICs to reduce the burden of maternal and infant undernutrition.

## Author contributions

The authors’ responsibilities were as follows – FG, RA, SA, REB, PC, MCK, KGD, JH, KK, MM, ERS, AHS: conceptualized and designed the study; FG, CP: conducted the literature searches, abstract screening, and data extraction; FG, CP: performed the overall and subgroup analysis, as well as some analyses of unpublished data, with statistical guidance from CA; CP, AA conducted risk of bias assessment; AA: performed the publication bias and leave-one-out sensitivity analysis; FG, AA, CP: drafted the manuscript; and all authors: read and approved the final manuscript.

## Data availability

Data described in the manuscript will be made available upon request.

## Funding

This work was primarily funded by the Eleanor Crook Foundation. Additional support was leveraged from Kirk Humanitarian and the Gates Foundation. The funders had no role in the design, data collection, analysis, interpretation, or decision to publish this manuscript.

## Conflict of interest

PC is an Associate Editor for the *American Journal of Clinical Nutrition* and played no role in the Journal’s evaluation of the manuscript. MNM reports financial support was provided by Eleanor Crook Foundation. Other authors report no conflicts of interest.
