# Supplementary material for: Effect of prenatal multiple micronutrient supplementation compared with iron and folic acid supplementation on size at birth and subsequent growth through 24 mo of age: a systematic review and meta-analysis
Source: Am J Clin Nutr. 2025 Apr 28;122(1):185–95. doi: 10.1016/j.ajcnut.2025.04.022 (PMC12308086; doi:10.1016/j.ajcnut.2025.04.022)

Effect of MMS vs IFA on size at birth and subsequent growth through 24 months of age: a systematic review and meta-analysis (Gomes et al)

Supplementary Materials

Contents

Supplemental Appendix - Search strategy on Medline (Ovid) on July 2nd, 2024.. 1

Supplemental Table 1 – Design effects of cluster-randomized controlled trials 2

Supplemental Figure 1 - PRISMA flow diagram (literature searches and screening)...........,,..................... 3

Supplemental Figures 2 – Forest plots for overall analyses (fixed and random effects) 4

Supplemental Tables 2 - Subgroup analyses stratified by supplementation in pregnancy vs pregnancy and postpartum, and by lower vs higher prevalence rates of low birthweight (LBW)...................................... 86

Supplemental Tables 3 – Sensitivity analyses limited to the 16 studies included in the WHO analyses... 106

Supplemental Figures 3 – Leave-one-out sensitivity analyses.. 108

Supplemental Figures 4 - Risk of bias assessment.. 273

Supplemental Figures 5 - Publication bias assessment... 275

Supplemental Appendix - Search strategy on Medline (Ovid) on July 2nd, 2024

1. RANDOMIZED CONTROLLED TRIAL.mp. or exp randomized controlled trial/
2. random$.ti,ab.
3. 1 or 2 I
4. exp pregnancy/
5. (pregnan* or maternal or pretenatal or antenatal).mp. [mp=title, book title, abstract, original title, name of substance word, subject heading word, floating sub-heading word, keyword heading word, organism supplementary concept word, protocol supplementary concept word, rare disease supplementary concept word, unique identifier, synonyms, population supplementary concept word, anatomy supplementary concept word]
6. 4 or 5
7. ((micronutrient* or multivitamin*) adj3 supplement*).mp. [mp=title, book title, abstract, original title, name of substance word, subject heading word, floating sub-heading word, keyword heading word, organism supplementary concept word, protocol supplementary concept word, rare disease supplementary concept word, unique identifier, synonyms, population supplementary concept word, anatomy supplementary concept word]
8. multiple* micronutrient*.mp.
9. (nutri* adj3 supplement*).mp.
10. 7 or 8 or 9
11. 3 and 6 and 10
12. limit 11 to yr="2018 -Current"

Supplemental Table 1 – Design effects of cluster-randomized controlled trials

**Trial**	**Design effect**
Bhutta 2009	2
Christian 2003	1.2
Sunawang 2009	1.2
West 2014	1.5
Zeng 2008	1.26 for weight and length; 1.7 for head circumference

Supplemental Figure 1 - PRISMA flow diagram (literature searches and screening)

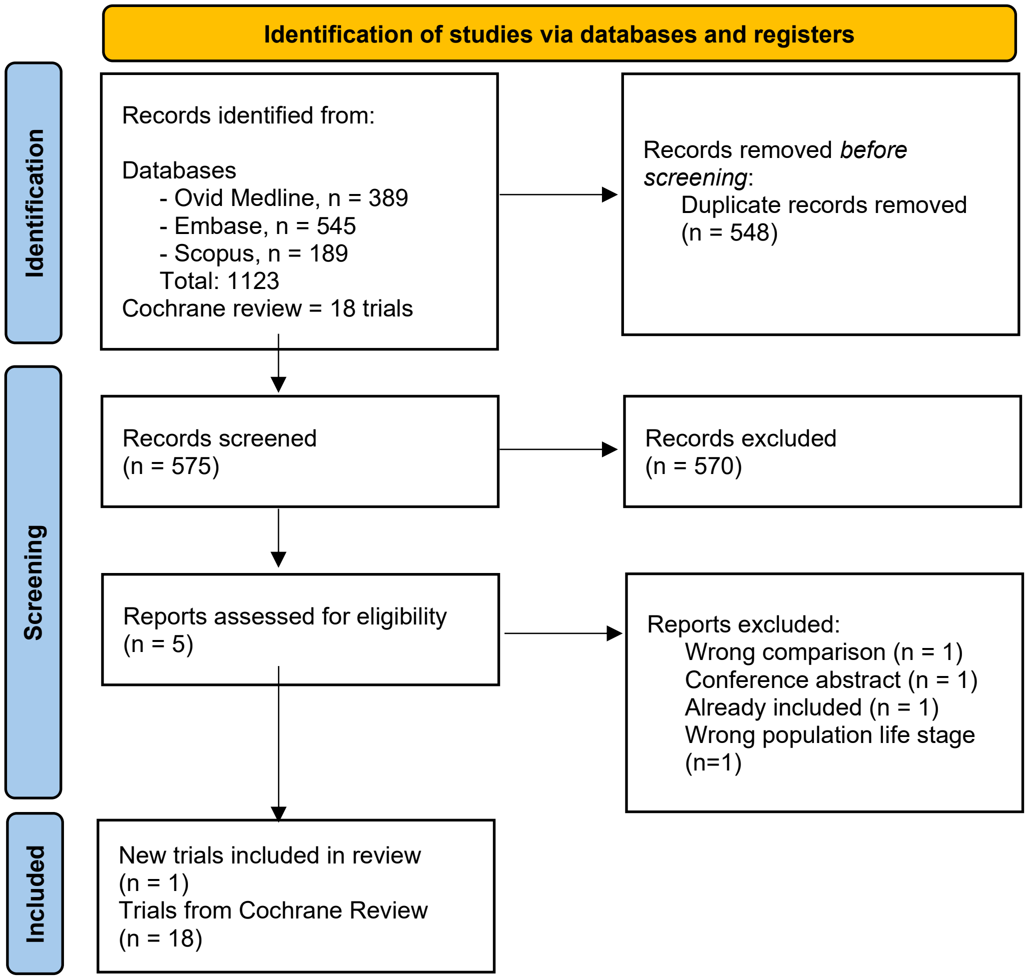

Supplemental Figures 2 – Forest plots for overall analyses (fixed and random effects)

Supplemental Figure 2.1. Effect of MMS vs IFA on length

Supplemental Figure 2.1.1. Effect of MMS vs IFA on length at birth

Fixed

	MMS			IFA				Mean Difference
Study or Subgroup	Mean	SD	Total	Mean	SD	Total	Weight	IV, Fixed, 95% CI
Adu-Afarwuah 2015	48.5	2	313	48.3	1.9	305	1.0%	0.20 [-0.11, 0.51]
Ashorn 2010	49.7	2.2	376	49.5	2.4	366	0.9%	0.20 [-0.13, 0.53]
Bhutta 2009	48.3	3.1	574	48.4	2.8	615	0.8%	-0.10 [-0.44, 0.24]
Christian 2003	47.4	2.31	588	47.4	2.48	529	1.2%	0.00 [-0.28, 0.28]
Fawzi 2007	47.5	5.6	2799	47.3	5.6	2794	1.1%	0.20 [-0.09, 0.49]
Friis 2004	48.8	3.3929	564	48.5	3.3929	542	0.6%	0.30 [-0.10, 0.70]
Liu 2013	50	1	5913	50	1.1	5922	66.9%	0.00 [-0.04, 0.04]
Osrin 2005	48.9	2.9	526	48.6	3.2	517	0.7%	0.30 [-0.07, 0.67]
Ramakrishnan 2003	48.61	1.82	307	48.66	1.83	300	1.1%	-0.05 [-0.34, 0.24]
Roberfroid 2008	48.3	2.5	534	48	2.2	539	1.2%	0.30 [0.02, 0.58]
Sunawang 2009	47.6	2.1	632	47.6	2	649	1.9%	0.00 [-0.22, 0.22]
Tofail 2008 (1)	47.5	2.8	486	47.6	2.2	1007	1.2%	-0.10 [-0.38, 0.18]
West 2014	46.7	2.1	6964	46.5	2.1	6861	19.6%	0.20 [0.13, 0.27]
Zeng 2008	49.1	2.8	993	49.1	2.7	1031	1.7%	0.00 [-0.24, 0.24]
**Total (95% CI)**			**21569**			**21977**	**100.0%**	**0.05 [0.02, 0.08]**

Heterogeneity: Chi² = 36.07, df = 13 (P = 0.0006); I² = 64%
Test for overall effect: Z = 3.17 (P = 0.002)

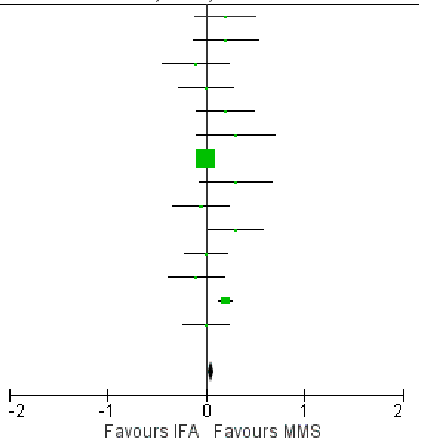

Footnotes
(1) Only the Usual Invitation to Food Supplementation groups were included. Both IFA arms were merged and compared with the MMS arm

Random

	MMS			IFA				Mean Difference
Study or Subgroup	Mean	SD	Total	Mean	SD	Total	Weight	IV, Random, 95% CI
Adu-Afarwuah 2015	48.5	2	313	48.3	1.9	305	5.4%	0.20 [-0.11, 0.51]
Ashorn 2010	49.7	2.2	376	49.5	2.4	366	4.9%	0.20 [-0.13, 0.53]
Bhutta 2009	48.3	3.1	574	48.4	2.8	615	4.8%	-0.10 [-0.44, 0.24]
Christian 2003	47.4	2.31	588	47.4	2.48	529	6.1%	0.00 [-0.28, 0.28]
Fawzi 2007	47.5	5.6	2799	47.3	5.6	2794	5.8%	0.20 [-0.09, 0.49]
Friis 2004	48.8	3.3929	564	48.5	3.3929	542	3.7%	0.30 [-0.10, 0.70]
Liu 2013	50	1	5913	50	1.1	5922	16.7%	0.00 [-0.04, 0.04]
Osrin 2005	48.9	2.9	526	48.6	3.2	517	4.1%	0.30 [-0.07, 0.67]
Ramakrishnan 2003	48.61	1.82	307	48.66	1.83	300	5.8%	-0.05 [-0.34, 0.24]
Roberfroid 2008	48.3	2.5	534	48	2.2	539	6.1%	0.30 [0.02, 0.58]
Sunawang 2009	47.6	2.1	632	47.6	2	649	7.9%	0.00 [-0.22, 0.22]
Tofail 2008 (1)	47.5	2.8	486	47.6	2.2	1007	6.0%	-0.10 [-0.38, 0.18]
West 2014	46.7	2.1	6964	46.5	2.1	6861	15.5%	0.20 [0.13, 0.27]
Zeng 2008	49.1	2.8	993	49.1	2.7	1031	7.4%	0.00 [-0.24, 0.24]
**Total (95% CI)**			**21569**			**21977**	**100.0%**	**0.09 [0.00, 0.18]**

Heterogeneity: Tau² = 0.01; Chi² = 36.07, df = 13 (P = 0.0006); I² = 64%
Test for overall effect: Z = 2.06 (P = 0.04)

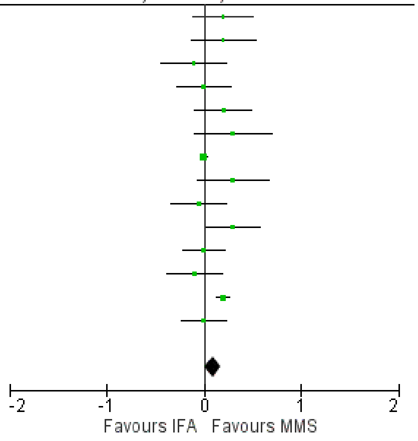

Footnotes
(1) Only the Usual Invitation to Food Supplementation groups were included. Both IFA arms were merged and compared with the MMS arm

Supplemental Figure 2.1.2. Effect of MMS vs IFA on length at 3 months

Fixed

	MMS			IFA				Mean Difference
Study or Subgroup	Mean	SD	Total	Mean	SD	Total	Weight	IV, Fixed, 95% CI
Adu-Afarwuah 2015	59.6	2.3	357	59.3	2.2	343	3.7%	0.30 [-0.03, 0.63]
Fawzi 2007	59.84	3.15	2928	59.74	2.98	2920	16.6%	0.10 [-0.06, 0.26]
Moore 2009	60.2	2.4	179	60.1	2.1	171	1.8%	0.10 [-0.37, 0.57]
Roberfroid 2008	58.9	2.9	306	58.9	2.8	349	2.1%	0.00 [-0.44, 0.44]
Sunawang 2009	59.2	2.5	151	59.1	2.2	148	1.4%	0.10 [-0.43, 0.63]
Tofail 2008 (1)	57.6	2.3	375	57.8	2.4	786	5.0%	-0.20 [-0.49, 0.09]
West 2014	58.13	2.42	7931	57.9	2.49	7725	69.3%	0.23 [0.15, 0.31]
**Total (95% CI)**			**12227**			**12442**	**100.0%**	**0.18 [0.12, 0.24]**

Heterogeneity: Chi² = 10.70, df = 6 (P = 0.10); I² = 44%
Test for overall effect: Z = 5.52 (P < 0.00001)

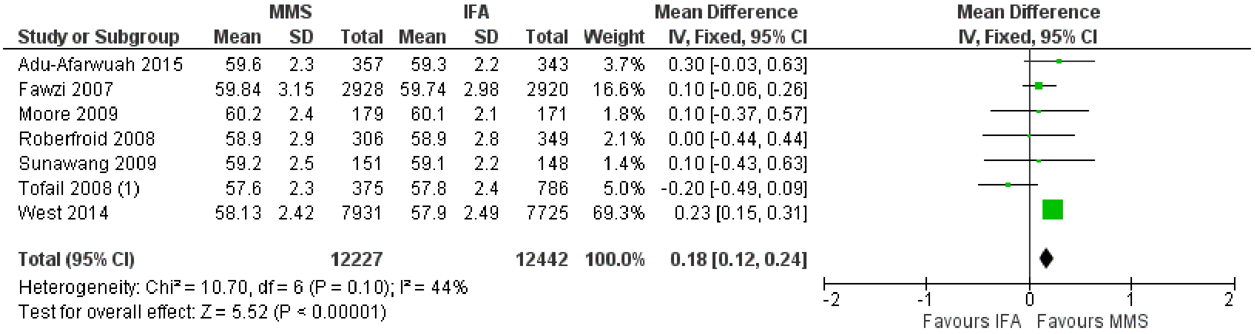

Footnotes
(1) Only the Usual Invitation to Food Supplementation groups were included. Both IFA arms were merged and compared with the MMS arm

Random

	MMS			IFA				Mean Difference
Study or Subgroup	Mean	SD	Total	Mean	SD	Total	Weight	IV, Random, 95% CI
Adu-Afarwuah 2015	59.6	2.3	357	59.3	2.2	343	10.4%	0.30 [-0.03, 0.63]
Fawzi 2007	59.84	3.15	2928	59.74	2.98	2920	24.5%	0.10 [-0.06, 0.26]
Moore 2009	60.2	2.4	179	60.1	2.1	171	6.0%	0.10 [-0.37, 0.57]
Roberfroid 2008	58.9	2.9	306	58.9	2.8	349	6.8%	0.00 [-0.44, 0.44]
Sunawang 2009	59.2	2.5	151	59.1	2.2	148	4.8%	0.10 [-0.43, 0.63]
Tofail 2008 (1)	57.6	2.3	375	57.8	2.4	786	12.9%	-0.20 [-0.49, 0.09]
West 2014	58.13	2.42	7931	57.9	2.49	7725	34.6%	0.23 [0.15, 0.31]
**Total (95% CI)**			**12227**			**12442**	**100.0%**	**0.12 [-0.01, 0.25]**

Heterogeneity: Tau² = 0.01; Chi² = 10.70, df = 6 (P = 0.10); I² = 44%
Test for overall effect: Z = 1.88 (P = 0.06)

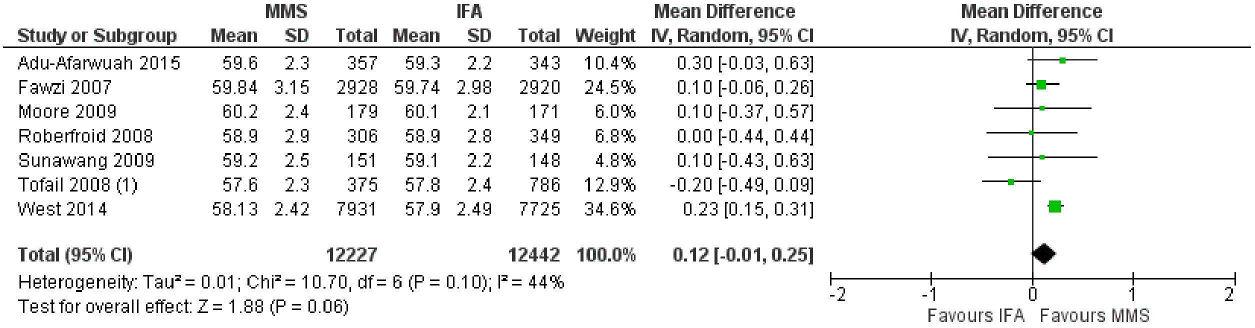

Footnotes
(1) Only the Usual Invitation to Food Supplementation groups were included. Both IFA arms were merged and compared with the MMS arm

Supplemental Figure 2.1.3. Effect of MMS vs IFA on length at 6 months

Fixed

	MMS			IFA				Mean Difference
**Study or Subgroup**	**Mean**	**SD**	**Total**	**Mean**	**SD**	**Total**	**Weight**	**IV, Fixed, 95% CI**
Adu-Afarwuah 2015	65.3	2.5	355	65.1	2.3	347	4.4%	0.20 [-0.16, 0.56]
Ashorn 2010 complete FU	64.2	2.6	213	64	2.8	220	2.1%	0.20 [-0.31, 0.71]
Ashorn 2010 simplified FU	64.2	2.7	131	64.6	2.7	130	1.3%	-0.40 [-1.06, 0.26]
Fawzi 2007	65.05	2.76	3008	65.06	2.88	2990	27.0%	-0.01 [-0.15, 0.13]
Moore 2009	65.1	2.6	170	65.1	2.4	172	2.0%	0.00 [-0.53, 0.53]
Roberfroid 2008	64.9	2.5	270	64.7	2.6	313	3.2%	0.20 [-0.21, 0.61]
Sunawang 2009	64.8	2.4	151	64.8	2.4	148	1.9%	0.00 [-0.54, 0.54]
Tofail 2008 (1)	63.5	2.5	449	63.5	2.5	888	6.8%	0.00 [-0.28, 0.28]
West 2014	64.2	2.49	4494	64.06	2.5	4429	51.4%	0.14 [0.04, 0.24]
**Total (95% CI)**			**9241**			**9637**	**100.0%**	**0.08 [0.01, 0.16]**

Heterogeneity: Chi² = 6.32, df = 8 (P = 0.61); I² = 0%
Test for overall effect: Z = 2.20 (P = 0.03)

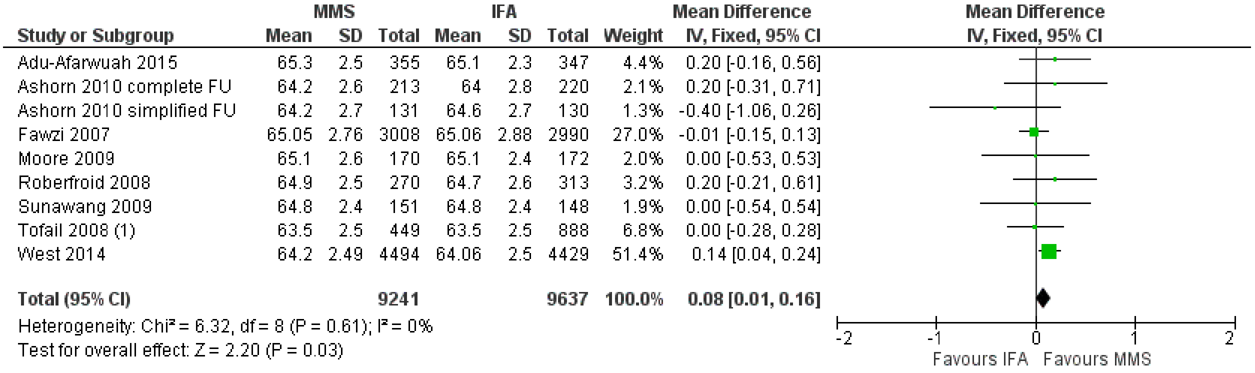

Footnotes
(1) Only the Usual Invitation to Food Supplementation groups were included. Both IFA arms were merged and compared with the MMS arm

Random

	MMS			IFA				Mean Difference
**Study or Subgroup**	**Mean**	**SD**	**Total**	**Mean**	**SD**	**Total**	**Weight**	**IV, Random, 95% CI**
Adu-Afarwuah 2015	65.3	2.5	355	65.1	2.3	347	4.4%	0.20 [-0.16, 0.56]
Ashorn 2010 complete FU	64.2	2.6	213	64	2.8	220	2.1%	0.20 [-0.31, 0.71]
Ashorn 2010 simplified FU	64.2	2.7	131	64.6	2.7	130	1.3%	-0.40 [-1.06, 0.26]
Fawzi 2007	65.05	2.76	3008	65.06	2.88	2990	27.0%	-0.01 [-0.15, 0.13]
Moore 2009	65.1	2.6	170	65.1	2.4	172	2.0%	0.00 [-0.53, 0.53]
Roberfroid 2008	64.9	2.5	270	64.7	2.6	313	3.2%	0.20 [-0.21, 0.61]
Sunawang 2009	64.8	2.4	151	64.8	2.4	148	1.9%	0.00 [-0.54, 0.54]
Tofail 2008 (1)	63.5	2.5	449	63.5	2.5	888	6.8%	0.00 [-0.28, 0.28]
West 2014	64.2	2.49	4494	64.06	2.5	4429	51.4%	0.14 [0.04, 0.24]
**Total (95% CI)**			**9241**			**9637**	**100.0%**	**0.08 [0.01, 0.16]**

Heterogeneity: Tau² = 0.00; Chi² = 6.32, df = 8 (P = 0.61); I² = 0%
Test for overall effect: Z = 2.20 (P = 0.03)

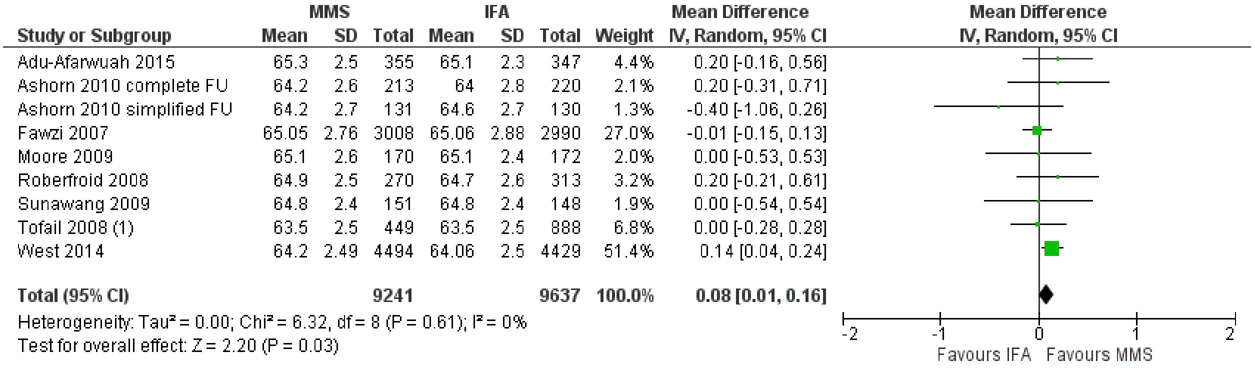

Footnotes
(1) Only the Usual Invitation to Food Supplementation groups were included. Both IFA arms were merged and compared with the MMS arm

Supplemental Figure 2.1.4. Effect of MMS vs IFA on length at 12 months

Fixed

	MMS			IFA				Mean Difference
Study or Subgroup	Mean	SD	Total	Mean	SD	Total	Weight	IV, Fixed, 95% CI
Adu-Afarwuah 2015	73.3	2.8	345	73	5.6	332	1.7%	0.30 [-0.37, 0.97]
Ashorn 2010 complete FU	71.3	3	214	71.4	2.8	218	2.6%	-0.10 [-0.65, 0.45]
Fawzi 2007	72.31	3.32	1635	72.31	3.24	1615	15.2%	0.00 [-0.23, 0.23]
Roberfroid 2008	71.5	2.8	240	71.7	2.8	248	3.1%	-0.20 [-0.70, 0.30]
Sunawang 2009	71.8	2.6	151	71.8	2.7	148	2.1%	0.00 [-0.60, 0.60]
Tofail 2008 (1)	70.6	2.7	504	70.7	2.8	1028	9.1%	-0.10 [-0.39, 0.19]
West 2014	70.98	2.72	4851	70.88	2.69	4785	66.2%	0.10 [-0.01, 0.21]
**Total (95% CI)**			**7940**			**8374**	**100.0%**	**0.05 [-0.03, 0.14]**

Heterogeneity: Chi² = 3.85, df = 6 (P = 0.70); I² = 0%
Test for overall effect: Z = 1.19 (P = 0.23)

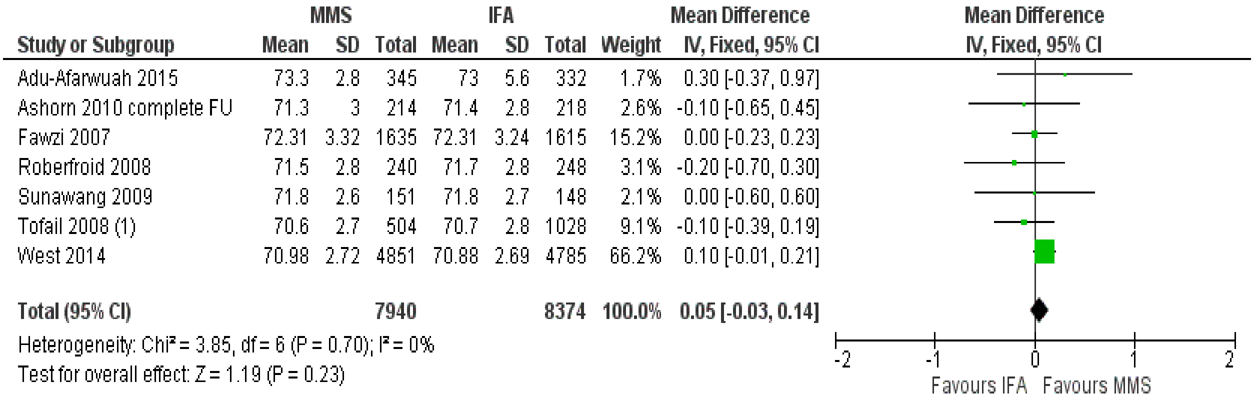

Footnotes
(1) Only the Usual Invitation to Food Supplementation groups were included. Both IFA arms were merged and compared with the MMS arm

Random

	MMS			IFA				Mean Difference
Study or Subgroup	Mean	SD	Total	Mean	SD	Total	Weight	IV, Random, 95% CI
Adu-Afarwuah 2015	73.3	2.8	345	73	5.6	332	1.7%	0.30 [-0.37, 0.97]
Ashorn 2010 complete FU	71.3	3	214	71.4	2.8	218	2.6%	-0.10 [-0.65, 0.45]
Fawzi 2007	72.31	3.32	1635	72.31	3.24	1615	15.2%	0.00 [-0.23, 0.23]
Roberfroid 2008	71.5	2.8	240	71.7	2.8	248	3.1%	-0.20 [-0.70, 0.30]
Sunawang 2009	71.8	2.6	151	71.8	2.7	148	2.1%	0.00 [-0.60, 0.60]
Tofail 2008 (1)	70.6	2.7	504	70.7	2.8	1028	9.1%	-0.10 [-0.39, 0.19]
West 2014	70.98	2.72	4851	70.88	2.69	4785	66.2%	0.10 [-0.01, 0.21]
**Total (95% CI)**			**7940**			**8374**	**100.0%**	**0.05 [-0.03, 0.14]**

Heterogeneity: Tau² = 0.00; Chi² = 3.85, df = 6 (P = 0.70); I² = 0%
Test for overall effect: Z = 1.19 (P = 0.23)

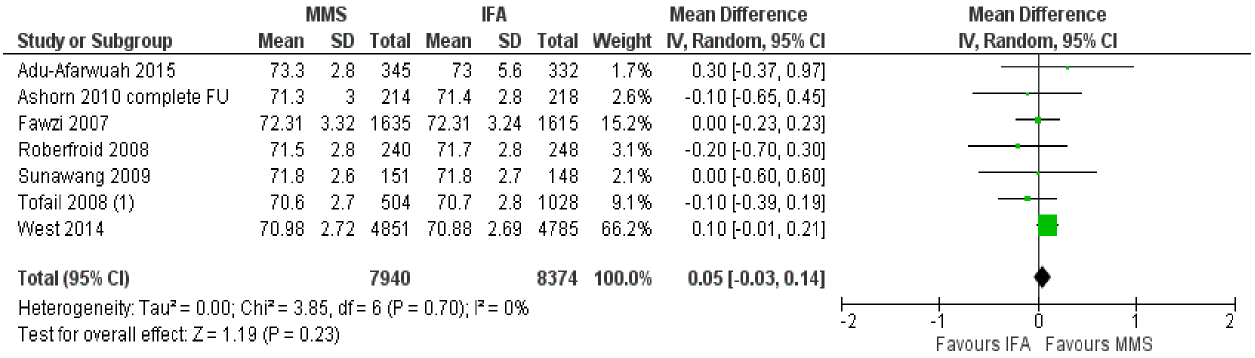

Footnotes
(1) Only the Usual Invitation to Food Supplementation groups were included. Both IFA arms were merged and compared with the MMS arm

Supplemental Figure 2.1.5. Effect of MMS vs IFA on length at 18 months

Fixed

	MMS			IFA				Mean Difference
**Study or Subgroup**	**Mean**	**SD**	**Total**	**Mean**	**SD**	**Total**	**Weight**	**IV, Fixed, 95% CI**
Adu-Afarwuah 2015	79.3	3	355	78.9	2.8	340	28.4%	0.40 [-0.03, 0.83]
Ashorn 2010 complete FU	76.9	3.5	222	77	3	220	14.3%	-0.10 [-0.71, 0.51]
Ashorn 2010 simplified FU	76.3	2.7	129	76.4	2.8	130	11.8%	-0.10 [-0.77, 0.57]
Fawzi 2007	77.78	2.94	39	76.93	3.26	55	3.3%	0.85 [-0.41, 2.11]
Tofail 2008 (1)	76	3.2	465	75.8	3.2	982	42.3%	0.20 [-0.15, 0.55]
**Total (95% CI)**			**1210**			**1727**	**100.0%**	**0.20 [-0.03, 0.43]**

Heterogeneity: Chi² = 3.55, df = 4 (P = 0.47); I² = 0%
Test for overall effect: Z = 1.71 (P = 0.09)

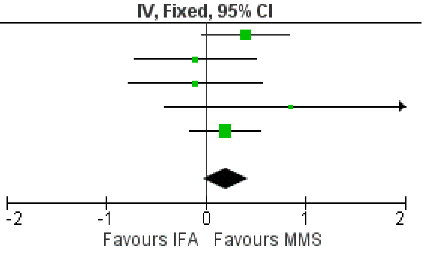

Footnotes
(1) Only the Usual Invitation to Food Supplementation groups were included. Both IFA arms were merged and compared with the MMS arm

Random

	MMS			IFA				Mean Difference
**Study or Subgroup**	**Mean**	**SD**	**Total**	**Mean**	**SD**	**Total**	**Weight**	**IV, Random, 95% CI**
Adu-Afarwuah 2015	79.3	3	355	78.9	2.8	340	28.4%	0.40 [-0.03, 0.83]
Ashorn 2010 complete FU	76.9	3.5	222	77	3	220	14.3%	-0.10 [-0.71, 0.51]
Ashorn 2010 simplified FU	76.3	2.7	129	76.4	2.8	130	11.8%	-0.10 [-0.77, 0.57]
Fawzi 2007	77.78	2.94	39	76.93	3.26	55	3.3%	0.85 [-0.41, 2.11]
Tofail 2008 (1)	76	3.2	465	75.8	3.2	982	42.3%	0.20 [-0.15, 0.55]
**Total (95% CI)**			**1210**			**1727**	**100.0%**	**0.20 [-0.03, 0.43]**

Heterogeneity: Tau² = 0.00; Chi² = 3.55, df = 4 (P = 0.47); I² = 0%
Test for overall effect: Z = 1.71 (P = 0.09)

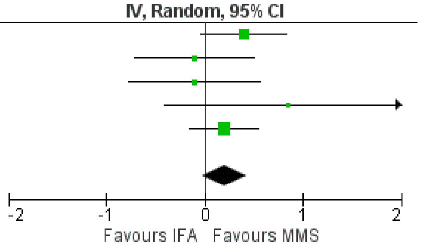

Footnotes
(1) Only the Usual Invitation to Food Supplementation groups were included. Both IFA arms were merged and compared with the MMS arm

Supplemental Figure 2.1.6. Effect of MMS vs IFA on length at 24 months

Fixed

	MMS			IFA				Mean Difference
**Study or Subgroup**	**Mean**	**SD**	**Total**	**Mean**	**SD**	**Total**	**Weight**	**IV, Fixed, 95% CI**
Ashorn 2010 complete FU	81.2	3.7	179	81.2	3.2	197	4.0%	0.00 [-0.70, 0.70]
Roberfroid 2008	81.5	4.4	54	81.6	4.6	55	0.7%	-0.10 [-1.79, 1.59]
Tofail 2008 (1)	80.3	3.4	480	80.5	3.5	971	14.1%	-0.20 [-0.58, 0.18]
West 2014	80.51	3.21	3283	80.55	3.22	3231	81.2%	-0.04 [-0.20, 0.12]
**Total (95% CI)**			**3996**			**4454**	**100.0%**	**-0.06 [-0.20, 0.08]**

Heterogeneity: Chi² = 0.63, df = 3 (P = 0.89); I² = 0%
Test for overall effect: Z = 0.85 (P = 0.39)

Mean Difference
IV, Fixed, 95% CI

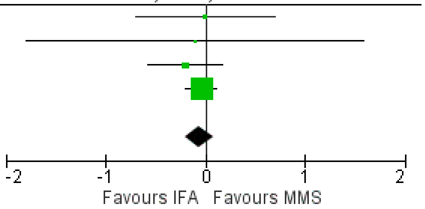

Footnotes
(1) Only the Usual Invitation to Food Supplementation groups were included. Both IFA arms were merged and compared with the MMS arm

Random

	MMS			IFA				Mean Difference
**Study or Subgroup**	**Mean**	**SD**	**Total**	**Mean**	**SD**	**Total**	**Weight**	**IV, Random, 95% CI**
Ashorn 2010 complete FU	81.2	3.7	179	81.2	3.2	197	4.0%	0.00 [-0.70, 0.70]
Roberfroid 2008	81.5	4.4	54	81.6	4.6	55	0.7%	-0.10 [-1.79, 1.59]
Tofail 2008 (1)	80.3	3.4	480	80.5	3.5	971	14.1%	-0.20 [-0.58, 0.18]
West 2014	80.51	3.21	3283	80.55	3.22	3231	81.2%	-0.04 [-0.20, 0.12]
**Total (95% CI)**			**3996**			**4454**	**100.0%**	**-0.06 [-0.20, 0.08]**

Heterogeneity: Tau² = 0.00; Chi² = 0.63, df = 3 (P = 0.89); I² = 0%
Test for overall effect: Z = 0.85 (P = 0.39)

Mean Difference
IV, Random, 95% CI

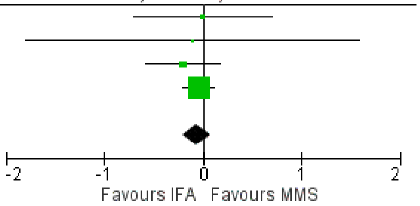

Footnotes
(1) Only the Usual Invitation to Food Supplementation groups were included. Both IFA arms were merged and compared with the MMS arm

Supplemental Figure 2.2. Effect of MMS vs IFA on weight

Supplemental Figure 2.2.1. Effect of MMS vs IFA on weight at birth

Fixed

	MMS			IFA				Mean Difference
**Study or Subgroup**	**Mean**	**SD**	**Total**	**Mean**	**SD**	**Total**	**Weight**	**IV, Fixed, 95% CI**
Adu-Afarwuah 2015	3.005	0.435	318	2.945	0.442	305	1.0%	0.06 [-0.01, 0.13]
Ashorn 2010	2.964	0.46	374	2.948	0.432	362	1.1%	0.02 [-0.05, 0.08]
Bhutta 2009	2.95	0.6	574	2.88	0.5	615	1.2%	0.07 [0.01, 0.13]
Christian 2003	2.659	0.446	588	2.652	0.436	529	1.7%	0.01 [-0.04, 0.06]
Fawzi 2007	3.14	0.52	3848	3.08	0.52	2833	7.3%	0.06 [0.03, 0.09]
Friis 2004	3.053	0.9246	564	3.004	0.9246	542	0.4%	0.05 [-0.06, 0.16]
Kaestel 2005 (1)	3.076	0.501	734	3.002	0.486	366	1.2%	0.07 [0.01, 0.14]
Liu 2013	3.299	0.3924	5913	3.293	0.389	5922	23.6%	0.01 [-0.01, 0.02]
Moore 2009	3.009	0.471	178	3.013	0.523	167	0.4%	-0.00 [-0.11, 0.10]
Osrin 2005	2.81	0.453	529	2.733	0.422	523	1.7%	0.08 [0.02, 0.13]
Ramakrishnan 2003	2.981	0.391	318	2.977	0.393	315	1.3%	0.00 [-0.06, 0.07]
Roberfroid 2008	2.9	0.459	535	2.872	0.416	541	1.7%	0.03 [-0.02, 0.08]
SUMMIT 2008	3.198	0.8867	5695	3.176	0.8867	5406	4.3%	0.02 [-0.01, 0.06]
Sunawang 2009	3.086	0.423	632	3.083	0.349	649	2.6%	0.00 [-0.04, 0.05]
Tofail 2008 (2)	2.637	0.43	490	2.62	0.433	1013	2.2%	0.02 [-0.03, 0.06]
West 2014	2.584	0.402	7127	2.533	0.407	7041	26.3%	0.05 [0.04, 0.06]
Zagre 2007	3.091	0.2059	1328	3.024	0.2059	1222	18.3%	0.07 [0.05, 0.08]
Zeng 2008	3.198	0.438	1116	3.174	0.424	1167	3.7%	0.02 [-0.01, 0.06]
**Total (95% CI)**			**30861**			**29518**	**100.0%**	**0.04 [0.03, 0.05]**

Heterogeneity: $Chi^2 = 52.52$, df = 17 ($P < 0.0001$); $I^2 = 68\%$
Test for overall effect: $Z = 11.01$ ($P < 0.00001$)

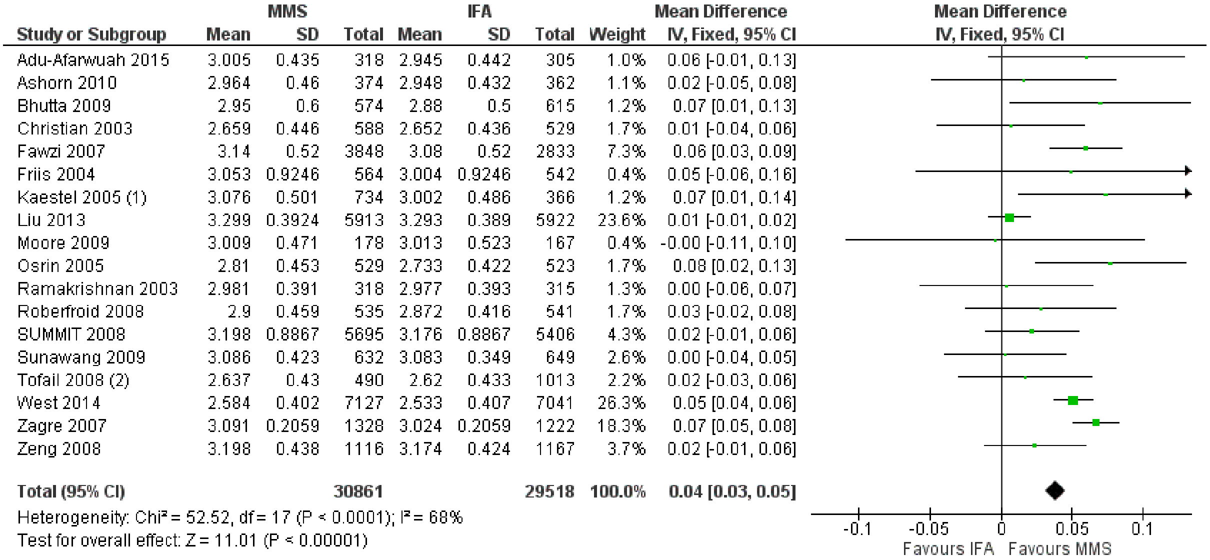

Footnotes
(1) Two MMS groups were combined by creating a single pairwise comparison as per the Cochrane guidance
(2) Only the Usual Invitation to Food Supplementation groups were included. Both IFA arms were merged and compared with the MMS arm

Random

	MMS			IFA				Mean Difference
**Study or Subgroup**	**Mean**	**SD**	**Total**	**Mean**	**SD**	**Total**	**Weight**	**IV, Random, 95% CI**
Adu-Afarwuah 2015	3.005	0.435	318	2.945	0.442	305	3.3%	0.06 [-0.01, 0.13]
Ashorn 2010	2.964	0.46	374	2.948	0.432	362	3.6%	0.02 [-0.05, 0.08]
Bhutta 2009	2.95	0.6	574	2.88	0.5	615	3.7%	0.07 [0.01, 0.13]
Christian 2003	2.659	0.446	588	2.652	0.436	529	4.8%	0.01 [-0.04, 0.06]
Fawzi 2007	3.14	0.52	3848	3.08	0.52	2833	8.5%	0.06 [0.03, 0.09]
Friis 2004	3.053	0.9246	564	3.004	0.9246	542	1.6%	0.05 [-0.06, 0.16]
Kaestel 2005 (1)	3.076	0.501	734	3.002	0.486	366	3.9%	0.07 [0.01, 0.14]
Liu 2013	3.299	0.3924	5913	3.293	0.389	5922	10.2%	0.01 [-0.01, 0.02]
Moore 2009	3.009	0.471	178	3.013	0.523	167	1.7%	-0.00 [-0.11, 0.10]
Osrin 2005	2.81	0.453	529	2.733	0.422	523	4.7%	0.08 [0.02, 0.13]
Ramakrishnan 2003	2.981	0.391	318	2.977	0.393	315	3.9%	0.00 [-0.06, 0.07]
Roberfroid 2008	2.9	0.459	535	2.872	0.416	541	4.7%	0.03 [-0.02, 0.08]
SUMMIT 2008	3.198	0.8867	5695	3.176	0.8867	5406	7.2%	0.02 [-0.01, 0.06]
Sunawang 2009	3.086	0.423	632	3.083	0.349	649	5.9%	0.00 [-0.04, 0.05]
Tofail 2008 (2)	2.637	0.43	490	2.62	0.433	1013	5.4%	0.02 [-0.03, 0.06]
West 2014	2.584	0.402	7127	2.533	0.407	7041	10.3%	0.05 [0.04, 0.06]
Zagre 2007	3.091	0.2059	1328	3.024	0.2059	1222	9.9%	0.07 [0.05, 0.08]
Zeng 2008	3.198	0.438	1116	3.174	0.424	1167	6.9%	0.02 [-0.01, 0.06]
**Total (95% CI)**			**30861**			**29518**	**100.0%**	**0.04 [0.02, 0.05]**

Heterogeneity: $Tau^2 = 0.00$; $Chi^2 = 52.52$, df = 17 ($P < 0.0001$); $I^2 = 68\%$
Test for overall effect: $Z = 4.73$ ($P < 0.00001$)

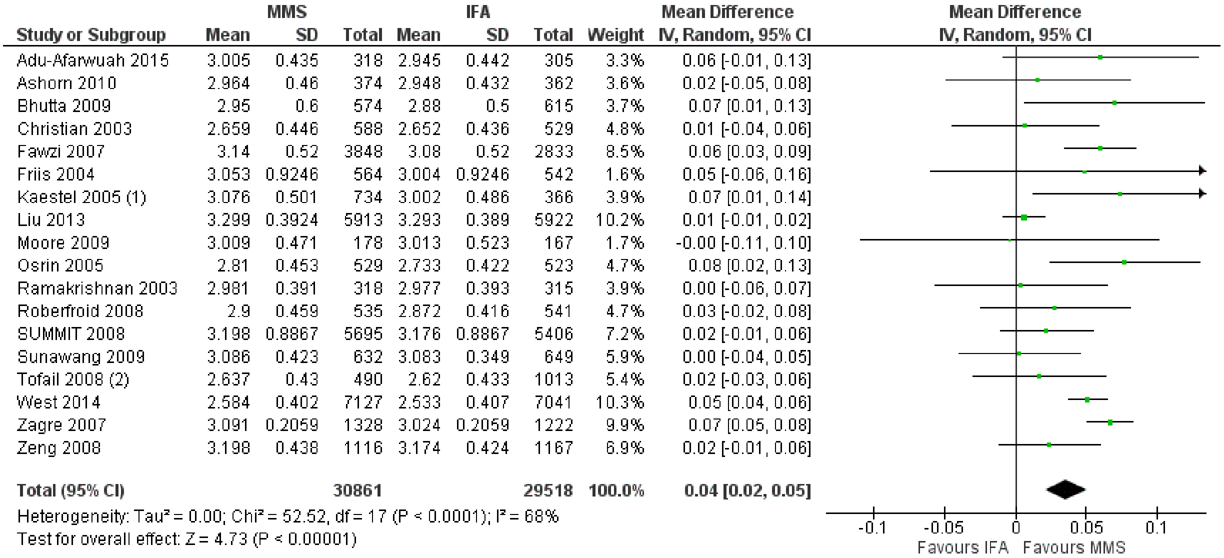

Footnotes
(1) Two MMS groups were combined by creating a single pairwise comparison as per the Cochrane guidance
(2) Only the Usual Invitation to Food Supplementation groups were included. Both IFA arms were merged and compared with the MMS arm

Supplemental Figure 2.2.2. Effect of MMS vs IFA on weight at 3 months

Fixed

Study or Subgroup	MMS Mean	MMS SD	MMS Total	IFA Mean	IFA SD	IFA Total	Weight	Mean Difference IV, Fixed, 95% CI
Adu-Afarwuah 2015	6.05	0.83	358	5.93	0.84	343	2.3%	0.12 [-0.00, 0.24]
Fawzi 2007	6.4	0.87	3019	6.13	0.85	3020	18.7%	0.27 [0.23, 0.31]
Moore 2009	5.826	0.762	179	5.827	0.776	171	1.4%	-0.00 [-0.16, 0.16]
Roberfroid 2008	5.77	0.816	306	5.698	0.825	349	2.2%	0.07 [-0.05, 0.20]
Sunawang 2009	5.76	0.74	151	5.71	0.74	148	1.3%	0.05 [-0.12, 0.22]
Tofail 2008 (1)	5.27	0.73	376	5.28	0.75	797	4.3%	-0.01 [-0.10, 0.08]
West 2014	5.26	0.72	7996	5.18	0.72	7777	69.8%	0.08 [0.06, 0.10]
**Total (95% CI)**			**12385**			**12605**	**100.0%**	**0.11 [0.09, 0.13]**

Heterogeneity: Chi² = 68.55, df = 6 (P < 0.00001); I² = 91%
Test for overall effect: Z = 11.58 (P < 0.00001)

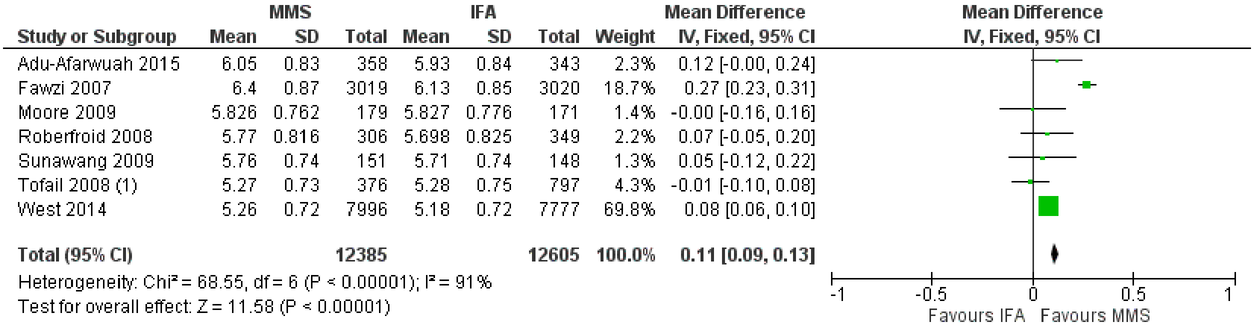

Footnotes
(1) Only the Usual Invitation to Food Supplementation groups were included. Both IFA arms were merged and compared with the MMS arm

Random

Study or Subgroup	MMS Mean	MMS SD	MMS Total	IFA Mean	IFA SD	IFA Total	Weight	Mean Difference IV, Random, 95% CI
Adu-Afarwuah 2015	6.05	0.83	358	5.93	0.84	343	13.5%	0.12 [-0.00, 0.24]
Fawzi 2007	6.4	0.87	3019	6.13	0.85	3020	17.3%	0.27 [0.23, 0.31]
Moore 2009	5.826	0.762	179	5.827	0.776	171	11.5%	-0.00 [-0.16, 0.16]
Roberfroid 2008	5.77	0.816	306	5.698	0.825	349	13.4%	0.07 [-0.05, 0.20]
Sunawang 2009	5.76	0.74	151	5.71	0.74	148	11.2%	0.05 [-0.12, 0.22]
Tofail 2008 (1)	5.27	0.73	376	5.28	0.75	797	15.3%	-0.01 [-0.10, 0.08]
West 2014	5.26	0.72	7996	5.18	0.72	7777	17.8%	0.08 [0.06, 0.10]
**Total (95% CI)**			**12385**			**12605**	**100.0%**	**0.09 [-0.00, 0.18]**

Heterogeneity: Tau² = 0.01; Chi² = 68.55, df = 6 (P < 0.00001); I² = 91%
Test for overall effect: Z = 1.94 (P = 0.05)

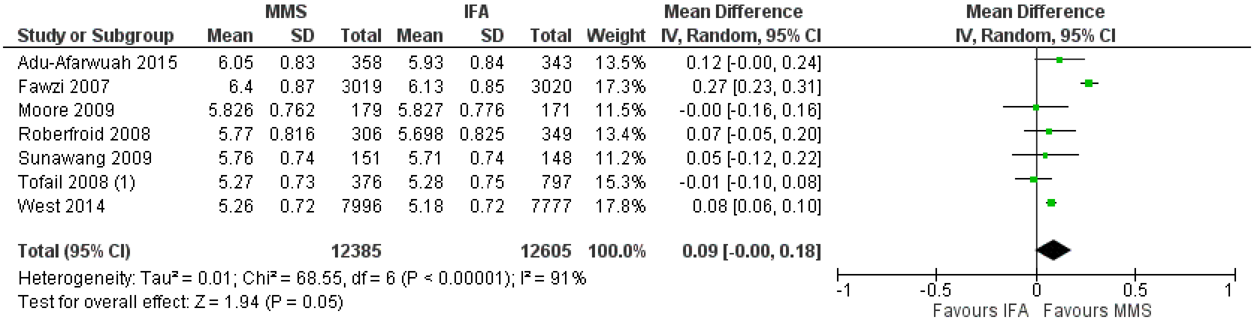

Footnotes
(1) Only the Usual Invitation to Food Supplementation groups were included. Both IFA arms were merged and compared with the MMS arm

Supplemental Figure 2.2.3. Effect of MMS vs IFA on weight at 6 months

Fixed

Study or Subgroup	MMS Mean	MMS SD	MMS Total	IFA Mean	IFA SD	IFA Total	Weight	Mean Difference IV, Fixed, 95% CI
Adu-Afarwuah 2015	7.3	0.99	356	7.19	1	347	3.1%	0.11 [-0.04, 0.26]
Ashorn 2010 complete FU	7.3	1.1	216	7.2	1.1	219	1.6%	0.10 [-0.11, 0.31]
Ashorn 2010 simplified FU	7.3	1.1	131	7.3	1	130	1.0%	0.00 [-0.26, 0.26]
Fawzi 2007	7.38	1.06	3055	7.36	1.03	3033	24.5%	0.02 [-0.03, 0.07]
Moore 2009	6.953	0.908	169	6.949	0.946	172	1.7%	0.00 [-0.19, 0.20]
Roberfroid 2008	6.918	0.1025	270	6.8	0.936	313	6.2%	0.12 [0.01, 0.22]
Sunawang 2009	7.05	0.84	151	7.05	0.91	148	1.7%	0.00 [-0.20, 0.20]
Tofail 2008 (1)	6.73	0.93	451	6.72	0.94	896	6.0%	0.01 [-0.10, 0.12]
West 2014	6.67	0.86	4539	6.63	0.85	4467	54.1%	0.04 [0.00, 0.08]
**Total (95% CI)**			**9338**			**9725**	**100.0%**	**0.04 [0.01, 0.07]**

Heterogeneity: Chi² = 4.58, df = 8 (P = 0.80); I² = 0%
Test for overall effect: Z = 2.98 (P = 0.003)

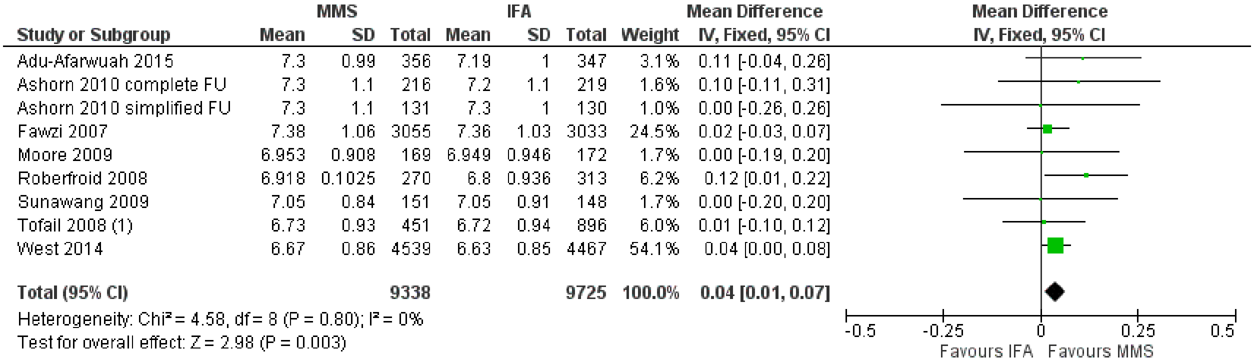

Footnotes
(1) Only the Usual Invitation to Food Supplementation groups were included. Both IFA arms were merged and compared with the MMS arm

Random

Study or Subgroup	MMS Mean	MMS SD	MMS Total	IFA Mean	IFA SD	IFA Total	Weight	Mean Difference IV, Random, 95% CI
Adu-Afarwuah 2015	7.3	0.99	356	7.19	1	347	3.1%	0.11 [-0.04, 0.26]
Ashorn 2010 complete FU	7.3	1.1	216	7.2	1.1	219	1.6%	0.10 [-0.11, 0.31]
Ashorn 2010 simplified FU	7.3	1.1	131	7.3	1	130	1.0%	0.00 [-0.26, 0.26]
Fawzi 2007	7.38	1.06	3055	7.36	1.03	3033	24.5%	0.02 [-0.03, 0.07]
Moore 2009	6.953	0.908	169	6.949	0.946	172	1.7%	0.00 [-0.19, 0.20]
Roberfroid 2008	6.918	0.1025	270	6.8	0.936	313	6.2%	0.12 [0.01, 0.22]
Sunawang 2009	7.05	0.84	151	7.05	0.91	148	1.7%	0.00 [-0.20, 0.20]
Tofail 2008 (1)	6.73	0.93	451	6.72	0.94	896	6.0%	0.01 [-0.10, 0.12]
West 2014	6.67	0.86	4539	6.63	0.85	4467	54.1%	0.04 [0.00, 0.08]
**Total (95% CI)**			**9338**			**9725**	**100.0%**	**0.04 [0.01, 0.07]**

Heterogeneity: Tau² = 0.00; Chi² = 4.58, df = 8 (P = 0.80); I² = 0%
Test for overall effect: Z = 2.98 (P = 0.003)

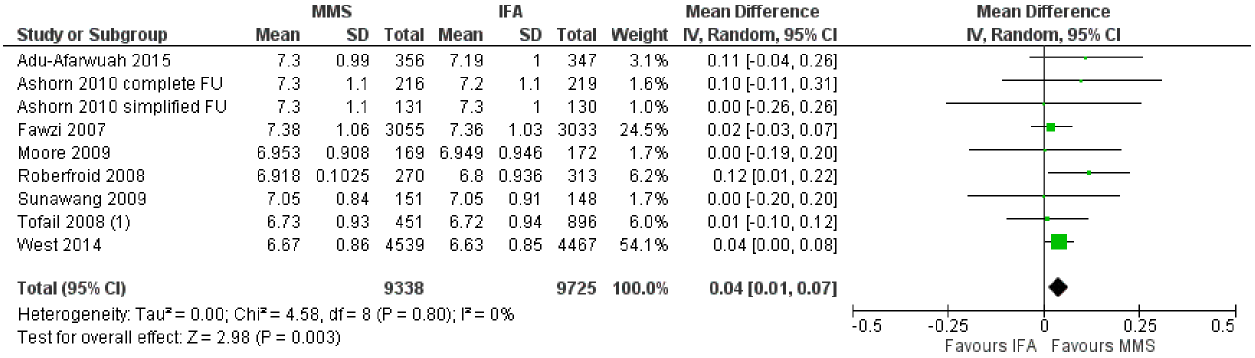

Footnotes
(1) Only the Usual Invitation to Food Supplementation groups were included. Both IFA arms were merged and compared with the MMS arm

Supplemental Figure 2.2.4. Effect of MMS vs IFA on weight at 12 months

Fixed

Study or Subgroup	MMS Mean	MMS SD	MMS Total	IFA Mean	IFA SD	IFA Total	Weight	Mean Difference IV, Fixed, 95% CI
Adu-Afarwuah 2015	8.69	1.13	346	8.52	1.11	332	3.4%	0.17 [0.00, 0.34]
Ashorn 2010 complete FU	8.5	1.2	215	8.6	1.1	219	2.1%	-0.10 [-0.32, 0.12]
Fawzi 2007	8.74	1.22	1646	8.72	1.23	1622	13.9%	0.02 [-0.06, 0.10]
Roberfroid 2008	7.876	0.98	240	7.878	0.98	248	3.2%	-0.00 [-0.18, 0.17]
Sunawang 2009	8.13	0.95	151	8.2	0.99	148	2.0%	-0.07 [-0.29, 0.15]
Tofail 2008 (1)	8.02	1.13	506	7.95	1.11	1033	6.8%	0.07 [-0.05, 0.19]
West 2014	7.77	0.95	4961	7.75	0.96	4867	68.5%	0.02 [-0.02, 0.06]
**Total (95% CI)**			**8065**			**8469**	**100.0%**	**0.02 [-0.01, 0.05]**

Heterogeneity: Chi² = 5.55, df = 6 (P = 0.48); I² = 0%
Test for overall effect: Z = 1.48 (P = 0.14)

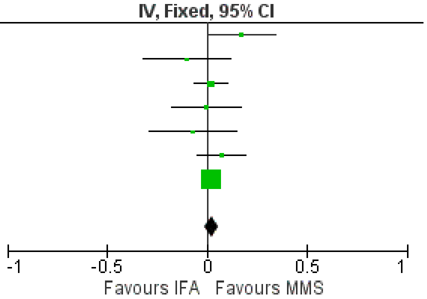

Footnotes
(1) Only the Usual Invitation to Food Supplementation groups were included. Both IFA arms were merged and compared with the MMS arm

Random

Study or Subgroup	MMS Mean	MMS SD	MMS Total	IFA Mean	IFA SD	IFA Total	Weight	Mean Difference IV, Random, 95% CI
Adu-Afarwuah 2015	8.69	1.13	346	8.52	1.11	332	3.4%	0.17 [0.00, 0.34]
Ashorn 2010 complete FU	8.5	1.2	215	8.6	1.1	219	2.1%	-0.10 [-0.32, 0.12]
Fawzi 2007	8.74	1.22	1646	8.72	1.23	1622	13.9%	0.02 [-0.06, 0.10]
Roberfroid 2008	7.876	0.98	240	7.878	0.98	248	3.2%	-0.00 [-0.18, 0.17]
Sunawang 2009	8.13	0.95	151	8.2	0.99	148	2.0%	-0.07 [-0.29, 0.15]
Tofail 2008 (1)	8.02	1.13	506	7.95	1.11	1033	6.8%	0.07 [-0.05, 0.19]
West 2014	7.77	0.95	4961	7.75	0.96	4867	68.5%	0.02 [-0.02, 0.06]
**Total (95% CI)**			**8065**			**8469**	**100.0%**	**0.02 [-0.01, 0.05]**

Heterogeneity: Tau² = 0.00; Chi² = 5.55, df = 6 (P = 0.48); I² = 0%
Test for overall effect: Z = 1.48 (P = 0.14)

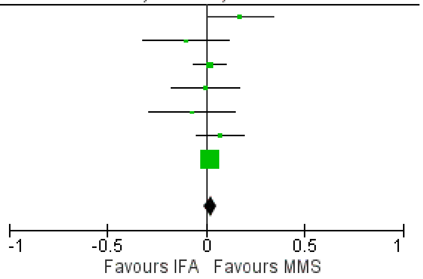

Footnotes
(1) Only the Usual Invitation to Food Supplementation groups were included. Both IFA arms were merged and compared with the MMS arm

Supplemental Figure 2.2.5. Effect of MMS vs IFA on weight at 18 months

Fixed

	MMS			IFA				Mean Difference	Mean Difference
Study or Subgroup	Mean	SD	Total	Mean	SD	Total	Weight	IV, Fixed, 95% CI	IV, Fixed, 95% CI
Adu-Afarwuah 2015	9.75	1.22	356	9.58	1.17	337	25.8%	0.17 [-0.01, 0.35]	
Ashorn 2010 complete FU	9.6	1.3	222	9.7	1.2	220	15.0%	-0.10 [-0.33, 0.13]	
Ashorn 2010 simplified FU	9.5	1.2	130	9.5	1.2	129	9.6%	0.00 [-0.29, 0.29]	
Fawzi 2007	9.57	1.49	40	9.49	1.45	55	2.3%	0.08 [-0.52, 0.68]	
Roberfroid 2008 (1)	0	0	0	0	0	0		Not estimable	
Sunawang 2009 (2)	0	0	0	0	0	0		Not estimable	
Tofail 2008 (3)	8.94	1.2	470	8.82	1.19	982	47.3%	0.12 [-0.01, 0.25]	
West 2014 (4)	0	0	0	0	0	0		Not estimable	
**Total (95% CI)**			**1218**			**1723**	**100.0%**	**0.09 [-0.00, 0.18]**	

Heterogeneity: Chi² = 3.89, df = 4 (P = 0.42); I² = 0%
Test for overall effect: Z = 1.89 (P = 0.06)

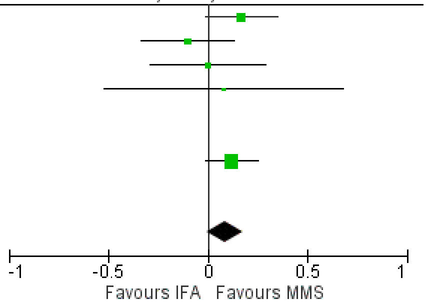

Footnotes
(1) Did not have a minimum of 30 participants per study arm (requirement to be included in the pooled analyses)
(2) No follow-up at 18m
(3) Only the Usual Invitation to Food Supplementation groups were included. Both IFA arms were merged and compared with the MMS arm
(4) No follow-up at 18m

Random

	MMS			IFA				Mean Difference	Mean Difference
Study or Subgroup	Mean	SD	Total	Mean	SD	Total	Weight	IV, Random, 95% CI	IV, Random, 95% CI
Adu-Afarwuah 2015	9.75	1.22	356	9.58	1.17	337	25.8%	0.17 [-0.01, 0.35]	
Ashorn 2010 complete FU	9.6	1.3	222	9.7	1.2	220	15.0%	-0.10 [-0.33, 0.13]	
Ashorn 2010 simplified FU	9.5	1.2	130	9.5	1.2	129	9.6%	0.00 [-0.29, 0.29]	
Fawzi 2007	9.57	1.49	40	9.49	1.45	55	2.3%	0.08 [-0.52, 0.68]	
Roberfroid 2008 (1)	0	0	0	0	0	0		Not estimable	
Sunawang 2009 (2)	0	0	0	0	0	0		Not estimable	
Tofail 2008 (3)	8.94	1.2	470	8.82	1.19	982	47.3%	0.12 [-0.01, 0.25]	
West 2014 (4)	0	0	0	0	0	0		Not estimable	
**Total (95% CI)**			**1218**			**1723**	**100.0%**	**0.09 [-0.00, 0.18]**	

Heterogeneity: Tau² = 0.00; Chi² = 3.89, df = 4 (P = 0.42); I² = 0%
Test for overall effect: Z = 1.89 (P = 0.06)

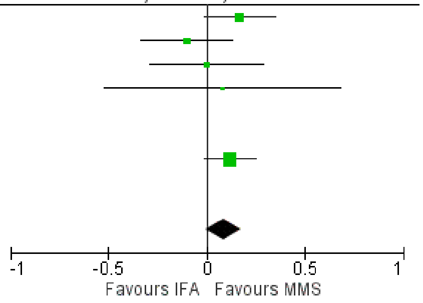

Footnotes
(1) Did not have a minimum of 30 participants per study arm (requirement to be included in the pooled analyses)
(2) No follow-up at 18m
(3) Only the Usual Invitation to Food Supplementation groups were included. Both IFA arms were merged and compared with the MMS arm
(4) No follow-up at 18m

Supplemental Figure 2.2.6. Effect of MMS vs IFA on weight at 24 months

Fixed

Study or Subgroup	MMS Mean	MMS SD	MMS Total	IFA Mean	IFA SD	IFA Total	Weight	Mean Difference IV, Fixed, 95% CI
Ashorn 2010 complete FU	10.5	1.4	177	10.6	1.2	196	3.3%	-0.10 [-0.37, 0.17]
Roberfroid 2008	10.1	1.3	54	10.1	1.5	55	0.8%	0.00 [-0.53, 0.53]
Tofail 2008 (1)	9.72	1.26	482	9.71	1.29	972	12.2%	0.01 [-0.13, 0.15]
West 2014	9.53	1.11	3412	9.52	1.11	3370	83.7%	0.01 [-0.04, 0.06]
**Total (95% CI)**			**4125**			**4593**	**100.0%**	**0.01 [-0.04, 0.05]**

Heterogeneity: Chi² = 0.64, df = 3 (P = 0.89); I² = 0%
Test for overall effect: Z = 0.25 (P = 0.80)

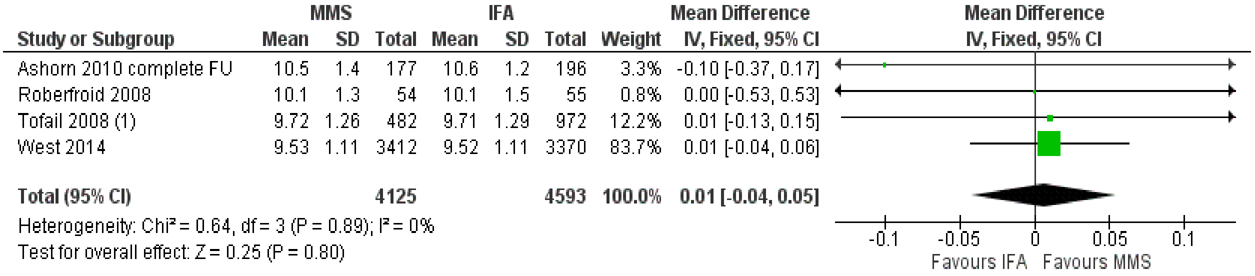

Footnotes
(1) Only the Usual Invitation to Food Supplementation groups were included. Both IFA arms were merged and compared with the MMS arm

Random

Study or Subgroup	MMS Mean	MMS SD	MMS Total	IFA Mean	IFA SD	IFA Total	Weight	Mean Difference IV, Random, 95% CI
Ashorn 2010 complete FU	10.5	1.4	177	10.6	1.2	196	3.3%	-0.10 [-0.37, 0.17]
Roberfroid 2008	10.1	1.3	54	10.1	1.5	55	0.8%	0.00 [-0.53, 0.53]
Tofail 2008 (1)	9.72	1.26	482	9.71	1.29	972	12.2%	0.01 [-0.13, 0.15]
West 2014	9.53	1.11	3412	9.52	1.11	3370	83.7%	0.01 [-0.04, 0.06]
**Total (95% CI)**			**4125**			**4593**	**100.0%**	**0.01 [-0.04, 0.05]**

Heterogeneity: Tau² = 0.00; Chi² = 0.64, df = 3 (P = 0.89); I² = 0%
Test for overall effect: Z = 0.25 (P = 0.80)

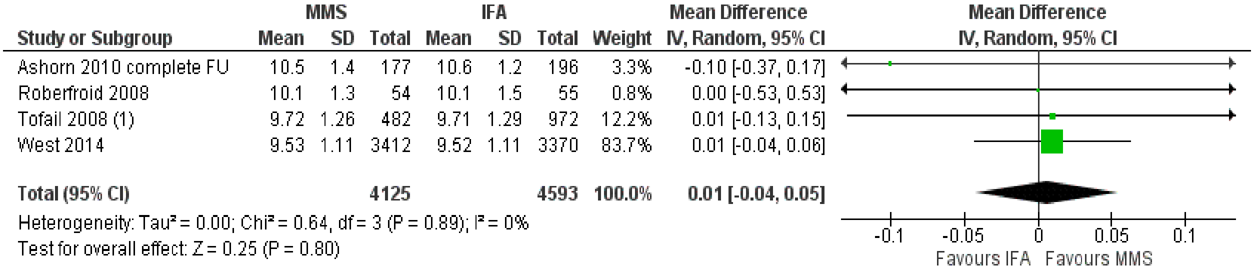

Footnotes
(1) Only the Usual Invitation to Food Supplementation groups were included. Both IFA arms were merged and compared with the MMS arm

Supplemental 2.3. Effect of MMS vs IFA on head circumference

Supplemental 2.3.1. Effect of MMS vs IFA on head circumference at birth

Fixed

Study or Subgroup	MMS Mean	MMS SD	MMS Total	IFA Mean	IFA SD	IFA Total	Weight	Mean Difference IV, Fixed, 95% CI
Adu-Afarwuah 2015	33.7	1.4	317	33.7	1.3	304	2.9%	0.00 [-0.21, 0.21]
Ashorn 2010	35.2	1.3	372	35.1	1.4	360	3.5%	0.10 [-0.10, 0.30]
Bhutta 2009	33.6	3.1	574	33.4	3.5	615	0.9%	0.20 [-0.18, 0.58]
Christian 2003	33.7	1.4	588	33.7	1.44	529	4.8%	0.00 [-0.17, 0.17]
Fawzi 2007	34.39	2.62	3338	34.3	2.81	3348	7.8%	0.09 [-0.04, 0.22]
Friis 2004	33.8	1.8661	564	33.6	1.8661	542	2.7%	0.20 [-0.02, 0.42]
Osrin 2005	33.8	2.2	526	33.6	2.2	519	1.9%	0.20 [-0.07, 0.47]
Roberfroid 2008	33.7	1.59	534	33.7	1.41	539	4.1%	0.00 [-0.18, 0.18]
Sunawang 2009	33.2	1.4	630	33.2	1.3	648	6.0%	0.00 [-0.15, 0.15]
Tofail 2008 (1)	32.61	1.95	490	32.3	1.77	1012	3.2%	0.31 [0.11, 0.51]
West 2014	32.71	1.44	7062	32.52	1.47	6963	57.2%	0.19 [0.14, 0.24]
Zeng 2008	33.1	1.6	726	33.2	1.6	752	5.0%	-0.10 [-0.26, 0.06]
**Total (95% CI)**			**15721**			**16131**	**100.0%**	**0.14 [0.10, 0.17]**

Heterogeneity: Chi² = 26.47, df = 11 (P = 0.006); I² = 58%
Test for overall effect: Z = 7.27 (P < 0.00001)

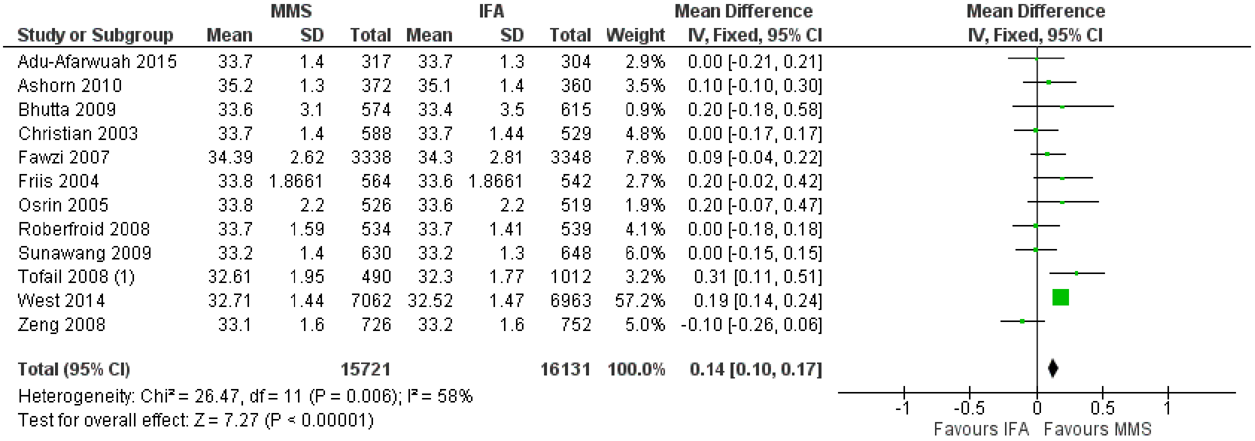

Footnotes
(1) Only the Usual Invitation to Food Supplementation groups were included. Both IFA arms were merged and compared with the MMS arm

Random

Study or Subgroup	MMS Mean	MMS SD	MMS Total	IFA Mean	IFA SD	IFA Total	Weight	Mean Difference IV, Random, 95% CI
Adu-Afarwuah 2015	33.7	1.4	317	33.7	1.3	304	7.0%	0.00 [-0.21, 0.21]
Ashorn 2010	35.2	1.3	372	35.1	1.4	360	7.6%	0.10 [-0.10, 0.30]
Bhutta 2009	33.6	3.1	574	33.4	3.5	615	3.1%	0.20 [-0.18, 0.58]
Christian 2003	33.7	1.4	588	33.7	1.44	529	9.0%	0.00 [-0.17, 0.17]
Fawzi 2007	34.39	2.62	3338	34.3	2.81	3348	11.0%	0.09 [-0.04, 0.22]
Friis 2004	33.8	1.8661	564	33.6	1.8661	542	6.7%	0.20 [-0.02, 0.42]
Osrin 2005	33.8	2.2	526	33.6	2.2	519	5.2%	0.20 [-0.07, 0.47]
Roberfroid 2008	33.7	1.59	534	33.7	1.41	539	8.3%	0.00 [-0.18, 0.18]
Sunawang 2009	33.2	1.4	630	33.2	1.3	648	10.0%	0.00 [-0.15, 0.15]
Tofail 2008 (1)	32.61	1.95	490	32.3	1.77	1012	7.3%	0.31 [0.11, 0.51]
West 2014	32.71	1.44	7062	32.52	1.47	6963	15.8%	0.19 [0.14, 0.24]
Zeng 2008	33.1	1.6	726	33.2	1.6	752	9.2%	-0.10 [-0.26, 0.06]
**Total (95% CI)**			**15721**			**16131**	**100.0%**	**0.09 [0.02, 0.16]**

Heterogeneity: Tau² = 0.01; Chi² = 26.47, df = 11 (P = 0.006); I² = 58%
Test for overall effect: Z = 2.44 (P = 0.01)

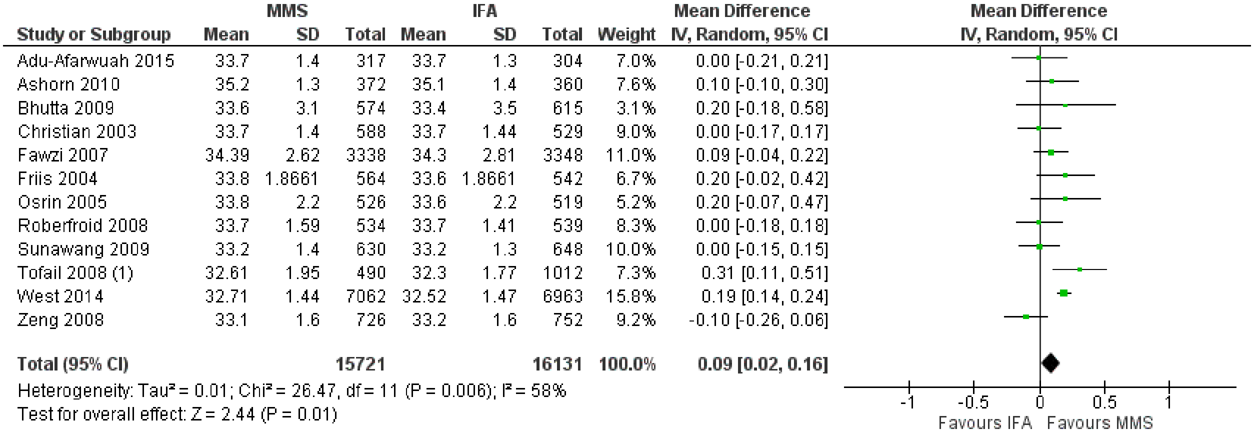

Footnotes
(1) Only the Usual Invitation to Food Supplementation groups were included. Both IFA arms were merged and compared with the MMS arm

Supplemental Figure 2.3.2. Effect of MMS vs IFA on head circumference at 3 months

Fixed

Study or Subgroup	MMS Mean	MMS SD	MMS Total	IFA Mean	IFA SD	IFA Total	Weight	Mean Difference IV, Fixed, 95% CI
Adu-Afarwuah 2015	39.4	1.2	358	39.2	1.2	343	3.7%	0.20 [0.02, 0.38]
Fawzi 2007	40.46	1.49	2994	40.43	1.46	3006	21.1%	0.03 [-0.04, 0.10]
Moore 2009	39.65	1.24	181	39.58	1.25	171	1.7%	0.07 [-0.19, 0.33]
Roberfroid 2008	39.8	1.36	306	39.7	1.36	349	2.7%	0.10 [-0.11, 0.31]
Tofail 2008 (1)	38.3	1.48	376	38.4	1.45	787	3.6%	-0.10 [-0.28, 0.08]
West 2014	38.89	1.34	8055	38.73	1.35	7839	67.2%	0.16 [0.12, 0.20]
**Total (95% CI)**			**12270**			**12495**	**100.0%**	**0.12 [0.09, 0.16]**

Heterogeneity: Chi² = 15.74, df = 5 (P = 0.008); I² = 68%
Test for overall effect: Z = 6.95 (P < 0.00001)

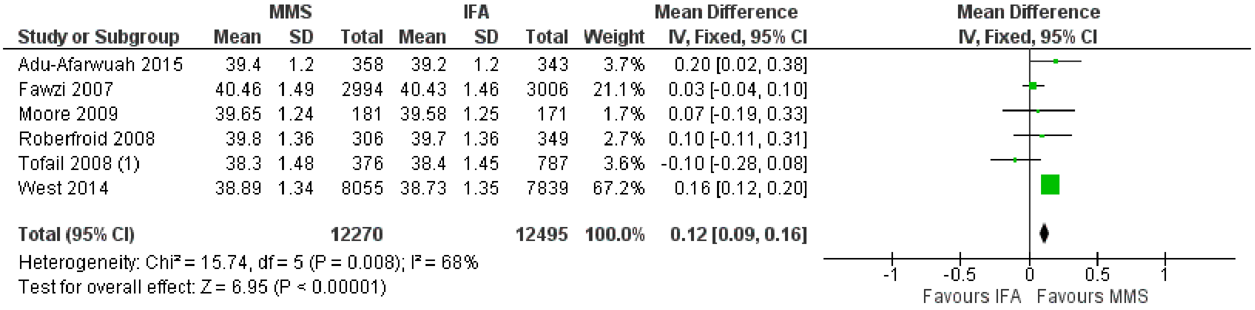

Footnotes
(1) Only the Usual Invitation to Food Supplementation groups were included. Both IFA arms were merged and compared with the MMS arm

Random

Study or Subgroup	MMS Mean	MMS SD	MMS Total	IFA Mean	IFA SD	IFA Total	Weight	Mean Difference IV, Random, 95% CI
Adu-Afarwuah 2015	39.4	1.2	358	39.2	1.2	343	13.6%	0.20 [0.02, 0.38]
Fawzi 2007	40.46	1.49	2994	40.43	1.46	3006	25.0%	0.03 [-0.04, 0.10]
Moore 2009	39.65	1.24	181	39.58	1.25	171	8.3%	0.07 [-0.19, 0.33]
Roberfroid 2008	39.8	1.36	306	39.7	1.36	349	11.2%	0.10 [-0.11, 0.31]
Tofail 2008 (1)	38.3	1.48	376	38.4	1.45	787	13.3%	-0.10 [-0.28, 0.08]
West 2014	38.89	1.34	8055	38.73	1.35	7839	28.6%	0.16 [0.12, 0.20]
**Total (95% CI)**			**12270**			**12495**	**100.0%**	**0.08 [-0.00, 0.17]**

Heterogeneity: Tau² = 0.01; Chi² = 15.74, df = 5 (P = 0.008); I² = 68%
Test for overall effect: Z = 1.88 (P = 0.06)

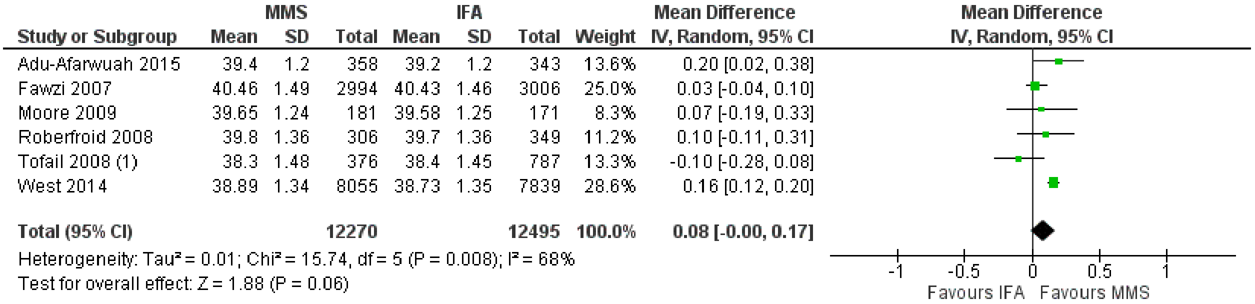

Footnotes
(1) Only the Usual Invitation to Food Supplementation groups were included. Both IFA arms were merged and compared with the MMS arm

Supplemental Figure 2.3.3. Effect of MMS vs IFA on head circumference at 6 months

Fixed

	MMS			IFA				Mean Difference
**Study or Subgroup**	**Mean**	**SD**	**Total**	**Mean**	**SD**	**Total**	**Weight**	**IV, Fixed, 95% CI**
Adu-Afarwuah 2015	41.9	1.2	356	41.7	1.3	347	4.8%	0.20 [0.01, 0.39]
Ashorn 2010 complete FU	42.6	1.4	216	42.6	1.5	218	2.2%	0.00 [-0.27, 0.27]
Ashorn 2010 simplified FU	42.1	1.5	131	42.2	1.4	130	1.3%	-0.10 [-0.45, 0.25]
Fawzi 2007	42.81	1.51	3037	42.81	1.46	3010	29.1%	0.00 [-0.07, 0.07]
Moore 2009	41.83	1.36	170	41.68	1.27	172	2.1%	0.15 [-0.13, 0.43]
Roberfroid 2008	42.3	1.43	270	42.2	1.36	313	3.1%	0.10 [-0.13, 0.33]
Tofail 2008 (1)	40.8	1.46	449	40.8	1.47	892	5.9%	0.00 [-0.17, 0.17]
West 2014	41.53	1.37	4559	41.44	1.36	4489	51.5%	0.09 [0.03, 0.15]
**Total (95% CI)**			**9188**			**9571**	**100.0%**	**0.06 [0.02, 0.10]**

Heterogeneity: Chi² = 7.76, df = 7 (P = 0.35); I² = 10%
Test for overall effect: Z = 2.95 (P = 0.003)

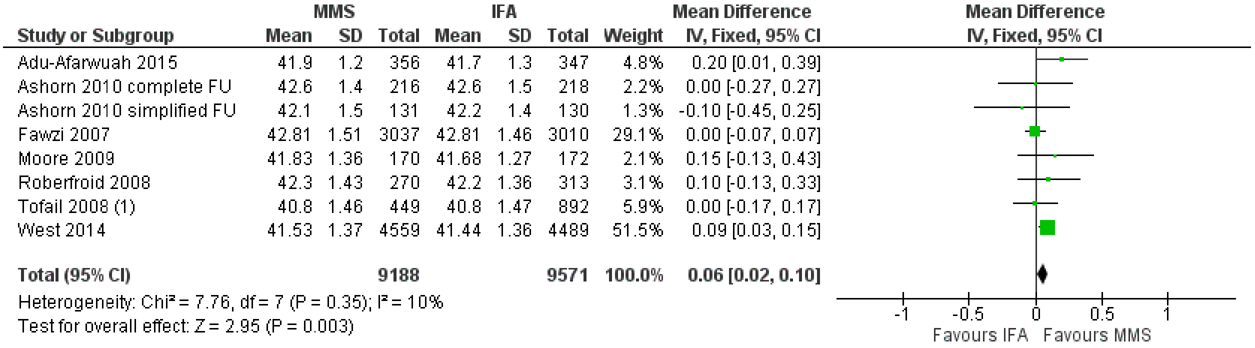

Footnotes
(1) Only the Usual Invitation to Food Supplementation groups were included. Both IFA arms were merged and compared with the MMS arm

Random

	MMS			IFA				Mean Difference
**Study or Subgroup**	**Mean**	**SD**	**Total**	**Mean**	**SD**	**Total**	**Weight**	**IV, Random, 95% CI**
Adu-Afarwuah 2015	41.9	1.2	356	41.7	1.3	347	6.2%	0.20 [0.01, 0.39]
Ashorn 2010 complete FU	42.6	1.4	216	42.6	1.5	218	3.0%	0.00 [-0.27, 0.27]
Ashorn 2010 simplified FU	42.1	1.5	131	42.2	1.4	130	1.8%	-0.10 [-0.45, 0.25]
Fawzi 2007	42.81	1.51	3037	42.81	1.46	3010	30.0%	0.00 [-0.07, 0.07]
Moore 2009	41.83	1.36	170	41.68	1.27	172	2.8%	0.15 [-0.13, 0.43]
Roberfroid 2008	42.3	1.43	270	42.2	1.36	313	4.2%	0.10 [-0.13, 0.33]
Tofail 2008 (1)	40.8	1.46	449	40.8	1.47	892	7.7%	0.00 [-0.17, 0.17]
West 2014	41.53	1.37	4559	41.44	1.36	4489	44.4%	0.09 [0.03, 0.15]
**Total (95% CI)**			**9188**			**9571**	**100.0%**	**0.06 [0.01, 0.11]**

Heterogeneity: Tau² = 0.00; Chi² = 7.76, df = 7 (P = 0.35); I² = 10%
Test for overall effect: Z = 2.44 (P = 0.01)

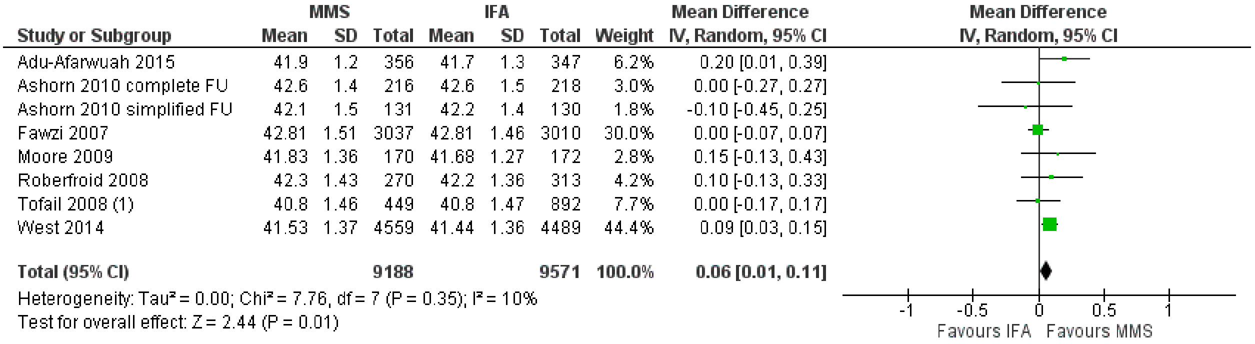

Footnotes
(1) Only the Usual Invitation to Food Supplementation groups were included. Both IFA arms were merged and compared with the MMS arm

Supplemental Figure 2.3.4. Effect of MMS vs IFA on head circumference at 12 months

Fixed

Study or Subgroup	MMS Mean	MMS SD	MMS Total	IFA Mean	IFA SD	IFA Total	Weight	Mean Difference IV, Fixed, 95% CI
Adu-Afarwuah 2015	44.2	1.3	346	44.1	1.4	332	4.7%	0.10 [-0.10, 0.30]
Ashorn 2010 complete FU	44.6	1.5	215	44.6	1.4	219	2.6%	0.00 [-0.27, 0.27]
Fawzi 2007	45.54	1.56	1624	45.52	1.47	1603	17.7%	0.02 [-0.08, 0.12]
Roberfroid 2008	44.4	1.59	240	44.3	1.39	245	2.7%	0.10 [-0.17, 0.37]
Tofail 2008 (1)	43.1	1.47	506	43	1.15	1033	9.1%	0.10 [-0.05, 0.25]
West 2014	43.69	1.41	4945	43.65	1.39	4862	63.1%	0.04 [-0.02, 0.10]
**Total (95% CI)**			**7876**			**8294**	**100.0%**	**0.05 [0.00, 0.09]**

Heterogeneity: Chi² = 1.34, df = 5 (P = 0.93); I² = 0%

Test for overall effect: Z = 2.02 (P = 0.04)

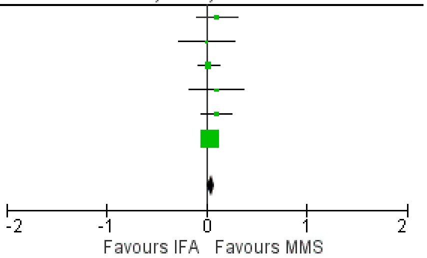

Footnotes

(1) Only the Usual Invitation to Food Supplementation groups were included. Both IFA arms were merged and compared with the MMS arm

Random

Study or Subgroup	MMS Mean	MMS SD	MMS Total	IFA Mean	IFA SD	IFA Total	Weight	Mean Difference IV, Random, 95% CI
Adu-Afarwuah 2015	44.2	1.3	346	44.1	1.4	332	4.7%	0.10 [-0.10, 0.30]
Ashorn 2010 complete FU	44.6	1.5	215	44.6	1.4	219	2.6%	0.00 [-0.27, 0.27]
Fawzi 2007	45.54	1.56	1624	45.52	1.47	1603	17.7%	0.02 [-0.08, 0.12]
Roberfroid 2008	44.4	1.59	240	44.3	1.39	245	2.7%	0.10 [-0.17, 0.37]
Tofail 2008 (1)	43.1	1.47	506	43	1.15	1033	9.1%	0.10 [-0.05, 0.25]
West 2014	43.69	1.41	4945	43.65	1.39	4862	63.1%	0.04 [-0.02, 0.10]
**Total (95% CI)**			**7876**			**8294**	**100.0%**	**0.05 [0.00, 0.09]**

Heterogeneity: Tau² = 0.00; Chi² = 1.34, df = 5 (P = 0.93); I² = 0%

Test for overall effect: Z = 2.02 (P = 0.04)

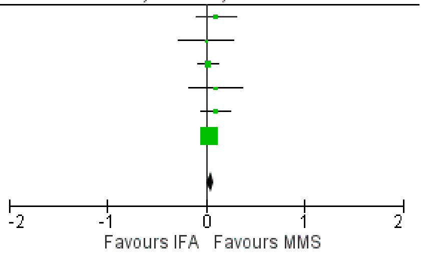

Footnotes

(1) Only the Usual Invitation to Food Supplementation groups were included. Both IFA arms were merged and compared with the MMS arm

Supplemental Figure 2.3.5. Effect of MMS vs IFA on head circumference at 18 months

Fixed

	MMS			IFA				Mean Difference
**Study or Subgroup**	**Mean**	**SD**	**Total**	**Mean**	**SD**	**Total**	**Weight**	**IV, Fixed, 95% CI**
Adu-Afarwuah 2015	45.3	1.27	355	45.2	1.35	337	51.3%	0.10 [-0.10, 0.30]
Ashorn 2010 complete FU	45.3	1.4	222	45.8	1.5	220	26.8%	-0.50 [-0.77, -0.23]
Ashorn 2010 simplified FU	45.7	1.3	130	45.7	1.5	130	16.9%	0.00 [-0.34, 0.34]
Fawzi 2007	46.7	1.45	39	46.77	1.62	55	5.0%	-0.07 [-0.69, 0.55]
**Total (95% CI)**			**746**			**742**	**100.0%**	**-0.09 [-0.23, 0.05]**

Heterogeneity: Chi² = 12.72, df = 3 (P = 0.005); I² = 76%
Test for overall effect: Z = 1.21 (P = 0.23)

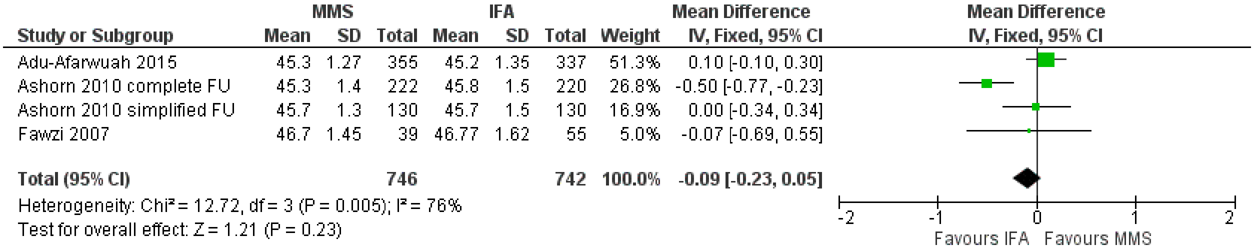

Random

	MMS			IFA				Mean Difference
**Study or Subgroup**	**Mean**	**SD**	**Total**	**Mean**	**SD**	**Total**	**Weight**	**IV, Random, 95% CI**
Adu-Afarwuah 2015	45.3	1.27	355	45.2	1.35	337	31.2%	0.10 [-0.10, 0.30]
Ashorn 2010 complete FU	45.3	1.4	222	45.8	1.5	220	28.2%	-0.50 [-0.77, -0.23]
Ashorn 2010 simplified FU	45.7	1.3	130	45.7	1.5	130	25.3%	0.00 [-0.34, 0.34]
Fawzi 2007	46.7	1.45	39	46.77	1.62	55	15.3%	-0.07 [-0.69, 0.55]
**Total (95% CI)**			**746**			**742**	**100.0%**	**-0.12 [-0.45, 0.20]**

Heterogeneity: Tau² = 0.08; Chi² = 12.72, df = 3 (P = 0.005); I² = 76%
Test for overall effect: Z = 0.73 (P = 0.47)

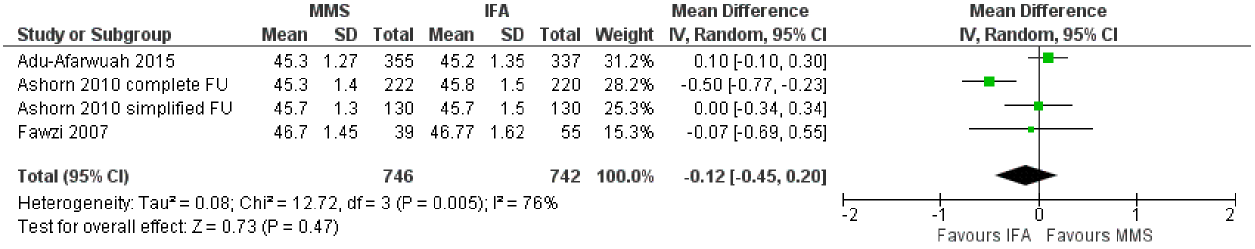

Supplemental Figure 2.3.6. Effect of MMS vs IFA on head circumference at 24 months

Fixed

	MMS			IFA				Mean Difference
**Study or Subgroup**	**Mean**	**SD**	**Total**	**Mean**	**SD**	**Total**	**Weight**	**IV, Fixed, 95% CI**
Ashorn 2010 complete FU	46.3	1.6	179	46.5	1.5	197	4.5%	-0.20 [-0.51, 0.11]
West 2014	45.55	1.43	3405	45.53	1.42	3355	95.5%	0.02 [-0.05, 0.09]
**Total (95% CI)**			**3584**			**3552**	**100.0%**	**0.01 [-0.06, 0.08]**

Heterogeneity: Chi² = 1.80, df = 1 (P = 0.18); I² = 44%
Test for overall effect: Z = 0.30 (P = 0.76)

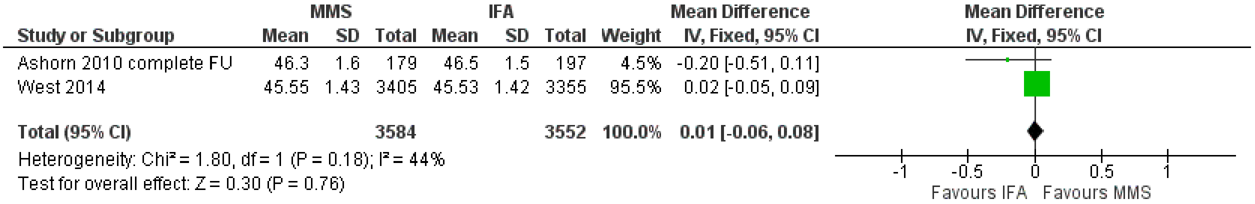

Random

	MMS			IFA				Mean Difference
**Study or Subgroup**	**Mean**	**SD**	**Total**	**Mean**	**SD**	**Total**	**Weight**	**IV, Random, 95% CI**
Ashorn 2010 complete FU	46.3	1.6	179	46.5	1.5	197	24.7%	-0.20 [-0.51, 0.11]
West 2014	45.55	1.43	3405	45.53	1.42	3355	75.3%	0.02 [-0.05, 0.09]
**Total (95% CI)**			**3584**			**3552**	**100.0%**	**-0.03 [-0.22, 0.15]**

Heterogeneity: Tau² = 0.01; Chi² = 1.80, df = 1 (P = 0.18); I² = 44%
Test for overall effect: Z = 0.36 (P = 0.72)

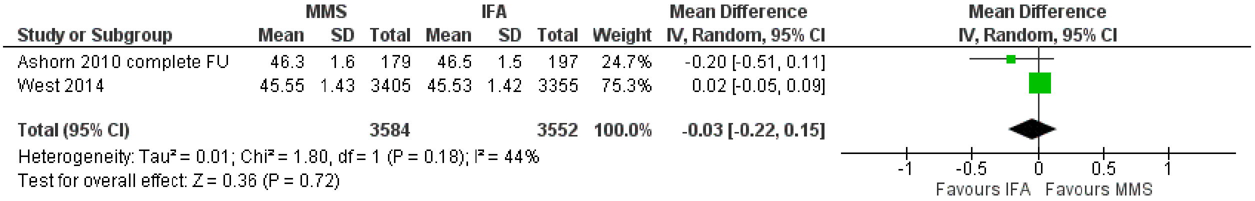

Supplemental Figure 2.4. Effect of MMS vs IFA on MUAC

Supplemental Figure 2.4.1. Effect of MMS vs IFA on MUAC at birth

Fixed

Study or Subgroup	MMS Mean	MMS SD	MMS Total	IFA Mean	IFA SD	IFA Total	Weight	Mean Difference IV, Fixed, 95% CI
Adu-Afarwuah 2015	10.5	0.86	310	10.4	0.92	297	3.3%	0.10 [-0.04, 0.24]
Ashorn 2010	10.6	0.9	376	10.5	1	366	3.5%	0.10 [-0.04, 0.24]
Bhutta 2009	9.6	1.3	574	9.5	1.3	615	3.0%	0.10 [-0.05, 0.25]
Roberfroid 2008	10.3	0.9	495	10.2	0.9	500	5.3%	0.10 [-0.01, 0.21]
West 2014	9.55	0.84	7157	9.44	0.86	7091	84.9%	0.11 [0.08, 0.14]
**Total (95% CI)**			**8912**			**8869**	**100.0%**	**0.11 [0.08, 0.13]**

Heterogeneity: Chi² = 0.07, df = 4 (P = 1.00); I² = 0%
Test for overall effect: Z = 8.27 (P < 0.00001)

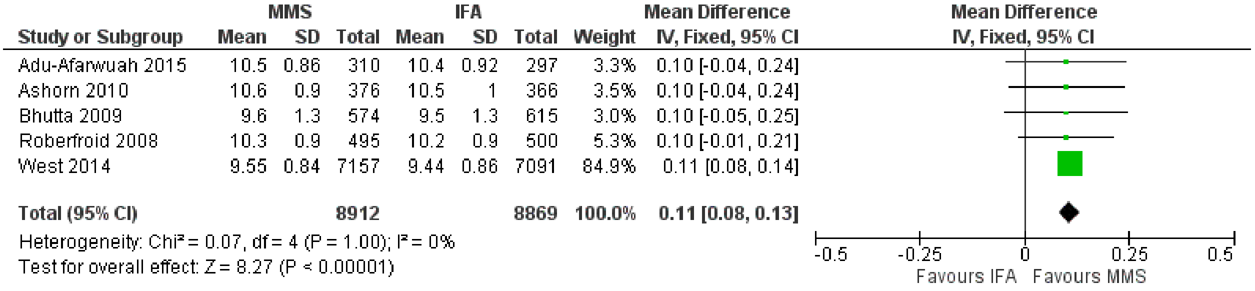

Random

Study or Subgroup	MMS Mean	MMS SD	MMS Total	IFA Mean	IFA SD	IFA Total	Weight	Mean Difference IV, Random, 95% CI
Adu-Afarwuah 2015	10.5	0.86	310	10.4	0.92	297	3.3%	0.10 [-0.04, 0.24]
Ashorn 2010	10.6	0.9	376	10.5	1	366	3.5%	0.10 [-0.04, 0.24]
Bhutta 2009	9.6	1.3	574	9.5	1.3	615	3.0%	0.10 [-0.05, 0.25]
Roberfroid 2008	10.3	0.9	495	10.2	0.9	500	5.3%	0.10 [-0.01, 0.21]
West 2014	9.55	0.84	7157	9.44	0.86	7091	84.9%	0.11 [0.08, 0.14]
**Total (95% CI)**			**8912**			**8869**	**100.0%**	**0.11 [0.08, 0.13]**

Heterogeneity: Tau² = 0.00; Chi² = 0.07, df = 4 (P = 1.00); I² = 0%
Test for overall effect: Z = 8.27 (P < 0.00001)

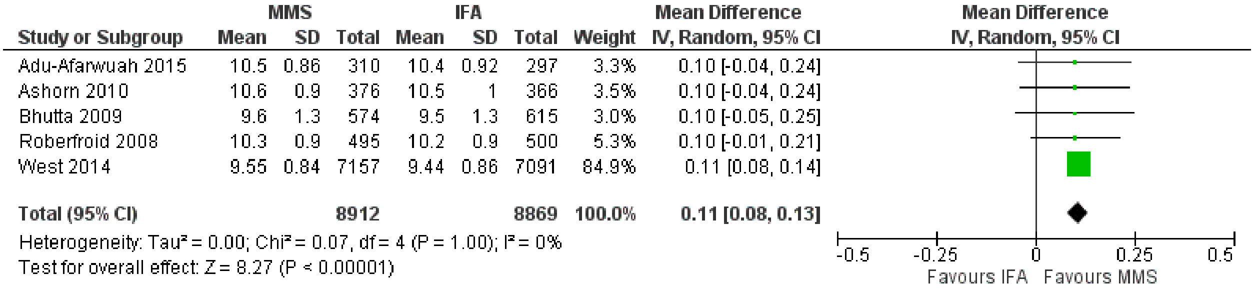

Supplemental Figure 2.4.2 Effect of MMS vs IFA on MUAC at 3 months

Fixed

Study or Subgroup	MMS Mean	MMS SD	MMS Total	IFA Mean	IFA SD	IFA Total	Weight	Mean Difference IV, Fixed, 95% CI
Adu-Afarwuah 2015	13.4	1.07	358	13.3	1.13	344	2.8%	0.10 [-0.06, 0.26]
Fawzi 2007	13.5	1.13	1488	13.51	1.13	1512	11.5%	-0.01 [-0.09, 0.07]
Moore 2009	13.4	0.96	180	13.39	0.93	171	1.9%	0.01 [-0.19, 0.21]
Roberfroid 2008	13.4	1	306	13.4	1.1	349	2.9%	0.00 [-0.16, 0.16]
Sunawang 2009	13.2	0.89	151	13.15	1.96	148	0.6%	0.05 [-0.30, 0.40]
West 2014	12.53	0.97	8072	12.43	1	7858	80.2%	0.10 [0.07, 0.13]
**Total (95% CI)**			**10555**			**10382**	**100.0%**	**0.08 [0.06, 0.11]**

Heterogeneity: Chi² = 7.89, df = 5 (P = 0.16); I² = 37%
Test for overall effect: Z = 5.89 (P < 0.00001)

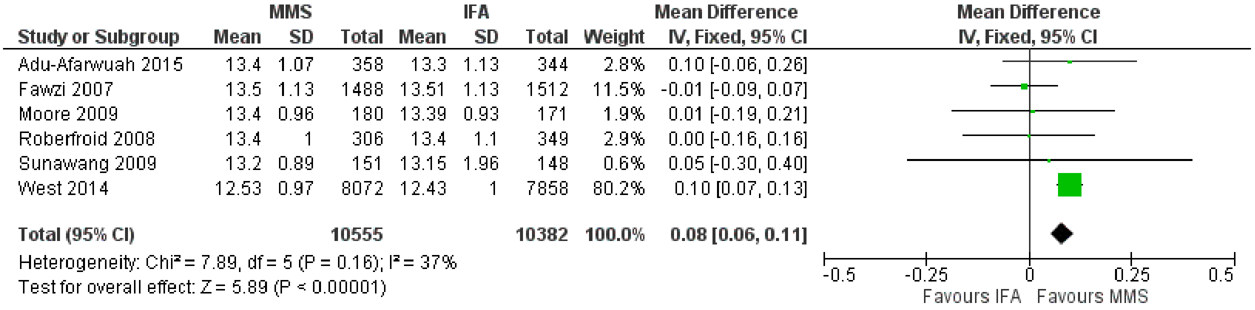

Random

Study or Subgroup	MMS Mean	MMS SD	MMS Total	IFA Mean	IFA SD	IFA Total	Weight	Mean Difference IV, Random, 95% CI
Adu-Afarwuah 2015	13.4	1.07	358	13.3	1.13	344	9.9%	0.10 [-0.06, 0.26]
Fawzi 2007	13.5	1.13	1488	13.51	1.13	1512	25.3%	-0.01 [-0.09, 0.07]
Moore 2009	13.4	0.96	180	13.39	0.93	171	7.2%	0.01 [-0.19, 0.21]
Roberfroid 2008	13.4	1	306	13.4	1.1	349	10.1%	0.00 [-0.16, 0.16]
Sunawang 2009	13.2	0.89	151	13.15	1.96	148	2.6%	0.05 [-0.30, 0.40]
West 2014	12.53	0.97	8072	12.43	1	7858	44.8%	0.10 [0.07, 0.13]
**Total (95% CI)**			**10555**			**10382**	**100.0%**	**0.05 [-0.00, 0.11]**

Heterogeneity: Tau² = 0.00; Chi² = 7.89, df = 5 (P = 0.16); I² = 37%
Test for overall effect: Z = 1.86 (P = 0.06)

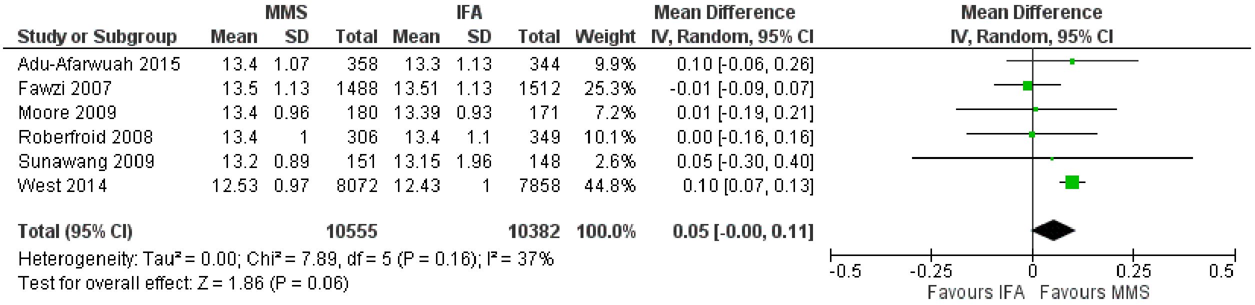

Supplemental Figure 2.4.3 Effect of MMS vs IFA on MUAC at 6 months

Fixed

Study or Subgroup	MMS Mean	MMS SD	MMS Total	IFA Mean	IFA SD	IFA Total	Weight	Mean Difference IV, Fixed, 95% CI
Adu-Afarwuah 2015	13.9	1.13	356	13.8	1.13	348	4.2%	0.10 [-0.07, 0.27]
Ashorn 2010 complete FU	14.2	1.4	216	14.1	1.4	220	1.7%	0.10 [-0.16, 0.36]
Ashorn 2010 simplified FU	14.3	1.3	131	14.4	1.2	130	1.3%	-0.10 [-0.40, 0.20]
Fawzi 2007	14.07	1.26	1853	14.08	1.23	1847	18.1%	-0.01 [-0.09, 0.07]
Moore 2009	13.91	0.98	170	13.85	1.1	172	2.4%	0.06 [-0.16, 0.28]
Roberfroid 2008	13.9	1.7	270	13.8	1.2	313	2.0%	0.10 [-0.14, 0.34]
Sunawang 2009	13.9	1	151	13.9	1.03	148	2.2%	0.00 [-0.23, 0.23]
West 2014	13.29	1	4564	13.27	1.01	4499	68.1%	0.02 [-0.02, 0.06]
**Total (95% CI)**			**7711**			**7677**	**100.0%**	**0.02 [-0.01, 0.05]**

Heterogeneity: Chi² = 2.95, df = 7 (P = 0.89); I² = 0%
Test for overall effect: Z = 1.14 (P = 0.25)

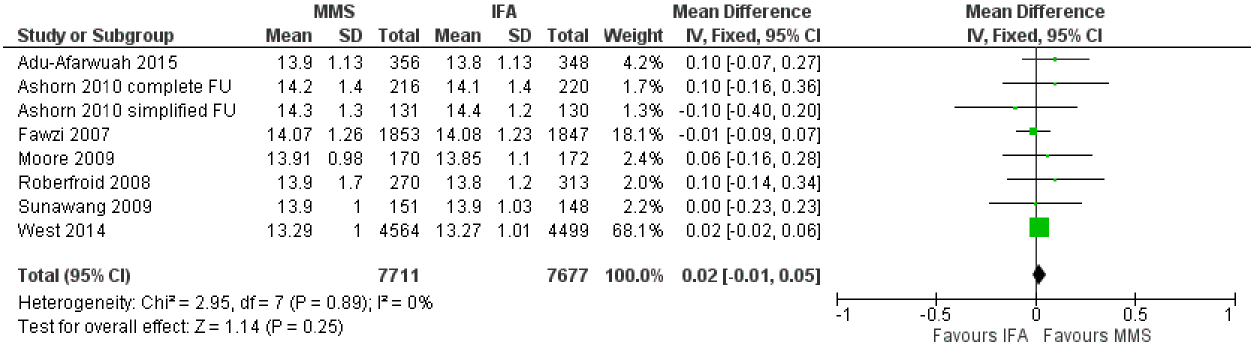

Random

Study or Subgroup	MMS Mean	MMS SD	MMS Total	IFA Mean	IFA SD	IFA Total	Weight	Mean Difference IV, Random, 95% CI
Adu-Afarwuah 2015	13.9	1.13	356	13.8	1.13	348	4.2%	0.10 [-0.07, 0.27]
Ashorn 2010 complete FU	14.2	1.4	216	14.1	1.4	220	1.7%	0.10 [-0.16, 0.36]
Ashorn 2010 simplified FU	14.3	1.3	131	14.4	1.2	130	1.3%	-0.10 [-0.40, 0.20]
Fawzi 2007	14.07	1.26	1853	14.08	1.23	1847	18.1%	-0.01 [-0.09, 0.07]
Moore 2009	13.91	0.98	170	13.85	1.1	172	2.4%	0.06 [-0.16, 0.28]
Roberfroid 2008	13.9	1.7	270	13.8	1.2	313	2.0%	0.10 [-0.14, 0.34]
Sunawang 2009	13.9	1	151	13.9	1.03	148	2.2%	0.00 [-0.23, 0.23]
West 2014	13.29	1	4564	13.27	1.01	4499	68.1%	0.02 [-0.02, 0.06]
**Total (95% CI)**			**7711**			**7677**	**100.0%**	**0.02 [-0.01, 0.05]**

Heterogeneity: Tau² = 0.00; Chi² = 2.95, df = 7 (P = 0.89); I² = 0%
Test for overall effect: Z = 1.14 (P = 0.25)

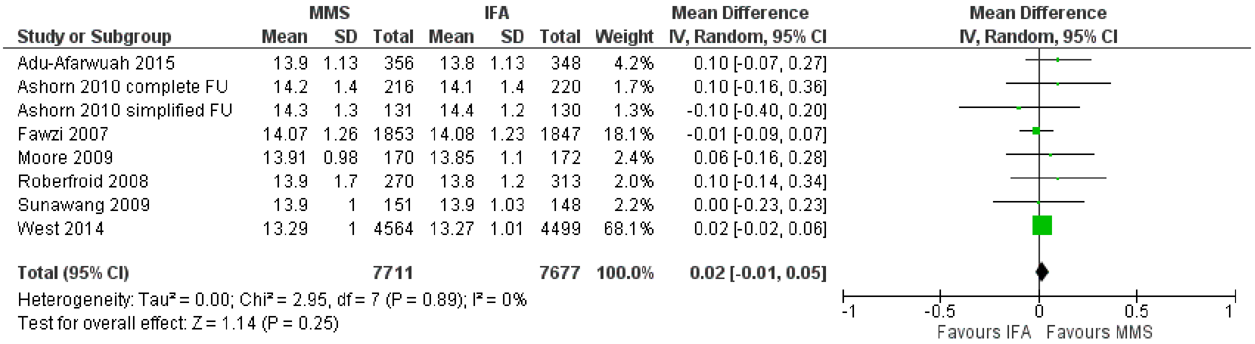

Supplemental Figure 2.4.4 Effect of MMS vs IFA on MUAC at 12 months

Fixed

Study or Subgroup	MMS Mean	MMS SD	MMS Total	IFA Mean	IFA SD	IFA Total	Weight	Mean Difference IV, Fixed, 95% CI
Adu-Afarwuah 2015	14.2	1.08	346	14.1	1.09	334	4.3%	0.10 [-0.06, 0.26]
Ashorn 2010 complete FU	14.5	1.3	215	14.6	1.1	219	2.2%	-0.10 [-0.33, 0.13]
Fawzi 2007	14.52	1.31	1492	14.5	1.28	1485	13.3%	0.02 [-0.07, 0.11]
Roberfroid 2008	13.9	1.3	240	13.8	1.1	248	2.5%	0.10 [-0.11, 0.31]
Sunawang 2009	13.9	1.05	150	13.9	0.94	148	2.3%	0.00 [-0.23, 0.23]
West 2014	13.36	0.99	4998	13.34	1	4913	75.3%	0.02 [-0.02, 0.06]
**Total (95% CI)**			**7441**			**7347**	**100.0%**	**0.02 [-0.01, 0.06]**

Heterogeneity: Chi² = 2.55, df = 5 (P = 0.77); I² = 0%
Test for overall effect: Z = 1.29 (P = 0.20)

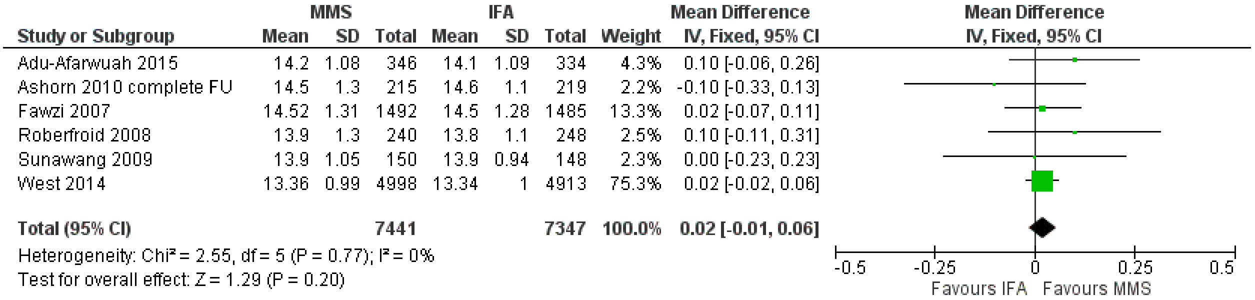

Random

Study or Subgroup	MMS Mean	MMS SD	MMS Total	IFA Mean	IFA SD	IFA Total	Weight	Mean Difference IV, Random, 95% CI
Adu-Afarwuah 2015	14.2	1.08	346	14.1	1.09	334	4.3%	0.10 [-0.06, 0.26]
Ashorn 2010 complete FU	14.5	1.3	215	14.6	1.1	219	2.2%	-0.10 [-0.33, 0.13]
Fawzi 2007	14.52	1.31	1492	14.5	1.28	1485	13.3%	0.02 [-0.07, 0.11]
Roberfroid 2008	13.9	1.3	240	13.8	1.1	248	2.5%	0.10 [-0.11, 0.31]
Sunawang 2009	13.9	1.05	150	13.9	0.94	148	2.3%	0.00 [-0.23, 0.23]
West 2014	13.36	0.99	4998	13.34	1	4913	75.3%	0.02 [-0.02, 0.06]
**Total (95% CI)**			**7441**			**7347**	**100.0%**	**0.02 [-0.01, 0.06]**

Heterogeneity: Tau² = 0.00; Chi² = 2.55, df = 5 (P = 0.77); I² = 0%
Test for overall effect: Z = 1.29 (P = 0.20)

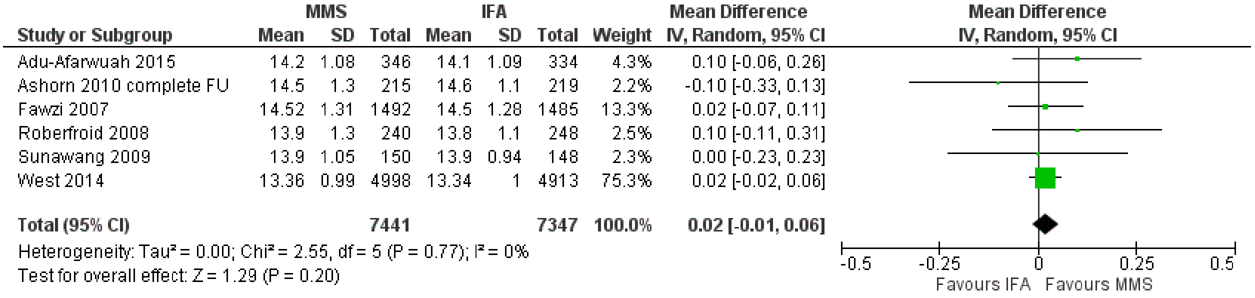

Supplemental Figure 2.4.5 Effect of MMS vs IFA on MUAC at 18 months

Fixed

	MMS			IFA				Mean Difference
Study or Subgroup	Mean	SD	Total	Mean	SD	Total	Weight	IV, Fixed, 95% CI
Adu-Afarwuah 2015	14.2	1.04	356	14.1	1.05	340	52.4%	0.10 [-0.06, 0.26]
Ashorn 2010 complete FU	14.8	1.3	222	14.8	1	220	27.1%	0.00 [-0.22, 0.22]
Ashorn 2010 simplified FU	14.8	1.2	130	14.7	1.1	130	16.1%	0.10 [-0.18, 0.38]
Fawzi 2007	14.57	1.15	39	14.79	1.47	53	4.4%	-0.22 [-0.76, 0.32]
**Total (95% CI)**			**747**			**743**	**100.0%**	**0.06 [-0.05, 0.17]**

Heterogeneity: Chi² = 1.68, df = 3 (P = 0.64); I² = 0%
Test for overall effect: Z = 1.03 (P = 0.31)

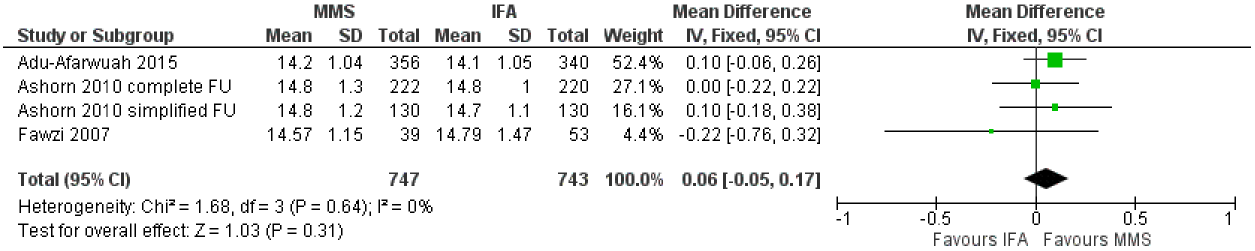

Random

	MMS			IFA				Mean Difference
Study or Subgroup	Mean	SD	Total	Mean	SD	Total	Weight	IV, Random, 95% CI
Adu-Afarwuah 2015	14.2	1.04	356	14.1	1.05	340	52.4%	0.10 [-0.06, 0.26]
Ashorn 2010 complete FU	14.8	1.3	222	14.8	1	220	27.1%	0.00 [-0.22, 0.22]
Ashorn 2010 simplified FU	14.8	1.2	130	14.7	1.1	130	16.1%	0.10 [-0.18, 0.38]
Fawzi 2007	14.57	1.15	39	14.79	1.47	53	4.4%	-0.22 [-0.76, 0.32]
**Total (95% CI)**			**747**			**743**	**100.0%**	**0.06 [-0.05, 0.17]**

Heterogeneity: Tau² = 0.00; Chi² = 1.68, df = 3 (P = 0.64); I² = 0%
Test for overall effect: Z = 1.03 (P = 0.31)

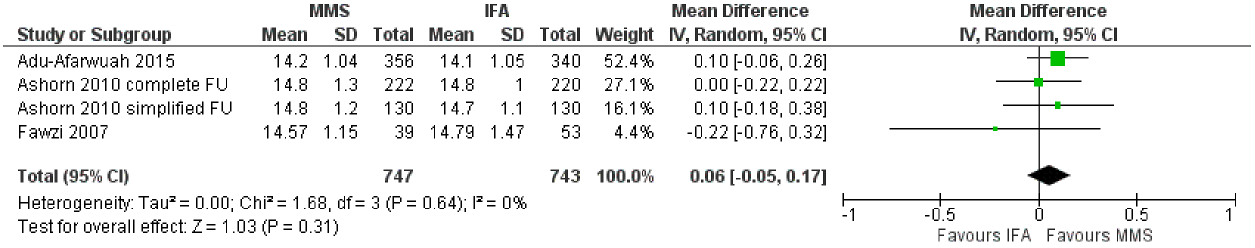

Supplemental Figure 2.4.6 Effect of MMS vs IFA on MUAC at 24 months

Fixed

Study or Subgroup	MMS Mean	MMS SD	MMS Total	IFA Mean	IFA SD	IFA Total	Weight	Mean Difference IV, Fixed, 95% CI
Ashorn 2010 complete FU	14.8	1.2	179	15	1.1	197	3.4%	-0.20 [-0.43, 0.03]
Bliznashka 2021	13.52	3.0058	642	13.68	3.0058	718	1.8%	-0.16 [-0.48, 0.16]
Roberfroid 2008	14.1	1.1	54	14.1	1.3	55	0.9%	0.00 [-0.45, 0.45]
West 2014	13.71	0.92	3433	13.71	0.95	3381	93.9%	0.00 [-0.04, 0.04]
**Total (95% CI)**			**4308**			**4351**	**100.0%**	**-0.01 [-0.05, 0.03]**

Heterogeneity: Chi² = 3.59, df = 3 (P = 0.31); I² = 16%
Test for overall effect: Z = 0.44 (P = 0.66)

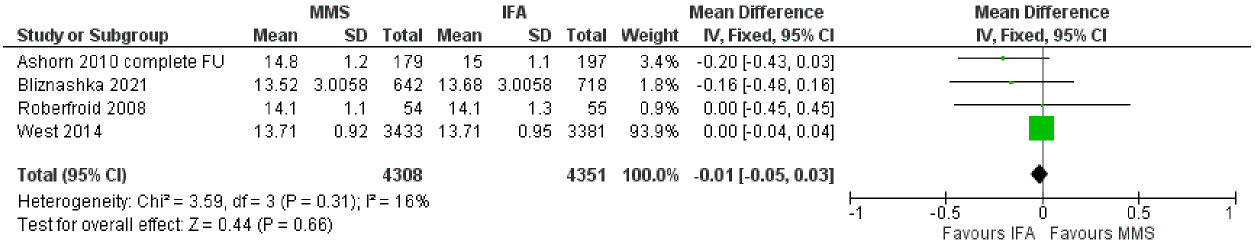

Random

Study or Subgroup	MMS Mean	MMS SD	MMS Total	IFA Mean	IFA SD	IFA Total	Weight	Mean Difference IV, Random, 95% CI
Ashorn 2010 complete FU	14.8	1.2	179	15	1.1	197	13.3%	-0.20 [-0.43, 0.03]
Bliznashka 2021	13.52	3.0058	642	13.68	3.0058	718	7.6%	-0.16 [-0.48, 0.16]
Roberfroid 2008	14.1	1.1	54	14.1	1.3	55	4.0%	0.00 [-0.45, 0.45]
West 2014	13.71	0.92	3433	13.71	0.95	3381	75.2%	0.00 [-0.04, 0.04]
**Total (95% CI)**			**4308**			**4351**	**100.0%**	**-0.04 [-0.13, 0.05]**

Heterogeneity: Tau² = 0.00; Chi² = 3.59, df = 3 (P = 0.31); I² = 16%
Test for overall effect: Z = 0.82 (P = 0.41)

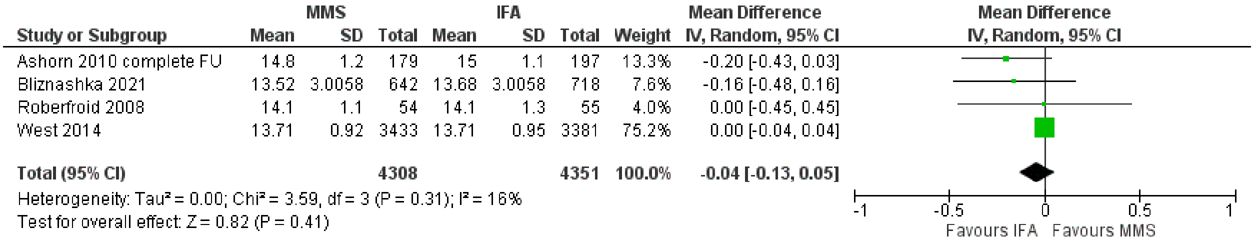

Supplemental Figure 2.5. Effect of MMS vs IFA on LAZ

Supplemental Figure 2.5.1. Effect of MMS vs IFA on LAZ at birth

Fixed

	MMS			IFA				Mean Difference
**Study or Subgroup**	**Mean**	**SD**	**Total**	**Mean**	**SD**	**Total**	**Weight**	**IV, Fixed, 95% CI**
Adu-Afarwuah 2015	-0.59	1.03	313	-0.73	1.02	305	3.6%	0.14 [-0.02, 0.30]
Ashorn 2010	-0.98	1.1	376	-1.1	1.21	366	3.4%	0.12 [-0.05, 0.29]
Fawzi 2007	-1.12	2.97	2799	-1.19	2.98	2794	3.8%	0.07 [-0.09, 0.23]
Osrin 2005	-0.34	1.28	526	-0.41	1.27	517	3.9%	0.07 [-0.08, 0.22]
Roberfroid 2008	-0.66	1.3	534	-0.8	1.18	539	4.2%	0.14 [-0.01, 0.29]
Sunawang 2009	-1.03	1.11	632	-1.02	1.08	649	6.5%	-0.01 [-0.13, 0.11]
Tofail 2008 (1)	-1.12	1.18	483	-1.1	1.17	1006	5.7%	-0.02 [-0.15, 0.11]
West 2014	-1.56	1.09	6964	-1.66	1.12	6861	68.8%	0.10 [0.06, 0.14]
**Total (95% CI)**			**12627**			**13037**	**100.0%**	**0.09 [0.06, 0.12]**

Heterogeneity: $Chi^2 = 6.83, df = 7 (P = 0.45); I^2 = 0\%$
Test for overall effect: Z = 5.61 (P < 0.00001)

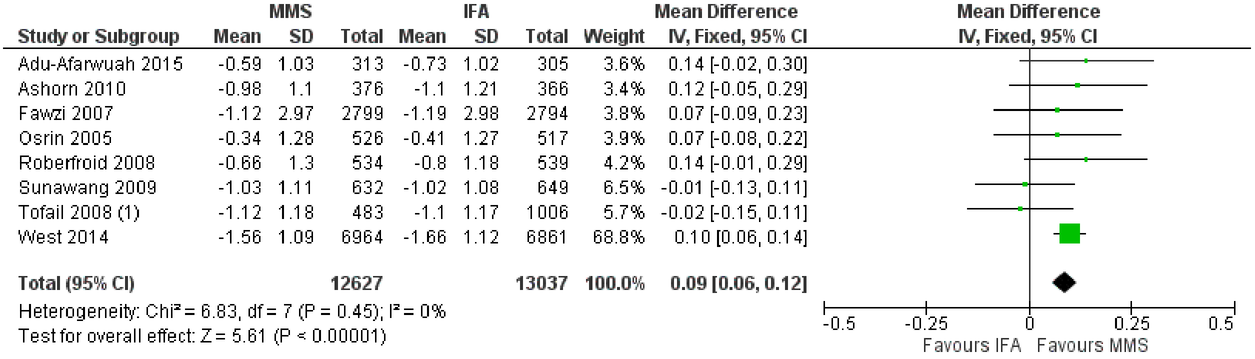

Footnotes
(1) Only the Usual Invitation to Food Supplementation groups were included. Both IFA arms were merged and compared with the MMS arm

Random

	MMS			IFA				Mean Difference
**Study or Subgroup**	**Mean**	**SD**	**Total**	**Mean**	**SD**	**Total**	**Weight**	**IV, Random, 95% CI**
Adu-Afarwuah 2015	-0.59	1.03	313	-0.73	1.02	305	3.6%	0.14 [-0.02, 0.30]
Ashorn 2010	-0.98	1.1	376	-1.1	1.21	366	3.4%	0.12 [-0.05, 0.29]
Fawzi 2007	-1.12	2.97	2799	-1.19	2.98	2794	3.8%	0.07 [-0.09, 0.23]
Osrin 2005	-0.34	1.28	526	-0.41	1.27	517	3.9%	0.07 [-0.08, 0.22]
Roberfroid 2008	-0.66	1.3	534	-0.8	1.18	539	4.2%	0.14 [-0.01, 0.29]
Sunawang 2009	-1.03	1.11	632	-1.02	1.08	649	6.5%	-0.01 [-0.13, 0.11]
Tofail 2008 (1)	-1.12	1.18	483	-1.1	1.17	1006	5.7%	-0.02 [-0.15, 0.11]
West 2014	-1.56	1.09	6964	-1.66	1.12	6861	68.8%	0.10 [0.06, 0.14]
**Total (95% CI)**			**12627**			**13037**	**100.0%**	**0.09 [0.06, 0.12]**

Heterogeneity: $Tau^2 = 0.00; Chi^2 = 6.83, df = 7 (P = 0.45); I^2 = 0\%$
Test for overall effect: Z = 5.61 (P < 0.00001)

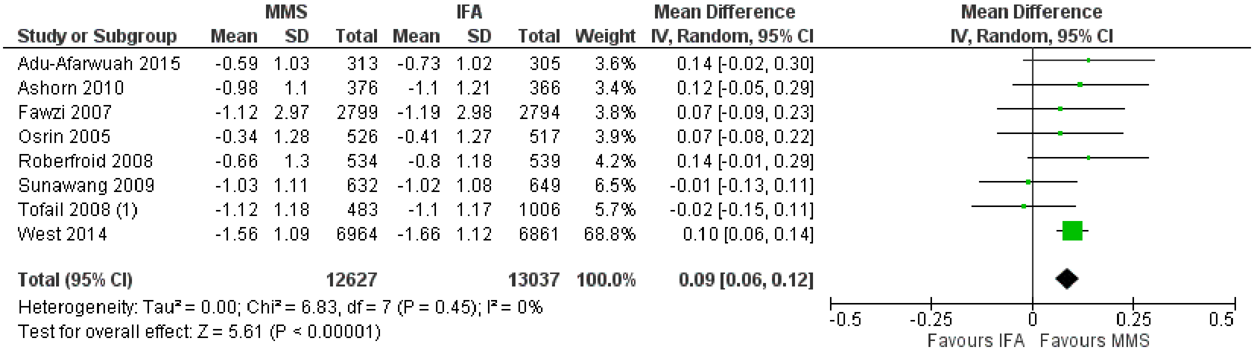

Footnotes
(1) Only the Usual Invitation to Food Supplementation groups were included. Both IFA arms were merged and compared with the MMS arm

Supplemental Figure 2.5.2 Effect of MMS vs IFA on LAZ at 3 months

Fixed

	MMS			IFA				Mean Difference
Study or Subgroup	Mean	SD	Total	Mean	SD	Total	Weight	IV, Fixed, 95% CI
Adu-Afarwuah 2015	-0.63	1.02	357	-0.8	0.98	343	3.9%	0.17 [0.02, 0.32]
Fawzi 2007	-0.23	1.39	2907	-0.28	1.42	2903	16.4%	0.05 [-0.02, 0.12]
Moore 2009	-0.34	1.11	179	-0.35	0.98	171	1.8%	0.01 [-0.21, 0.23]
Roberfroid 2008	-0.81	1.22	306	-0.85	1.22	349	2.4%	0.04 [-0.15, 0.23]
Sunawang 2009	-0.69	1.16	151	-0.69	1.03	148	1.4%	0.00 [-0.25, 0.25]
Tofail 2008 (1)	-1.4	1.09	372	-1.35	1.08	783	4.8%	-0.05 [-0.18, 0.08]
West 2014	-1.29	1.1	7931	-1.4	1.14	7725	69.4%	0.11 [0.07, 0.15]
**Total (95% CI)**			**12203**			**12422**	**100.0%**	**0.09 [0.06, 0.12]**

Heterogeneity: Chi² = 9.02, df = 6 (P = 0.17); I² = 33%
Test for overall effect: Z = 6.03 (P < 0.00001)

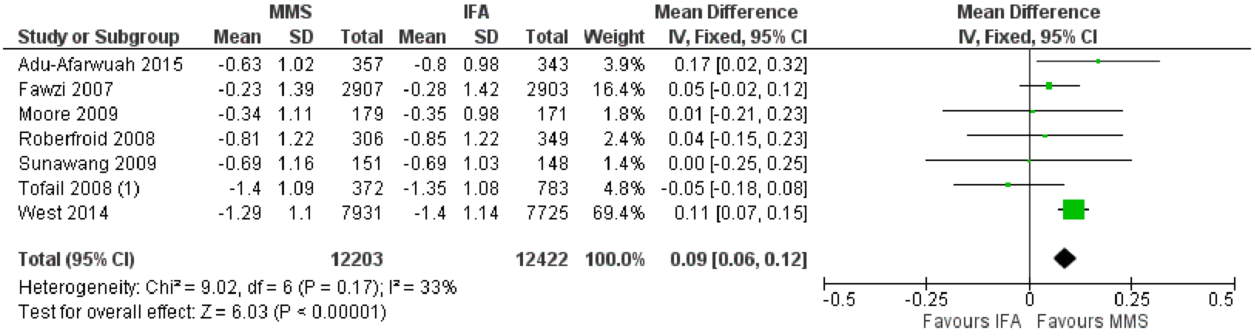

Footnotes
(1) Only the Usual Invitation to Food Supplementation groups were included. Both IFA arms were merged and compared with the MMS arm

Random

	MMS			IFA				Mean Difference
Study or Subgroup	Mean	SD	Total	Mean	SD	Total	Weight	IV, Random, 95% CI
Adu-Afarwuah 2015	-0.63	1.02	357	-0.8	0.98	343	9.5%	0.17 [0.02, 0.32]
Fawzi 2007	-0.23	1.39	2907	-0.28	1.42	2903	24.6%	0.05 [-0.02, 0.12]
Moore 2009	-0.34	1.11	179	-0.35	0.98	171	4.9%	0.01 [-0.21, 0.23]
Roberfroid 2008	-0.81	1.22	306	-0.85	1.22	349	6.4%	0.04 [-0.15, 0.23]
Sunawang 2009	-0.69	1.16	151	-0.69	1.03	148	3.9%	0.00 [-0.25, 0.25]
Tofail 2008 (1)	-1.4	1.09	372	-1.35	1.08	783	11.1%	-0.05 [-0.18, 0.08]
West 2014	-1.29	1.1	7931	-1.4	1.14	7725	39.6%	0.11 [0.07, 0.15]
**Total (95% CI)**			**12203**			**12422**	**100.0%**	**0.07 [0.02, 0.12]**

Heterogeneity: Tau² = 0.00; Chi² = 9.02, df = 6 (P = 0.17); I² = 33%
Test for overall effect: Z = 2.68 (P = 0.007)

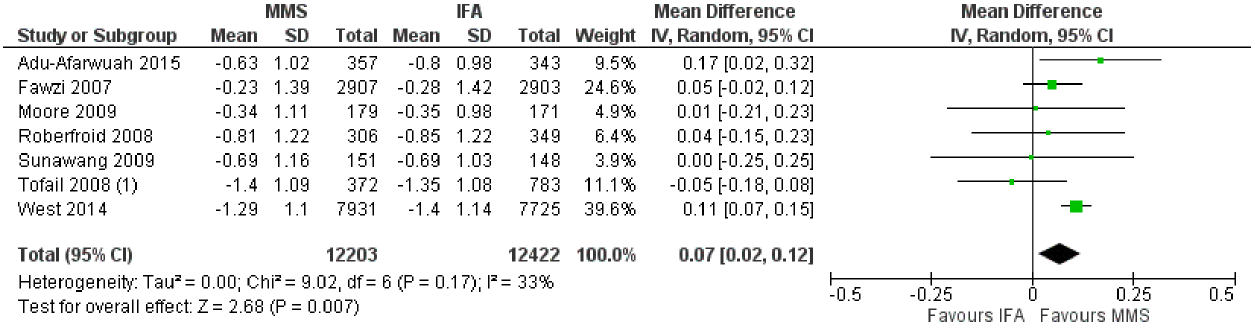

Footnotes
(1) Only the Usual Invitation to Food Supplementation groups were included. Both IFA arms were merged and compared with the MMS arm

Supplemental Figure 2.5.3 Effect of MMS vs IFA on LAZ at 6 months

Fixed

Study or Subgroup	MMS Mean	MMS SD	MMS Total	IFA Mean	IFA SD	IFA Total	Weight	Mean Difference IV, Fixed, 95% CI
Adu-Afarwuah 2015	-0.71	1.01	355	-0.79	0.98	347	4.7%	0.08 [-0.07, 0.23]
Ashorn 2010 complete FU	-1.19	1.15	213	-1.3	1.21	220	2.1%	0.11 [-0.11, 0.33]
Ashorn 2010 simplified FU	-1.24	1.18	131	-1.08	1.17	130	1.3%	-0.16 [-0.45, 0.13]
Fawzi 2007	-0.52	1.21	3000	-0.54	1.26	2978	26.1%	0.02 [-0.04, 0.08]
Moore 2009	-0.43	1.12	170	-0.47	0.94	171	2.1%	0.04 [-0.18, 0.26]
Roberfroid 2008	-0.86	1.1	270	-0.94	1.15	313	3.1%	0.08 [-0.10, 0.26]
Sunawang 2009	-0.89	1.02	151	-0.82	0.98	148	2.0%	-0.07 [-0.30, 0.16]
Tofail 2008 (1)	-1.39	1.07	445	-1.36	1.08	885	6.8%	-0.03 [-0.15, 0.09]
West 2014	-1.3	1.06	4494	-1.36	1.08	4429	51.9%	0.06 [0.02, 0.10]
**Total (95% CI)**			**9229**			**9621**	**100.0%**	**0.04 [0.01, 0.07]**

Heterogeneity: Chi² = 6.07, df = 8 (P = 0.64); I² = 0%
Test for overall effect: Z = 2.46 (P = 0.01)

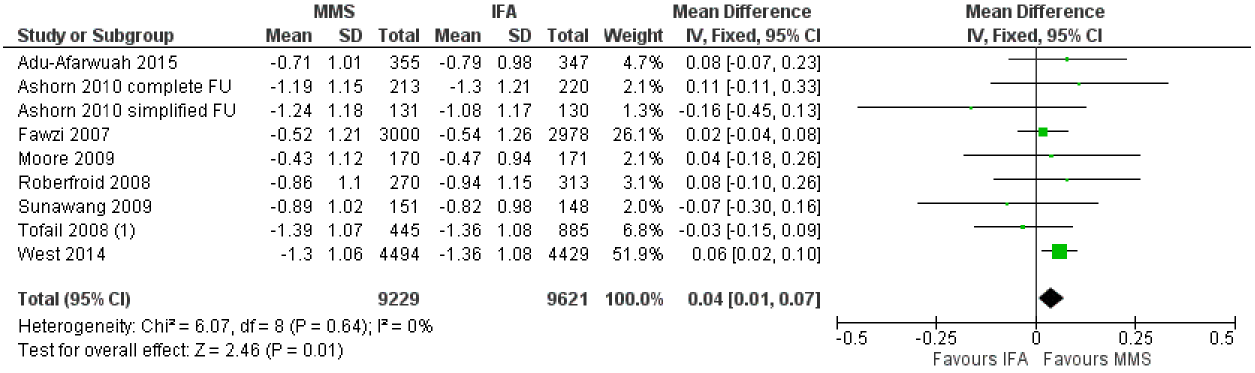

Footnotes
(1) Only the Usual Invitation to Food Supplementation groups were included. Both IFA arms were merged and compared with the MMS arm

Random

Study or Subgroup	MMS Mean	MMS SD	MMS Total	IFA Mean	IFA SD	IFA Total	Weight	Mean Difference IV, Random, 95% CI
Adu-Afarwuah 2015	-0.71	1.01	355	-0.79	0.98	347	4.7%	0.08 [-0.07, 0.23]
Ashorn 2010 complete FU	-1.19	1.15	213	-1.3	1.21	220	2.1%	0.11 [-0.11, 0.33]
Ashorn 2010 simplified FU	-1.24	1.18	131	-1.08	1.17	130	1.3%	-0.16 [-0.45, 0.13]
Fawzi 2007	-0.52	1.21	3000	-0.54	1.26	2978	26.1%	0.02 [-0.04, 0.08]
Moore 2009	-0.43	1.12	170	-0.47	0.94	171	2.1%	0.04 [-0.18, 0.26]
Roberfroid 2008	-0.86	1.1	270	-0.94	1.15	313	3.1%	0.08 [-0.10, 0.26]
Sunawang 2009	-0.89	1.02	151	-0.82	0.98	148	2.0%	-0.07 [-0.30, 0.16]
Tofail 2008 (1)	-1.39	1.07	445	-1.36	1.08	885	6.8%	-0.03 [-0.15, 0.09]
West 2014	-1.3	1.06	4494	-1.36	1.08	4429	51.9%	0.06 [0.02, 0.10]
**Total (95% CI)**			**9229**			**9621**	**100.0%**	**0.04 [0.01, 0.07]**

Heterogeneity: Tau² = 0.00; Chi² = 6.07, df = 8 (P = 0.64); I² = 0%
Test for overall effect: Z = 2.46 (P = 0.01)

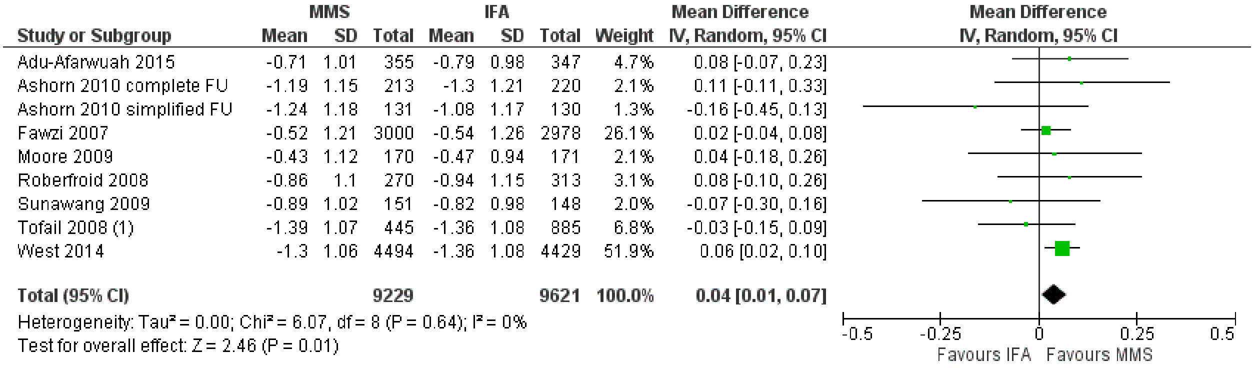

Footnotes
(1) Only the Usual Invitation to Food Supplementation groups were included. Both IFA arms were merged and compared with the MMS arm

Supplemental Figure 2.5.4 Effect of MMS vs IFA on LAZ at 12 months

Fixed

Study or Subgroup	MMS Mean	MMS SD	MMS Total	IFA Mean	IFA SD	IFA Total	Weight	Mean Difference IV, Fixed, 95% CI
Adu-Afarwuah 2015	-0.71	1.05	345	-0.84	1	332	5.6%	0.13 [-0.02, 0.28]
Ashorn 2010 complete FU	-1.48	1.23	214	-1.47	1.07	218	2.8%	-0.01 [-0.23, 0.21]
Fawzi 2007	-1.1	1.22	1630	-1.11	1.22	1606	18.7%	0.01 [-0.07, 0.09]
Roberfroid 2008	-1.38	1.1	240	-1.33	1.1	248	3.5%	-0.05 [-0.25, 0.15]
Sunawang 2009	-0.98	1.17	151	-0.99	0.97	148	2.2%	0.01 [-0.23, 0.25]
Tofail 2008 (1)	-1.68	1.09	502	-1.64	1.08	1023	9.8%	-0.04 [-0.16, 0.08]
West 2014	-1.74	1.04	2851	-1.77	1.03	4785	57.4%	0.03 [-0.02, 0.08]
**Total (95% CI)**			**5933**			**8360**	**100.0%**	**0.02 [-0.02, 0.06]**

Heterogeneity: Chi² = 3.77, df = 6 (P = 0.71); I² = 0%
Test for overall effect: Z = 1.11 (P = 0.27)

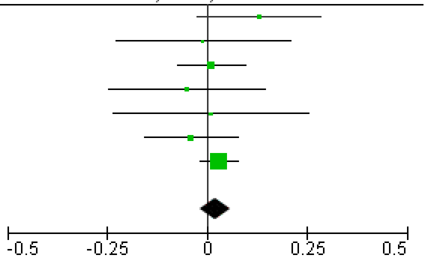

Footnotes
(1) Only the Usual Invitation to Food Supplementation groups were included. Both IFA arms were merged and compared with the MMS arm

Random

Study or Subgroup	MMS Mean	MMS SD	MMS Total	IFA Mean	IFA SD	IFA Total	Weight	Mean Difference IV, Random, 95% CI
Adu-Afarwuah 2015	-0.71	1.05	345	-0.84	1	332	5.6%	0.13 [-0.02, 0.28]
Ashorn 2010 complete FU	-1.48	1.23	214	-1.47	1.07	218	2.8%	-0.01 [-0.23, 0.21]
Fawzi 2007	-1.1	1.22	1630	-1.11	1.22	1606	18.7%	0.01 [-0.07, 0.09]
Roberfroid 2008	-1.38	1.1	240	-1.33	1.1	248	3.5%	-0.05 [-0.25, 0.15]
Sunawang 2009	-0.98	1.17	151	-0.99	0.97	148	2.2%	0.01 [-0.23, 0.25]
Tofail 2008 (1)	-1.68	1.09	502	-1.64	1.08	1023	9.8%	-0.04 [-0.16, 0.08]
West 2014	-1.74	1.04	2851	-1.77	1.03	4785	57.4%	0.03 [-0.02, 0.08]
**Total (95% CI)**			**5933**			**8360**	**100.0%**	**0.02 [-0.02, 0.06]**

Heterogeneity: Tau² = 0.00; Chi² = 3.77, df = 6 (P = 0.71); I² = 0%
Test for overall effect: Z = 1.11 (P = 0.27)

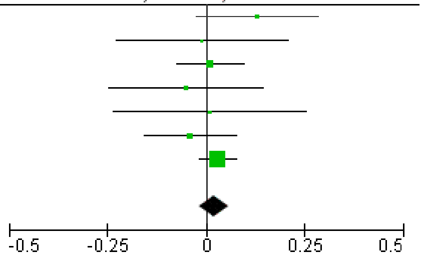

Footnotes
(1) Only the Usual Invitation to Food Supplementation groups were included. Both IFA arms were merged and compared with the MMS arm

Supplemental Figure 2.5.5 Effect of MMS vs IFA on LAZ at 18 months

Fixed

Study or Subgroup	MMS Mean	MMS SD	MMS Total	IFA Mean	IFA SD	IFA Total	Weight	Mean Difference IV, Fixed, 95% CI
Adu-Afarwuah 2015	-0.81	1.01	355	-0.97	0.97	340	28.7%	0.16 [0.01, 0.31]
Ashorn 2010 complete FU	-1.64	1.22	222	-1.63	1.02	220	14.2%	-0.01 [-0.22, 0.20]
Ashorn 2010 simplified FU	-1.88	0.95	129	-1.85	1.17	130	9.2%	-0.03 [-0.29, 0.23]
Fawzi 2007	-1.2	1	39	-1.57	1.1	55	3.4%	0.37 [-0.06, 0.80]
Tofail 2008 (1)	-2.03	1.06	463	-1.94	1.09	974	44.4%	-0.09 [-0.21, 0.03]
**Total (95% CI)**			**1208**			**1719**	**100.0%**	**0.01 [-0.06, 0.09]**

Heterogeneity: Chi² = 9.57, df = 4 (P = 0.05); I² = 58%
Test for overall effect: Z = 0.36 (P = 0.72)

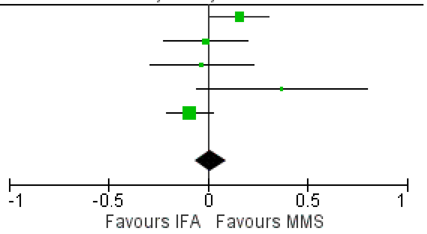

Footnotes
(1) Only the Usual Invitation to Food Supplementation groups were included. Both IFA arms were merged and compared with the MMS arm

Random

Study or Subgroup	MMS Mean	MMS SD	MMS Total	IFA Mean	IFA SD	IFA Total	Weight	Mean Difference IV, Random, 95% CI
Adu-Afarwuah 2015	-0.81	1.01	355	-0.97	0.97	340	26.3%	0.16 [0.01, 0.31]
Ashorn 2010 complete FU	-1.64	1.22	222	-1.63	1.02	220	20.1%	-0.01 [-0.22, 0.20]
Ashorn 2010 simplified FU	-1.88	0.95	129	-1.85	1.17	130	16.1%	-0.03 [-0.29, 0.23]
Fawzi 2007	-1.2	1	39	-1.57	1.1	55	8.1%	0.37 [-0.06, 0.80]
Tofail 2008 (1)	-2.03	1.06	463	-1.94	1.09	974	29.4%	-0.09 [-0.21, 0.03]
**Total (95% CI)**			**1208**			**1719**	**100.0%**	**0.04 [-0.10, 0.18]**

Heterogeneity: Tau² = 0.01; Chi² = 9.57, df = 4 (P = 0.05); I² = 58%
Test for overall effect: Z = 0.55 (P = 0.58)

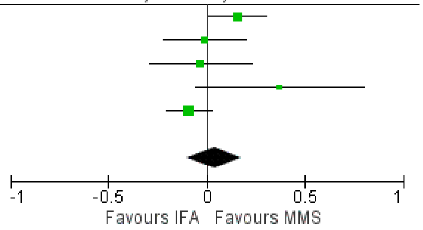

Footnotes
(1) Only the Usual Invitation to Food Supplementation groups were included. Both IFA arms were merged and compared with the MMS arm

Supplemental Figure 2.5.6 Effect of MMS vs IFA on LAZ at 24 months

Fixed

Study or Subgroup	MMS Mean	MMS SD	MMS Total	IFA Mean	IFA SD	IFA Total	Weight	Mean Difference IV, Fixed, 95% CI
Ashorn 2010 complete FU	-1.71	1.18	179	-1.69	1.01	197	3.7%	-0.02 [-0.24, 0.20]
Bliznashka 2021	-2.6	1.06	117	-2.53	0.99	131	2.8%	-0.07 [-0.33, 0.19]
Roberfroid 2008	-1.7	1.38	54	-1.76	1.46	55	0.6%	0.06 [-0.47, 0.59]
Tofail 2008 (1)	-2.1	1.09	479	-2.03	1.09	967	12.9%	-0.07 [-0.19, 0.05]
West 2014	-2.01	0.98	3283	-1.99	0.99	3231	80.0%	-0.02 [-0.07, 0.03]
**Total (95% CI)**			**4112**			**4581**	**100.0%**	**-0.03 [-0.07, 0.02]**

Heterogeneity: Chi² = 0.79, df = 4 (P = 0.94); I² = 0%
Test for overall effect: Z = 1.25 (P = 0.21)

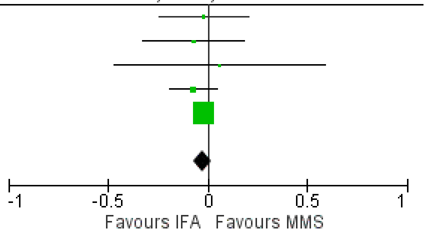

Footnotes
(1) Only the Usual Invitation to Food Supplementation groups were included. Both IFA arms were merged and compared with the MMS arm

Random

Study or Subgroup	MMS Mean	MMS SD	MMS Total	IFA Mean	IFA SD	IFA Total	Weight	Mean Difference IV, Random, 95% CI
Ashorn 2010 complete FU	-1.71	1.18	179	-1.69	1.01	197	3.7%	-0.02 [-0.24, 0.20]
Bliznashka 2021	-2.6	1.06	117	-2.53	0.99	131	2.8%	-0.07 [-0.33, 0.19]
Roberfroid 2008	-1.7	1.38	54	-1.76	1.46	55	0.6%	0.06 [-0.47, 0.59]
Tofail 2008 (1)	-2.1	1.09	479	-2.03	1.09	967	12.9%	-0.07 [-0.19, 0.05]
West 2014	-2.01	0.98	3283	-1.99	0.99	3231	80.0%	-0.02 [-0.07, 0.03]
**Total (95% CI)**			**4112**			**4581**	**100.0%**	**-0.03 [-0.07, 0.02]**

Heterogeneity: Tau² = 0.00; Chi² = 0.79, df = 4 (P = 0.94); I² = 0%
Test for overall effect: Z = 1.25 (P = 0.21)

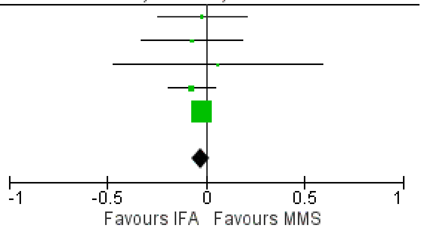

Footnotes
(1) Only the Usual Invitation to Food Supplementation groups were included. Both IFA arms were merged and compared with the MMS arm

Supplemental Figure 2.6. Effect of MMS vs IFA on WAZ

Supplemental Figure 2.6.1. Effect of MMS vs IFA on WAZ at birth

Fixed

	MMS			IFA				Mean Difference
**Study or Subgroup**	**Mean**	**SD**	**Total**	**Mean**	**SD**	**Total**	**Weight**	**IV, Fixed, 95% CI**
Adu-Afarwuah 2015	-0.62	1	318	-0.76	1.01	305	2.3%	0.14 [-0.02, 0.30]
Ashorn 2010	-0.57	1.02	374	-0.64	1.08	362	2.5%	0.07 [-0.08, 0.22]
Fawzi 2007	-0.39	1.17	3838	-0.52	1.18	3823	21.1%	0.13 [0.08, 0.18]
Moore 2009	-0.623	0.92	178	-0.605	0.893	167	1.6%	-0.02 [-0.21, 0.17]
Osrin 2005	-1.08	1.0325	529	-1.28	0.8031	523	4.7%	0.20 [0.09, 0.31]
Roberfroid 2008	-0.91	1.08	535	-0.97	0.97	541	3.9%	0.06 [-0.06, 0.18]
Sunawang 2009	-0.49	0.93	632	-0.49	0.99	649	5.3%	0.00 [-0.11, 0.11]
Tofail 2008 (1)	-1.51	1.04	490	-1.54	1.05	1012	4.6%	0.03 [-0.08, 0.14]
West 2014	-1.61	0.99	7127	-1.74	1.01	7041	53.9%	0.13 [0.10, 0.16]
**Total (95% CI)**			**14021**			**14423**	**100.0%**	**0.12 [0.09, 0.14]**

Heterogeneity: Chi² = 13.18, df = 8 (P = 0.11); I² = 39%
Test for overall effect: Z = 9.35 (P < 0.00001)

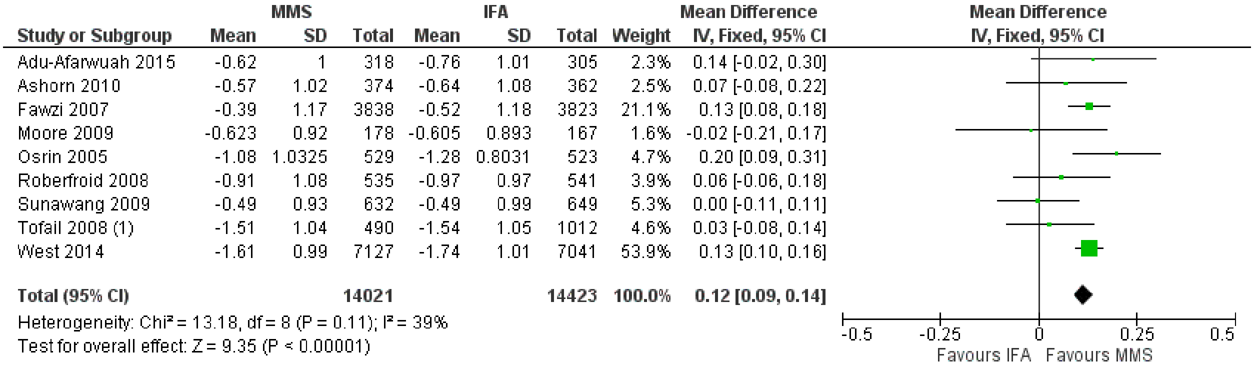

Footnotes
(1) Only the Usual Invitation to Food Supplementation groups were included. Both IFA arms were merged and compared with the MMS arm

Random

	MMS			IFA				Mean Difference
**Study or Subgroup**	**Mean**	**SD**	**Total**	**Mean**	**SD**	**Total**	**Weight**	**IV, Random, 95% CI**
Adu-Afarwuah 2015	-0.62	1	318	-0.76	1.01	305	5.3%	0.14 [-0.02, 0.30]
Ashorn 2010	-0.57	1.02	374	-0.64	1.08	362	5.7%	0.07 [-0.08, 0.22]
Fawzi 2007	-0.39	1.17	3838	-0.52	1.18	3823	21.3%	0.13 [0.08, 0.18]
Moore 2009	-0.623	0.92	178	-0.605	0.893	167	3.8%	-0.02 [-0.21, 0.17]
Osrin 2005	-1.08	1.0325	529	-1.28	0.8031	523	9.2%	0.20 [0.09, 0.31]
Roberfroid 2008	-0.91	1.08	535	-0.97	0.97	541	8.0%	0.06 [-0.06, 0.18]
Sunawang 2009	-0.49	0.93	632	-0.49	0.99	649	10.0%	0.00 [-0.11, 0.11]
Tofail 2008 (1)	-1.51	1.04	490	-1.54	1.05	1012	9.1%	0.03 [-0.08, 0.14]
West 2014	-1.61	0.99	7127	-1.74	1.01	7041	27.6%	0.13 [0.10, 0.16]
**Total (95% CI)**			**14021**			**14423**	**100.0%**	**0.10 [0.06, 0.14]**

Heterogeneity: Tau² = 0.00; Chi² = 13.18, df = 8 (P = 0.11); I² = 39%
Test for overall effect: Z = 4.95 (P < 0.00001)

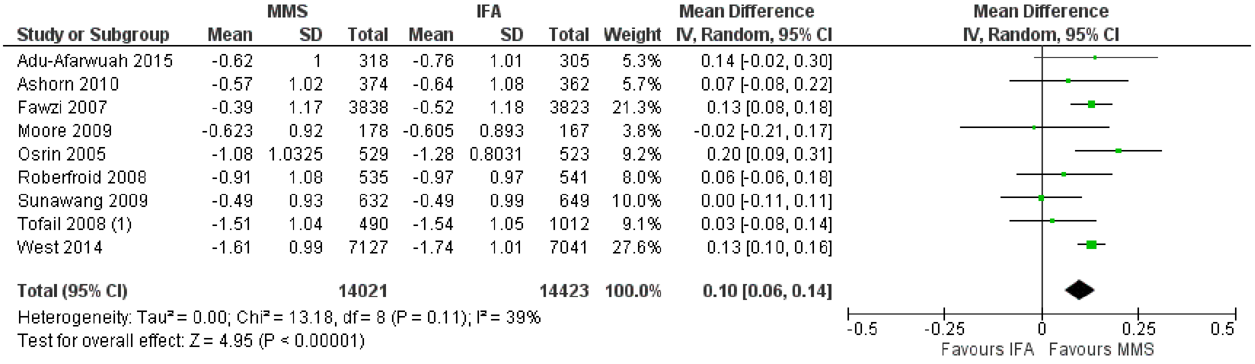

Footnotes
(1) Only the Usual Invitation to Food Supplementation groups were included. Both IFA arms were merged and compared with the MMS arm

Supplemental Figure 2.6.2 Effect of MMS vs IFA on WAZ at 3 months

Fixed

	MMS			IFA				Mean Difference
**Study or Subgroup**	**Mean**	**SD**	**Total**	**Mean**	**SD**	**Total**	**Weight**	**IV, Fixed, 95% CI**
Adu-Afarwuah 2015	-0.25	1.07	358	-0.43	1.11	343	2.8%	0.18 [0.02, 0.34]
Fawzi 2007	0.09	1.17	3011	0.08	1.19	3013	20.4%	0.01 [-0.05, 0.07]
Moore 2009	-0.537	1.013	179	-0.582	1.06	171	1.5%	0.04 [-0.17, 0.26]
Roberfroid 2008	-0.51	1.11	306	-0.64	1.13	349	2.5%	0.13 [-0.04, 0.30]
Sunawang 2009	-0.55	1.03	151	-0.58	0.95	148	1.4%	0.03 [-0.19, 0.25]
Tofail 2008 (1)	-1.22	1.08	373	-1.24	1.09	794	4.1%	0.02 [-0.11, 0.15]
West 2014	-1.35	1.03	7996	-1.48	1.07	7777	67.3%	0.13 [0.10, 0.16]
**Total (95% CI)**			**12374**			**12595**	**100.0%**	**0.10 [0.07, 0.13]**

Heterogeneity: Chi² = 15.04, df = 6 (P = 0.02); I² = 60%
Test for overall effect: Z = 7.26 (P < 0.00001)

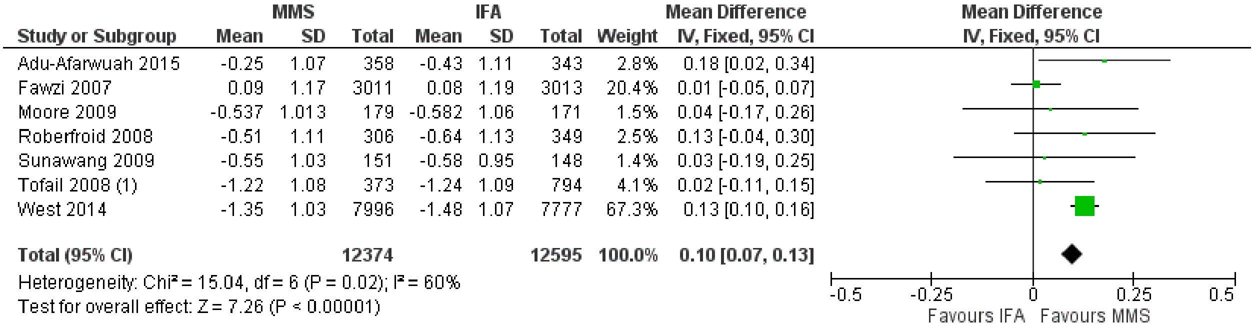

Footnotes
(1) Only the Usual Invitation to Food Supplementation groups were included. Both IFA arms were merged and compared with the MMS arm

Random

	MMS			IFA				Mean Difference
**Study or Subgroup**	**Mean**	**SD**	**Total**	**Mean**	**SD**	**Total**	**Weight**	**IV, Random, 95% CI**
Adu-Afarwuah 2015	-0.25	1.07	358	-0.43	1.11	343	10.4%	0.18 [0.02, 0.34]
Fawzi 2007	0.09	1.17	3011	0.08	1.19	3013	24.6%	0.01 [-0.05, 0.07]
Moore 2009	-0.537	1.013	179	-0.582	1.06	171	6.8%	0.04 [-0.17, 0.26]
Roberfroid 2008	-0.51	1.11	306	-0.64	1.13	349	9.6%	0.13 [-0.04, 0.30]
Sunawang 2009	-0.55	1.03	151	-0.58	0.95	148	6.4%	0.03 [-0.19, 0.25]
Tofail 2008 (1)	-1.22	1.08	373	-1.24	1.09	794	13.3%	0.02 [-0.11, 0.15]
West 2014	-1.35	1.03	7996	-1.48	1.07	7777	28.9%	0.13 [0.10, 0.16]
**Total (95% CI)**			**12374**			**12595**	**100.0%**	**0.08 [0.02, 0.14]**

Heterogeneity: Tau² = 0.00; Chi² = 15.04, df = 6 (P = 0.02); I² = 60%
Test for overall effect: Z = 2.42 (P = 0.02)

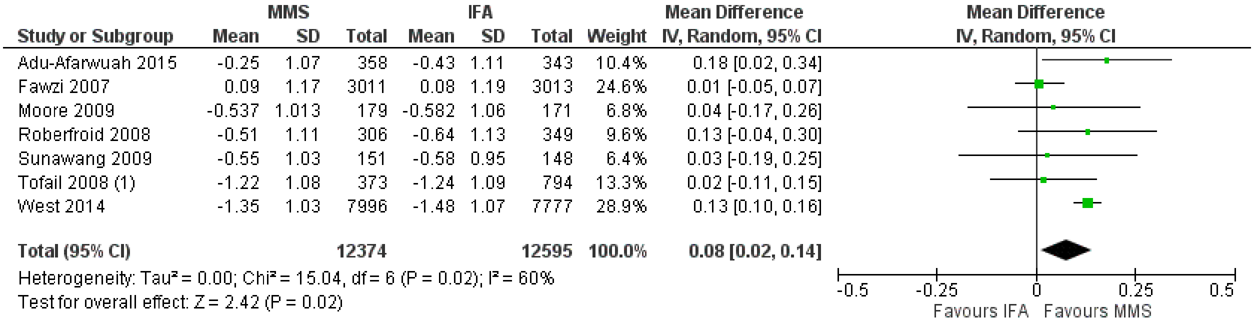

Footnotes
(1) Only the Usual Invitation to Food Supplementation groups were included. Both IFA arms were merged and compared with the MMS arm

Supplemental Figure 2.6.3 Effect of MMS vs IFA on WAZ at 6 months

Fixed

Study or Subgroup	MMS Mean	MMS SD	MMS Total	IFA Mean	IFA SD	IFA Total	Weight	Mean Difference IV, Fixed, 95% CI
Adu-Afarwuah 2015	-0.48	1.1	356	-0.61	1.15	347	3.5%	0.13 [-0.04, 0.30]
Ashorn 2010 complete FU	-0.54	1.25	216	-0.57	1.19	219	1.9%	0.03 [-0.20, 0.26]
Ashorn 2010 simplified FU	-0.54	1.25	131	-0.55	1.07	130	1.2%	0.01 [-0.27, 0.29]
Fawzi 2007	-0.22	1.18	3050	-0.24	1.17	3023	28.1%	0.02 [-0.04, 0.08]
Moore 2009	-0.661	1.061	169	-0.697	1.136	172	1.8%	0.04 [-0.20, 0.27]
Roberfroid 2008	-0.93	1.24	270	-1.07	1.17	313	2.5%	0.14 [-0.06, 0.34]
Sunawang 2009	-0.75	1.01	151	-0.7	1.01	148	1.9%	-0.05 [-0.28, 0.18]
Tofail 2008 (1)	-1.11	1.13	447	-1.12	1.13	893	6.0%	0.01 [-0.12, 0.14]
West 2014	-1.32	1.04	4539	-1.36	1.04	4467	53.1%	0.04 [-0.00, 0.08]
**Total (95% CI)**			**9329**			**9712**	**100.0%**	**0.04 [0.00, 0.07]**

Heterogeneity: Chi² = 3.35, df = 8 (P = 0.91); I² = 0%
Test for overall effect: Z = 2.25 (P = 0.02)

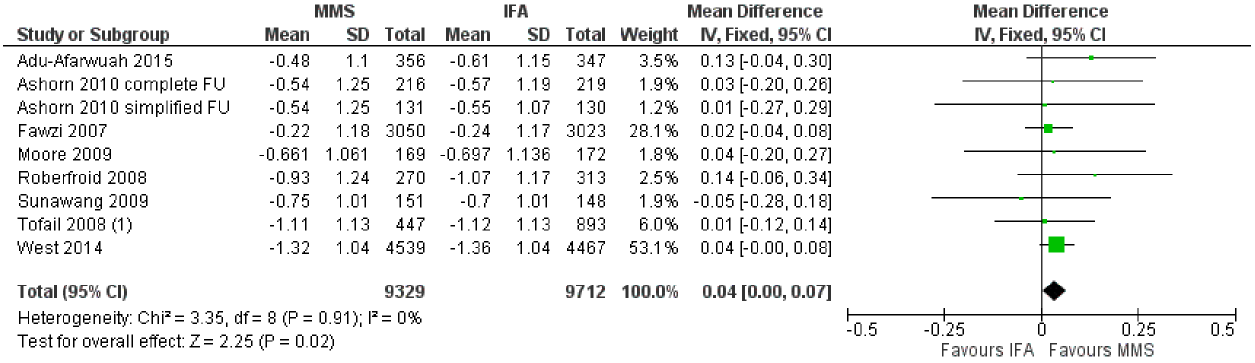

Footnotes
(1) Only the Usual Invitation to Food Supplementation groups were included. Both IFA arms were merged and compared with the MMS arm

Random

Study or Subgroup	MMS Mean	MMS SD	MMS Total	IFA Mean	IFA SD	IFA Total	Weight	Mean Difference IV, Random, 95% CI
Adu-Afarwuah 2015	-0.48	1.1	356	-0.61	1.15	347	3.5%	0.13 [-0.04, 0.30]
Ashorn 2010 complete FU	-0.54	1.25	216	-0.57	1.19	219	1.9%	0.03 [-0.20, 0.26]
Ashorn 2010 simplified FU	-0.54	1.25	131	-0.55	1.07	130	1.2%	0.01 [-0.27, 0.29]
Fawzi 2007	-0.22	1.18	3050	-0.24	1.17	3023	28.1%	0.02 [-0.04, 0.08]
Moore 2009	-0.661	1.061	169	-0.697	1.136	172	1.8%	0.04 [-0.20, 0.27]
Roberfroid 2008	-0.93	1.24	270	-1.07	1.17	313	2.5%	0.14 [-0.06, 0.34]
Sunawang 2009	-0.75	1.01	151	-0.7	1.01	148	1.9%	-0.05 [-0.28, 0.18]
Tofail 2008 (1)	-1.11	1.13	447	-1.12	1.13	893	6.0%	0.01 [-0.12, 0.14]
West 2014	-1.32	1.04	4539	-1.36	1.04	4467	53.1%	0.04 [-0.00, 0.08]
**Total (95% CI)**			**9329**			**9712**	**100.0%**	**0.04 [0.00, 0.07]**

Heterogeneity: Tau² = 0.00; Chi² = 3.35, df = 8 (P = 0.91); I² = 0%
Test for overall effect: Z = 2.25 (P = 0.02)

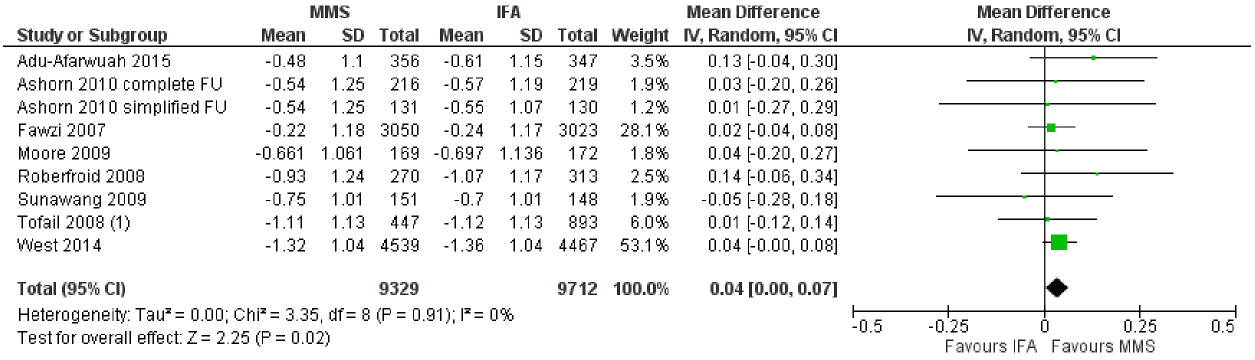

Footnotes
(1) Only the Usual Invitation to Food Supplementation groups were included. Both IFA arms were merged and compared with the MMS arm

Supplemental Figure 2.6.4 Effect of MMS vs IFA on WAZ at 12 months

Fixed

Study or Subgroup	MMS Mean	MMS SD	MMS Total	IFA Mean	IFA SD	IFA Total	Weight	Mean Difference IV, Fixed, 95% CI
Adu-Afarwuah 2015	-0.69	1.05	346	-0.87	1.12	332	3.9%	0.18 [0.02, 0.34]
Ashorn 2010 complete FU	-0.83	1.22	215	-0.72	1.03	219	2.3%	-0.11 [-0.32, 0.10]
Fawzi 2007	-0.67	1.18	1644	-0.69	1.21	1620	15.4%	0.02 [-0.06, 0.10]
Roberfroid 2008	-1.59	1.14	240	-1.53	1.07	248	2.7%	-0.06 [-0.26, 0.14]
Sunawang 2009	-0.476	0.93	151	-0.47	0.87	148	2.5%	-0.01 [-0.21, 0.20]
Tofail 2008 (1)	-1.35	1.16	504	-1.41	1.13	1028	6.9%	0.06 [-0.06, 0.18]
West 2014	-1.7	1	4961	-1.72	1	4867	66.3%	0.02 [-0.02, 0.06]
**Total (95% CI)**			**8061**			**8462**	**100.0%**	**0.02 [-0.01, 0.06]**

Heterogeneity: Chi² = 6.18, df = 6 (P = 0.40); I² = 3%
Test for overall effect: Z = 1.41 (P = 0.16)

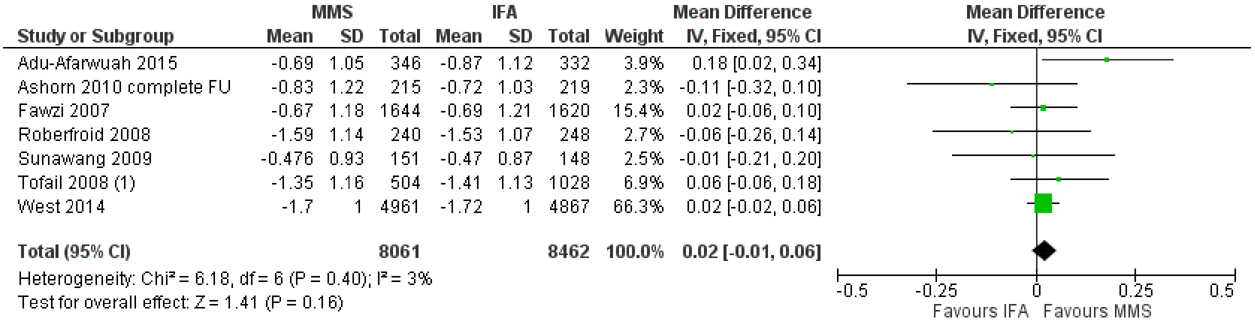

Footnotes
(1) Only the Usual Invitation to Food Supplementation groups were included. Both IFA arms were merged and compared with the MMS arm

Random

Study or Subgroup	MMS Mean	MMS SD	MMS Total	IFA Mean	IFA SD	IFA Total	Weight	Mean Difference IV, Random, 95% CI
Adu-Afarwuah 2015	-0.69	1.05	346	-0.87	1.12	332	4.4%	0.18 [0.02, 0.34]
Ashorn 2010 complete FU	-0.83	1.22	215	-0.72	1.03	219	2.6%	-0.11 [-0.32, 0.10]
Fawzi 2007	-0.67	1.18	1644	-0.69	1.21	1620	16.9%	0.02 [-0.06, 0.10]
Roberfroid 2008	-1.59	1.14	240	-1.53	1.07	248	3.1%	-0.06 [-0.26, 0.14]
Sunawang 2009	-0.476	0.93	151	-0.47	0.87	148	2.9%	-0.01 [-0.21, 0.20]
Tofail 2008 (1)	-1.35	1.16	504	-1.41	1.13	1028	7.8%	0.06 [-0.06, 0.18]
West 2014	-1.7	1	4961	-1.72	1	4867	62.3%	0.02 [-0.02, 0.06]
**Total (95% CI)**			**8061**			**8462**	**100.0%**	**0.02 [-0.01, 0.06]**

Heterogeneity: Tau² = 0.00; Chi² = 6.18, df = 6 (P = 0.40); I² = 3%
Test for overall effect: Z = 1.33 (P = 0.18)

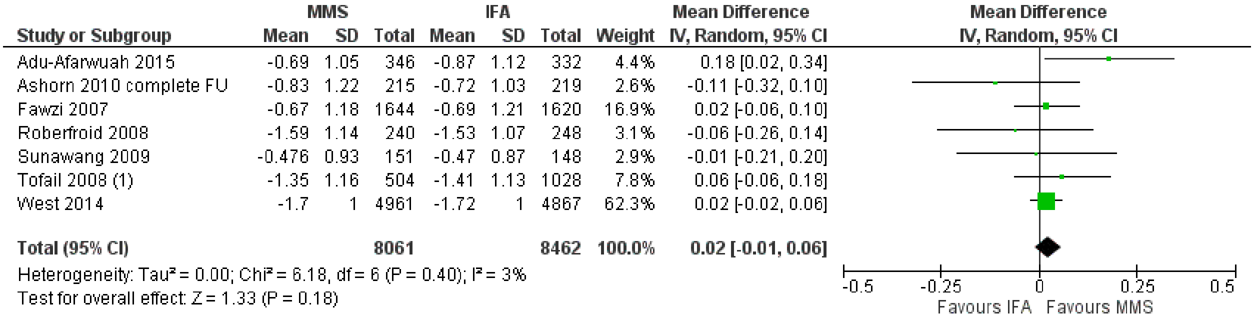

Footnotes
(1) Only the Usual Invitation to Food Supplementation groups were included. Both IFA arms were merged and compared with the MMS arm

Supplemental Figure 2.6.5 Effect of MMS vs IFA on WAZ at 18 months

Fixed

Study or Subgroup	MMS Mean	MMS SD	MMS Total	IFA Mean	IFA SD	IFA Total	Weight	Mean Difference IV, Fixed, 95% CI
Adu-Afarwuah 2015	-0.78	1.01	356	-0.93	1.04	337	27.6%	0.15 [-0.00, 0.30]
Ashorn 2010 complete FU	-0.93	1.12	222	-0.83	0.98	220	16.8%	-0.10 [-0.30, 0.10]
Ashorn 2010 simplified FU	-1.01	1.01	130	-1.02	1.05	129	10.2%	0.01 [-0.24, 0.26]
Fawzi 2007	-0.89	1.25	40	-1.02	1.27	55	2.5%	0.13 [-0.38, 0.64]
Tofail 2008 (1)	-1.88	1.12	463	-1.94	1.08	974	42.9%	0.06 [-0.06, 0.18]
**Total (95% CI)**			**1211**			**1715**	**100.0%**	**0.05 [-0.03, 0.13]**

Heterogeneity: Chi² = 4.10, df = 4 (P = 0.39); I² = 2%
Test for overall effect: Z = 1.33 (P = 0.18)

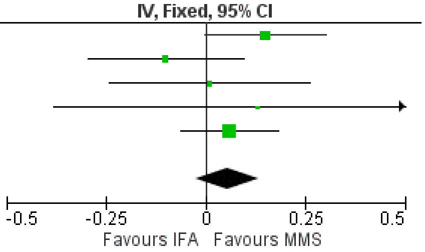

Footnotes
(1) Only the Usual Invitation to Food Supplementation groups were included. Both IFA arms were merged and compared with the MMS arm

Random

Study or Subgroup	MMS Mean	MMS SD	MMS Total	IFA Mean	IFA SD	IFA Total	Weight	Mean Difference IV, Random, 95% CI
Adu-Afarwuah 2015	-0.78	1.01	356	-0.93	1.04	337	27.7%	0.15 [-0.00, 0.30]
Ashorn 2010 complete FU	-0.93	1.12	222	-0.83	0.98	220	17.0%	-0.10 [-0.30, 0.10]
Ashorn 2010 simplified FU	-1.01	1.01	130	-1.02	1.05	129	10.5%	0.01 [-0.24, 0.26]
Fawzi 2007	-0.89	1.25	40	-1.02	1.27	55	2.5%	0.13 [-0.38, 0.64]
Tofail 2008 (1)	-1.88	1.12	463	-1.94	1.08	974	42.2%	0.06 [-0.06, 0.18]
**Total (95% CI)**			**1211**			**1715**	**100.0%**	**0.05 [-0.03, 0.14]**

Heterogeneity: Tau² = 0.00; Chi² = 4.10, df = 4 (P = 0.39); I² = 2%
Test for overall effect: Z = 1.30 (P = 0.19)

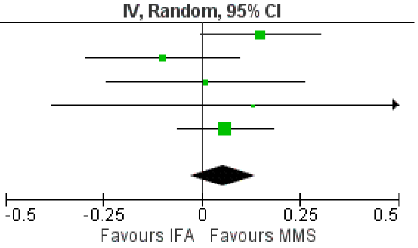

Footnotes
(1) Only the Usual Invitation to Food Supplementation groups were included. Both IFA arms were merged and compared with the MMS arm

Supplemental Figure 2.6.6 Effect of MMS vs IFA on WAZ at 24 months

Fixed

Study or Subgroup	MMS Mean	MMS SD	MMS Total	IFA Mean	IFA SD	IFA Total	Weight	Mean Difference IV, Fixed, 95% CI
Ashorn 2010 complete FU	-1.12	1.14	177	-1	0.91	196	3.7%	-0.12 [-0.33, 0.09]
Bliznashka 2021	-1.8	2.2544	642	-1.75	2.2544	718	2.8%	-0.05 [-0.29, 0.19]
Roberfroid 2008	-2.52	1.04	54	-2.63	1.29	55	0.8%	0.11 [-0.33, 0.55]
Tofail 2008 (1)	-1.68	1.05	481	-1.69	1.07	968	12.2%	0.01 [-0.11, 0.13]
West 2014	-1.97	0.94	3412	-1.98	0.95	3370	80.5%	0.01 [-0.03, 0.05]
**Total (95% CI)**			**4766**			**5307**	**100.0%**	**0.00 [-0.04, 0.04]**

Heterogeneity: Chi² = 1.83, df = 4 (P = 0.77); I² = 0%
Test for overall effect: Z = 0.21 (P = 0.83)

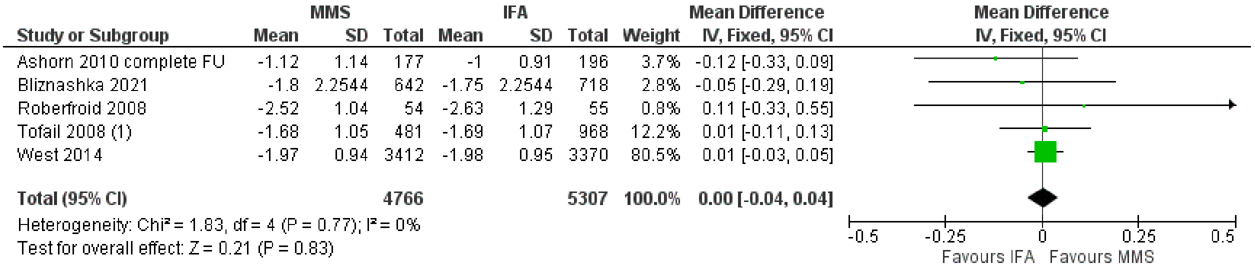

Footnotes
(1) Only the Usual Invitation to Food Supplementation groups were included. Both IFA arms were merged and compared with the MMS arm

Random

Study or Subgroup	MMS Mean	MMS SD	MMS Total	IFA Mean	IFA SD	IFA Total	Weight	Mean Difference IV, Random, 95% CI
Ashorn 2010 complete FU	-1.12	1.14	177	-1	0.91	196	3.7%	-0.12 [-0.33, 0.09]
Bliznashka 2021	-1.8	2.2544	642	-1.75	2.2544	718	2.8%	-0.05 [-0.29, 0.19]
Roberfroid 2008	-2.52	1.04	54	-2.63	1.29	55	0.8%	0.11 [-0.33, 0.55]
Tofail 2008 (1)	-1.68	1.05	481	-1.69	1.07	968	12.2%	0.01 [-0.11, 0.13]
West 2014	-1.97	0.94	3412	-1.98	0.95	3370	80.5%	0.01 [-0.03, 0.05]
**Total (95% CI)**			**4766**			**5307**	**100.0%**	**0.00 [-0.04, 0.04]**

Heterogeneity: Tau² = 0.00; Chi² = 1.83, df = 4 (P = 0.77); I² = 0%
Test for overall effect: Z = 0.21 (P = 0.83)

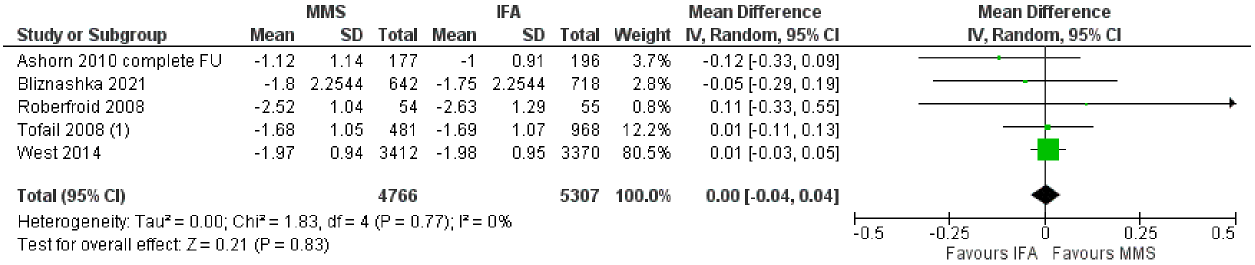

Footnotes
(1) Only the Usual Invitation to Food Supplementation groups were included. Both IFA arms were merged and compared with the MMS arm

Supplemental Figure 2.7. Effect of MMS vs IFA on WLZ

Supplemental Figure 2.7.1. Effect of MMS vs IFA on WLZ or BMIZ at birth

Fixed

Study or Subgroup	MMS Mean	MMS SD	MMS Total	IFA Mean	IFA SD	IFA Total	Weight	Mean Difference IV, Fixed, 95% CI
Adu-Afarwuah 2015 (1)	-0.56	1.03	313	-0.71	1	305	3.6%	0.15 [-0.01, 0.31]
Ashorn 2010	0.07	1.08	364	0.09	1.18	344	3.3%	-0.02 [-0.19, 0.15]
Fawzi 2007 (2)	0.6	3.47	2777	0.4	3.47	2781	2.8%	0.20 [0.02, 0.38]
Osrin 2005 (3)	-1.42	1.2532	501	-1.63	1.2532	501	3.9%	0.21 [0.05, 0.37]
Roberfroid 2008 (4)	-0.86	1.22	534	-0.81	1.1	536	4.8%	-0.05 [-0.19, 0.09]
Sunawang 2009	0.1	0.9	632	0.09	0.98	649	8.8%	0.01 [-0.09, 0.11]
Tofail 2008 (5)	-1.55	1.12	483	-1.63	1.16	1010	6.2%	0.08 [-0.04, 0.20]
West 2014	-0.71	1.01	5695	-0.8	1	5459	66.7%	0.09 [0.05, 0.13]
**Total (95% CI)**			**11299**			**11585**	**100.0%**	**0.08 [0.05, 0.11]**

Heterogeneity: Chi² = 11.86, df = 7 (P = 0.11); I² = 41%
Test for overall effect: Z = 5.27 (P < 0.00001)

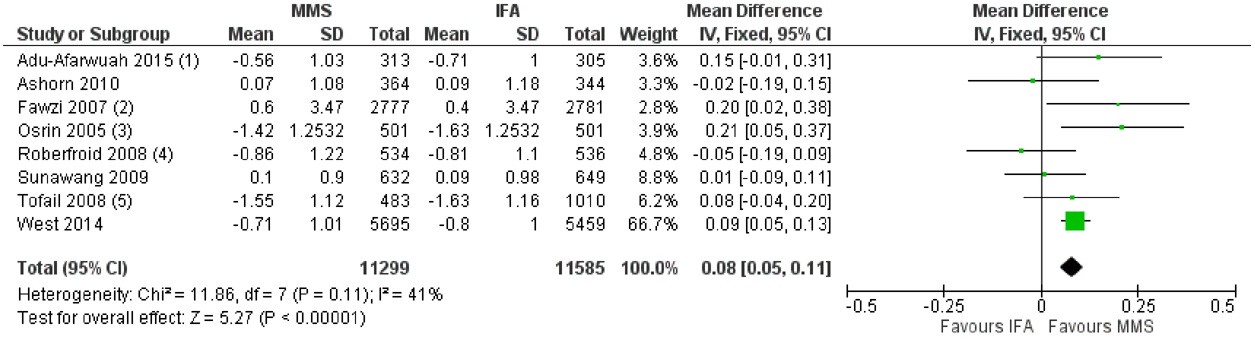

Footnotes
(1) BMI-for-age z score
(2) BMI-for-age z score
(3) BMI-for-age z score
(4) BMI-for-age z score
(5) BMI-for-age z score. Only the Usual Invitation to Food Supplementation groups were included. Both IFA arms were merged and compared with the...

Random

Study or Subgroup	MMS Mean	MMS SD	MMS Total	IFA Mean	IFA SD	IFA Total	Weight	Mean Difference IV, Random, 95% CI
Adu-Afarwuah 2015 (1)	-0.56	1.03	313	-0.71	1	305	8.5%	0.15 [-0.01, 0.31]
Ashorn 2010	0.07	1.08	364	0.09	1.18	344	8.0%	-0.02 [-0.19, 0.15]
Fawzi 2007 (2)	0.6	3.47	2777	0.4	3.47	2781	7.0%	0.20 [0.02, 0.38]
Osrin 2005 (3)	-1.42	1.2532	501	-1.63	1.2532	501	8.9%	0.21 [0.05, 0.37]
Roberfroid 2008 (4)	-0.86	1.22	534	-0.81	1.1	536	10.4%	-0.05 [-0.19, 0.09]
Sunawang 2009	0.1	0.9	632	0.09	0.98	649	15.2%	0.01 [-0.09, 0.11]
Tofail 2008 (5)	-1.55	1.12	483	-1.63	1.16	1010	12.3%	0.08 [-0.04, 0.20]
West 2014	-0.71	1.01	5695	-0.8	1	5459	29.6%	0.09 [0.05, 0.13]
**Total (95% CI)**			**11299**			**11585**	**100.0%**	**0.08 [0.02, 0.13]**

Heterogeneity: Tau² = 0.00; Chi² = 11.86, df = 7 (P = 0.11); I² = 41%
Test for overall effect: Z = 2.79 (P = 0.005)

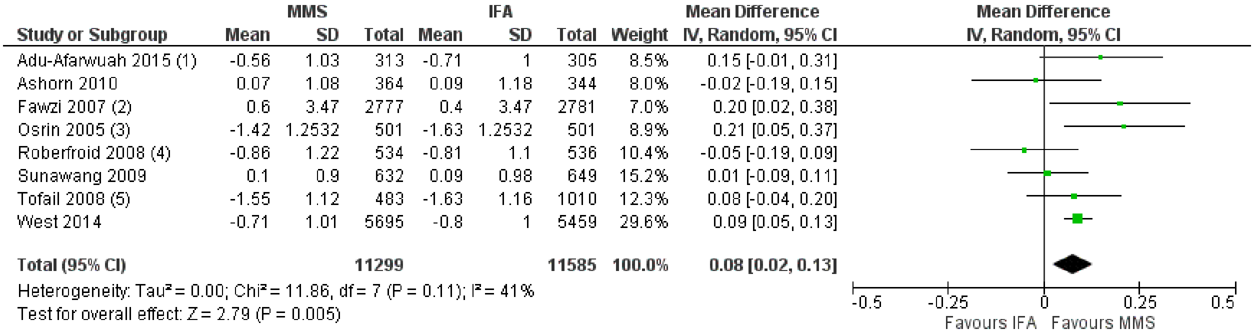

Footnotes
(1) BMI-for-age z score
(2) BMI-for-age z score
(3) BMI-for-age z score
(4) BMI-for-age z score
(5) BMI-for-age z score. Only the Usual Invitation to Food Supplementation groups were included. Both IFA arms were merged and compared with the MMS...

Supplemental Figure 2.7.2 Effect of MMS vs IFA on WLZ at 3 months

Fixed

	MMS			IFA				Mean Difference
Study or Subgroup	Mean	SD	Total	Mean	SD	Total	Weight	IV, Fixed, 95% CI
Adu-Afarwuah 2015	0.37	1.11	357	0.32	1.17	343	2.8%	0.05 [-0.12, 0.22]
Fawzi 2007	0.39	1.29	2897	0.42	1.29	2889	18.4%	-0.03 [-0.10, 0.04]
Moore 2009	-0.323	1.223	179	-0.319	1.137	171	1.3%	-0.00 [-0.25, 0.24]
Roberfroid 2008	0.25	1.25	306	0.12	1.15	349	2.4%	0.13 [-0.05, 0.31]
Sunawang 2009	0.07	1.09	151	0	1.15	148	1.3%	0.07 [-0.18, 0.32]
Tofail 2008 (1)	-0.09	1.19	370	-0.15	1.18	782	3.8%	0.06 [-0.09, 0.21]
West 2014	-0.39	1.09	7967	-0.43	1.09	7779	70.0%	0.04 [0.01, 0.07]
**Total (95% CI)**			**12227**			**12461**	**100.0%**	**0.03 [0.00, 0.06]**

Heterogeneity: Chi² = 4.97, df = 6 (P = 0.55); I² = 0%
Test for overall effect: Z = 2.07 (P = 0.04)

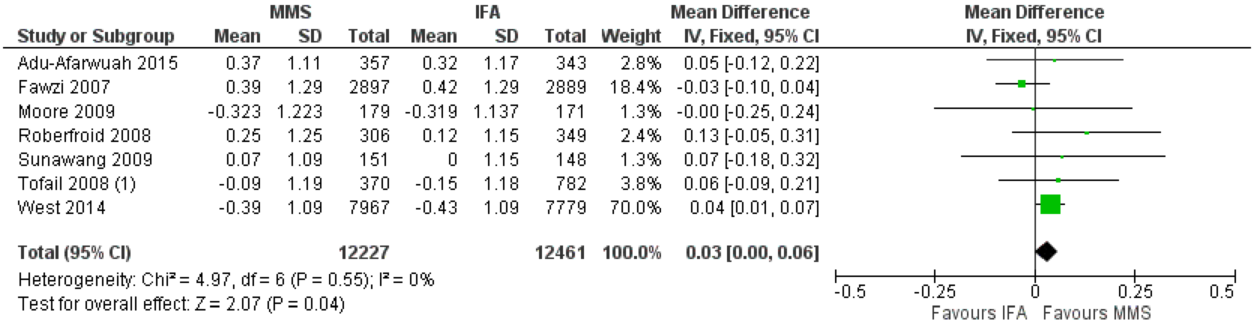

Footnotes
(1) Only the Usual Invitation to Food Supplementation groups were included. Both IFA arms were merged and compared with the MMS arm

Random

	MMS			IFA				Mean Difference
Study or Subgroup	Mean	SD	Total	Mean	SD	Total	Weight	IV, Random, 95% CI
Adu-Afarwuah 2015	0.37	1.11	357	0.32	1.17	343	2.8%	0.05 [-0.12, 0.22]
Fawzi 2007	0.39	1.29	2897	0.42	1.29	2889	18.4%	-0.03 [-0.10, 0.04]
Moore 2009	-0.323	1.223	179	-0.319	1.137	171	1.3%	-0.00 [-0.25, 0.24]
Roberfroid 2008	0.25	1.25	306	0.12	1.15	349	2.4%	0.13 [-0.05, 0.31]
Sunawang 2009	0.07	1.09	151	0	1.15	148	1.3%	0.07 [-0.18, 0.32]
Tofail 2008 (1)	-0.09	1.19	370	-0.15	1.18	782	3.8%	0.06 [-0.09, 0.21]
West 2014	-0.39	1.09	7967	-0.43	1.09	7779	70.0%	0.04 [0.01, 0.07]
**Total (95% CI)**			**12227**			**12461**	**100.0%**	**0.03 [0.00, 0.06]**

Heterogeneity: Tau² = 0.00; Chi² = 4.97, df = 6 (P = 0.55); I² = 0%
Test for overall effect: Z = 2.07 (P = 0.04)

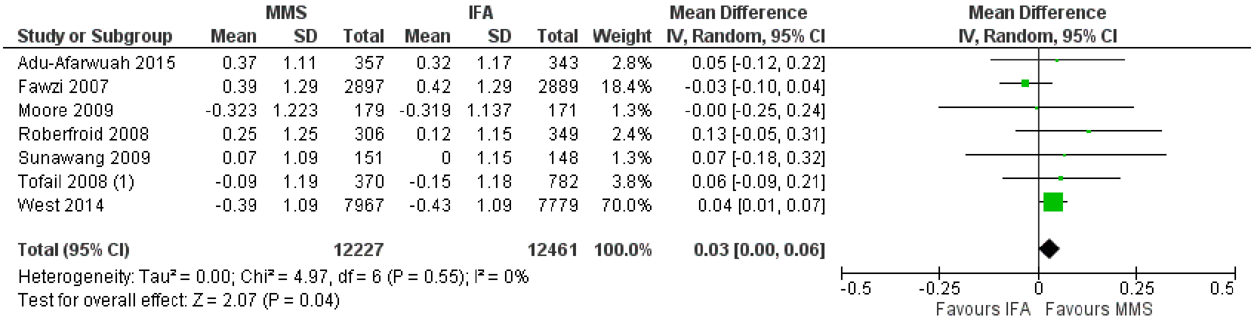

Footnotes
(1) Only the Usual Invitation to Food Supplementation groups were included. Both IFA arms were merged and compared with the MMS arm

Supplemental Figure 2.7.3 Effect of MMS vs IFA on WLZ at 6 months

Fixed

	MMS			IFA				Mean Difference
**Study or Subgroup**	**Mean**	**SD**	**Total**	**Mean**	**SD**	**Total**	**Weight**	**IV, Fixed, 95% CI**
Adu-Afarwuah 2015	0.03	1.08	355	-0.08	1.15	347	3.8%	0.11 [-0.06, 0.28]
Ashorn 2010 complete FU	0.36	1.19	213	0.43	1.15	219	2.1%	-0.07 [-0.29, 0.15]
Ashorn 2010 simplified FU	0.41	1.16	131	0.26	1.26	130	1.2%	0.15 [-0.14, 0.44]
Fawzi 2007	0.21	1.29	2998	0.2	1.26	2971	24.6%	0.01 [-0.05, 0.07]
Moore 2009	-0.431	1.128	169	-0.474	1.216	172	1.7%	0.04 [-0.21, 0.29]
Roberfroid 2008	-0.85	1.1	270	-0.94	1.1	313	3.2%	0.09 [-0.09, 0.27]
Sunawang 2009	-0.17	1.03	151	-0.18	1.09	148	1.8%	0.01 [-0.23, 0.25]
Tofail 2008 (1)	-1.39	1.07	445	-1.36	1.08	885	6.9%	-0.03 [-0.15, 0.09]
West 2014	-0.6	1.05	4503	-0.59	1.04	4448	54.8%	-0.01 [-0.05, 0.03]
**Total (95% CI)**			**9235**			**9633**	**100.0%**	**0.00 [-0.03, 0.04]**

Heterogeneity: Chi² = 4.67, df = 8 (P = 0.79); I² = 0%
Test for overall effect: Z = 0.19 (P = 0.85)

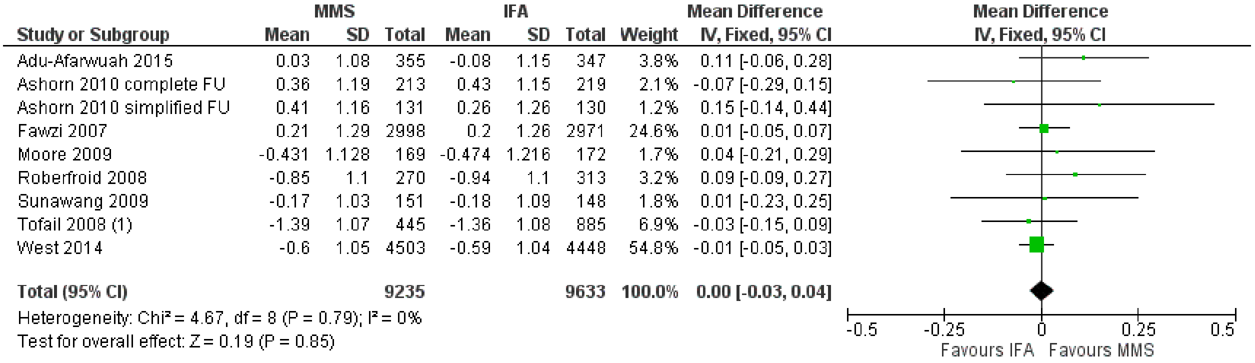

Footnotes
(1) Only the Usual Invitation to Food Supplementation groups were included. Both IFA arms were merged and compared with the MMS arm

Random

	MMS			IFA				Mean Difference
**Study or Subgroup**	**Mean**	**SD**	**Total**	**Mean**	**SD**	**Total**	**Weight**	**IV, Random, 95% CI**
Adu-Afarwuah 2015	0.03	1.08	355	-0.08	1.15	347	3.8%	0.11 [-0.06, 0.28]
Ashorn 2010 complete FU	0.36	1.19	213	0.43	1.15	219	2.1%	-0.07 [-0.29, 0.15]
Ashorn 2010 simplified FU	0.41	1.16	131	0.26	1.26	130	1.2%	0.15 [-0.14, 0.44]
Fawzi 2007	0.21	1.29	2998	0.2	1.26	2971	24.6%	0.01 [-0.05, 0.07]
Moore 2009	-0.431	1.128	169	-0.474	1.216	172	1.7%	0.04 [-0.21, 0.29]
Roberfroid 2008	-0.85	1.1	270	-0.94	1.1	313	3.2%	0.09 [-0.09, 0.27]
Sunawang 2009	-0.17	1.03	151	-0.18	1.09	148	1.8%	0.01 [-0.23, 0.25]
Tofail 2008 (1)	-1.39	1.07	445	-1.36	1.08	885	6.9%	-0.03 [-0.15, 0.09]
West 2014	-0.6	1.05	4503	-0.59	1.04	4448	54.8%	-0.01 [-0.05, 0.03]
**Total (95% CI)**			**9235**			**9633**	**100.0%**	**0.00 [-0.03, 0.04]**

Heterogeneity: Tau² = 0.00; Chi² = 4.67, df = 8 (P = 0.79); I² = 0%
Test for overall effect: Z = 0.19 (P = 0.85)

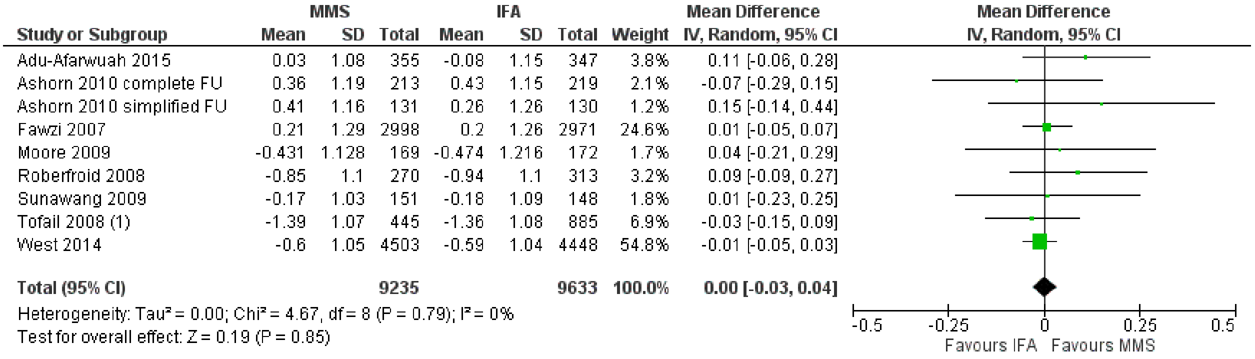

Footnotes
(1) Only the Usual Invitation to Food Supplementation groups were included. Both IFA arms were merged and compared with the MMS arm

Supplemental Figure 2.7.4 Effect of MMS vs IFA on WLZ at 12 months

Fixed

Study or Subgroup	MMS Mean	MMS SD	MMS Total	IFA Mean	IFA SD	IFA Total	Weight	Mean Difference IV, Fixed, 95% CI
Adu-Afarwuah 2015	-0.46	1.05	345	-0.63	1.13	332	6.4%	0.17 [0.01, 0.33]
Ashorn 2010 complete FU	-0.12	1.09	214	0	1.06	218	4.2%	-0.12 [-0.32, 0.08]
Fawzi 2007	-0.16	1.24	1628	-0.19	1.26	1607	23.4%	0.03 [-0.06, 0.12]
Roberfroid 2008	-1.11	1.1	240	-1.18	1	248	5.0%	0.07 [-0.12, 0.26]
Sunawang 2009	0.44	0.87	151	0.58	0.88	148	4.4%	-0.14 [-0.34, 0.06]
Tofail 2008 (1)	-0.68	1.13	502	-0.78	1.089	1023	12.2%	0.10 [-0.02, 0.22]
West 2014	-1.12	1.99	4856	-1.13	1	4785	44.2%	0.01 [-0.05, 0.07]
**Total (95% CI)**			**7936**			**8361**	**100.0%**	**0.03 [-0.01, 0.07]**

Heterogeneity: Chi² = 9.57, df = 6 (P = 0.14); I² = 37%
Test for overall effect: Z = 1.26 (P = 0.21)

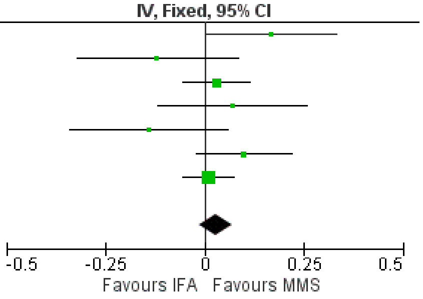

Footnotes
(1) Only the Usual Invitation to Food Supplementation groups were included. Both IFA arms were merged and compared with the MMS arm

Random

Study or Subgroup	MMS Mean	MMS SD	MMS Total	IFA Mean	IFA SD	IFA Total	Weight	Mean Difference IV, Random, 95% CI
Adu-Afarwuah 2015	-0.46	1.05	345	-0.63	1.13	332	10.1%	0.17 [0.01, 0.33]
Ashorn 2010 complete FU	-0.12	1.09	214	0	1.06	218	7.2%	-0.12 [-0.32, 0.08]
Fawzi 2007	-0.16	1.24	1628	-0.19	1.26	1607	22.4%	0.03 [-0.06, 0.12]
Roberfroid 2008	-1.11	1.1	240	-1.18	1	248	8.3%	0.07 [-0.12, 0.26]
Sunawang 2009	0.44	0.87	151	0.58	0.88	148	7.5%	-0.14 [-0.34, 0.06]
Tofail 2008 (1)	-0.68	1.13	502	-0.78	1.089	1023	15.8%	0.10 [-0.02, 0.22]
West 2014	-1.12	1.99	4856	-1.13	1	4785	28.7%	0.01 [-0.05, 0.07]
**Total (95% CI)**			**7936**			**8361**	**100.0%**	**0.03 [-0.03, 0.09]**

Heterogeneity: Tau² = 0.00; Chi² = 9.57, df = 6 (P = 0.14); I² = 37%
Test for overall effect: Z = 0.95 (P = 0.34)

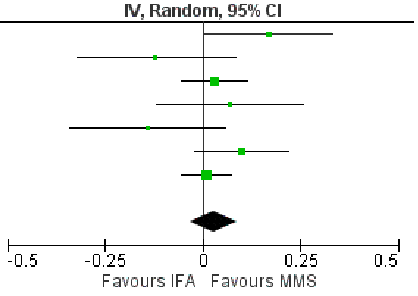

Footnotes
(1) Only the Usual Invitation to Food Supplementation groups were included. Both IFA arms were merged and compared with the MMS arm

Supplemental Figure 2.7.5 Effect of MMS vs IFA on WLZ at 18 months

Fixed

	MMS			IFA				Mean Difference
Study or Subgroup	Mean	SD	Total	Mean	SD	Total	Weight	IV, Fixed, 95% CI
Adu-Afarwuah 2015	-0.54	1	355	-0.64	1.02	337	26.7%	0.10 [-0.05, 0.25]
Ashorn 2010 complete FU	-0.19	1	222	-0.08	1	220	17.4%	-0.11 [-0.30, 0.08]
Ashorn 2010 simplified FU	-0.17	1.04	129	-0.19	1.07	128	9.1%	0.02 [-0.24, 0.28]
Fawzi 2007	-0.38	1.22	39	-0.39	1.37	55	2.2%	0.01 [-0.52, 0.54]
Tofail 2008 (1)	-0.83	1.08	463	-0.94	1	974	44.5%	0.11 [-0.01, 0.23]
**Total (95% CI)**			**1208**			**1714**	**100.0%**	**0.06 [-0.02, 0.14]**

Heterogeneity: Chi² = 4.30, df = 4 (P = 0.37); I² = 7%
Test for overall effect: Z = 1.47 (P = 0.14)

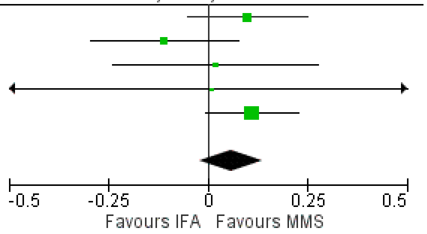

Footnotes
(1) Only the Usual Invitation to Food Supplementation groups were included. Both IFA arms were merged and compared with the MMS arm

Random

	MMS			IFA				Mean Difference
Study or Subgroup	Mean	SD	Total	Mean	SD	Total	Weight	IV, Random, 95% CI
Adu-Afarwuah 2015	-0.54	1	355	-0.64	1.02	337	27.1%	0.10 [-0.05, 0.25]
Ashorn 2010 complete FU	-0.19	1	222	-0.08	1	220	18.3%	-0.11 [-0.30, 0.08]
Ashorn 2010 simplified FU	-0.17	1.04	129	-0.19	1.07	128	9.9%	0.02 [-0.24, 0.28]
Fawzi 2007	-0.38	1.22	39	-0.39	1.37	55	2.4%	0.01 [-0.52, 0.54]
Tofail 2008 (1)	-0.83	1.08	463	-0.94	1	974	42.2%	0.11 [-0.01, 0.23]
**Total (95% CI)**			**1208**			**1714**	**100.0%**	**0.06 [-0.03, 0.14]**

Heterogeneity: Tau² = 0.00; Chi² = 4.30, df = 4 (P = 0.37); I² = 7%
Test for overall effect: Z = 1.32 (P = 0.19)

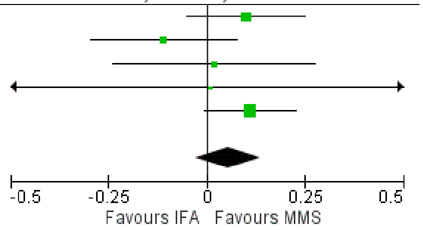

Footnotes
(1) Only the Usual Invitation to Food Supplementation groups were included. Both IFA arms were merged and compared with the MMS arm

Supplemental Figure 2.7.6 Effect of MMS vs IFA on WLZ at 24 months

Fixed

	MMS			IFA				Mean Difference
**Study or Subgroup**	**Mean**	**SD**	**Total**	**Mean**	**SD**	**Total**	**Weight**	**IV, Fixed, 95% CI**
Ashorn 2010 complete FU	-0.3	0.96	176	-0.17	0.93	196	4.5%	-0.13 [-0.32, 0.06]
Bliznashka 2021	-0.64	1.8786	642	-0.59	1.8786	718	4.2%	-0.05 [-0.25, 0.15]
Roberfroid 2008	-2.27	1.79	54	-2.4	2.22	55	0.3%	0.13 [-0.63, 0.89]
Tofail 2008 (1)	-0.83	0.97	479	-0.88	0.99	967	14.6%	0.05 [-0.06, 0.16]
West 2014	-1.29	0.96	3285	-1.29	0.97	3238	76.4%	0.00 [-0.05, 0.05]
**Total (95% CI)**			**4636**			**5174**	**100.0%**	**-0.00 [-0.04, 0.04]**

Heterogeneity: Chi² = 2.94, df = 4 (P = 0.57); I² = 0%
Test for overall effect: Z = 0.01 (P = 0.99)

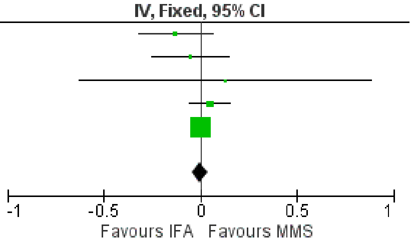

Footnotes
(1) Only the Usual Invitation to Food Supplementation groups were included. Both IFA arms were merged and compared with the MMS arm

Random

	MMS			IFA				Mean Difference
**Study or Subgroup**	**Mean**	**SD**	**Total**	**Mean**	**SD**	**Total**	**Weight**	**IV, Random, 95% CI**
Ashorn 2010 complete FU	-0.3	0.96	176	-0.17	0.93	196	4.5%	-0.13 [-0.32, 0.06]
Bliznashka 2021	-0.64	1.8786	642	-0.59	1.8786	718	4.2%	-0.05 [-0.25, 0.15]
Roberfroid 2008	-2.27	1.79	54	-2.4	2.22	55	0.3%	0.13 [-0.63, 0.89]
Tofail 2008 (1)	-0.83	0.97	479	-0.88	0.99	967	14.6%	0.05 [-0.06, 0.16]
West 2014	-1.29	0.96	3285	-1.29	0.97	3238	76.4%	0.00 [-0.05, 0.05]
**Total (95% CI)**			**4636**			**5174**	**100.0%**	**-0.00 [-0.04, 0.04]**

Heterogeneity: Tau² = 0.00; Chi² = 2.94, df = 4 (P = 0.57); I² = 0%
Test for overall effect: Z = 0.01 (P = 0.99)

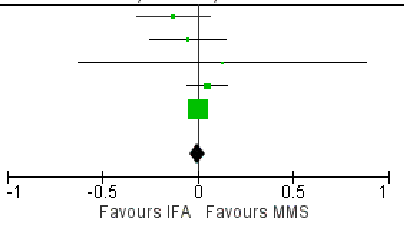

Footnotes
(1) Only the Usual Invitation to Food Supplementation groups were included. Both IFA arms were merged and compared with the MMS arm

Supplemental Figure 2.8. Effect of MMS vs IFA on HCAZ

Supplemental Figure 2.8.1. Effect of MMS vs IFA on HCAZ at birth

Fixed

	MMS			IFA				Mean Difference
Study or Subgroup	Mean	SD	Total	Mean	SD	Total	Weight	IV, Fixed, 95% CI
Adu-Afarwuah 2015	-0.41	1.16	317	-0.45	1.08	304	3.4%	0.04 [-0.14, 0.22]
Ashorn 2010	-0.14	1.11	372	-0.24	1.12	360	4.0%	0.10 [-0.06, 0.26]
Fawzi 2007	0.17	2.15	3332	0.11	2.29	3339	9.2%	0.06 [-0.05, 0.17]
Roberfroid 2008	-0.36	1.23	534	-0.41	1.14	539	5.2%	0.05 [-0.09, 0.19]
Sunawang 2009	-0.74	1.08	630	-0.76	1.09	648	7.4%	0.02 [-0.10, 0.14]
Tofail 2008 (1)	-1.34	3.11	490	-1.59	1.42	1012	1.3%	0.25 [-0.04, 0.54]
West 2014	-1.25	1.16	7062	-1.4	1.18	6963	69.6%	0.15 [0.11, 0.19]
**Total (95% CI)**			**12737**			**13165**	**100.0%**	**0.12 [0.09, 0.15]**

Heterogeneity: Chi² = 8.78, df = 6 (P = 0.19); I² = 32%
Test for overall effect: Z = 7.43 (P < 0.00001)

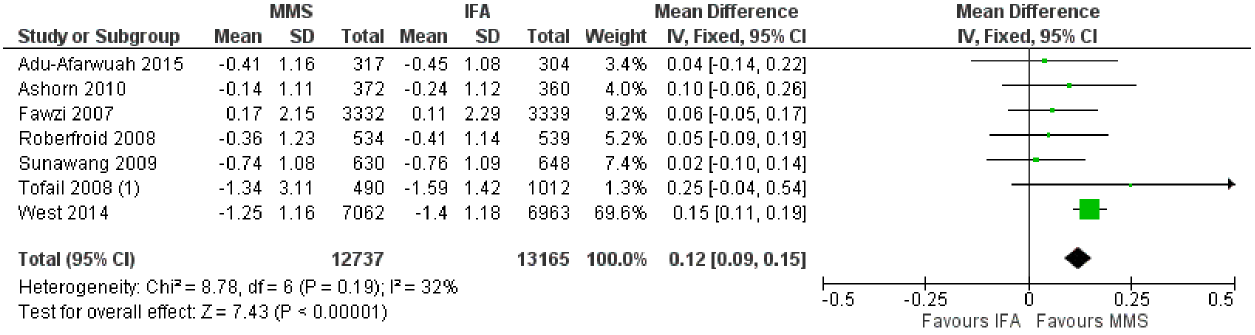

Footnotes
(1) Only the Usual Invitation to Food Supplementation groups were included. Both IFA arms were merged and compared with the MMS arm

Random

	MMS			IFA				Mean Difference
Study or Subgroup	Mean	SD	Total	Mean	SD	Total	Weight	IV, Random, 95% CI
Adu-Afarwuah 2015	-0.41	1.16	317	-0.45	1.08	304	7.7%	0.04 [-0.14, 0.22]
Ashorn 2010	-0.14	1.11	372	-0.24	1.12	360	8.9%	0.10 [-0.06, 0.26]
Fawzi 2007	0.17	2.15	3332	0.11	2.29	3339	16.5%	0.06 [-0.05, 0.17]
Roberfroid 2008	-0.36	1.23	534	-0.41	1.14	539	10.9%	0.05 [-0.09, 0.19]
Sunawang 2009	-0.74	1.08	630	-0.76	1.09	648	14.2%	0.02 [-0.10, 0.14]
Tofail 2008 (1)	-1.34	3.11	490	-1.59	1.42	1012	3.2%	0.25 [-0.04, 0.54]
West 2014	-1.25	1.16	7062	-1.4	1.18	6963	38.6%	0.15 [0.11, 0.19]
**Total (95% CI)**			**12737**			**13165**	**100.0%**	**0.10 [0.04, 0.15]**

Heterogeneity: Tau² = 0.00; Chi² = 8.78, df = 6 (P = 0.19); I² = 32%
Test for overall effect: Z = 3.53 (P = 0.0004)

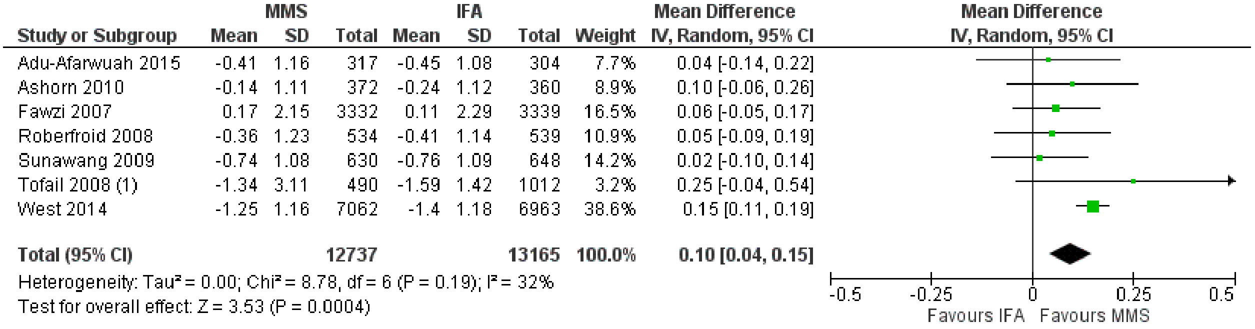

Footnotes
(1) Only the Usual Invitation to Food Supplementation groups were included. Both IFA arms were merged and compared with the MMS arm

Supplemental Figure 2.8.2 Effect of MMS vs IFA on HCAZ at 3 months

Fixed

Study or Subgroup	MMS Mean	MMS SD	MMS Total	IFA Mean	IFA SD	IFA Total	Weight	Mean Difference IV, Fixed, 95% CI
Adu-Afarwuah 2015	-0.63	0.93	358	-0.82	0.95	343	3.6%	0.19 [0.05, 0.33]
Fawzi 2007	0.47	1.25	2969	0.46	1.24	2986	17.5%	0.01 [-0.05, 0.07]
Moore 2009	-0.401	0.972	181	-0.457	0.981	171	1.7%	0.06 [-0.15, 0.26]
Roberfroid 2008	-0.19	1.04	306	-0.3	1.03	349	2.8%	0.11 [-0.05, 0.27]
Tofail 2008 (1)	-1.35	1.19	376	-1.35	1.13	787	3.4%	0.00 [-0.14, 0.14]
West 2014	-1.01	1	8055	-1.13	1.02	7839	71.0%	0.12 [0.09, 0.15]
**Total (95% CI)**			**12245**			**12475**	**100.0%**	**0.10 [0.07, 0.12]**

Heterogeneity: Chi² = 12.97, df = 5 (P = 0.02); I² = 61%
Test for overall effect: Z = 7.24 (P < 0.00001)

Mean Difference
IV, Fixed, 95% CI

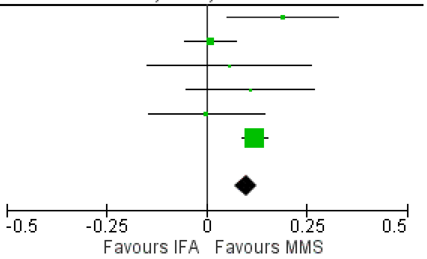

Footnotes
(1) Only the Usual Invitation to Food Supplementation groups were included. Both IFA arms were merged and compared with the MMS arm

Random

Study or Subgroup	MMS Mean	MMS SD	MMS Total	IFA Mean	IFA SD	IFA Total	Weight	Mean Difference IV, Random, 95% CI
Adu-Afarwuah 2015	-0.63	0.93	358	-0.82	0.95	343	12.9%	0.19 [0.05, 0.33]
Fawzi 2007	0.47	1.25	2969	0.46	1.24	2986	25.2%	0.01 [-0.05, 0.07]
Moore 2009	-0.401	0.972	181	-0.457	0.981	171	7.6%	0.06 [-0.15, 0.26]
Roberfroid 2008	-0.19	1.04	306	-0.3	1.03	349	10.9%	0.11 [-0.05, 0.27]
Tofail 2008 (1)	-1.35	1.19	376	-1.35	1.13	787	12.4%	0.00 [-0.14, 0.14]
West 2014	-1.01	1	8055	-1.13	1.02	7839	31.0%	0.12 [0.09, 0.15]
**Total (95% CI)**			**12245**			**12475**	**100.0%**	**0.08 [0.02, 0.14]**

Heterogeneity: Tau² = 0.00; Chi² = 12.97, df = 5 (P = 0.02); I² = 61%
Test for overall effect: Z = 2.47 (P = 0.01)

Mean Difference
IV, Random, 95% CI

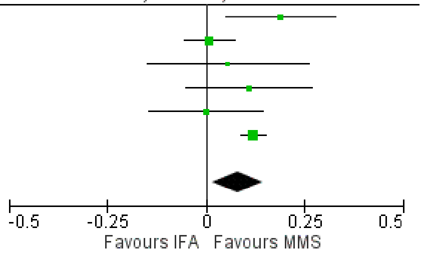

Footnotes
(1) Only the Usual Invitation to Food Supplementation groups were included. Both IFA arms were merged and compared with the MMS arm

Supplemental Figure 2.8.3 Effect of MMS vs IFA on HCAZ at 6 months

Fixed

	MMS			IFA				Mean Difference
Study or Subgroup	Mean	SD	Total	Mean	SD	Total	Weight	IV, Fixed, 95% CI
Adu-Afarwuah 2015	-0.75	0.9	356	-0.87	0.96	347	4.6%	0.12 [-0.02, 0.26]
Ashorn 2010 complete FU	-0.22	1.07	216	-0.24	1.1	218	2.1%	0.02 [-0.18, 0.22]
Ashorn 2010 simplified FU	-0.56	1.04	131	-0.53	1.02	130	1.4%	-0.03 [-0.28, 0.22]
Fawzi 2007	0.18	1.15	3026	0.19	1.1	2996	27.0%	-0.01 [-0.07, 0.05]
Moore 2009	-0.501	0.972	170	-0.663	0.987	172	2.0%	0.16 [-0.05, 0.37]
Roberfroid 2008	-0.42	1.08	270	-0.48	0.99	313	3.1%	0.06 [-0.11, 0.23]
Tofail 2008 (1)	-1.49	1.09	449	-1.51	1.1	892	5.7%	0.02 [-0.10, 0.14]
West 2014	-1.12	0.98	4559	-1.18	0.97	4489	54.1%	0.06 [0.02, 0.10]
**Total (95% CI)**			**9177**			**9557**	**100.0%**	**0.04 [0.01, 0.07]**

Heterogeneity: Chi² = 7.03, df = 7 (P = 0.43); I² = 0%
Test for overall effect: Z = 2.75 (P = 0.006)

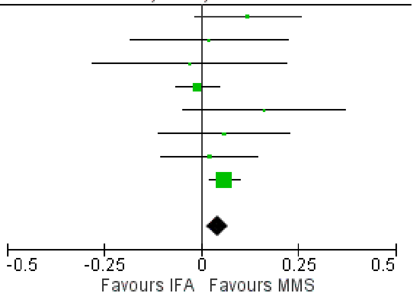

Footnotes
(1) Only the Usual Invitation to Food Supplementation groups were included. Both IFA arms were merged and compared with the MMS arm

Random

	MMS			IFA				Mean Difference
Study or Subgroup	Mean	SD	Total	Mean	SD	Total	Weight	IV, Random, 95% CI
Adu-Afarwuah 2015	-0.75	0.9	356	-0.87	0.96	347	4.7%	0.12 [-0.02, 0.26]
Ashorn 2010 complete FU	-0.22	1.07	216	-0.24	1.1	218	2.1%	0.02 [-0.18, 0.22]
Ashorn 2010 simplified FU	-0.56	1.04	131	-0.53	1.02	130	1.4%	-0.03 [-0.28, 0.22]
Fawzi 2007	0.18	1.15	3026	0.19	1.1	2996	27.2%	-0.01 [-0.07, 0.05]
Moore 2009	-0.501	0.972	170	-0.663	0.987	172	2.1%	0.16 [-0.05, 0.37]
Roberfroid 2008	-0.42	1.08	270	-0.48	0.99	313	3.1%	0.06 [-0.11, 0.23]
Tofail 2008 (1)	-1.49	1.09	449	-1.51	1.1	892	5.8%	0.02 [-0.10, 0.14]
West 2014	-1.12	0.98	4559	-1.18	0.97	4489	53.7%	0.06 [0.02, 0.10]
**Total (95% CI)**			**9177**			**9557**	**100.0%**	**0.04 [0.01, 0.07]**

Heterogeneity: Tau² = 0.00; Chi² = 7.03, df = 7 (P = 0.43); I² = 0%
Test for overall effect: Z = 2.72 (P = 0.006)

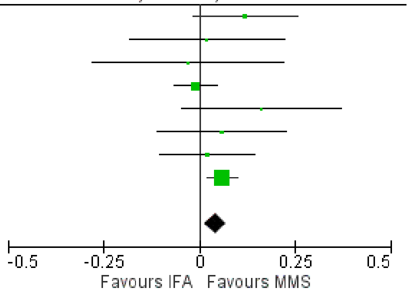

Footnotes
(1) Only the Usual Invitation to Food Supplementation groups were included. Both IFA arms were merged and compared with the MMS arm

Supplemental Figure 2.8.4 Effect of MMS vs IFA on HCAZ at 12 months

Fixed

	MMS			IFA				Mean Difference
Study or Subgroup	Mean	SD	Total	Mean	SD	Total	Weight	IV, Fixed, 95% CI
Adu-Afarwuah 2015	-1.01	0.86	346	-1.08	0.98	332	4.9%	0.07 [-0.07, 0.21]
Ashorn 2010 complete FU	-0.68	1.09	215	-0.65	0.97	219	2.5%	-0.03 [-0.22, 0.16]
Fawzi 2007	0.01	1.07	1620	-0.02	1.06	1600	17.3%	0.03 [-0.04, 0.10]
Roberfroid 2008	-0.83	1.19	240	-0.95	0.96	245	2.5%	0.12 [-0.07, 0.31]
Tofail 2008 (1)	-1.81	1.08	506	-1.81	1.08	1033	7.1%	0.00 [-0.11, 0.11]
West 2014	-1.44	0.96	4945	-1.46	0.95	4862	65.7%	0.02 [-0.02, 0.06]
**Total (95% CI)**			**7872**			**8291**	**100.0%**	**0.02 [-0.01, 0.05]**

Heterogeneity: Chi² = 1.91, df = 5 (P = 0.86); I² = 0%
Test for overall effect: Z = 1.54 (P = 0.12)

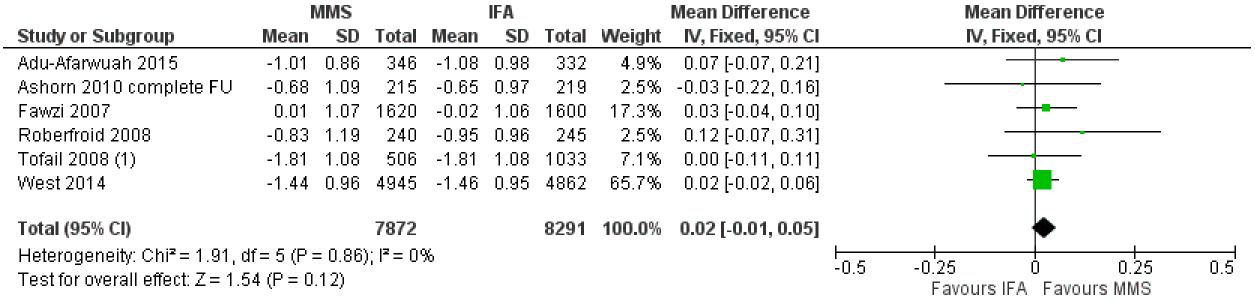

Footnotes
(1) Only the Usual Invitation to Food Supplementation groups were included. Both IFA arms were merged and compared with the MMS arm

Random

	MMS			IFA				Mean Difference
Study or Subgroup	Mean	SD	Total	Mean	SD	Total	Weight	IV, Random, 95% CI
Adu-Afarwuah 2015	-1.01	0.86	346	-1.08	0.98	332	4.9%	0.07 [-0.07, 0.21]
Ashorn 2010 complete FU	-0.68	1.09	215	-0.65	0.97	219	2.5%	-0.03 [-0.22, 0.16]
Fawzi 2007	0.01	1.07	1620	-0.02	1.06	1600	17.3%	0.03 [-0.04, 0.10]
Roberfroid 2008	-0.83	1.19	240	-0.95	0.96	245	2.5%	0.12 [-0.07, 0.31]
Tofail 2008 (1)	-1.81	1.08	506	-1.81	1.08	1033	7.1%	0.00 [-0.11, 0.11]
West 2014	-1.44	0.96	4945	-1.46	0.95	4862	65.7%	0.02 [-0.02, 0.06]
**Total (95% CI)**			**7872**			**8291**	**100.0%**	**0.02 [-0.01, 0.05]**

Heterogeneity: Tau² = 0.00; Chi² = 1.91, df = 5 (P = 0.86); I² = 0%
Test for overall effect: Z = 1.54 (P = 0.12)

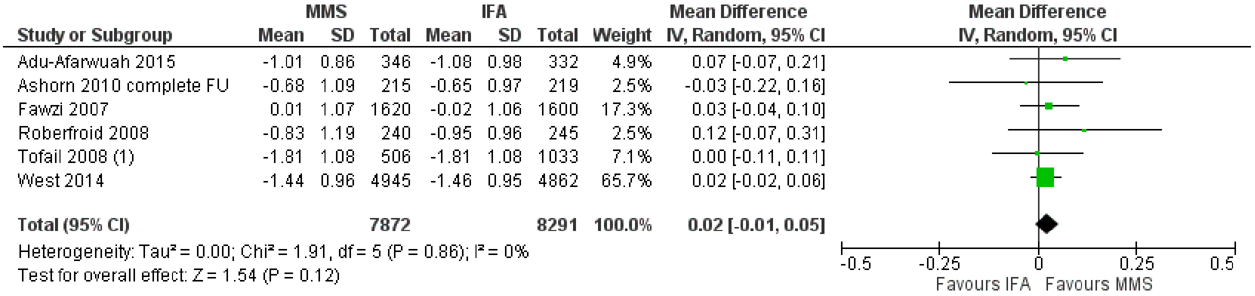

Footnotes
(1) Only the Usual Invitation to Food Supplementation groups were included. Both IFA arms were merged and compared with the MMS arm

Supplemental Figure 2.8.5 Effect of MMS vs IFA on HCAZ at 18 months

Fixed

	MMS			IFA				Mean Difference
**Study or Subgroup**	**Mean**	**SD**	**Total**	**Mean**	**SD**	**Total**	**Weight**	**IV, Fixed, 95% CI**
Adu-Afarwuah 2015	-1.1	0.86	355	-1.18	0.94	337	51.6%	0.08 [-0.05, 0.21]
Ashorn 2010 complete FU	-0.78	1	222	-0.76	1.02	220	26.3%	-0.02 [-0.21, 0.17]
Ashorn 2010 simplified FU	-0.85	0.89	130	-0.86	1	130	17.6%	0.01 [-0.22, 0.24]
Fawzi 2007	0.01	1.04	39	-0.04	1.2	55	4.5%	0.05 [-0.41, 0.51]
**Total (95% CI)**			**746**			**742**	**100.0%**	**0.04 [-0.06, 0.14]**

Heterogeneity: Chi² = 0.80, df = 3 (P = 0.85); I² = 0%
Test for overall effect: Z = 0.81 (P = 0.42)

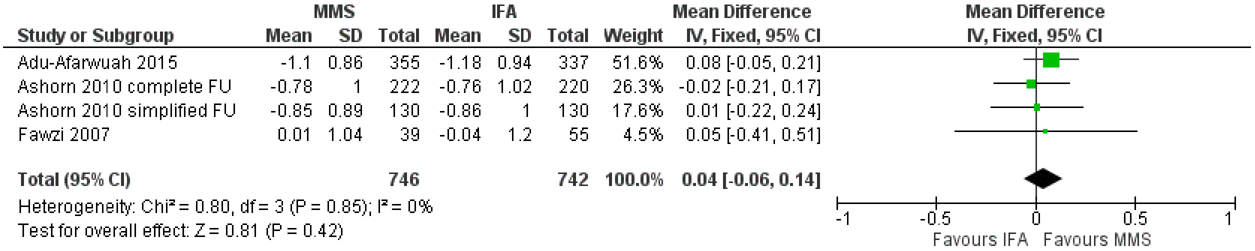

Random

	MMS			IFA				Mean Difference
**Study or Subgroup**	**Mean**	**SD**	**Total**	**Mean**	**SD**	**Total**	**Weight**	**IV, Random, 95% CI**
Adu-Afarwuah 2015	-1.1	0.86	355	-1.18	0.94	337	51.6%	0.08 [-0.05, 0.21]
Ashorn 2010 complete FU	-0.78	1	222	-0.76	1.02	220	26.3%	-0.02 [-0.21, 0.17]
Ashorn 2010 simplified FU	-0.85	0.89	130	-0.86	1	130	17.6%	0.01 [-0.22, 0.24]
Fawzi 2007	0.01	1.04	39	-0.04	1.2	55	4.5%	0.05 [-0.41, 0.51]
**Total (95% CI)**			**746**			**742**	**100.0%**	**0.04 [-0.06, 0.14]**

Heterogeneity: Tau² = 0.00; Chi² = 0.80, df = 3 (P = 0.85); I² = 0%
Test for overall effect: Z = 0.81 (P = 0.42)

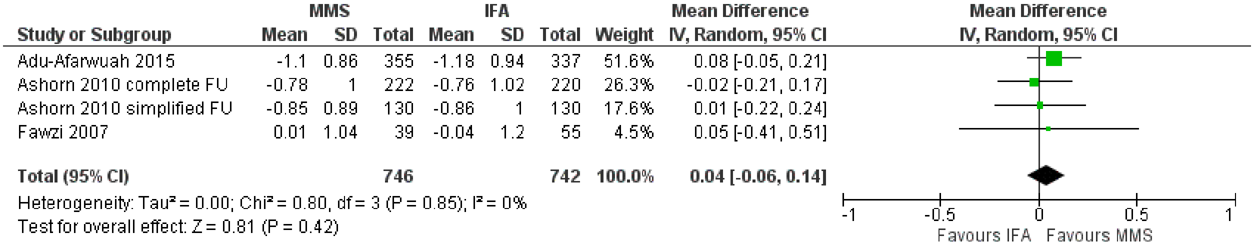

Supplemental Figure 2.8.6 Effect of MMS vs IFA on HCAZ at 24 months

Fixed

Study or Subgroup	MMS Mean	MMS SD	MMS Total	IFA Mean	IFA SD	IFA Total	Weight	Mean Difference IV, Fixed, 95% CI
Ashorn 2010 complete FU	-0.99	1.1	179	-0.85	0.99	197	4.3%	-0.14 [-0.35, 0.07]
West 2014	-1.63	0.94	3405	-1.64	0.94	3355	95.7%	0.01 [-0.03, 0.05]
**Total (95% CI)**			**3584**			**3552**	**100.0%**	**0.00 [-0.04, 0.05]**

Heterogeneity: Chi² = 1.84, df = 1 (P = 0.18); I² = 46%
Test for overall effect: Z = 0.16 (P = 0.87)

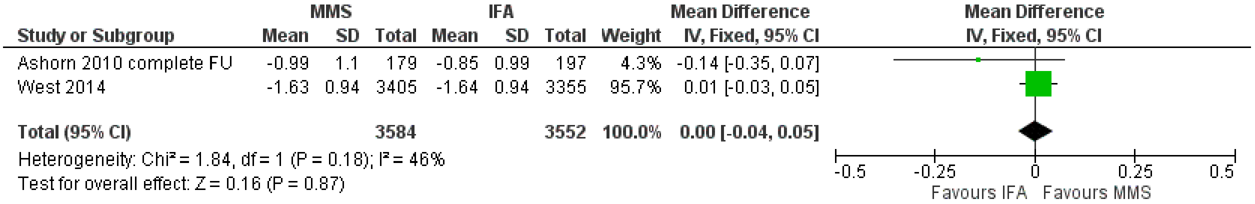

Random

Study or Subgroup	MMS Mean	MMS SD	MMS Total	IFA Mean	IFA SD	IFA Total	Weight	Mean Difference IV, Random, 95% CI
Ashorn 2010 complete FU	-0.99	1.1	179	-0.85	0.99	197	25.1%	-0.14 [-0.35, 0.07]
West 2014	-1.63	0.94	3405	-1.64	0.94	3355	74.9%	0.01 [-0.03, 0.05]
**Total (95% CI)**			**3584**			**3552**	**100.0%**	**-0.03 [-0.16, 0.10]**

Heterogeneity: Tau² = 0.01; Chi² = 1.84, df = 1 (P = 0.18); I² = 46%
Test for overall effect: Z = 0.42 (P = 0.67)

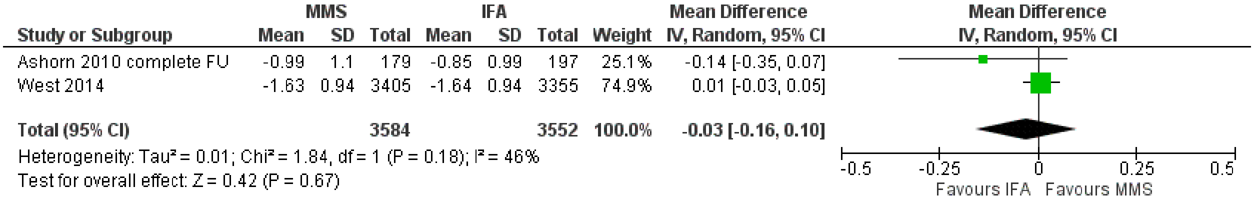

Supplemental Figure 2.9. Effect of MMS vs IFA on MUACZ

Supplemental Figure 2.9.1 Effect of MMS vs IFA on MUACZ at 3 months

Fixed

	MMS			IFA				Mean Difference
Study or Subgroup	Mean	SD	Total	Mean	SD	Total	Weight	IV, Fixed, 95% CI
Adu-Afarwuah 2015	0.12	1.01	340	-0.05	1.09	318	2.7%	0.17 [0.01, 0.33]
Fawzi 2007	0.15	1.05	1431	0.16	1.05	1456	12.0%	-0.01 [-0.09, 0.07]
Moore 2009	0.109	0.867	150	0.063	0.895	154	1.8%	0.05 [-0.15, 0.24]
Roberfroid 2008	0.07	0.76	306	0.06	0.74	349	5.3%	0.01 [-0.11, 0.13]
Sunawang 2009	-0.07	0.85	151	-0.11	0.9	148	1.8%	0.04 [-0.16, 0.24]
West 2014	-0.76	0.96	8072	-0.85	0.99	7858	76.5%	0.09 [0.06, 0.12]
**Total (95% CI)**			**10450**			**10283**	**100.0%**	**0.07 [0.05, 0.10]**

Heterogeneity: Chi² = 8.43, df = 5 (P = 0.13); I² = 41%
Test for overall effect: Z = 5.50 (P < 0.00001)

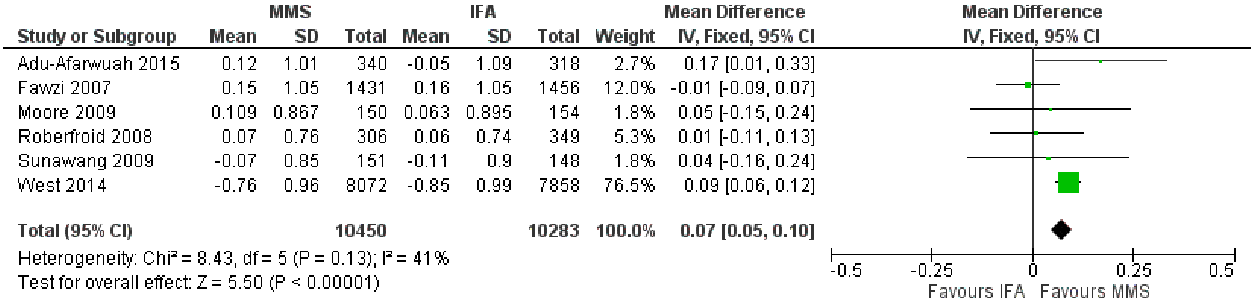

Random

	MMS			IFA				Mean Difference
Study or Subgroup	Mean	SD	Total	Mean	SD	Total	Weight	IV, Random, 95% CI
Adu-Afarwuah 2015	0.12	1.01	340	-0.05	1.09	318	8.8%	0.17 [0.01, 0.33]
Fawzi 2007	0.15	1.05	1431	0.16	1.05	1456	23.7%	-0.01 [-0.09, 0.07]
Moore 2009	0.109	0.867	150	0.063	0.895	154	6.2%	0.05 [-0.15, 0.24]
Roberfroid 2008	0.07	0.76	306	0.06	0.74	349	14.6%	0.01 [-0.11, 0.13]
Sunawang 2009	-0.07	0.85	151	-0.11	0.9	148	6.2%	0.04 [-0.16, 0.24]
West 2014	-0.76	0.96	8072	-0.85	0.99	7858	40.4%	0.09 [0.06, 0.12]
**Total (95% CI)**			**10450**			**10283**	**100.0%**	**0.06 [0.00, 0.11]**

Heterogeneity: Tau² = 0.00; Chi² = 8.43, df = 5 (P = 0.13); I² = 41%
Test for overall effect: Z = 2.06 (P = 0.04)

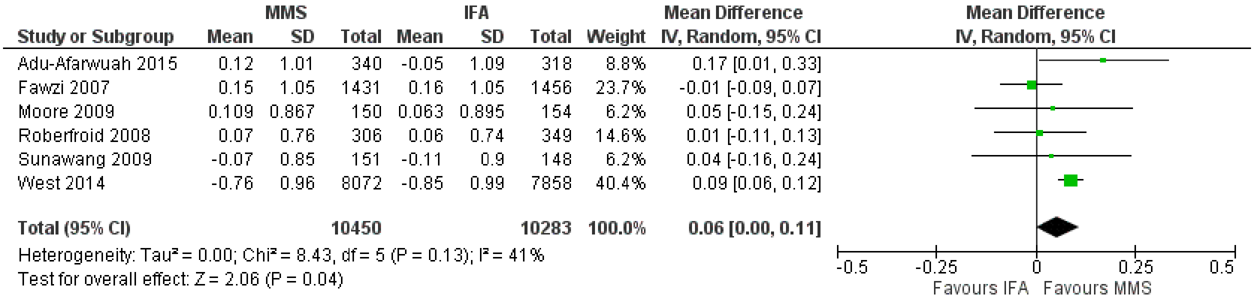

Supplemental Figure 2.9.2 Effect of MMS vs IFA on MUACZ at 6 months

Fixed

	MMS			IFA				Mean Difference
**Study or Subgroup**	**Mean**	**SD**	**Total**	**Mean**	**SD**	**Total**	**Weight**	**IV, Fixed, 95% CI**
Adu-Afarwuah 2015	-0.12	1	356	-0.21	1.01	348	4.3%	0.09 [-0.06, 0.24]
Ashorn 2010 complete FU	0.13	1.25	216	0	1.21	220	1.8%	0.13 [-0.10, 0.36]
Ashorn 2010 simplified FU	0.21	1.12	131	0.24	1.06	130	1.4%	-0.03 [-0.29, 0.23]
Fawzi 2007	0.01	1.12	1852	0.02	1.09	1842	18.8%	-0.01 [-0.08, 0.06]
Moore 2009	-0.055	0.87	170	-0.131	0.992	172	2.4%	0.08 [-0.12, 0.27]
Roberfroid 2008	-0.13	1.05	270	-0.28	1.06	313	3.2%	0.15 [-0.02, 0.32]
Sunawang 2009	-0.18	0.9	151	-0.12	0.89	148	2.3%	-0.06 [-0.26, 0.14]
West 2014	-0.72	0.92	4564	-0.74	0.93	4499	65.7%	0.02 [-0.02, 0.06]
**Total (95% CI)**			**7710**			**7672**	**100.0%**	**0.02 [-0.01, 0.05]**

Heterogeneity: Chi² = 5.63, df = 7 (P = 0.58); I² = 0%

Test for overall effect: Z = 1.42 (P = 0.16)

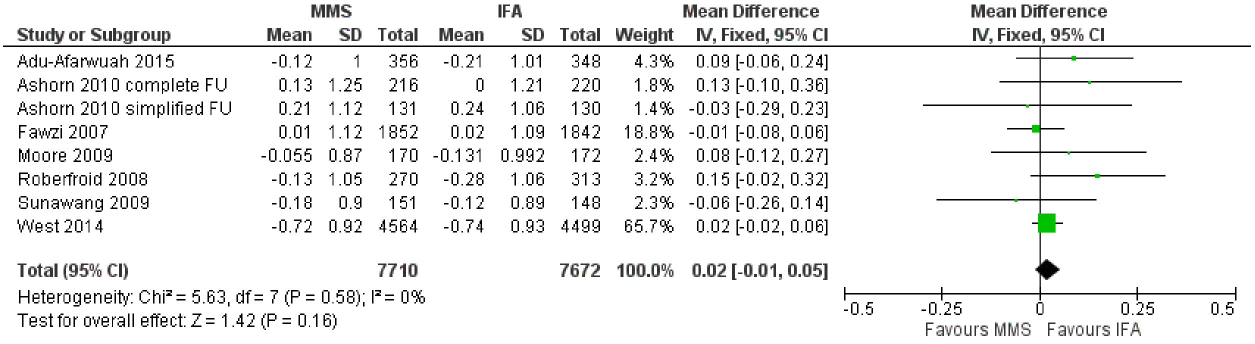

Random

	MMS			IFA				Mean Difference
**Study or Subgroup**	**Mean**	**SD**	**Total**	**Mean**	**SD**	**Total**	**Weight**	**IV, Random, 95% CI**
Adu-Afarwuah 2015	-0.12	1	356	-0.21	1.01	348	4.3%	0.09 [-0.06, 0.24]
Ashorn 2010 complete FU	0.13	1.25	216	0	1.21	220	1.8%	0.13 [-0.10, 0.36]
Ashorn 2010 simplified FU	0.21	1.12	131	0.24	1.06	130	1.4%	-0.03 [-0.29, 0.23]
Fawzi 2007	0.01	1.12	1852	0.02	1.09	1842	18.8%	-0.01 [-0.08, 0.06]
Moore 2009	-0.055	0.87	170	-0.131	0.992	172	2.4%	0.08 [-0.12, 0.27]
Roberfroid 2008	-0.13	1.05	270	-0.28	1.06	313	3.2%	0.15 [-0.02, 0.32]
Sunawang 2009	-0.18	0.9	151	-0.12	0.89	148	2.3%	-0.06 [-0.26, 0.14]
West 2014	-0.72	0.92	4564	-0.74	0.93	4499	65.7%	0.02 [-0.02, 0.06]
**Total (95% CI)**			**7710**			**7672**	**100.0%**	**0.02 [-0.01, 0.05]**

Heterogeneity: Tau² = 0.00; Chi² = 5.63, df = 7 (P = 0.58); I² = 0%

Test for overall effect: Z = 1.42 (P = 0.16)

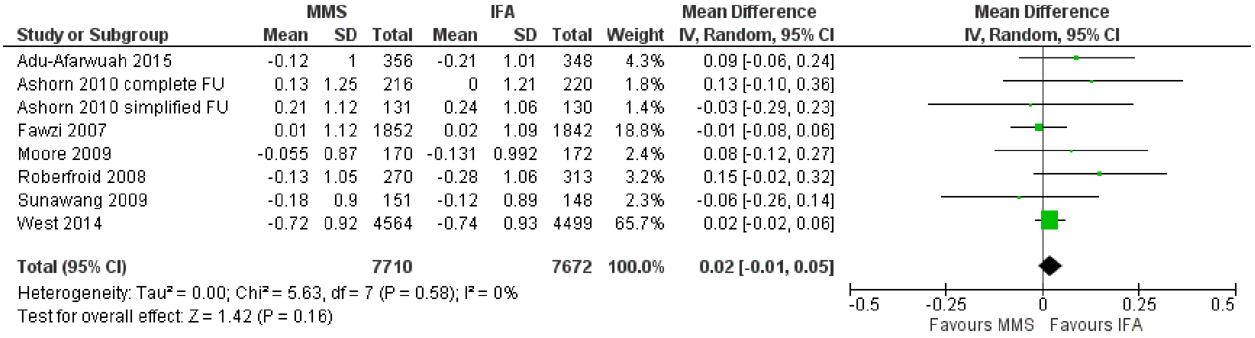

Supplemental Figure 2.9.3 Effect of MMS vs IFA on MUACZ at 12 months

Fixed

	MMS			IFA				Mean Difference
**Study or Subgroup**	**Mean**	**SD**	**Total**	**Mean**	**SD**	**Total**	**Weight**	**IV, Fixed, 95% CI**
Adu-Afarwuah 2015	-0.19	0.92	346	-0.35	0.94	334	4.9%	0.16 [0.02, 0.30]
Ashorn 2010 complete FU	0.03	1.15	215	0.1	0.92	219	2.5%	-0.07 [-0.27, 0.13]
Fawzi 2007	0.02	1.13	1490	0	1.1	1483	14.9%	0.02 [-0.06, 0.10]
Roberfroid 2008	-0.64	1.15	240	-0.68	1.03	248	2.5%	0.04 [-0.15, 0.23]
Sunawang 2009	-0.79	1.01	150	-0.83	1.09	148	1.7%	0.04 [-0.20, 0.28]
West 2014	-1.02	0.91	4998	-1.03	0.92	4913	73.6%	0.01 [-0.03, 0.05]
**Total (95% CI)**			**7439**			**7345**	**100.0%**	**0.02 [-0.01, 0.05]**

Heterogeneity: Chi² = 5.01, df = 5 (P = 0.42); I² = 0%
Test for overall effect: Z = 1.15 (P = 0.25)

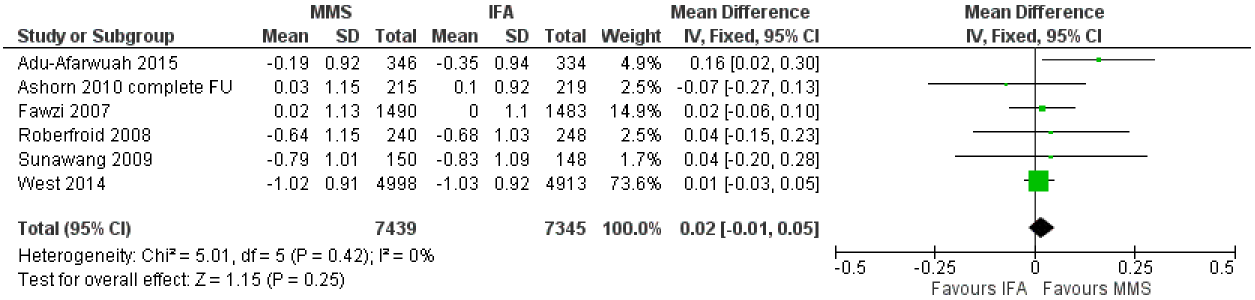

Random

	MMS			IFA				Mean Difference
**Study or Subgroup**	**Mean**	**SD**	**Total**	**Mean**	**SD**	**Total**	**Weight**	**IV, Random, 95% CI**
Adu-Afarwuah 2015	-0.19	0.92	346	-0.35	0.94	334	4.9%	0.16 [0.02, 0.30]
Ashorn 2010 complete FU	0.03	1.15	215	0.1	0.92	219	2.5%	-0.07 [-0.27, 0.13]
Fawzi 2007	0.02	1.13	1490	0	1.1	1483	15.0%	0.02 [-0.06, 0.10]
Roberfroid 2008	-0.64	1.15	240	-0.68	1.03	248	2.6%	0.04 [-0.15, 0.23]
Sunawang 2009	-0.79	1.01	150	-0.83	1.09	148	1.7%	0.04 [-0.20, 0.28]
West 2014	-1.02	0.91	4998	-1.03	0.92	4913	73.4%	0.01 [-0.03, 0.05]
**Total (95% CI)**			**7439**			**7345**	**100.0%**	**0.02 [-0.01, 0.05]**

Heterogeneity: Tau² = 0.00; Chi² = 5.01, df = 5 (P = 0.42); I² = 0%
Test for overall effect: Z = 1.15 (P = 0.25)

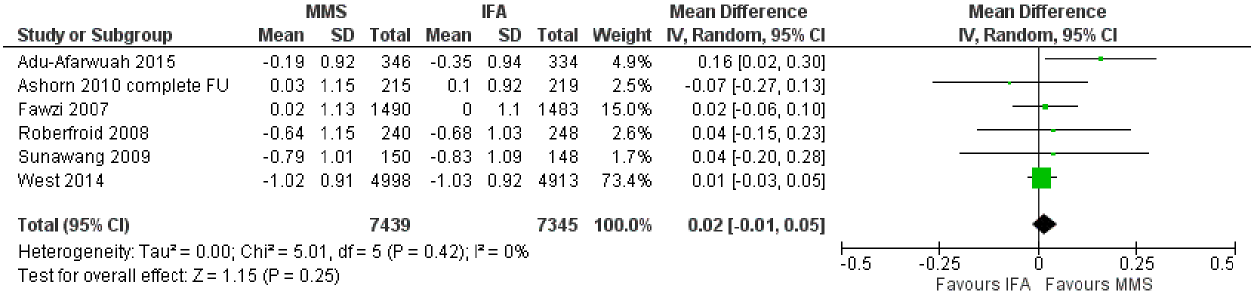

Supplemental Figure 2.9.4 Effect of MMS vs IFA on MUACZ at 18 months

Fixed

Study or Subgroup	MMS Mean	MMS SD	MMS Total	IFA Mean	IFA SD	IFA Total	Weight	Mean Difference IV, Fixed, 95% CI
Adu-Afarwuah 2015	-0.42	0.9	356	-0.54	0.94	340	51.1%	0.12 [-0.02, 0.26]
Ashorn 2010 complete FU	0.06	1.11	222	0.08	0.88	220	27.5%	-0.02 [-0.21, 0.17]
Ashorn 2010 simplified FU	0.07	0.98	130	-0.02	0.99	130	16.7%	0.09 [-0.15, 0.33]
Fawzi 2007	-0.09	0.97	39	0.04	1.24	53	4.7%	-0.13 [-0.58, 0.32]
**Total (95% CI)**			**747**			**743**	**100.0%**	**0.06 [-0.03, 0.16]**

Heterogeneity: Chi² = 2.17, df = 3 (P = 0.54); I² = 0%
Test for overall effect: Z = 1.30 (P = 0.19)

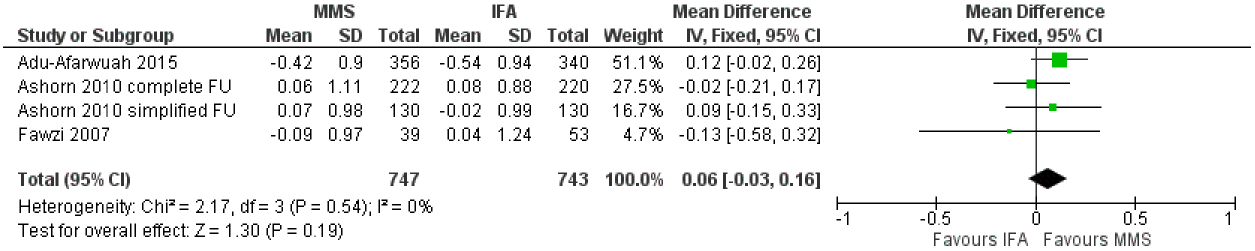

Random

Study or Subgroup	MMS Mean	MMS SD	MMS Total	IFA Mean	IFA SD	IFA Total	Weight	Mean Difference IV, Random, 95% CI
Adu-Afarwuah 2015	-0.42	0.9	356	-0.54	0.94	340	51.1%	0.12 [-0.02, 0.26]
Ashorn 2010 complete FU	0.06	1.11	222	0.08	0.88	220	27.5%	-0.02 [-0.21, 0.17]
Ashorn 2010 simplified FU	0.07	0.98	130	-0.02	0.99	130	16.7%	0.09 [-0.15, 0.33]
Fawzi 2007	-0.09	0.97	39	0.04	1.24	53	4.7%	-0.13 [-0.58, 0.32]
**Total (95% CI)**			**747**			**743**	**100.0%**	**0.06 [-0.03, 0.16]**

Heterogeneity: Tau² = 0.00; Chi² = 2.17, df = 3 (P = 0.54); I² = 0%
Test for overall effect: Z = 1.30 (P = 0.19)

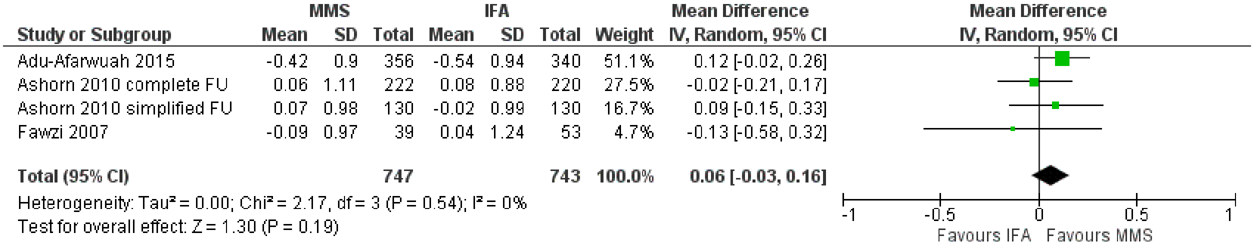

Supplemental Figure 2.9.5 Effect of MMS vs IFA on MUACZ at 24 months

Fixed

	MMS			IFA				Mean Difference
**Study or Subgroup**	**Mean**	**SD**	**Total**	**Mean**	**SD**	**Total**	**Weight**	**IV, Fixed, 95% CI**
Ashorn 2010 complete FU	-0.21	1.03	179	-0.03	0.9	197	4.1%	-0.18 [-0.38, 0.02]
Roberfroid 2008	-0.88	0.96	54	-0.92	1.19	55	1.0%	0.04 [-0.37, 0.45]
West 2014	-1.22	0.84	3433	-1.22	0.87	3381	95.0%	0.00 [-0.04, 0.04]
**Total (95% CI)**			**3666**			**3633**	**100.0%**	**-0.01 [-0.05, 0.03]**

Heterogeneity: Chi² = 3.15, df = 2 (P = 0.21); I² = 36%
Test for overall effect: Z = 0.34 (P = 0.73)

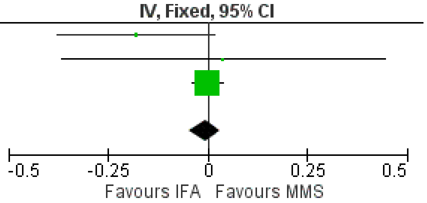

Random

	MMS			IFA				Mean Difference
**Study or Subgroup**	**Mean**	**SD**	**Total**	**Mean**	**SD**	**Total**	**Weight**	**IV, Random, 95% CI**
Ashorn 2010 complete FU	-0.21	1.03	179	-0.03	0.9	197	24.2%	-0.18 [-0.38, 0.02]
Roberfroid 2008	-0.88	0.96	54	-0.92	1.19	55	7.6%	0.04 [-0.37, 0.45]
West 2014	-1.22	0.84	3433	-1.22	0.87	3381	68.2%	0.00 [-0.04, 0.04]
**Total (95% CI)**			**3666**			**3633**	**100.0%**	**-0.04 [-0.16, 0.08]**

Heterogeneity: Tau² = 0.00; Chi² = 3.15, df = 2 (P = 0.21); I² = 36%
Test for overall effect: Z = 0.68 (P = 0.50)

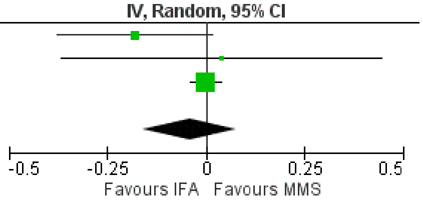

Supplemental Figure 2.10. Effect of MMS vs IFA on stunting

Supplemental Figure 2.10.1. Effect of MMS vs IFA on stunting at birth

Fixed

Study or Subgroup	log[Risk Ratio]	SE	MMS Total	IFA Total	Weight	Risk Ratio IV, Fixed, 95% CI
Adu-Afarwuah 2015	-0.241	0.2565	313	305	0.6%	0.79 [0.48, 1.30]
Ashorn 2010	-0.3157	0.1681	372	360	1.4%	0.73 [0.52, 1.01]
Fawzi 2007	-0.0653	0.0434	2799	2794	20.6%	0.94 [0.86, 1.02]
Osrin 2005	-0.0914	0.1824	526	517	1.2%	0.91 [0.64, 1.30]
Roberfroid 2008	-0.0989	0.1529	534	539	1.7%	0.91 [0.67, 1.22]
Sunawang 2009	0.071	0.1211	632	649	2.6%	1.07 [0.85, 1.36]
Tofail 2008 (1)	0.0591	0.1038	483	1006	3.6%	1.06 [0.87, 1.30]
West 2014	-0.1123	0.0238	6964	6861	68.4%	0.89 [0.85, 0.94]
**Total (95% CI)**			**12623**	**13031**	**100.0%**	**0.91 [0.88, 0.95]**

Heterogeneity: Chi² = 7.13, df = 7 (P = 0.42); I² = 2%
Test for overall effect: Z = 4.81 (P < 0.00001)

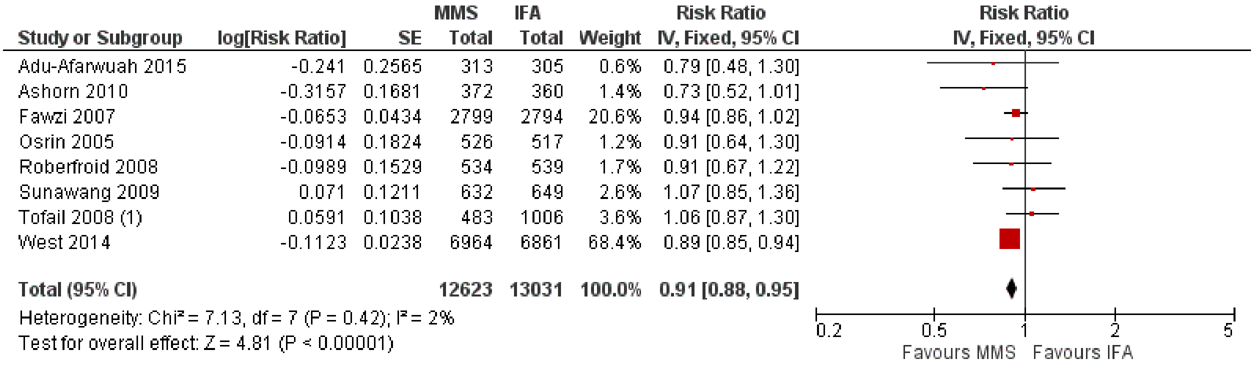

Footnotes
(1) Only the Usual Invitation to Food Supplementation groups were included. Both IFA arms were merged and compared with the MMS arm

Random

Study or Subgroup	log[Risk Ratio]	SE	MMS Total	IFA Total	Weight	Risk Ratio IV, Random, 95% CI
Adu-Afarwuah 2015	-0.241	0.2565	313	305	0.7%	0.79 [0.48, 1.30]
Ashorn 2010	-0.3157	0.1681	372	360	1.5%	0.73 [0.52, 1.01]
Fawzi 2007	-0.0653	0.0434	2799	2794	22.1%	0.94 [0.86, 1.02]
Osrin 2005	-0.0914	0.1824	526	517	1.3%	0.91 [0.64, 1.30]
Roberfroid 2008	-0.0989	0.1529	534	539	1.9%	0.91 [0.67, 1.22]
Sunawang 2009	0.071	0.1211	632	649	3.0%	1.07 [0.85, 1.36]
Tofail 2008 (1)	0.0591	0.1038	483	1006	4.0%	1.06 [0.87, 1.30]
West 2014	-0.1123	0.0238	6964	6861	65.5%	0.89 [0.85, 0.94]
**Total (95% CI)**			**12623**	**13031**	**100.0%**	**0.91 [0.87, 0.95]**

Heterogeneity: Tau² = 0.00; Chi² = 7.13, df = 7 (P = 0.42); I² = 2%
Test for overall effect: Z = 4.45 (P < 0.00001)

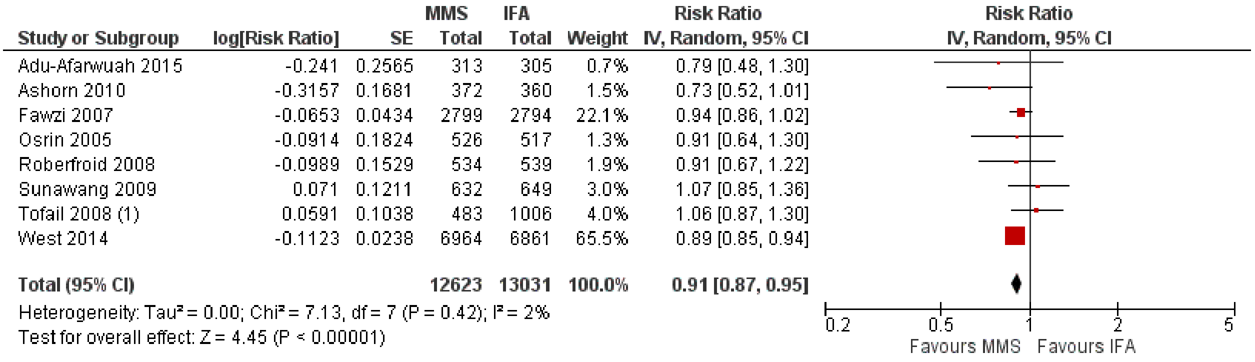

Footnotes
(1) Only the Usual Invitation to Food Supplementation groups were included. Both IFA arms were merged and compared with the MMS arm

Supplemental Figure 2.10.2 Effect of MMS vs IFA on stunting at 3

months Fixed

Study or Subgroup	log[Risk Ratio]	SE	MMS Total	IFA Total	Weight	Risk Ratio IV, Fixed, 95% CI
Adu-Afarwuah 2015	-0.3103	0.2347	357	343	1.1%	0.73 [0.46, 1.16]
Fawzi 2007	-0.0884	0.085	2907	2903	8.3%	0.92 [0.77, 1.08]
Moore 2009	0.6474	0.4884	179	171	0.3%	1.91 [0.73, 4.98]
Roberfroid 2008	-0.0476	0.1728	306	349	2.0%	0.95 [0.68, 1.34]
Sunawang 2009	0.0853	0.3036	151	148	0.6%	1.09 [0.60, 1.97]
Tofail 2008 (1)	0.075	0.1015	372	783	5.8%	1.08 [0.88, 1.32]
West 2014	-0.1728	0.027	7931	7725	81.9%	0.84 [0.80, 0.89]
**Total (95% CI)**			**12203**	**12422**	**100.0%**	**0.86 [0.82, 0.91]**

Heterogeneity: Chi² = 10.22, df = 6 (P = 0.12); I² = 41%
Test for overall effect: Z = 6.00 (P < 0.00001)

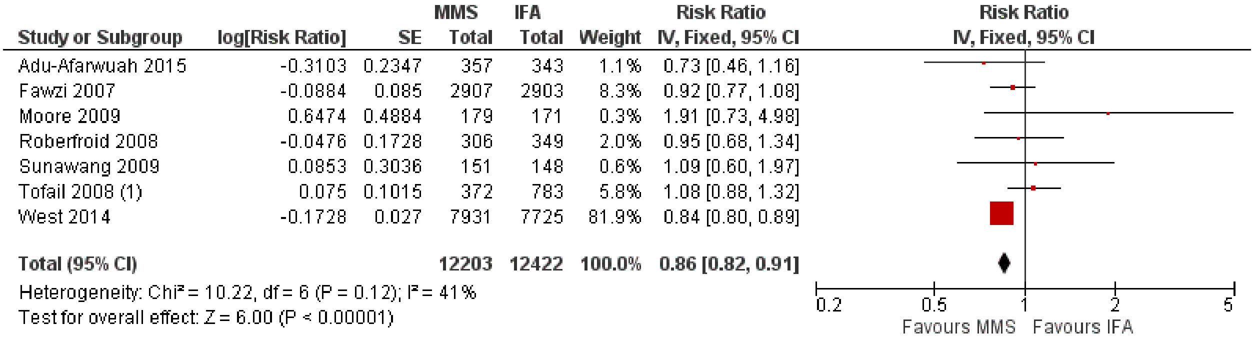

Footnotes
(1) nly the Usual Invitation to Food Supplementation groups were included. Both IFA arms were merged and compared with the MMS arm

Random

Study or Subgroup	log[Risk Ratio]	SE	MMS Total	IFA Total	Weight	Risk Ratio IV, Random, 95% CI
Adu-Afarwuah 2015	-0.3103	0.2347	357	343	5.4%	0.73 [0.46, 1.16]
Fawzi 2007	-0.0884	0.085	2907	2903	22.5%	0.92 [0.77, 1.08]
Moore 2009	0.6474	0.4884	179	171	1.4%	1.91 [0.73, 4.98]
Roberfroid 2008	-0.0476	0.1728	306	349	9.0%	0.95 [0.68, 1.34]
Sunawang 2009	0.0853	0.3036	151	148	3.4%	1.09 [0.60, 1.97]
Tofail 2008 (1)	0.075	0.1015	372	783	18.7%	1.08 [0.88, 1.32]
West 2014	-0.1728	0.027	7931	7725	39.5%	0.84 [0.80, 0.89]
**Total (95% CI)**			**12203**	**12422**	**100.0%**	**0.92 [0.82, 1.03]**

Heterogeneity: Tau² = 0.01; Chi² = 10.22, df = 6 (P = 0.12); I² = 41%
Test for overall effect: Z = 1.42 (P = 0.15)

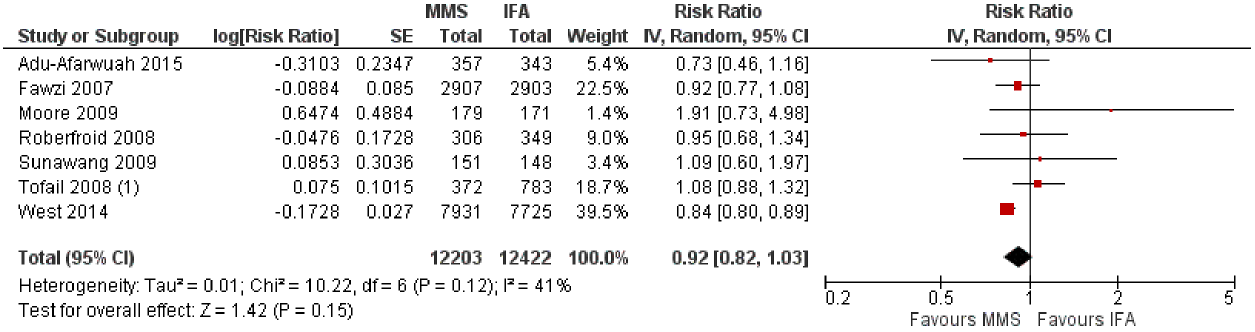

Footnotes
(1) nly the Usual Invitation to Food Supplementation groups were included. Both IFA arms were merged and compared with the MMS arm

Supplemental Figure 2.10.3 Effect of MMS vs IFA on stunting at 6 months

Fixed

Study or Subgroup	log[Risk Ratio]	SE	MMS Total	IFA Total	Weight	Risk Ratio IV, Fixed, 95% CI
Adu-Afarwuah 2015	-0.2152	0.2227	355	347	1.7%	0.81 [0.52, 1.25]
Ashorn 2010 complete FU (1)	-0.128	0.1761	213	220	2.7%	0.88 [0.62, 1.24]
Ashorn 2010 simplified FU (2)	0.0262	0.2291	131	130	1.6%	1.03 [0.66, 1.61]
Fawzi 2007	-0.0743	0.0736	3000	2978	15.4%	0.93 [0.80, 1.07]
Moore 2009	0.4637	0.4712	170	172	0.4%	1.59 [0.63, 4.00]
Roberfroid 2008	-0.0135	0.1984	270	313	2.1%	0.99 [0.67, 1.46]
Sunawang 2009	0.3629	0.3142	151	148	0.8%	1.44 [0.78, 2.66]
Tofail 2008 (3)	0.187	0.0914	445	885	10.0%	1.21 [1.01, 1.44]
West 2014	-0.0654	0.0357	4494	4429	65.4%	0.94 [0.87, 1.00]
**Total (95% CI)**			**9229**	**9622**	**100.0%**	**0.96 [0.91, 1.02]**

Heterogeneity: Chi² = 10.64, df = 8 (P = 0.22); I² = 25%
Test for overall effect: Z = 1.30 (P = 0.19)

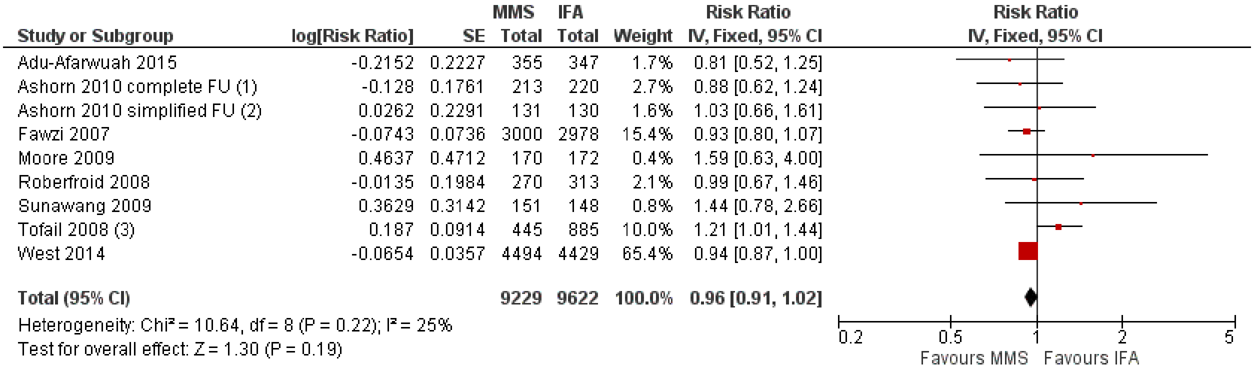

Footnotes
(1) intervention continued daily MMS for 6m PP; control received daily 200mg Ca for 6m PP
(2) intervention and control stopped at delivery and there were less follow-ups
(3) Only the Usual Invitation to Food Supplementation groups were included. Both IFA arms were merged and compared with the MMS arm

Random

Study or Subgroup	log[Risk Ratio]	SE	MMS Total	IFA Total	Weight	Risk Ratio IV, Random, 95% CI
Adu-Afarwuah 2015	-0.2152	0.2227	355	347	3.9%	0.81 [0.52, 1.25]
Ashorn 2010 complete FU (1)	-0.128	0.1761	213	220	6.0%	0.88 [0.62, 1.24]
Ashorn 2010 simplified FU (2)	0.0262	0.2291	131	130	3.7%	1.03 [0.66, 1.61]
Fawzi 2007	-0.0743	0.0736	3000	2978	22.2%	0.93 [0.80, 1.07]
Moore 2009	0.4637	0.4712	170	172	0.9%	1.59 [0.63, 4.00]
Roberfroid 2008	-0.0135	0.1984	270	313	4.9%	0.99 [0.67, 1.46]
Sunawang 2009	0.3629	0.3142	151	148	2.1%	1.44 [0.78, 2.66]
Tofail 2008 (3)	0.187	0.0914	445	885	17.0%	1.21 [1.01, 1.44]
West 2014	-0.0654	0.0357	4494	4429	39.3%	0.94 [0.87, 1.00]
**Total (95% CI)**			**9229**	**9622**	**100.0%**	**0.99 [0.90, 1.08]**

Heterogeneity: Tau² = 0.00; Chi² = 10.64, df = 8 (P = 0.22); I² = 25%
Test for overall effect: Z = 0.32 (P = 0.75)

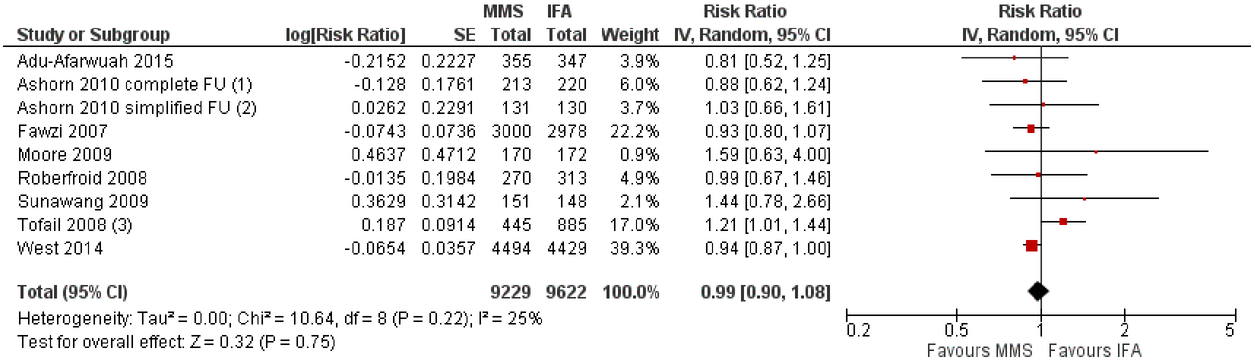

Footnotes
(1) intervention continued daily MMS for 6m PP; control received daily 200mg Ca for 6m PP
(2) intervention and control stopped at delivery and there were less follow-ups
(3) Only the Usual Invitation to Food Supplementation groups were included. Both IFA arms were merged and compared with the MMS arm

Supplemental Figure 2.10.4 Effect of MMS vs IFA on stunting at 12

months Fixed

Study or Subgroup	log[Risk Ratio]	SE	MMS Total	IFA Total	Weight	Risk Ratio IV, Fixed, 95% CI
Adu-Afarwuah 2015	-0.0094	0.2282	345	332	0.9%	0.99 [0.63, 1.55]
Ashorn 2010 complete FU (1)	-0.0628	0.1527	214	218	2.0%	0.94 [0.70, 1.27]
Fawzi 2007	0.0613	0.0654	1630	1609	10.9%	1.06 [0.94, 1.21]
Roberfroid 2008	0.1006	0.1604	240	248	1.8%	1.11 [0.81, 1.51]
Sunawang 2009	0.3629	0.3142	151	148	0.5%	1.44 [0.78, 2.66]
Tofail 2008 (2)	0.0514	0.0717	502	1023	9.0%	1.05 [0.91, 1.21]
West 2014	0.0002	0.0249	4851	4785	74.9%	1.00 [0.95, 1.05]
**Total (95% CI)**			**7933**	**8363**	**100.0%**	**1.01 [0.97, 1.06]**

Heterogeneity: Chi² = 2.89, df = 6 (P = 0.82); I² = 0%
Test for overall effect: Z = 0.63 (P = 0.53)

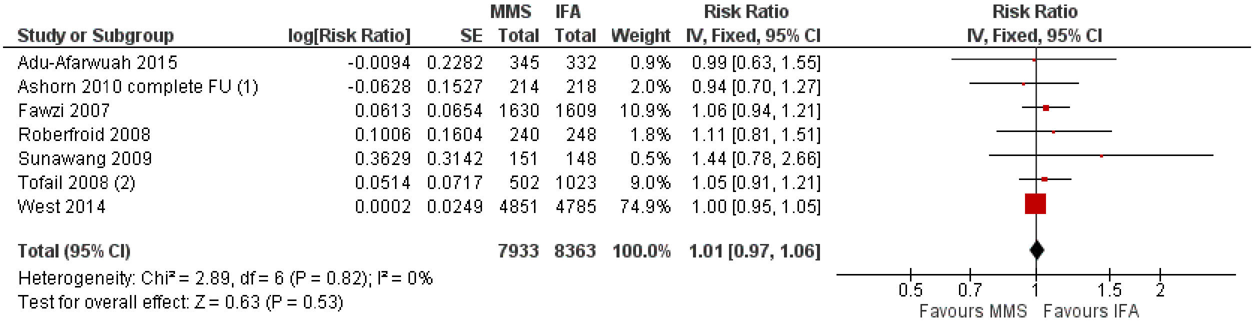

Footnotes
(1) intervention continued daily MMS for 6m PP; control received daily 200mg Ca for 6m PP
(2) Only the Usual Invitation to Food Supplementation groups were included. Both IFA arms were merged and compared with the MMS arm

Random

Study or Subgroup	log[Risk Ratio]	SE	MMS Total	IFA Total	Weight	Risk Ratio IV, Random, 95% CI
Adu-Afarwuah 2015	-0.0094	0.2282	345	332	0.9%	0.99 [0.63, 1.55]
Ashorn 2010 complete FU (1)	-0.0628	0.1527	214	218	2.0%	0.94 [0.70, 1.27]
Fawzi 2007	0.0613	0.0654	1630	1609	10.9%	1.06 [0.94, 1.21]
Roberfroid 2008	0.1006	0.1604	240	248	1.8%	1.11 [0.81, 1.51]
Sunawang 2009	0.3629	0.3142	151	148	0.5%	1.44 [0.78, 2.66]
Tofail 2008 (2)	0.0514	0.0717	502	1023	9.0%	1.05 [0.91, 1.21]
West 2014	0.0002	0.0249	4851	4785	74.9%	1.00 [0.95, 1.05]
**Total (95% CI)**			**7933**	**8363**	**100.0%**	**1.01 [0.97, 1.06]**

Heterogeneity: Tau² = 0.00; Chi² = 2.89, df = 6 (P = 0.82); I² = 0%
Test for overall effect: Z = 0.63 (P = 0.53)

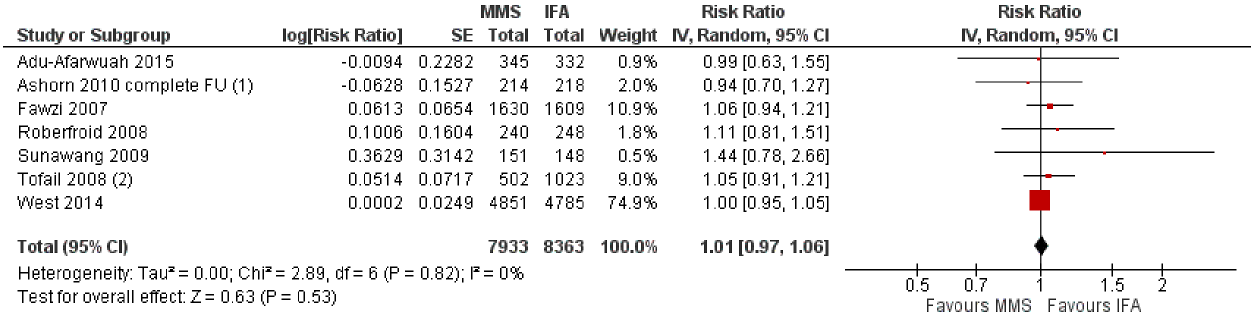

Footnotes
(1) intervention continued daily MMS for 6m PP; control received daily 200mg Ca for 6m PP
(2) Only the Usual Invitation to Food Supplementation groups were included. Both IFA arms were merged and compared with the MMS arm

Supplemental Figure 2.10.5 Effect of MMS vs IFA on stunting at 18 months

Fixed

Study or Subgroup	log[Risk Ratio]	SE	MMS Total	IFA Total	Weight	Risk Ratio IV, Fixed, 95% CI
Adu-Afarwuah 2015	-0.2703	0.1955	355	337	6.2%	0.76 [0.52, 1.12]
Ashorn 2010 complete FU (1)	0.1087	0.1311	222	220	13.7%	1.11 [0.86, 1.44]
Ashorn 2010 simplified FU (2)	-0.037	0.1468	129	129	10.9%	0.96 [0.72, 1.28]
Fawzi 2007	-0.4829	0.4019	39	55	1.5%	0.62 [0.28, 1.36]
Tofail 2008	0.0043	0.0589	463	974	67.8%	1.00 [0.89, 1.13]
**Total (95% CI)**			**1208**	**1715**	**100.0%**	**0.99 [0.90, 1.09]**

Heterogeneity: Chi² = 4.07, df = 4 (P = 0.40); I² = 2%
Test for overall effect: Z = 0.20 (P = 0.84)

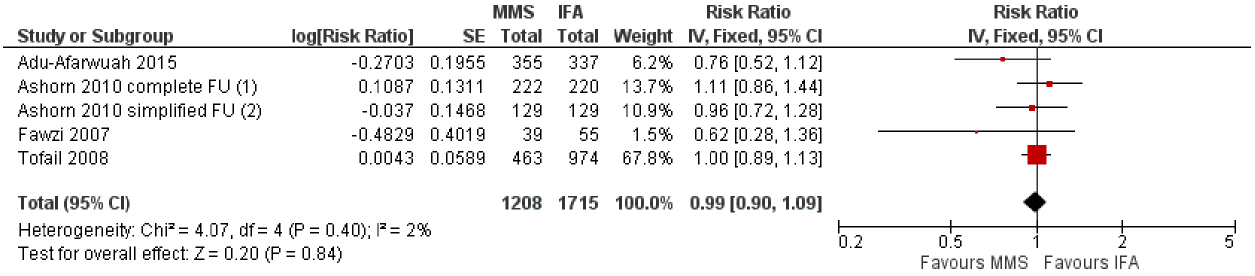

Footnotes
(1) intervention continued daily MMS for 6m PP; control received daily 200mg Ca for 6m PP
(2) intervention and control stopped at delivery and there were less follow-ups

Random

Study or Subgroup	log[Risk Ratio]	SE	MMS Total	IFA Total	Weight	Risk Ratio IV, Random, 95% CI
Adu-Afarwuah 2015	-0.2703	0.1955	355	337	6.5%	0.76 [0.52, 1.12]
Ashorn 2010 complete FU (1)	0.1087	0.1311	222	220	14.3%	1.11 [0.86, 1.44]
Ashorn 2010 simplified FU (2)	-0.037	0.1468	129	129	11.5%	0.96 [0.72, 1.28]
Fawzi 2007	-0.4829	0.4019	39	55	1.6%	0.62 [0.28, 1.36]
Tofail 2008	0.0043	0.0589	463	974	66.1%	1.00 [0.89, 1.13]
**Total (95% CI)**			**1208**	**1715**	**100.0%**	**0.99 [0.90, 1.09]**

Heterogeneity: Tau² = 0.00; Chi² = 4.07, df = 4 (P = 0.40); I² = 2%
Test for overall effect: Z = 0.22 (P = 0.83)

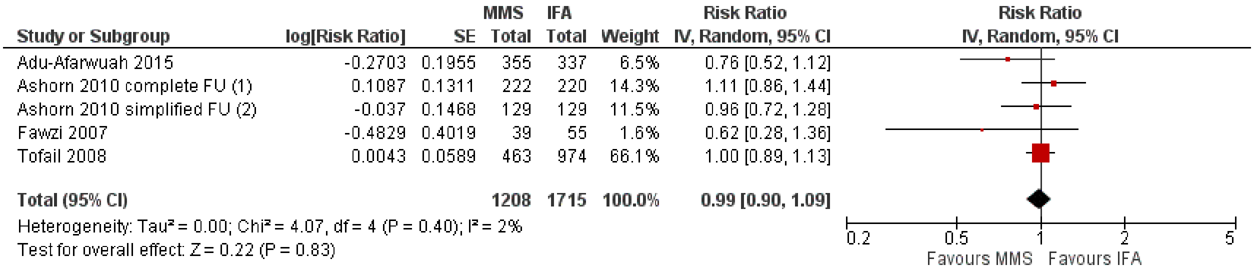

Footnotes
(1) intervention continued daily MMS for 6m PP; control received daily 200mg Ca for 6m PP
(2) intervention and control stopped at delivery and there were less follow-ups

Supplemental Figure 2.10.6 Effect of MMS vs IFA on stunting at 24 months

Fixed

Study or Subgroup	log[Risk Ratio]	SE	MMS Total	IFA Total	Weight	Risk Ratio IV, Fixed, 95% CI
Ashorn 2010 complete FU (1)	-0.0712	0.1316	179	197	2.6%	0.93 [0.72, 1.21]
Bliznashka 2021	0.01	0.0703	642	718	9.2%	1.01 [0.88, 1.16]
Roberfroid 2008	0.1462	0.2208	54	55	0.9%	1.16 [0.75, 1.78]
Tofail 2008 (2)	0.0512	0.0531	479	967	16.0%	1.05 [0.95, 1.17]
West 2014	0.0246	0.0252	3283	3231	71.3%	1.02 [0.98, 1.08]
**Total (95% CI)**			**4637**	**5168**	**100.0%**	**1.03 [0.98, 1.07]**

Heterogeneity: Chi² = 1.12, df = 4 (P = 0.89); I² = 0%
Test for overall effect: Z = 1.23 (P = 0.22)

Risk Ratio IV, Fixed, 95% CI

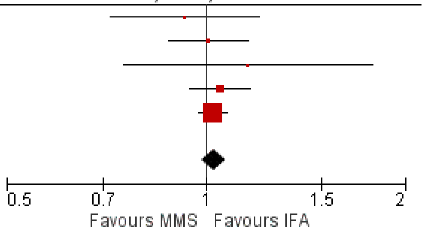

Footnotes
(1) intervention continued daily MMS for 6m PP; control received daily 200mg Ca for 6m PP
(2) Only the Usual Invitation to Food Supplementation groups were included. Both IFA arms were merged and compared with the MMS arm

Random

Study or Subgroup	log[Risk Ratio]	SE	MMS Total	IFA Total	Weight	Risk Ratio IV, Random, 95% CI
Ashorn 2010 complete FU (1)	-0.0712	0.1316	179	197	2.6%	0.93 [0.72, 1.21]
Bliznashka 2021	0.01	0.0703	642	718	9.2%	1.01 [0.88, 1.16]
Roberfroid 2008	0.1462	0.2208	54	55	0.9%	1.16 [0.75, 1.78]
Tofail 2008 (2)	0.0512	0.0531	479	967	16.0%	1.05 [0.95, 1.17]
West 2014	0.0246	0.0252	3283	3231	71.3%	1.02 [0.98, 1.08]
**Total (95% CI)**			**4637**	**5168**	**100.0%**	**1.03 [0.98, 1.07]**

Heterogeneity: Tau² = 0.00; Chi² = 1.12, df = 4 (P = 0.89); I² = 0%
Test for overall effect: Z = 1.23 (P = 0.22)

Risk Ratio IV, Random, 95% CI

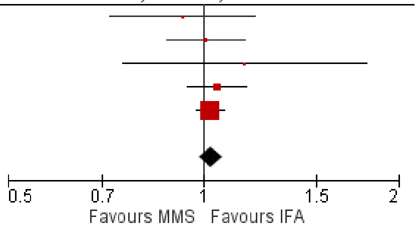

Footnotes
(1) intervention continued daily MMS for 6m PP; control received daily 200mg Ca for 6m PP
(2) Only the Usual Invitation to Food Supplementation groups were included. Both IFA arms were merged and compared with the MMS arm

Supplemental Figure 2.11. Effect of MMS vs IFA on underweight

Supplemental Figure 2.11.1. Effect of MMS vs IFA on underweight at birth

Fixed

Study or Subgroup	log[Risk Ratio]	SE	MMs Total	IFA Total	Weight	Risk Ratio IV, Fixed, 95% CI
Adu-Afarwuah 2015	-0.2886	0.2546	318	305	0.7%	0.75 [0.45, 1.23]
Ashorn 2010	-0.1917	0.2418	374	362	0.7%	0.83 [0.51, 1.33]
Fawzi 2007	-0.1452	0.0807	3838	3823	6.6%	0.86 [0.74, 1.01]
Moore 2009	-0.3515	0.4276	178	167	0.2%	0.70 [0.30, 1.63]
Osrin 2005	-0.2673	0.1212	529	523	2.9%	0.77 [0.60, 0.97]
Roberfroid 2008	0.0675	0.1564	535	541	1.8%	1.07 [0.79, 1.45]
Sunawang 2009	-0.1848	0.2238	632	649	0.9%	0.83 [0.54, 1.29]
Tofail 2008	-0.0612	0.0867	490	1012	5.8%	0.94 [0.79, 1.11]
West 2014	-0.1358	0.0232	7127	7041	80.4%	0.87 [0.83, 0.91]
**Total (95% CI)**			**14021**	**14423**	**100.0%**	**0.87 [0.84, 0.91]**

Heterogeneity: Chi² = 4.33, df = 8 (P = 0.83); I² = 0%
Test for overall effect: Z = 6.48 (P < 0.00001)

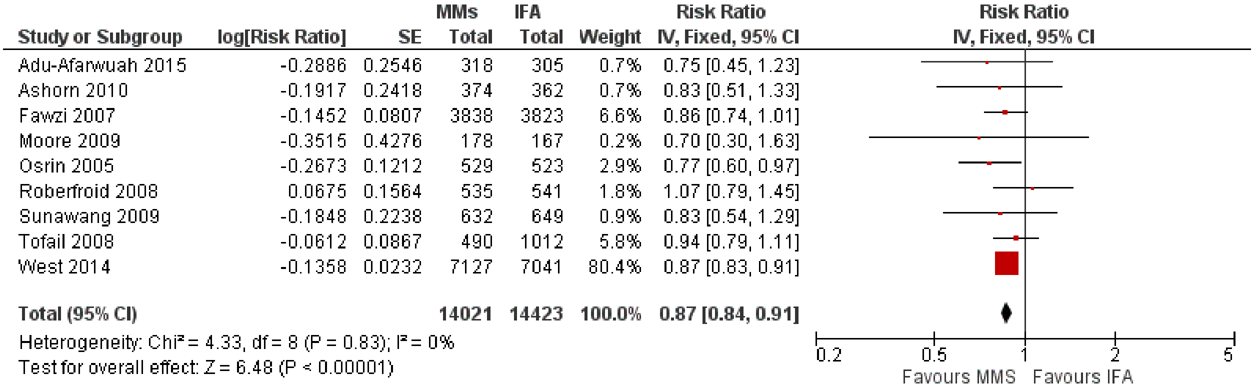

Random

Study or Subgroup	log[Risk Ratio]	SE	MMs Total	IFA Total	Weight	Risk Ratio IV, Random, 95% CI
Adu-Afarwuah 2015	-0.2886	0.2546	318	305	0.7%	0.75 [0.45, 1.23]
Ashorn 2010	-0.1917	0.2418	374	362	0.7%	0.83 [0.51, 1.33]
Fawzi 2007	-0.1452	0.0807	3838	3823	6.6%	0.86 [0.74, 1.01]
Moore 2009	-0.3515	0.4276	178	167	0.2%	0.70 [0.30, 1.63]
Osrin 2005	-0.2673	0.1212	529	523	2.9%	0.77 [0.60, 0.97]
Roberfroid 2008	0.0675	0.1564	535	541	1.8%	1.07 [0.79, 1.45]
Sunawang 2009	-0.1848	0.2238	632	649	0.9%	0.83 [0.54, 1.29]
Tofail 2008	-0.0612	0.0867	490	1012	5.8%	0.94 [0.79, 1.11]
West 2014	-0.1358	0.0232	7127	7041	80.4%	0.87 [0.83, 0.91]
**Total (95% CI)**			**14021**	**14423**	**100.0%**	**0.87 [0.84, 0.91]**

Heterogeneity: Tau² = 0.00; Chi² = 4.33, df = 8 (P = 0.83); I² = 0%
Test for overall effect: Z = 6.48 (P < 0.00001)

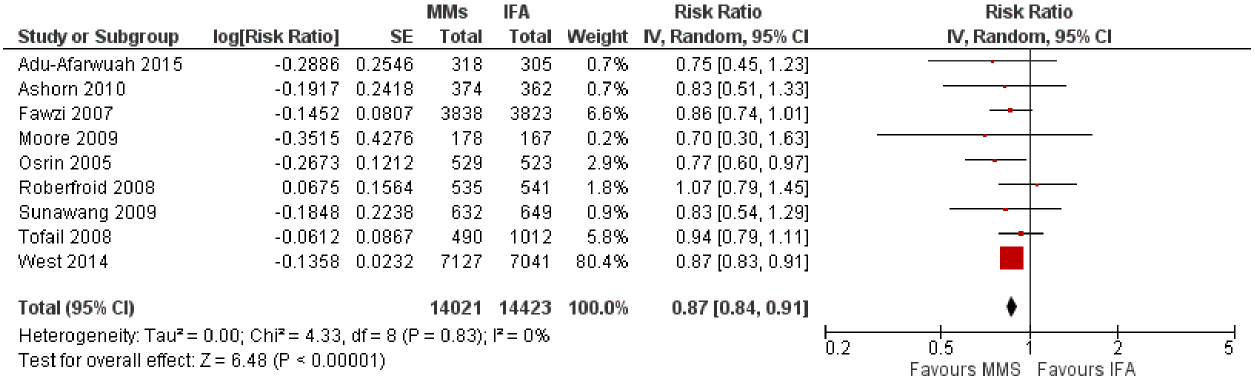

Supplemental Figure 2.11.2 Effect of MMS vs IFA on underweight at 3 months

Fixed

Study or Subgroup	log[Risk Ratio]	SE	MMs Total	IFA Total	Weight	Risk Ratio IV, Fixed, 95% CI
Adu-Afarwuah 2015	-0.6559	0.336	358	343	0.6%	0.52 [0.27, 1.00]
Fawzi 2007	-0.2137	0.1378	3011	3013	3.4%	0.81 [0.62, 1.06]
Moore 2009	-0.3081	0.4068	179	171	0.4%	0.73 [0.33, 1.63]
Roberfroid 2008	-0.441	0.2549	306	349	1.0%	0.64 [0.39, 1.06]
Sunawang 2009	-0.0201	0.3748	151	148	0.5%	0.98 [0.47, 2.04]
Tofail 2008	0.0334	0.1164	373	794	4.7%	1.03 [0.82, 1.30]
West 2014	-0.1566	0.0268	7996	7777	89.5%	0.86 [0.81, 0.90]
**Total (95% CI)**			**12374**	**12595**	**100.0%**	**0.86 [0.81, 0.90]**

Heterogeneity: Chi² = 6.56, df = 6 (P = 0.36); I² = 8%
Test for overall effect: Z = 6.12 (P < 0.00001)

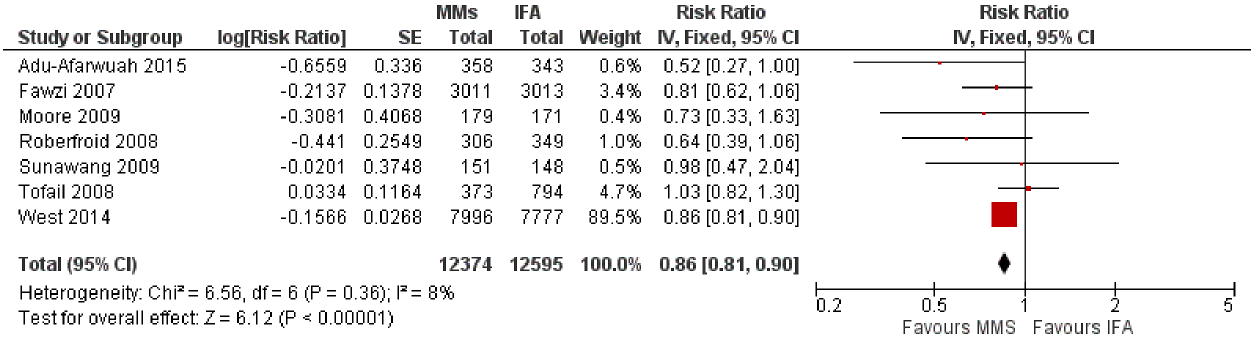

Random

Study or Subgroup	log[Risk Ratio]	SE	MMs Total	IFA Total	Weight	Risk Ratio IV, Random, 95% CI
Adu-Afarwuah 2015	-0.6559	0.336	358	343	1.6%	0.52 [0.27, 1.00]
Fawzi 2007	-0.2137	0.1378	3011	3013	8.9%	0.81 [0.62, 1.06]
Moore 2009	-0.3081	0.4068	179	171	1.1%	0.73 [0.33, 1.63]
Roberfroid 2008	-0.441	0.2549	306	349	2.8%	0.64 [0.39, 1.06]
Sunawang 2009	-0.0201	0.3748	151	148	1.3%	0.98 [0.47, 2.04]
Tofail 2008	0.0334	0.1164	373	794	12.0%	1.03 [0.82, 1.30]
West 2014	-0.1566	0.0268	7996	7777	72.4%	0.86 [0.81, 0.90]
**Total (95% CI)**			**12374**	**12595**	**100.0%**	**0.86 [0.79, 0.93]**

Heterogeneity: Tau² = 0.00; Chi² = 6.56, df = 6 (P = 0.36); I² = 8%
Test for overall effect: Z = 3.60 (P = 0.0003)

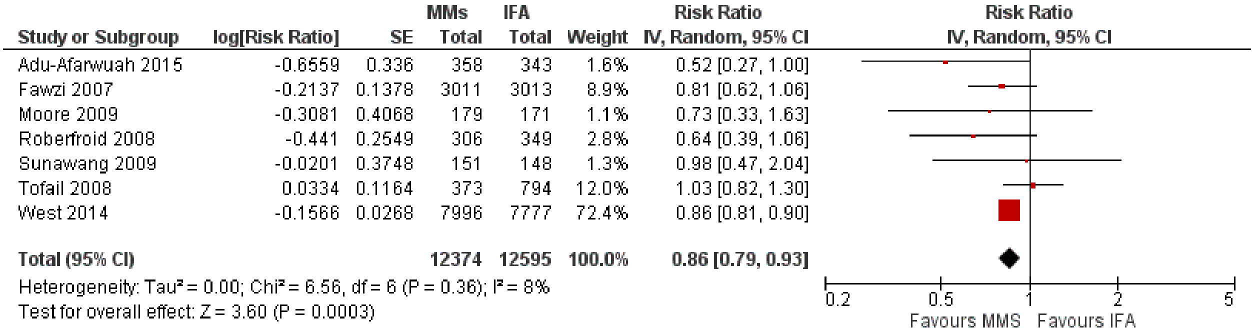

Supplemental Figure 2.11.3 Effect of MMS vs IFA on underweight at 6 months

Fixed

Study or Subgroup	log[Risk Ratio]	SE	MMs Total	IFA Total	Weight	Risk Ratio IV, Fixed, 95% CI
Adu-Afarwuah 2015	-0.4051	0.2431	356	347	1.7%	0.67 [0.41, 1.07]
Ashorn 2010 complete FU	-0.2581	0.3177	216	219	1.0%	0.77 [0.41, 1.44]
Ashorn 2010 simplified FU	0.0793	0.3986	131	130	0.6%	1.08 [0.50, 2.36]
Fawzi 2007	-0.0196	0.1002	3050	3023	10.0%	0.98 [0.81, 1.19]
Moore 2009	0.1288	0.3158	169	172	1.0%	1.14 [0.61, 2.11]
Roberfroid 2008	-0.1554	0.1712	270	313	3.4%	0.86 [0.61, 1.20]
Sunawang 2009	0.2312	0.3371	151	148	0.9%	1.26 [0.65, 2.44]
Tofail 2008	0.0572	0.1141	447	983	7.7%	1.06 [0.85, 1.32]
West 2014	-0.025	0.0368	4539	4467	73.8%	0.98 [0.91, 1.05]
**Total (95% CI)**			**9329**	**9802**	**100.0%**	**0.97 [0.91, 1.04]**

Heterogeneity: Chi² = 4.96, df = 8 (P = 0.76); I² = 0%
Test for overall effect: Z = 0.85 (P = 0.40)

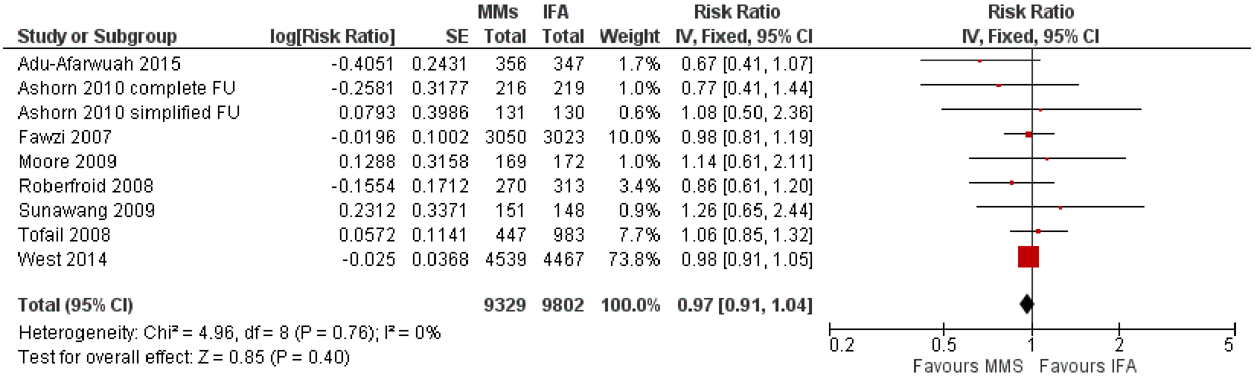

Random

Study or Subgroup	log[Risk Ratio]	SE	MMs Total	IFA Total	Weight	Risk Ratio IV, Random, 95% CI
Adu-Afarwuah 2015	-0.4051	0.2431	356	347	1.7%	0.67 [0.41, 1.07]
Ashorn 2010 complete FU	-0.2581	0.3177	216	219	1.0%	0.77 [0.41, 1.44]
Ashorn 2010 simplified FU	0.0793	0.3986	131	130	0.6%	1.08 [0.50, 2.36]
Fawzi 2007	-0.0196	0.1002	3050	3023	10.0%	0.98 [0.81, 1.19]
Moore 2009	0.1288	0.3158	169	172	1.0%	1.14 [0.61, 2.11]
Roberfroid 2008	-0.1554	0.1712	270	313	3.4%	0.86 [0.61, 1.20]
Sunawang 2009	0.2312	0.3371	151	148	0.9%	1.26 [0.65, 2.44]
Tofail 2008	0.0572	0.1141	447	983	7.7%	1.06 [0.85, 1.32]
West 2014	-0.025	0.0368	4539	4467	73.8%	0.98 [0.91, 1.05]
**Total (95% CI)**			**9329**	**9802**	**100.0%**	**0.97 [0.91, 1.04]**

Heterogeneity: Tau² = 0.00; Chi² = 4.96, df = 8 (P = 0.76); I² = 0%
Test for overall effect: Z = 0.85 (P = 0.40)

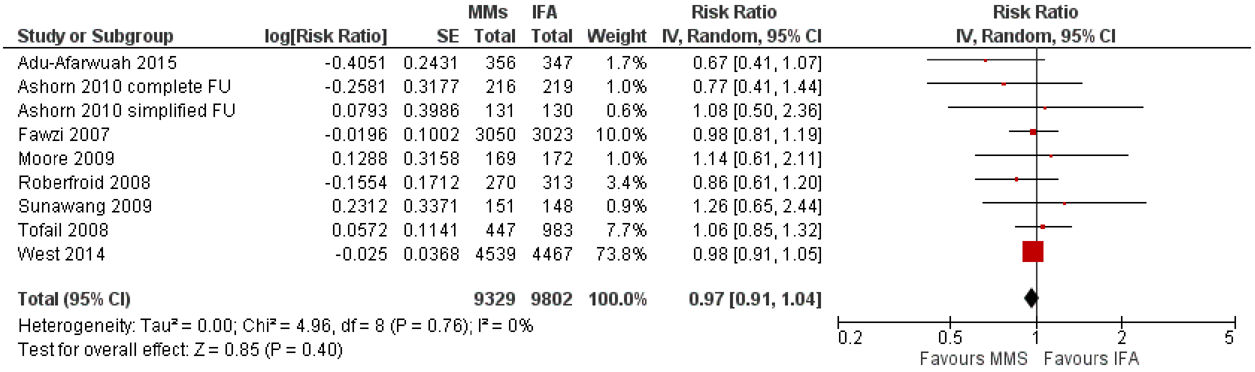

Supplemental Figure 2.11.4 Effect of MMS vs IFA on underweight at 12 months

Fixed

Study or Subgroup	log[Risk Ratio]	SE	MMs Total	IFA Total	Weight	Risk Ratio IV, Fixed, 95% CI
Adu-Afarwuah 2015	-0.4903	0.2207	346	332	1.1%	0.61 [0.40, 0.94]
Ashorn 2010 complete FU	0.3286	0.2638	215	219	0.8%	1.39 [0.83, 2.33]
Fawzi 2007	0.0095	0.092	1644	1620	6.4%	1.01 [0.84, 1.21]
Roberfroid 2008	-0.0326	0.1341	240	248	3.0%	0.97 [0.74, 1.26]
Sunawang 2009	-0.0201	0.5219	151	148	0.2%	0.98 [0.35, 2.73]
Tofail 2008	-0.1172	0.0885	504	1028	6.9%	0.89 [0.75, 1.06]
West 2014	-0.0261	0.0257	4961	4867	81.7%	0.97 [0.93, 1.02]
**Total (95% CI)**			**8061**	**8462**	**100.0%**	**0.97 [0.92, 1.01]**

Heterogeneity: Chi² = 7.36, df = 6 (P = 0.29); I² = 19%
Test for overall effect: Z = 1.41 (P = 0.16)

Risk Ratio
IV, Fixed, 95% CI

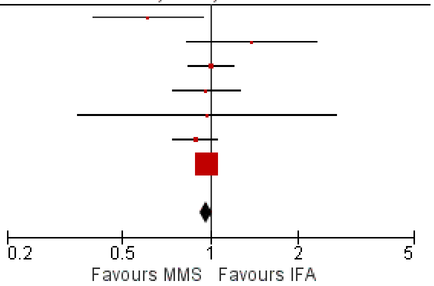

Random

Study or Subgroup	log[Risk Ratio]	SE	MMs Total	IFA Total	Weight	Risk Ratio IV, Random, 95% CI
Adu-Afarwuah 2015	-0.4903	0.2207	346	332	3.2%	0.61 [0.40, 0.94]
Ashorn 2010 complete FU	0.3286	0.2638	215	219	2.2%	1.39 [0.83, 2.33]
Fawzi 2007	0.0095	0.092	1644	1620	15.0%	1.01 [0.84, 1.21]
Roberfroid 2008	-0.0326	0.1341	240	248	8.0%	0.97 [0.74, 1.26]
Sunawang 2009	-0.0201	0.5219	151	148	0.6%	0.98 [0.35, 2.73]
Tofail 2008	-0.1172	0.0885	504	1028	16.0%	0.89 [0.75, 1.06]
West 2014	-0.0261	0.0257	4961	4867	55.0%	0.97 [0.93, 1.02]
**Total (95% CI)**			**8061**	**8462**	**100.0%**	**0.96 [0.89, 1.04]**

Heterogeneity: Tau² = 0.00; Chi² = 7.36, df = 6 (P = 0.29); I² = 19%
Test for overall effect: Z = 1.06 (P = 0.29)

Risk Ratio
IV, Random, 95% CI

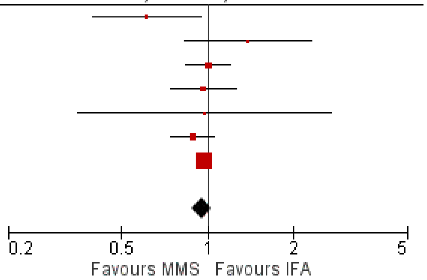

Supplemental Figure 2.11.5 Effect of MMS vs IFA on underweight at 18 months

Fixed

Study or Subgroup	log[Risk Ratio]	SE	MMs Total	IFA Total	Weight	Risk Ratio IV, Fixed, 95% CI
Adu-Afarwuah 2015	-0.4787	0.2005	356	337	11.7%	0.62 [0.42, 0.92]
Ashorn 2010 complete FU	0.3011	0.2641	222	220	6.8%	1.35 [0.81, 2.27]
Ashorn 2010 simplified FU	-0.0668	0.2952	130	128	5.4%	0.94 [0.52, 1.67]
Fawzi 2007	0.0671	0.4591	40	55	2.2%	1.07 [0.43, 2.63]
Tofail 2008	-0.1103	0.0799	463	974	73.9%	0.90 [0.77, 1.05]
**Total (95% CI)**			**1211**	**1714**	**100.0%**	**0.89 [0.78, 1.02]**

Heterogeneity: Chi² = 5.96, df = 4 (P = 0.20); I² = 33%
Test for overall effect: Z = 1.74 (P = 0.08)

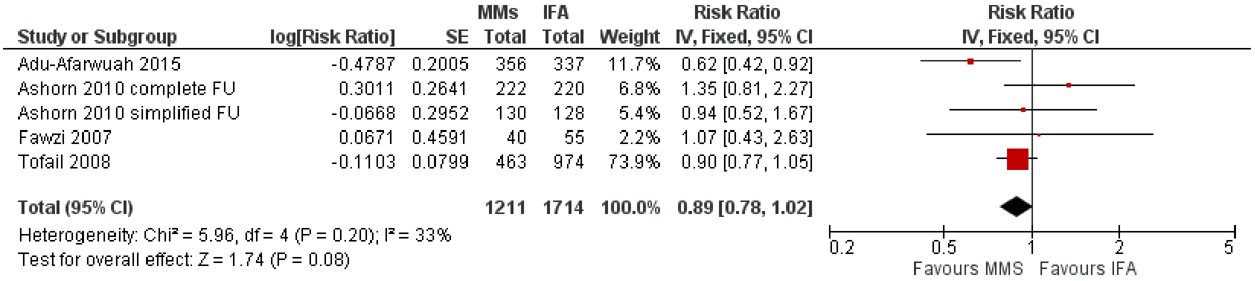

Random

Study or Subgroup	log[Risk Ratio]	SE	MMs Total	IFA Total	Weight	Risk Ratio IV, Random, 95% CI
Adu-Afarwuah 2015	-0.4787	0.2005	356	337	21.1%	0.62 [0.42, 0.92]
Ashorn 2010 complete FU	0.3011	0.2641	222	220	14.3%	1.35 [0.81, 2.27]
Ashorn 2010 simplified FU	-0.0668	0.2952	130	128	12.0%	0.94 [0.52, 1.67]
Fawzi 2007	0.0671	0.4591	40	55	5.6%	1.07 [0.43, 2.63]
Tofail 2008	-0.1103	0.0799	463	974	47.0%	0.90 [0.77, 1.05]
**Total (95% CI)**			**1211**	**1714**	**100.0%**	**0.89 [0.71, 1.12]**

Heterogeneity: Tau² = 0.02; Chi² = 5.96, df = 4 (P = 0.20); I² = 33%
Test for overall effect: Z = 1.00 (P = 0.32)

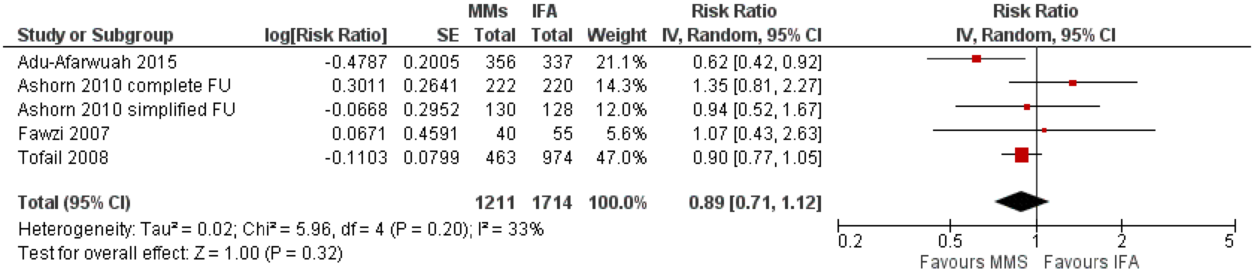

Supplemental Figure 2.11.6 Effect of MMS vs IFA on underweight at 24 months

Fixed

Study or Subgroup	log[Risk Ratio]	SE	MMS Total	IFA Total	Weight	Risk Ratio IV, Fixed, 95% CI
Ashorn 2010 complete FU	0.2779	0.2449	177	196	0.9%	1.32 [0.82, 2.13]
Bliznashka 2021	0.077	0.1162	642	718	3.9%	1.08 [0.86, 1.36]
Roberfroid 2008	-0.074	0.158	54	55	2.1%	0.93 [0.68, 1.27]
Tofail 2008	-0.0487	0.0722	481	968	10.1%	0.95 [0.83, 1.10]
West 2014	0.0103	0.0252	3412	3370	83.0%	1.01 [0.96, 1.06]
**Total (95% CI)**			**4766**	**5307**	**100.0%**	**1.01 [0.96, 1.05]**

Heterogeneity: Chi² = 2.46, df = 4 (P = 0.65); I² = 0%
Test for overall effect: Z = 0.33 (P = 0.74)

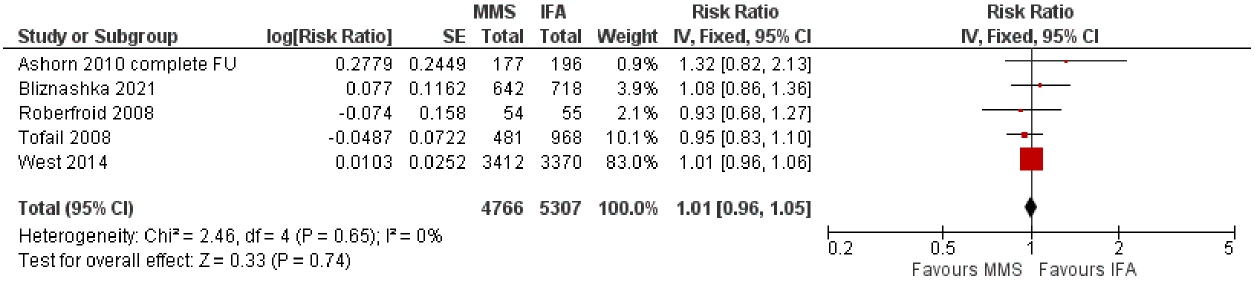

Random

Study or Subgroup	log[Risk Ratio]	SE	MMS Total	IFA Total	Weight	Risk Ratio IV, Random, 95% CI
Ashorn 2010 complete FU	0.2779	0.2449	177	196	0.9%	1.32 [0.82, 2.13]
Bliznashka 2021	0.077	0.1162	642	718	3.9%	1.08 [0.86, 1.36]
Roberfroid 2008	-0.074	0.158	54	55	2.1%	0.93 [0.68, 1.27]
Tofail 2008	-0.0487	0.0722	481	968	10.1%	0.95 [0.83, 1.10]
West 2014	0.0103	0.0252	3412	3370	83.0%	1.01 [0.96, 1.06]
**Total (95% CI)**			**4766**	**5307**	**100.0%**	**1.01 [0.96, 1.05]**

Heterogeneity: Tau² = 0.00; Chi² = 2.46, df = 4 (P = 0.65); I² = 0%
Test for overall effect: Z = 0.33 (P = 0.74)

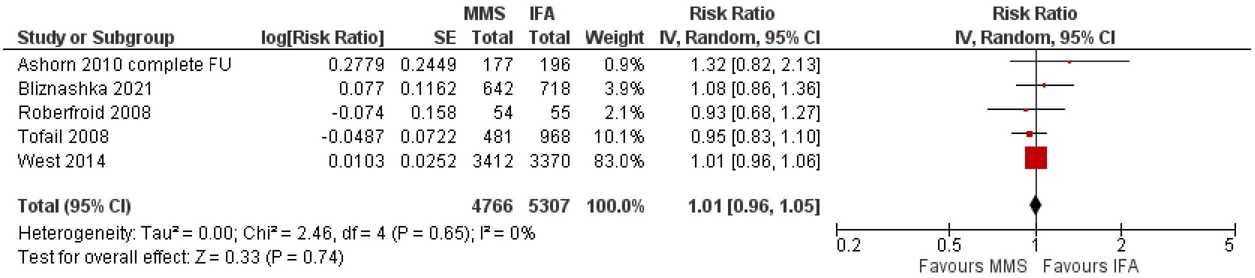

Supplemental Figure 2.12. Effect of MMS vs IFA on wasting

Supplemental Figure 2.12.1. Effect of MMS vs IFA on wasting at birth

Fixed

Study or Subgroup	log[Risk Ratio]	SE	MMS Total	IFA Total	Weight	Risk Ratio IV, Fixed, 95% CI
Adu-Afarwuah 2015	-0.1016	0.3499	302	291	0.9%	0.90 [0.46, 1.79]
Ashorn 2010	-0.1255	0.3639	364	344	0.8%	0.88 [0.43, 1.80]
Fawzi 2007	-0.1128	0.0528	2229	2243	37.5%	0.89 [0.81, 0.99]
Osrin 2005	-0.219	0.0828	526	517	15.2%	0.80 [0.68, 0.94]
Roberfroid 2008 (1)	0.1787	0.1527	534	536	4.5%	1.20 [0.89, 1.61]
Sunawang 2009	-0.6536	1.2233	570	593	0.1%	0.52 [0.05, 5.72]
Tofail 2008 (2)	-0.1227	0.118	438	926	7.5%	0.88 [0.70, 1.11]
West 2014	-0.0683	0.0558	5695	5459	33.6%	0.93 [0.84, 1.04]
**Total (95% CI)**			**10658**	**10909**	**100.0%**	**0.90 [0.85, 0.96]**

Heterogeneity: Chi² = 6.02, df = 7 (P = 0.54); I² = 0%
Test for overall effect: Z = 3.16 (P = 0.002)

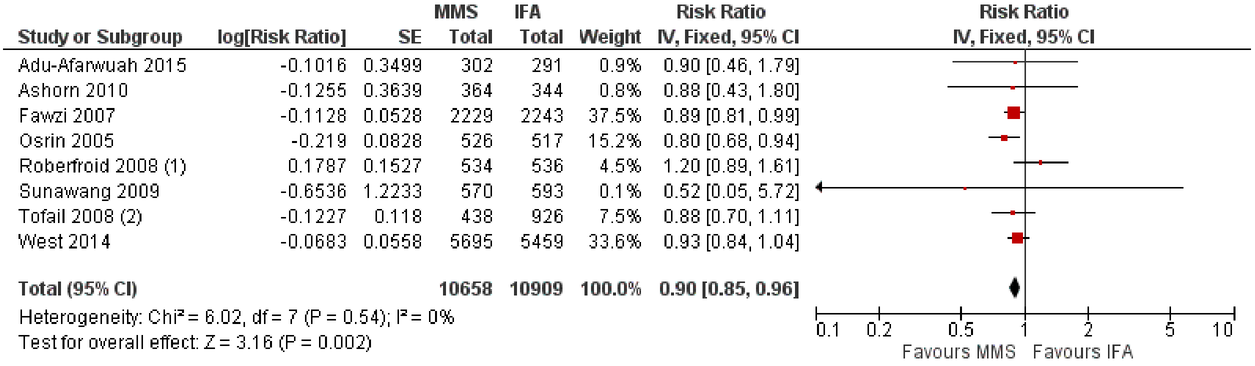

Footnotes
(1) BMIZ<-2
(2) Only the Usual Invitation to Food Supplementation groups were included. Both IFA arms were merged and compared with the MMS arm

Random

Study or Subgroup	log[Risk Ratio]	SE	MMS Total	IFA Total	Weight	Risk Ratio IV, Random, 95% CI
Adu-Afarwuah 2015	-0.1016	0.3499	302	291	0.9%	0.90 [0.46, 1.79]
Ashorn 2010	-0.1255	0.3639	364	344	0.8%	0.88 [0.43, 1.80]
Fawzi 2007	-0.1128	0.0528	2229	2243	37.5%	0.89 [0.81, 0.99]
Osrin 2005	-0.219	0.0828	526	517	15.2%	0.80 [0.68, 0.94]
Roberfroid 2008 (1)	0.1787	0.1527	534	536	4.5%	1.20 [0.89, 1.61]
Sunawang 2009	-0.6536	1.2233	570	593	0.1%	0.52 [0.05, 5.72]
Tofail 2008 (2)	-0.1227	0.118	438	926	7.5%	0.88 [0.70, 1.11]
West 2014	-0.0683	0.0558	5695	5459	33.6%	0.93 [0.84, 1.04]
**Total (95% CI)**			**10658**	**10909**	**100.0%**	**0.90 [0.85, 0.96]**

Heterogeneity: Tau² = 0.00; Chi² = 6.02, df = 7 (P = 0.54); I² = 0%
Test for overall effect: Z = 3.16 (P = 0.002)

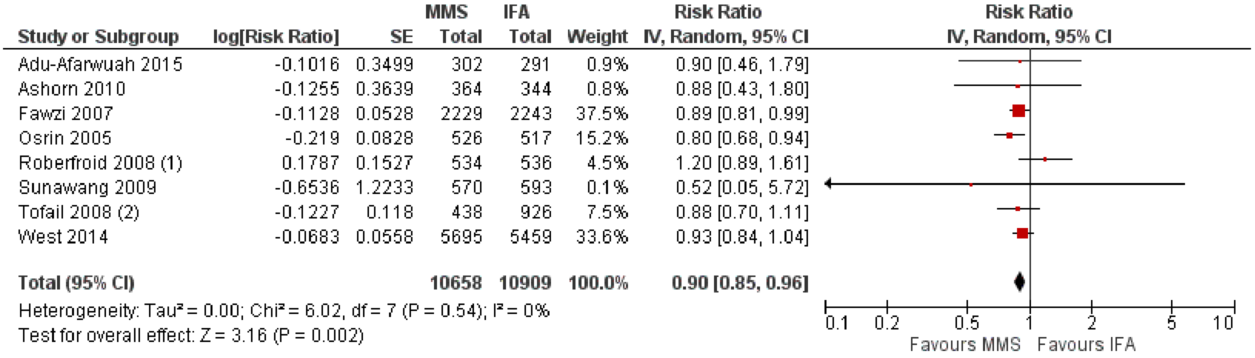

Footnotes
(1) BMIZ<-2
(2) Only the Usual Invitation to Food Supplementation groups were included. Both IFA arms were merged and compared with the MMS arm

Supplemental Figure 2.12.2 Effect of MMS vs IFA on wasting at 3 months

Fixed

Study or Subgroup	log[Risk Ratio]	SE	MMS Total	IFA Total	Weight	Risk Ratio IV, Fixed, 95% CI
Adu-Afarwuah 2015	-0.6278	0.5526	357	343	0.9%	0.53 [0.18, 1.58]
Fawzi 2007	-0.0028	0.1419	2897	2889	13.2%	1.00 [0.76, 1.32]
Moore 2009	0.0878	0.35	179	171	2.2%	1.09 [0.55, 2.17]
Roberfroid 2008	0.1315	0.4649	306	349	1.2%	1.14 [0.46, 2.84]
Sunawang 2009	0.1623	0.5944	151	148	0.8%	1.18 [0.37, 3.77]
Tofail 2008 (1)	0.1171	0.2394	370	782	4.6%	1.12 [0.70, 1.80]
West 2014	-0.0865	0.0586	7967	7779	77.2%	0.92 [0.82, 1.03]
**Total (95% CI)**			**12227**	**12461**	**100.0%**	**0.94 [0.85, 1.04]**

Heterogeneity: Chi² = 2.46, df = 6 (P = 0.87); I² = 0%
Test for overall effect: Z = 1.21 (P = 0.23)

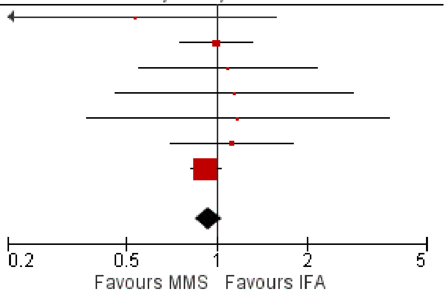

Footnotes
(1) Only the Usual Invitation to Food Supplementation groups were included. Both IFA arms were merged and compared with the MMS arm

Random

Study or Subgroup	log[Risk Ratio]	SE	MMS Total	IFA Total	Weight	Risk Ratio IV, Random, 95% CI
Adu-Afarwuah 2015	-0.6278	0.5526	357	343	0.9%	0.53 [0.18, 1.58]
Fawzi 2007	-0.0028	0.1419	2897	2889	13.2%	1.00 [0.76, 1.32]
Moore 2009	0.0878	0.35	179	171	2.2%	1.09 [0.55, 2.17]
Roberfroid 2008	0.1315	0.4649	306	349	1.2%	1.14 [0.46, 2.84]
Sunawang 2009	0.1623	0.5944	151	148	0.8%	1.18 [0.37, 3.77]
Tofail 2008 (1)	0.1171	0.2394	370	782	4.6%	1.12 [0.70, 1.80]
West 2014	-0.0865	0.0586	7967	7779	77.2%	0.92 [0.82, 1.03]
**Total (95% CI)**			**12227**	**12461**	**100.0%**	**0.94 [0.85, 1.04]**

Heterogeneity: Tau² = 0.00; Chi² = 2.46, df = 6 (P = 0.87); I² = 0%
Test for overall effect: Z = 1.21 (P = 0.23)

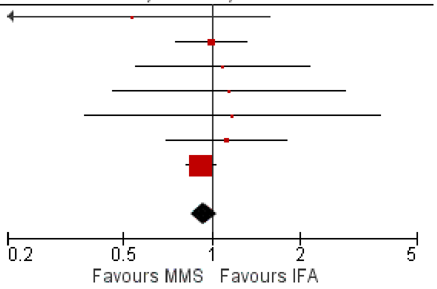

Footnotes
(1) Only the Usual Invitation to Food Supplementation groups were included. Both IFA arms were merged and compared with the MMS arm

Supplemental Figure 2.12.3 Effect of MMS vs IFA on wasting at 6 months

Fixed

Study or Subgroup	log[Risk Ratio]	SE	MMS Total	IFA Total	Weight	Risk Ratio IV, Fixed, 95% CI
Adu-Afarwuah 2015	0.046	0.2471	270	313	4.9%	1.05 [0.65, 1.70]
Ashorn 2010 complete FU (1)	0.8387	0.5932	213	219	0.9%	2.31 [0.72, 7.40]
Ashorn 2010 simplified FU (2)	0.6855	1.2185	131	130	0.2%	1.98 [0.18, 21.62]
Fawzi 2007	-0.0005	0.1284	2998	2971	18.2%	1.00 [0.78, 1.29]
Moore 2009	0.1354	0.3261	169	172	2.8%	1.14 [0.60, 2.17]
Roberfroid 2008	0.1404	0.2477	270	344	4.9%	1.15 [0.71, 1.87]
Sunawang 2009	-0.0201	0.6218	151	148	0.8%	0.98 [0.29, 3.32]
Tofail 2008 (3)	-0.2545	0.2389	445	885	5.3%	0.78 [0.49, 1.24]
West 2014	0.0591	0.0696	4503	4448	62.0%	1.06 [0.93, 1.22]
**Total (95% CI)**			**9150**	**9630**	**100.0%**	**1.05 [0.94, 1.16]**

Heterogeneity: Chi² = 4.04, df = 8 (P = 0.85); I² = 0%
Test for overall effect: Z = 0.81 (P = 0.42)

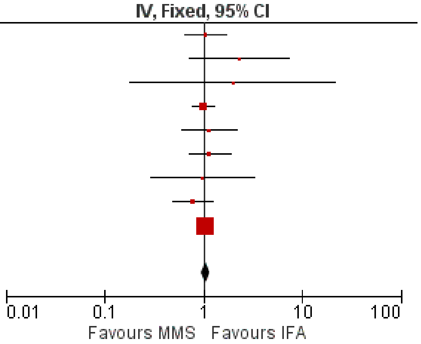

Footnotes
(1) intervention continued daily MMS for 6m PP; control received daily 200mg Ca for 6m PP
(2) intervention and control stopped at delivery and there were less follow-ups
(3) Only the Usual Invitation to Food Supplementation groups were included. Both IFA arms were merged and compared with the MMS arm

Random

Study or Subgroup	log[Risk Ratio]	SE	MMS Total	IFA Total	Weight	Risk Ratio IV, Random, 95% CI
Adu-Afarwuah 2015	0.046	0.2471	270	313	4.9%	1.05 [0.65, 1.70]
Ashorn 2010 complete FU (1)	0.8387	0.5932	213	219	0.9%	2.31 [0.72, 7.40]
Ashorn 2010 simplified FU (2)	0.6855	1.2185	131	130	0.2%	1.98 [0.18, 21.62]
Fawzi 2007	-0.0005	0.1284	2998	2971	18.2%	1.00 [0.78, 1.29]
Moore 2009	0.1354	0.3261	169	172	2.8%	1.14 [0.60, 2.17]
Roberfroid 2008	0.1404	0.2477	270	344	4.9%	1.15 [0.71, 1.87]
Sunawang 2009	-0.0201	0.6218	151	148	0.8%	0.98 [0.29, 3.32]
Tofail 2008 (3)	-0.2545	0.2389	445	885	5.3%	0.78 [0.49, 1.24]
West 2014	0.0591	0.0696	4503	4448	62.0%	1.06 [0.93, 1.22]
**Total (95% CI)**			**9150**	**9630**	**100.0%**	**1.05 [0.94, 1.16]**

Heterogeneity: Tau² = 0.00; Chi² = 4.04, df = 8 (P = 0.85); I² = 0%
Test for overall effect: Z = 0.81 (P = 0.42)

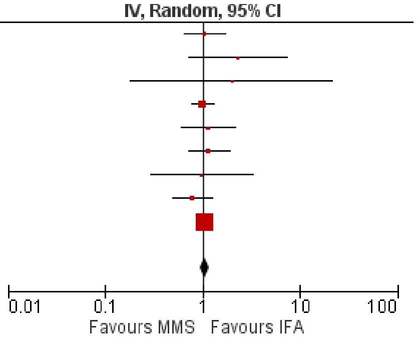

Footnotes
(1) intervention continued daily MMS for 6m PP; control received daily 200mg Ca for 6m PP
(2) intervention and control stopped at delivery and there were less follow-ups
(3) Only the Usual Invitation to Food Supplementation groups were included. Both IFA arms were merged and compared with the MMS arm

Supplemental Figure 2.12.4 Effect of MMS vs IFA on wasting at 12 months

Fixed

Study or Subgroup	log[Risk Ratio]	SE	MMS Total	IFA Total	Weight	Risk Ratio IV, Fixed, 95% CI
Adu-Afarwuah 2015	-0.4439	0.2521	345	332	2.3%	0.64 [0.39, 1.05]
Ashorn 2010 complete FU (1)	0.2698	0.4947	214	218	0.6%	1.31 [0.50, 3.45]
Fawzi 2007	-0.0399	0.1292	1628	1607	8.8%	0.96 [0.75, 1.24]
Roberfroid 2008	-0.0233	0.1709	240	248	5.0%	0.98 [0.70, 1.37]
Sunawang 2009 (2)	0	0	151	148		Not estimable
Tofail 2008 (3)	-0.0389	0.1483	502	1023	6.7%	0.96 [0.72, 1.29]
West 2014	-0.0229	0.0439	4856	4785	76.5%	0.98 [0.90, 1.07]
**Total (95% CI)**			**7936**	**8361**	**100.0%**	**0.97 [0.90, 1.04]**

Heterogeneity: Chi² = 3.09, df = 5 (P = 0.69); I² = 0%
Test for overall effect: Z = 0.87 (P = 0.38)

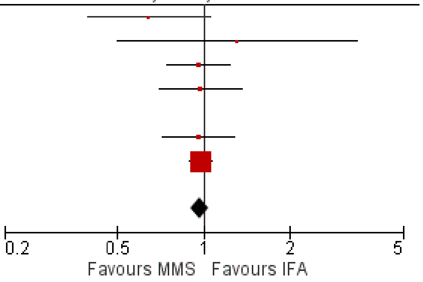

Footnotes
(1) intervention continued daily MMS for 6m PP; control received daily 200mg Ca for 6m PP
(2) Children were followed up to 12m, but there are no cases of wasting at 12m
(3) Only the Usual Invitation to Food Supplementation groups were included. Both IFA arms were merged and compared with the MMS arm

Random

Study or Subgroup	log[Risk Ratio]	SE	MMS Total	IFA Total	Weight	Risk Ratio IV, Random, 95% CI
Adu-Afarwuah 2015	-0.4439	0.2521	345	332	2.3%	0.64 [0.39, 1.05]
Ashorn 2010 complete FU (1)	0.2698	0.4947	214	218	0.6%	1.31 [0.50, 3.45]
Fawzi 2007	-0.0399	0.1292	1628	1607	8.8%	0.96 [0.75, 1.24]
Roberfroid 2008	-0.0233	0.1709	240	248	5.0%	0.98 [0.70, 1.37]
Sunawang 2009 (2)	0	0	151	148		Not estimable
Tofail 2008 (3)	-0.0389	0.1483	502	1023	6.7%	0.96 [0.72, 1.29]
West 2014	-0.0229	0.0439	4856	4785	76.5%	0.98 [0.90, 1.07]
**Total (95% CI)**			**7936**	**8361**	**100.0%**	**0.97 [0.90, 1.04]**

Heterogeneity: Tau² = 0.00; Chi² = 3.09, df = 5 (P = 0.69); I² = 0%
Test for overall effect: Z = 0.87 (P = 0.38)

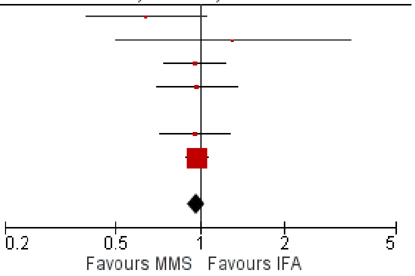

Footnotes
(1) intervention continued daily MMS for 6m PP; control received daily 200mg Ca for 6m PP
(2) Children were followed up to 12m, but there are no cases of wasting at 12m
(3) Only the Usual Invitation to Food Supplementation groups were included. Both IFA arms were merged and compared with the MMS arm

Supplemental Figure 2.12.5 Effect of MMS vs IFA on wasting at 18 months

Fixed

Study or Subgroup	log[Risk Ratio]	SE	MMS Total	IFA Total	Weight	Risk Ratio IV, Fixed, 95% CI
Adu-Afarwuah 2015	-0.4415	0.2722	355	337	18.9%	0.64 [0.38, 1.10]
Ashorn 2010 complete FU (1)	-0.1426	0.5087	222	220	5.4%	0.87 [0.32, 2.35]
Ashorn 2010 simplified FU (2)	-0.1901	0.5925	129	128	4.0%	0.83 [0.26, 2.64]
Fawzi 2007	-0.4672	0.5633	39	55	4.4%	0.63 [0.21, 1.89]
Tofail 2008 (3)	0.0266	0.1443	463	974	67.3%	1.03 [0.77, 1.36]
**Total (95% CI)**			**1208**	**1714**	**100.0%**	**0.90 [0.72, 1.14]**

Heterogeneity: Chi² = 2.80, df = 4 (P = 0.59); I² = 0%
Test for overall effect: Z = 0.86 (P = 0.39)

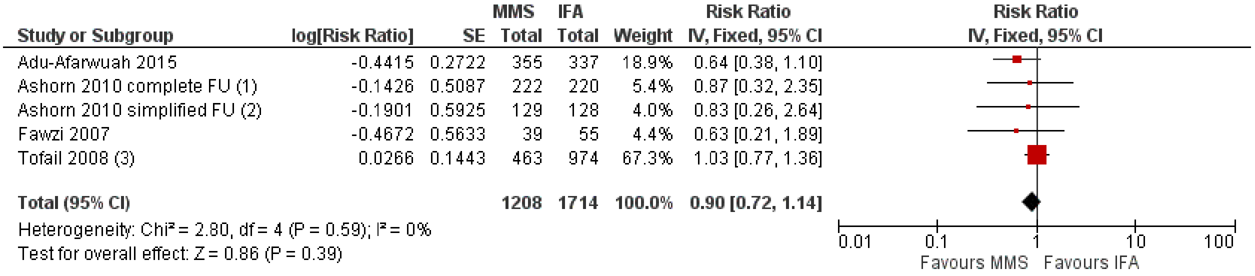

Footnotes
(1) intervention continued daily MMS for 6m PP; control received daily 200mg Ca for 6m PP
(2) intervention and control stopped at delivery and there were less follow-ups
(3) Only the Usual Invitation to Food Supplementation groups were included. Both IFA arms were merged and compared with the MMS arm

Random

Study or Subgroup	log[Risk Ratio]	SE	MMS Total	IFA Total	Weight	Risk Ratio IV, Random, 95% CI
Adu-Afarwuah 2015	-0.4415	0.2722	355	337	18.9%	0.64 [0.38, 1.10]
Ashorn 2010 complete FU (1)	-0.1426	0.5087	222	220	5.4%	0.87 [0.32, 2.35]
Ashorn 2010 simplified FU (2)	-0.1901	0.5925	129	128	4.0%	0.83 [0.26, 2.64]
Fawzi 2007	-0.4672	0.5633	39	55	4.4%	0.63 [0.21, 1.89]
Tofail 2008 (3)	0.0266	0.1443	463	974	67.3%	1.03 [0.77, 1.36]
**Total (95% CI)**			**1208**	**1714**	**100.0%**	**0.90 [0.72, 1.14]**

Heterogeneity: Tau² = 0.00; Chi² = 2.80, df = 4 (P = 0.59); I² = 0%
Test for overall effect: Z = 0.86 (P = 0.39)

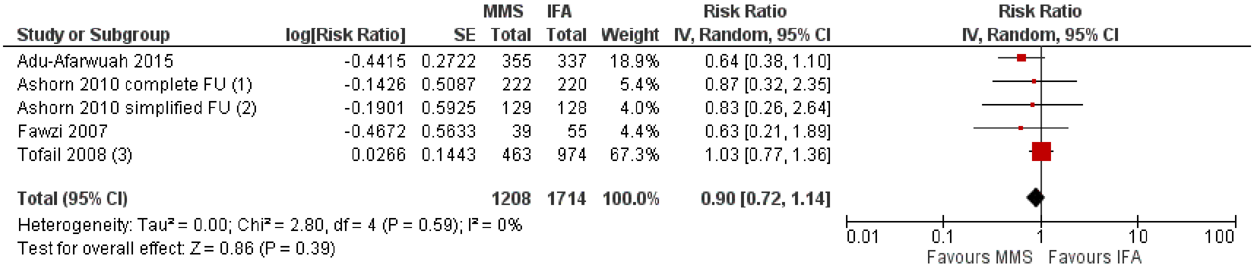

Footnotes
(1) intervention continued daily MMS for 6m PP; control received daily 200mg Ca for 6m PP
(2) intervention and control stopped at delivery and there were less follow-ups
(3) Only the Usual Invitation to Food Supplementation groups were included. Both IFA arms were merged and compared with the MMS arm

Supplemental Figure 2.12.6 Effect of MMS vs IFA on wasting at 24 months

Fixed

Study or Subgroup	log[Risk Ratio]	SE	MMS Total	IFA Total	Weight	Risk Ratio IV, Fixed, 95% CI
Ashorn 2010 complete FU (1)	0.2254	0.4747	176	196	0.8%	1.25 [0.49, 3.18]
Bliznashka 2021	0.1133	0.2326	642	718	3.4%	1.12 [0.71, 1.77]
Roberfroid 2008	0.1201	0.1768	54	55	5.8%	1.13 [0.80, 1.59]
Tofail 2008 (2)	-0.1368	0.153	479	967	7.8%	0.87 [0.65, 1.18]
West 2014	0.0039	0.047	3285	3238	82.3%	1.00 [0.92, 1.10]
**Total (95% CI)**			**4636**	**5174**	**100.0%**	**1.01 [0.92, 1.09]**

Heterogeneity: Chi² = 1.72, df = 4 (P = 0.79); I² = 0%
Test for overall effect: Z = 0.12 (P = 0.90)

Risk Ratio IV, Fixed, 95% CI

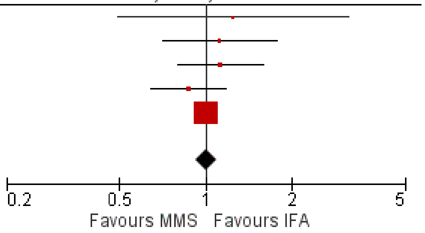

Footnotes
(1) intervention continued daily MMS for 6m PP; control received daily 200mg Ca for 6m PP
(2) Only the Usual Invitation to Food Supplementation groups were included. Both IFA arms were merged and compared with the MMS arm

Random

Study or Subgroup	log[Risk Ratio]	SE	MMS Total	IFA Total	Weight	Risk Ratio IV, Random, 95% CI
Ashorn 2010 complete FU (1)	0.2254	0.4747	176	196	0.8%	1.25 [0.49, 3.18]
Bliznashka 2021	0.1133	0.2326	642	718	3.4%	1.12 [0.71, 1.77]
Roberfroid 2008	0.1201	0.1768	54	55	5.8%	1.13 [0.80, 1.59]
Tofail 2008 (2)	-0.1368	0.153	479	967	7.8%	0.87 [0.65, 1.18]
West 2014	0.0039	0.047	3285	3238	82.3%	1.00 [0.92, 1.10]
**Total (95% CI)**			**4636**	**5174**	**100.0%**	**1.01 [0.92, 1.09]**

Heterogeneity: Tau² = 0.00; Chi² = 1.72, df = 4 (P = 0.79); I² = 0%
Test for overall effect: Z = 0.12 (P = 0.90)

Risk Ratio IV, Random, 95% CI

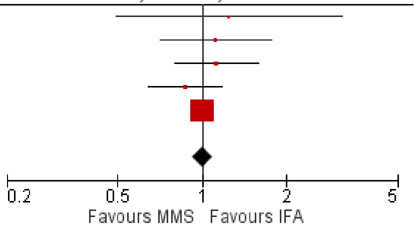

Footnotes
(1) intervention continued daily MMS for 6m PP; control received daily 200mg Ca for 6m PP
(2) Only the Usual Invitation to Food Supplementation groups were included. Both IFA arms were merged and compared with the MMS arm

Supplemental Figure 2.13. Effect of MMS vs IFA on small HC

Supplemental Figure 2.13.1. Effect of MMS vs IFA on small HC at birth

Fixed

Study or Subgroup	log[Risk Ratio]	SE	MMS Total	IFA Total	Weight	Risk Ratio IV, Fixed, 95% CI
Adu-Afarwuah 2015	-0.3442	0.3096	317	304	0.6%	0.71 [0.39, 1.30]
Ashorn 2010	-0.6794	0.3648	372	360	0.4%	0.51 [0.25, 1.04]
Fawzi 2007	-0.1835	0.091	3332	3339	7.2%	0.83 [0.70, 0.99]
Osrin 2005	-0.2278	0.1883	526	519	1.7%	0.80 [0.55, 1.15]
Roberfroid 2008	0.0902	0.2126	534	539	1.3%	1.09 [0.72, 1.66]
Sunawang 2009	-0.1238	0.1569	630	648	2.4%	0.88 [0.65, 1.20]
Tofail 2008	0.06	0.0788	490	1012	9.6%	1.06 [0.91, 1.24]
West 2014	-0.148	0.0279	7062	6963	76.7%	0.86 [0.82, 0.91]
**Total (95% CI)**			**13263**	**13684**	**100.0%**	**0.88 [0.84, 0.92]**

Heterogeneity: Chi² = 10.66, df = 7 (P = 0.15); I² = 34%
Test for overall effect: Z = 5.39 (P < 0.00001)

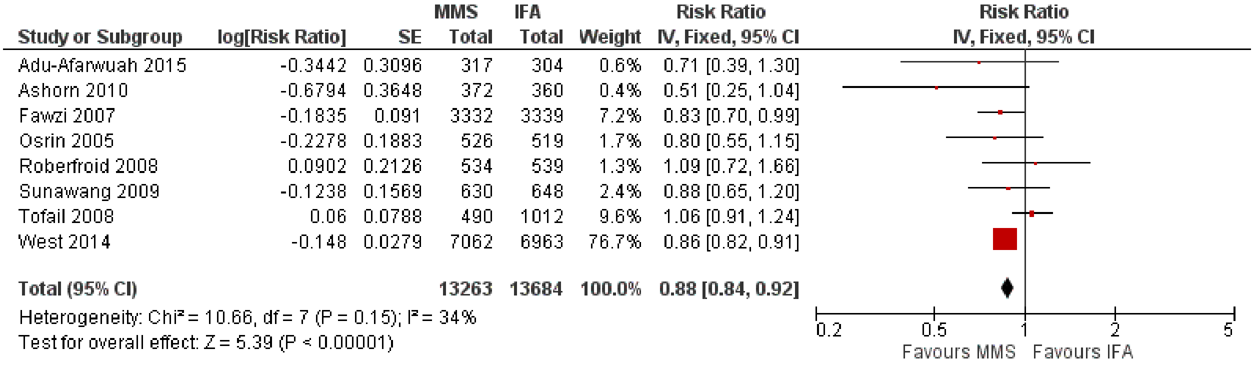

Random

Study or Subgroup	log[Risk Ratio]	SE	MMS Total	IFA Total	Weight	Risk Ratio IV, Random, 95% CI
Adu-Afarwuah 2015	-0.3442	0.3096	317	304	2.4%	0.71 [0.39, 1.30]
Ashorn 2010	-0.6794	0.3648	372	360	1.8%	0.51 [0.25, 1.04]
Fawzi 2007	-0.1835	0.091	3332	3339	17.6%	0.83 [0.70, 0.99]
Osrin 2005	-0.2278	0.1883	526	519	5.9%	0.80 [0.55, 1.15]
Roberfroid 2008	0.0902	0.2126	534	539	4.8%	1.09 [0.72, 1.66]
Sunawang 2009	-0.1238	0.1569	630	648	8.1%	0.88 [0.65, 1.20]
Tofail 2008	0.06	0.0788	490	1012	20.7%	1.06 [0.91, 1.24]
West 2014	-0.148	0.0279	7062	6963	38.7%	0.86 [0.82, 0.91]
**Total (95% CI)**			**13263**	**13684**	**100.0%**	**0.89 [0.81, 0.98]**

Heterogeneity: Tau² = 0.01; Chi² = 10.66, df = 7 (P = 0.15); I² = 34%
Test for overall effect: Z = 2.36 (P = 0.02)

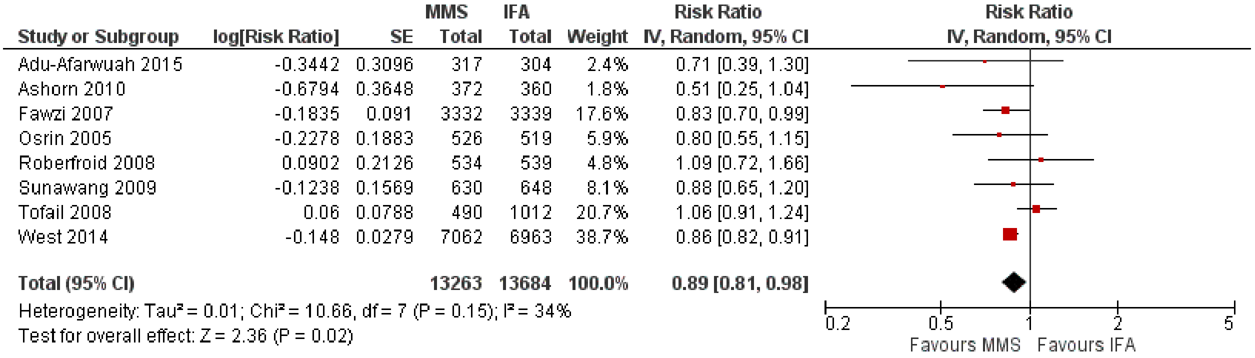

Supplemental Figure 2.13.2 Effect of MMS vs IFA on small HC at 3 months

Fixed

Study or Subgroup	log[Risk Ratio]	SE	MMS Total	IFA Total	Weight	Risk Ratio IV, Fixed, 95% CI
Adu-Afarwuah 2015	-0.6847	0.2657	358	343	1.5%	0.50 [0.30, 0.85]
Fawzi 2007	0.2499	0.1931	2969	2986	2.9%	1.28 [0.88, 1.87]
Moore 2009	-0.1622	0.4469	181	171	0.5%	0.85 [0.35, 2.04]
Roberfroid 2008	-0.205	0.3289	306	349	1.0%	0.81 [0.43, 1.55]
Tofail 2008	0.03	0.1078	376	787	9.4%	1.03 [0.83, 1.27]
West 2014	-0.2015	0.0359	8055	7839	84.6%	0.82 [0.76, 0.88]
**Total (95% CI)**			**12245**	**12475**	**100.0%**	**0.84 [0.79, 0.90]**

Heterogeneity: Chi² = 12.69, df = 5 (P = 0.03); I² = 61%
Test for overall effect: Z = 5.27 (P < 0.00001)

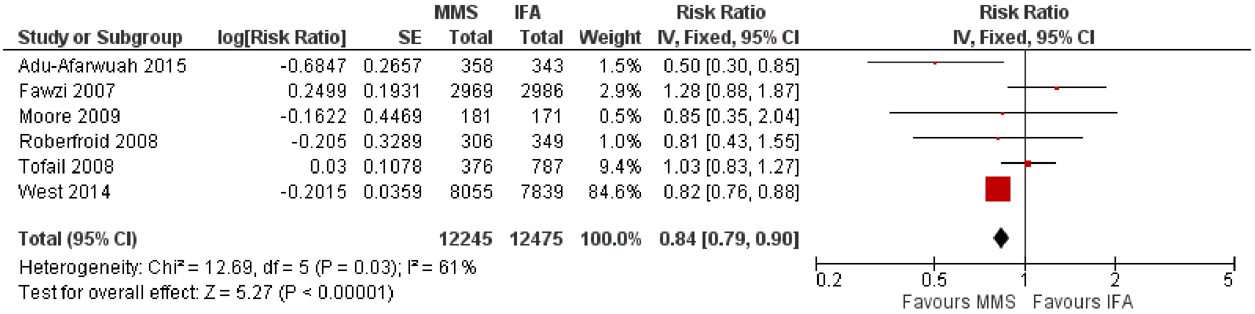

Random

Study or Subgroup	log[Risk Ratio]	SE	MMS Total	IFA Total	Weight	Risk Ratio IV, Random, 95% CI
Adu-Afarwuah 2015	-0.6847	0.2657	358	343	10.8%	0.50 [0.30, 0.85]
Fawzi 2007	0.2499	0.1931	2969	2986	16.1%	1.28 [0.88, 1.87]
Moore 2009	-0.1622	0.4469	181	171	4.8%	0.85 [0.35, 2.04]
Roberfroid 2008	-0.205	0.3289	306	349	7.9%	0.81 [0.43, 1.55]
Tofail 2008	0.03	0.1078	376	787	26.0%	1.03 [0.83, 1.27]
West 2014	-0.2015	0.0359	8055	7839	34.4%	0.82 [0.76, 0.88]
**Total (95% CI)**			**12245**	**12475**	**100.0%**	**0.89 [0.72, 1.09]**

Heterogeneity: Tau² = 0.03; Chi² = 12.69, df = 5 (P = 0.03); I² = 61%
Test for overall effect: Z = 1.14 (P = 0.25)

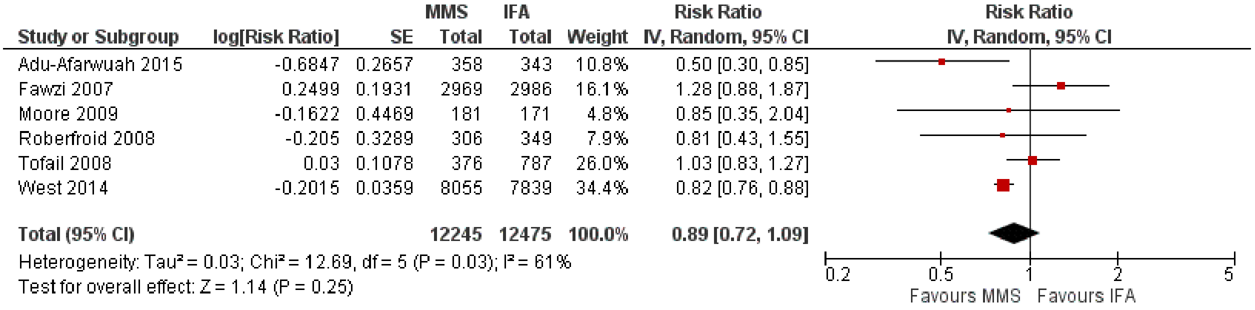

Supplemental Figure 2.13.3 Effect of MMS vs IFA on small HC at 6 months

Fixed

Study or Subgroup	log[Risk Ratio]	SE	MMS Total	IFA Total	Weight	Risk Ratio IV, Fixed, 95% CI
Adu-Afarwuah 2015	-0.1508	0.2389	356	347	2.5%	0.86 [0.54, 1.37]
Ashorn 2010 complete FU	0.2969	0.4304	216	218	0.8%	1.35 [0.58, 3.13]
Ashorn 2010 simplified FU	0.3978	0.4393	131	130	0.7%	1.49 [0.63, 3.52]
Fawzi 2007	0.2323	0.1677	3026	2996	5.1%	1.26 [0.91, 1.75]
Moore 2009	-0.2566	0.3522	170	172	1.2%	0.77 [0.39, 1.54]
Roberfroid 2008	0.0937	0.3183	270	313	1.4%	1.10 [0.59, 2.05]
Tofail 2008	-0.0067	0.0854	449	892	19.7%	0.99 [0.84, 1.17]
West 2014	-0.0395	0.0457	4559	4489	68.6%	0.96 [0.88, 1.05]
**Total (95% CI)**			**9177**	**9557**	**100.0%**	**0.98 [0.91, 1.06]**

Heterogeneity: Chi² = 4.79, df = 7 (P = 0.69); I² = 0%
Test for overall effect: Z = 0.44 (P = 0.66)

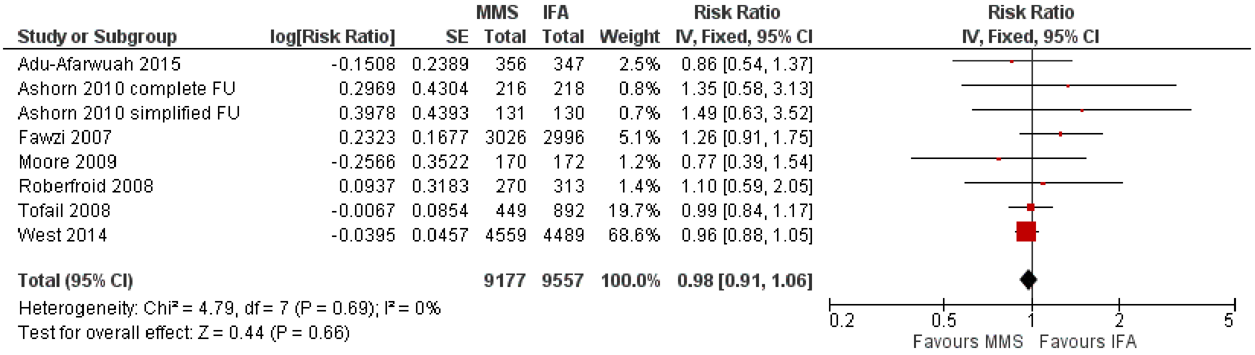

Random

Study or Subgroup	log[Risk Ratio]	SE	MMS Total	IFA Total	Weight	Risk Ratio IV, Random, 95% CI
Adu-Afarwuah 2015	-0.1508	0.2389	356	347	2.5%	0.86 [0.54, 1.37]
Ashorn 2010 complete FU	0.2969	0.4304	216	218	0.8%	1.35 [0.58, 3.13]
Ashorn 2010 simplified FU	0.3978	0.4393	131	130	0.7%	1.49 [0.63, 3.52]
Fawzi 2007	0.2323	0.1677	3026	2996	5.1%	1.26 [0.91, 1.75]
Moore 2009	-0.2566	0.3522	170	172	1.2%	0.77 [0.39, 1.54]
Roberfroid 2008	0.0937	0.3183	270	313	1.4%	1.10 [0.59, 2.05]
Tofail 2008	-0.0067	0.0854	449	892	19.7%	0.99 [0.84, 1.17]
West 2014	-0.0395	0.0457	4559	4489	68.6%	0.96 [0.88, 1.05]
**Total (95% CI)**			**9177**	**9557**	**100.0%**	**0.98 [0.91, 1.06]**

Heterogeneity: Tau² = 0.00; Chi² = 4.79, df = 7 (P = 0.69); I² = 0%
Test for overall effect: Z = 0.44 (P = 0.66)

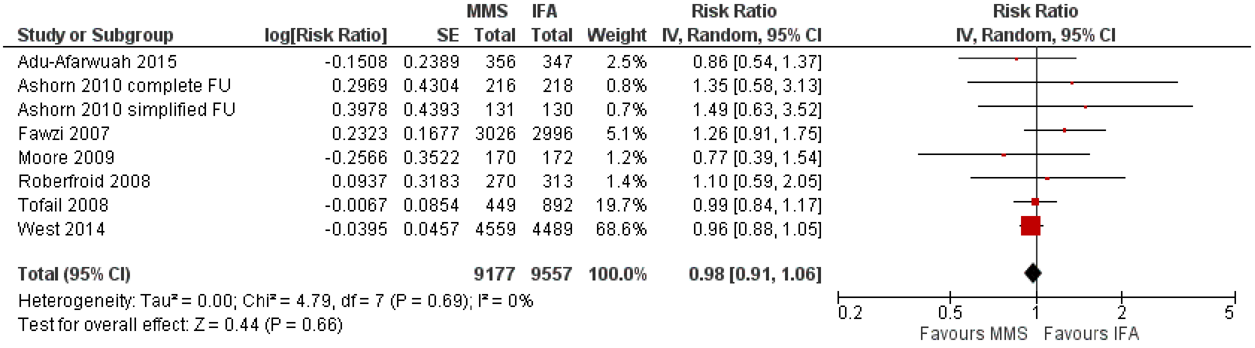

Supplemental Figure 2.13.4 Effect of MMS vs IFA on small HC at 12 months

Fixed

Study or Subgroup	log[Risk Ratio]	SE	MMS Total	IFA Total	Weight	Risk Ratio IV, Fixed, 95% CI
Adu-Afarwuah 2015	-0.2691	0.1894	346	332	2.1%	0.76 [0.53, 1.11]
Ashorn 2010 complete FU	0.3207	0.3051	215	219	0.8%	1.38 [0.76, 2.51]
Fawzi 2007	0.0746	0.1833	1620	1600	2.3%	1.08 [0.75, 1.54]
Roberfroid 2008	-0.1419	0.2149	240	245	1.7%	0.87 [0.57, 1.32]
Tofail 2008	0.0269	0.0606	505	1033	20.8%	1.03 [0.91, 1.16]
West 2014	-0.0543	0.0325	4945	4862	72.3%	0.95 [0.89, 1.01]
**Total (95% CI)**			**7871**	**8291**	**100.0%**	**0.96 [0.91, 1.02]**

Heterogeneity: Chi² = 4.88, df = 5 (P = 0.43); I² = 0%
Test for overall effect: Z = 1.35 (P = 0.18)

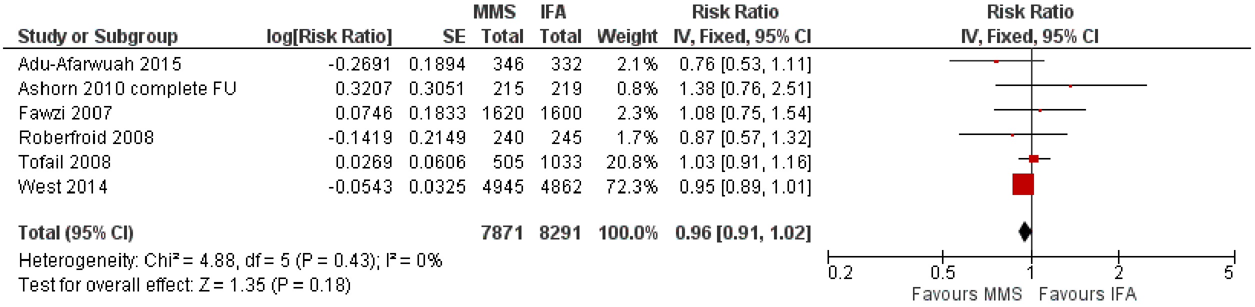

Random

Study or Subgroup	log[Risk Ratio]	SE	MMS Total	IFA Total	Weight	Risk Ratio IV, Random, 95% CI
Adu-Afarwuah 2015	-0.2691	0.1894	346	332	2.1%	0.76 [0.53, 1.11]
Ashorn 2010 complete FU	0.3207	0.3051	215	219	0.8%	1.38 [0.76, 2.51]
Fawzi 2007	0.0746	0.1833	1620	1600	2.3%	1.08 [0.75, 1.54]
Roberfroid 2008	-0.1419	0.2149	240	245	1.7%	0.87 [0.57, 1.32]
Tofail 2008	0.0269	0.0606	505	1033	20.8%	1.03 [0.91, 1.16]
West 2014	-0.0543	0.0325	4945	4862	72.3%	0.95 [0.89, 1.01]
**Total (95% CI)**			**7871**	**8291**	**100.0%**	**0.96 [0.91, 1.02]**

Heterogeneity: Tau² = 0.00; Chi² = 4.88, df = 5 (P = 0.43); I² = 0%
Test for overall effect: Z = 1.35 (P = 0.18)

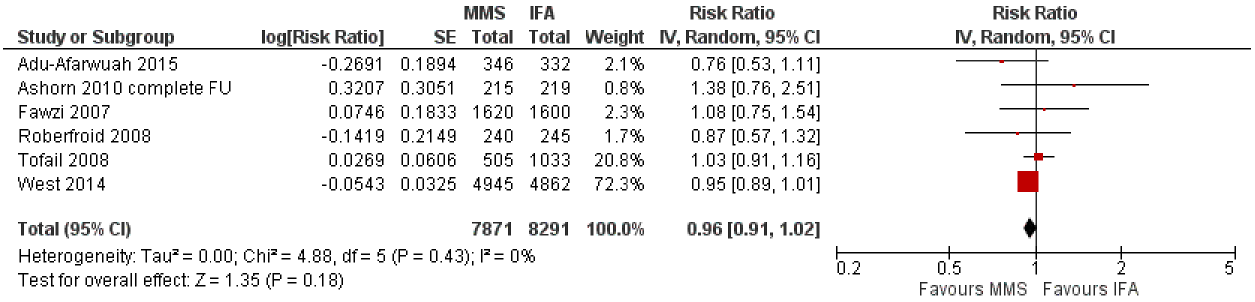

Supplemental Figure 2.13.5 Effect of MMS vs IFA on small HC at 18 months

Fixed

Study or Subgroup	log[Risk Ratio]	SE	MMS Total	IFA Total	Weight	Risk Ratio IV, Fixed, 95% CI
Adu-Afarwuah 2015	-0.2344	0.1757	355	337	60.7%	0.79 [0.56, 1.12]
Ashorn 2010 complete FU	0.2045	0.2775	222	220	24.4%	1.23 [0.71, 2.11]
Ashorn 2010 simplified FU	-0.2309	0.3668	130	129	13.9%	0.79 [0.39, 1.63]
Fawzi 2007	0.3438	1.3986	39	55	1.0%	1.41 [0.09, 21.87]
**Total (95% CI)**			**746**	**741**	**100.0%**	**0.89 [0.68, 1.16]**

Heterogeneity: Chi² = 1.99, df = 3 (P = 0.57); I² = 0%
Test for overall effect: Z = 0.89 (P = 0.38)

Risk Ratio
IV, Fixed, 95% CI

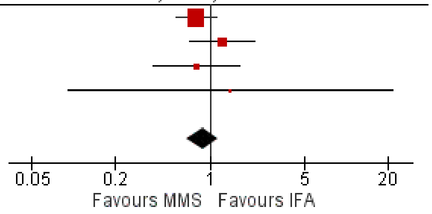

Random

Study or Subgroup	log[Risk Ratio]	SE	MMS Total	IFA Total	Weight	Risk Ratio IV, Random, 95% CI
Adu-Afarwuah 2015	-0.2344	0.1757	355	337	60.7%	0.79 [0.56, 1.12]
Ashorn 2010 complete FU	0.2045	0.2775	222	220	24.4%	1.23 [0.71, 2.11]
Ashorn 2010 simplified FU	-0.2309	0.3668	130	129	13.9%	0.79 [0.39, 1.63]
Fawzi 2007	0.3438	1.3986	39	55	1.0%	1.41 [0.09, 21.87]
**Total (95% CI)**			**746**	**741**	**100.0%**	**0.89 [0.68, 1.16]**

Heterogeneity: Tau² = 0.00; Chi² = 1.99, df = 3 (P = 0.57); I² = 0%
Test for overall effect: Z = 0.89 (P = 0.38)

Risk Ratio
IV, Random, 95% CI

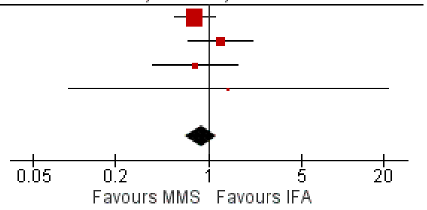

Supplemental Figure 2.13.6 Effect of MMS vs IFA on small HC at 24 months

Fixed

Study or Subgroup	log[Risk Ratio]	SE	MMS Total	IFA Total	Weight	Risk Ratio IV, Fixed, 95% CI
Ashorn 2010 complete FU	0.1699	0.252	179	197	1.8%	1.19 [0.72, 1.94]
West 2014	-0.0061	0.0338	3405	3355	98.2%	0.99 [0.93, 1.06]
**Total (95% CI)**			**3584**	**3552**	**100.0%**	**1.00 [0.93, 1.06]**

Heterogeneity: Chi² = 0.48, df = 1 (P = 0.49); I² = 0%
Test for overall effect: Z = 0.09 (P = 0.93)

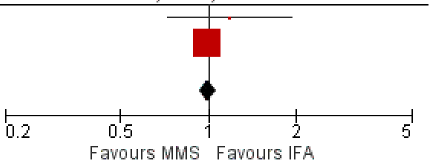

Random

Study or Subgroup	log[Risk Ratio]	SE	MMS Total	IFA Total	Weight	Risk Ratio IV, Random, 95% CI
Ashorn 2010 complete FU	0.1699	0.252	179	197	1.8%	1.19 [0.72, 1.94]
West 2014	-0.0061	0.0338	3405	3355	98.2%	0.99 [0.93, 1.06]
**Total (95% CI)**			**3584**	**3552**	**100.0%**	**1.00 [0.93, 1.06]**

Heterogeneity: Tau² = 0.00; Chi² = 0.48, df = 1 (P = 0.49); I² = 0%
Test for overall effect: Z = 0.09 (P = 0.93)

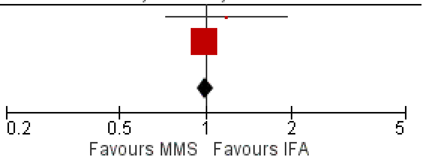

Supplemental Figure 2.14. Effect of MMS vs IFA on low MUAC

Supplemental Figure 2.14.1 Effect of MMS vs IFA on low MUAC at 3 months

Fixed

Study or Subgroup	log[Risk Ratio]	SE	MMS Total	IFA Total	Weight	Risk Ratio IV, Fixed, 95% CI
Adu-Afarwuah 2015	-0.9424	0.5265	340	318	0.8%	0.39 [0.14, 1.09]
Fawzi 2007	-0.1751	0.2322	1431	1456	4.2%	0.84 [0.53, 1.32]
Moore 2009	0.0263	1.4096	150	154	0.1%	1.03 [0.06, 16.27]
Roberfroid 2008	-0.274	0.6407	306	349	0.5%	0.76 [0.22, 2.67]
Sunawang 2009	-0.0201	0.8083	151	148	0.3%	0.98 [0.20, 4.78]
West 2014	-0.0952	0.0488	8072	7858	94.0%	0.91 [0.83, 1.00]
**Total (95% CI)**			**10450**	**10283**	**100.0%**	**0.90 [0.82, 0.99]**

Heterogeneity: Chi² = 2.75, df = 5 (P = 0.74); I² = 0%
Test for overall effect: Z = 2.24 (P = 0.03)

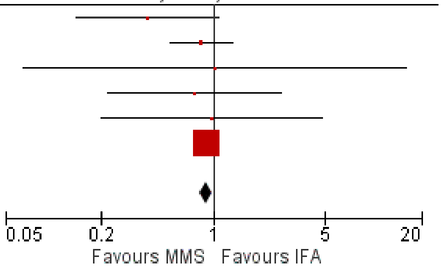

Random

Study or Subgroup	log[Risk Ratio]	SE	MMS Total	IFA Total	Weight	Risk Ratio IV, Random, 95% CI
Adu-Afarwuah 2015	-0.9424	0.5265	340	318	0.8%	0.39 [0.14, 1.09]
Fawzi 2007	-0.1751	0.2322	1431	1456	4.2%	0.84 [0.53, 1.32]
Moore 2009	0.0263	1.4096	150	154	0.1%	1.03 [0.06, 16.27]
Roberfroid 2008	-0.274	0.6407	306	349	0.5%	0.76 [0.22, 2.67]
Sunawang 2009	-0.0201	0.8083	151	148	0.3%	0.98 [0.20, 4.78]
West 2014	-0.0952	0.0488	8072	7858	94.0%	0.91 [0.83, 1.00]
**Total (95% CI)**			**10450**	**10283**	**100.0%**	**0.90 [0.82, 0.99]**

Heterogeneity: Tau² = 0.00; Chi² = 2.75, df = 5 (P = 0.74); I² = 0%
Test for overall effect: Z = 2.24 (P = 0.03)

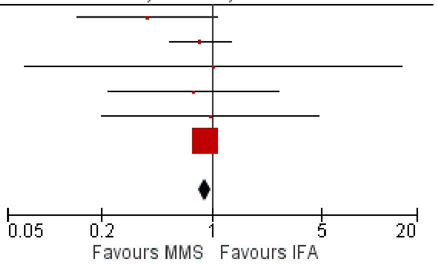

Supplemental Figure 2.14.2 Effect of MMS vs IFA on low MUAC at 6 months

Fixed

Study or Subgroup	log[Risk Ratio]	SE	MMS Total	IFA Total	Weight	Risk Ratio IV, Fixed, 95% CI
Adu-Afarwuah 2015	-0.1898	0.4027	356	348	2.3%	0.83 [0.38, 1.82]
Ashorn 2010 complete FU	0.2415	0.4646	216	220	1.7%	1.27 [0.51, 3.16]
Ashorn 2010 simplified FU (1)	1.3786	1.1112	131	130	0.3%	3.97 [0.45, 35.04]
Fawzi 2007	0.1262	0.168	1852	1842	13.2%	1.13 [0.82, 1.58]
Moore 2009	-1.0869	0.8093	170	172	0.6%	0.34 [0.07, 1.65]
Roberfroid 2008	0.0788	0.3622	270	313	2.8%	1.08 [0.53, 2.20]
Sunawang 2009	-0.0201	0.9933	151	148	0.4%	0.98 [0.14, 6.87]
West 2014	-0.035	0.0688	4564	4499	78.7%	0.97 [0.84, 1.11]
**Total (95% CI)**			**7710**	**7672**	**100.0%**	**0.99 [0.88, 1.11]**

Heterogeneity: Chi² = 4.67, df = 7 (P = 0.70); I² = 0%
Test for overall effect: Z = 0.18 (P = 0.86)

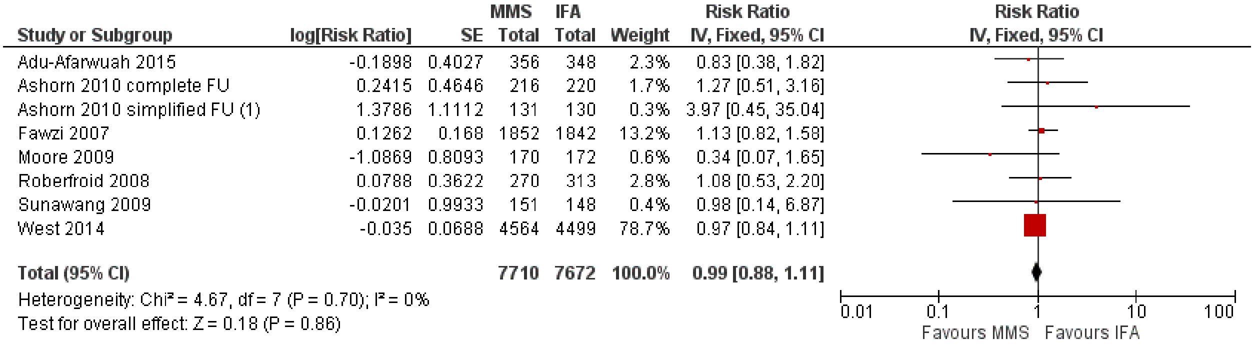

Footnotes
(1) Only 4 events in the MMS arm and 1 event on the IFA arm

Random

Study or Subgroup	log[Risk Ratio]	SE	MMS Total	IFA Total	Weight	Risk Ratio IV, Random, 95% CI
Adu-Afarwuah 2015	-0.1898	0.4027	356	348	2.3%	0.83 [0.38, 1.82]
Ashorn 2010 complete FU	0.2415	0.4646	216	220	1.7%	1.27 [0.51, 3.16]
Ashorn 2010 simplified FU (1)	1.3786	1.1112	131	130	0.3%	3.97 [0.45, 35.04]
Fawzi 2007	0.1262	0.168	1852	1842	13.2%	1.13 [0.82, 1.58]
Moore 2009	-1.0869	0.8093	170	172	0.6%	0.34 [0.07, 1.65]
Roberfroid 2008	0.0788	0.3622	270	313	2.8%	1.08 [0.53, 2.20]
Sunawang 2009	-0.0201	0.9933	151	148	0.4%	0.98 [0.14, 6.87]
West 2014	-0.035	0.0688	4564	4499	78.7%	0.97 [0.84, 1.11]
**Total (95% CI)**			**7710**	**7672**	**100.0%**	**0.99 [0.88, 1.11]**

Heterogeneity: Tau² = 0.00; Chi² = 4.67, df = 7 (P = 0.70); I² = 0%
Test for overall effect: Z = 0.18 (P = 0.86)

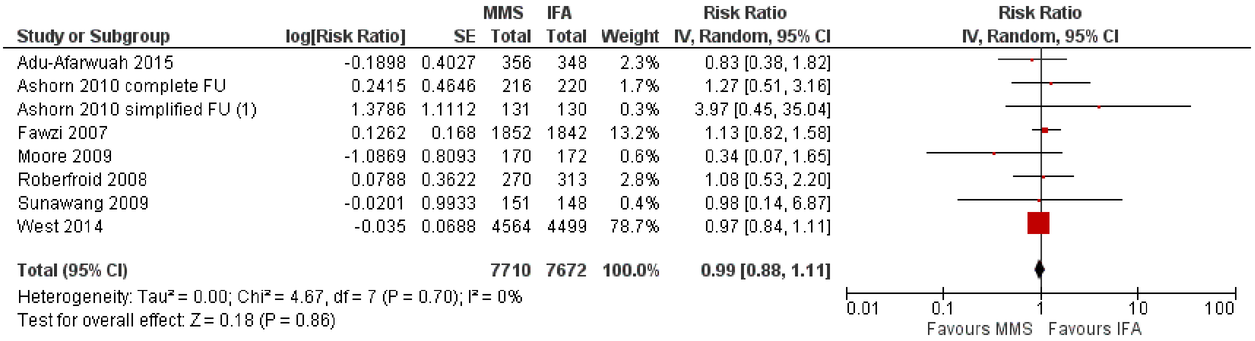

Footnotes
(1) Only 4 events in the MMS arm and 1 event on the IFA arm

Supplemental Figure 2.14.3 Effect of MMS vs IFA on low MUAC at 12 months

Fixed

Study or Subgroup	log[Risk Ratio]	SE	MMS Total	IFA Total	Weight	Risk Ratio IV, Fixed, 95% CI
Adu-Afarwuah 2015	-0.8338	0.394	346	334	1.4%	0.43 [0.20, 0.94]
Ashorn 2010 complete FU (1)	2.7264	1.4574	215	219	0.1%	15.28 [0.88, 265.83]
Fawzi 2007	0.1556	0.1597	1490	1483	8.6%	1.17 [0.85, 1.60]
Roberfroid 2008	-0.0387	0.2516	240	248	3.5%	0.96 [0.59, 1.58]
Sunawang 2009	-0.3157	0.2981	150	148	2.5%	0.73 [0.41, 1.31]
West 2014	-0.0066	0.0512	4998	4913	83.9%	0.99 [0.90, 1.10]
**Total (95% CI)**			**7439**	**7345**	**100.0%**	**0.99 [0.90, 1.09]**

Heterogeneity: Chi² = 10.04, df = 5 (P = 0.07); I² = 50%
Test for overall effect: Z = 0.22 (P = 0.83)

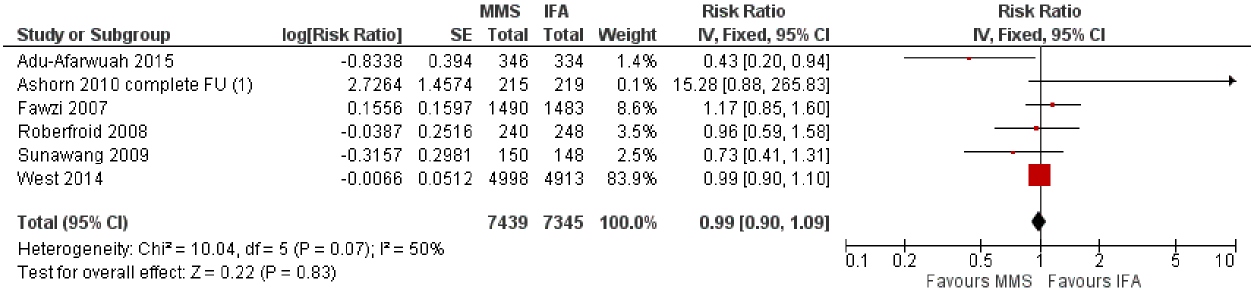

Footnotes
(1) 7 events in the MMS arm and no events in the IFA arm

Random

Study or Subgroup	log[Risk Ratio]	SE	MMS Total	IFA Total	Weight	Risk Ratio IV, Random, 95% CI
Adu-Afarwuah 2015	-0.8338	0.394	346	334	8.2%	0.43 [0.20, 0.94]
Ashorn 2010 complete FU (1)	2.7264	1.4574	215	219	0.7%	15.28 [0.88, 265.83]
Fawzi 2007	0.1556	0.1597	1490	1483	24.7%	1.17 [0.85, 1.60]
Roberfroid 2008	-0.0387	0.2516	240	248	15.6%	0.96 [0.59, 1.58]
Sunawang 2009	-0.3157	0.2981	150	148	12.4%	0.73 [0.41, 1.31]
West 2014	-0.0066	0.0512	4998	4913	38.4%	0.99 [0.90, 1.10]
**Total (95% CI)**			**7439**	**7345**	**100.0%**	**0.94 [0.74, 1.21]**

Heterogeneity: Tau² = 0.04; Chi² = 10.04, df = 5 (P = 0.07); I² = 50%
Test for overall effect: Z = 0.46 (P = 0.65)

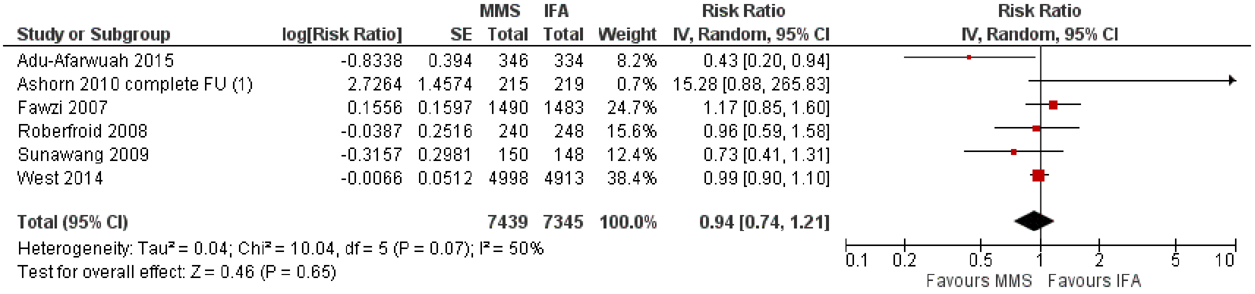

Footnotes
(1) 7 events in the MMS arm and no events in the IFA arm

Supplemental Figure 2.14.4 Effect of MMS vs IFA on low MUAC at 18 months

Fixed

Study or Subgroup	log[Risk Ratio]	SE	MMS Total	IFA Total	Weight	Risk Ratio IV, Fixed, 95% CI
Adu-Afarwuah 2015	-0.3561	0.3896	356	340	52.6%	0.70 [0.33, 1.50]
Ashorn 2010 complete FU	0.3964	0.5184	222	220	29.7%	1.49 [0.54, 4.11]
Ashorn 2010 simplified FU	-0.0077	0.807	130	129	12.3%	0.99 [0.20, 4.83]
Fawzi 2007	-0.3864	1.2064	39	53	5.5%	0.68 [0.06, 7.23]
**Total (95% CI)**			**747**	**742**	**100.0%**	**0.91 [0.52, 1.59]**

Heterogeneity: Chi² = 1.42, df = 3 (P = 0.70); I² = 0%
Test for overall effect: Z = 0.32 (P = 0.75)

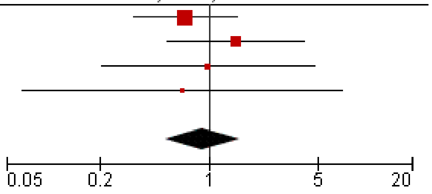

Random

Study or Subgroup	log[Risk Ratio]	SE	MMS Total	IFA Total	Weight	Risk Ratio IV, Random, 95% CI
Adu-Afarwuah 2015	-0.3561	0.3896	356	340	52.6%	0.70 [0.33, 1.50]
Ashorn 2010 complete FU	0.3964	0.5184	222	220	29.7%	1.49 [0.54, 4.11]
Ashorn 2010 simplified FU	-0.0077	0.807	130	129	12.3%	0.99 [0.20, 4.83]
Fawzi 2007	-0.3864	1.2064	39	53	5.5%	0.68 [0.06, 7.23]
**Total (95% CI)**			**747**	**742**	**100.0%**	**0.91 [0.52, 1.59]**

Heterogeneity: Tau² = 0.00; Chi² = 1.42, df = 3 (P = 0.70); I² = 0%
Test for overall effect: Z = 0.32 (P = 0.75)

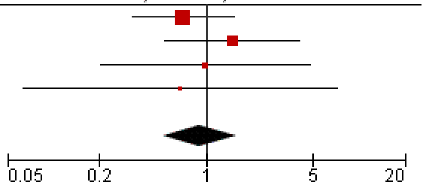

Supplemental Figure 2.14.5 Effect of MMS vs IFA on low MUAC at 24 months

Fixed

Study or Subgroup	log[Risk Ratio]	SE	MMS Total	IFA Total	Weight	Risk Ratio IV, Fixed, 95% CI
Ashorn 2010 complete FU	0.0958	0.624	179	197	0.7%	1.10 [0.32, 3.74]
Roberfroid 2008	-0.233	0.4661	54	55	1.3%	0.79 [0.32, 1.97]
West 2014	-0.0839	0.0542	3433	3381	97.9%	0.92 [0.83, 1.02]
**Total (95% CI)**			**3666**	**3633**	**100.0%**	**0.92 [0.83, 1.02]**

Heterogeneity: Chi² = 0.19, df = 2 (P = 0.91); I² = 0%
Test for overall effect: Z = 1.58 (P = 0.11)

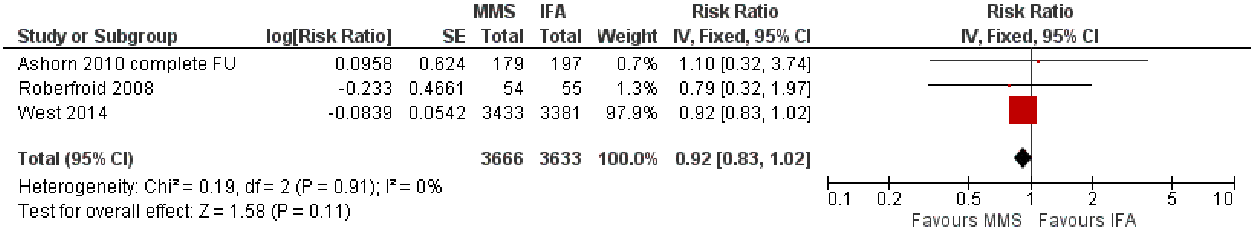

Random

Study or Subgroup	log[Risk Ratio]	SE	MMS Total	IFA Total	Weight	Risk Ratio IV, Random, 95% CI
Ashorn 2010 complete FU	0.0958	0.624	179	197	0.7%	1.10 [0.32, 3.74]
Roberfroid 2008	-0.233	0.4661	54	55	1.3%	0.79 [0.32, 1.97]
West 2014	-0.0839	0.0542	3433	3381	97.9%	0.92 [0.83, 1.02]
**Total (95% CI)**			**3666**	**3633**	**100.0%**	**0.92 [0.83, 1.02]**

Heterogeneity: Tau² = 0.00; Chi² = 0.19, df = 2 (P = 0.91); I² = 0%
Test for overall effect: Z = 1.58 (P = 0.11)

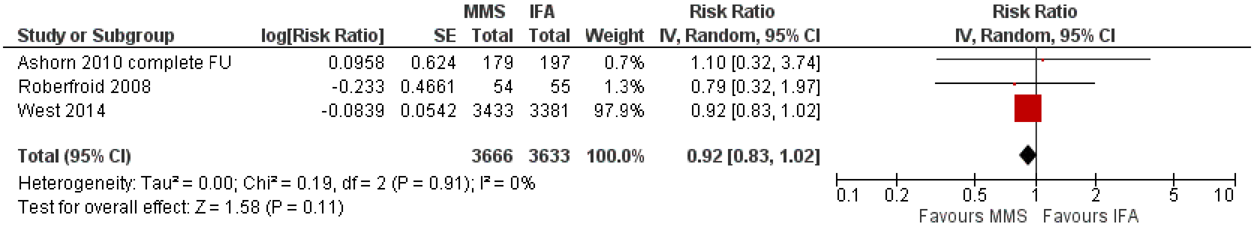

Supplemental Tables 2 - Subgroup analyses stratified by supplementation in pregnancy vs pregnancy and postpartum, and by lower vs higher prevalence rates of low birthweight (LBW)

Supplemental Table 2.1 – Effect of MMS vs IFA on length stratified by supplementation in pregnancy vs pregnancy and postpartum, and by lower vs higher prevalence rates of low birthweight (LBW)

Effect of MMS vs IFA on infant/child length (cm) at each age	n of studies	n of participants	Mean difference (95% CI) Fixed effects model	p value (subgroup differences)	Mean difference (95% CI) Random effects model	p value (subgroup differences)
A. Stratified by supplementation in pregnancy vs. pregnancy and postpartum						
At 3 months of age						
Pregnancy	2	1511	-0.12 (-0.36, 0.13)	0.01	-0.11 (-0.38, 0.16)	0.03
Pregnancy and postpartum	5	23158	0.20 (0.14; 0.27)		0.20 (0.14; 0.27)	
At 6 months of age						
Pregnancy	3	1940	-0.05 (-0.28, 0.18)	0.23	-0.05 (-0.28, 0.18)	0.23
Pregnancy and postpartum	6	16938	0.10 (0.02; 0.18)		0.10 (0.02; 0.18)	
At 12 months of age						
Pregnancy	1	1532	-0.10 (-0.39; 0.19)	0.28	-0.10 (-0.39; 0.19)	0.28
Pregnancy and postpartum	6	14782	0.07 (-0.02; 0.16)		0.07 (-0.02; 0.16)	
At 18 months of age						
Pregnancy	2	1706	0.13 (-0.18; 0.45)	0.55	0.13 (-0.18; 0.45)	0.61
Pregnancy and postpartum	3	1231	0.28 (-0.06; 0.62)		0.27 (-0.15; 0.69)	
At 24 months of age						
Pregnancy	1	1451	-0.20 (-0.58; 0.18)	0.43	-0.20 (-0.58; 0.18)	0.43
Pregnancy and postpartum	3	6999	-0.04 (-0.19; 0.11)		-0.04 (-0.19; 0.11)	
B. Stratified by lower (≤ 11.9%) and higher prevalence (>11.9%) rates of LBW						
At birth						
Lower prevalence of LBW	7	23064	0.01 (-0.03; 0.04)	<0.00001	0.01 (-0.03; 0.04)	0.04
Higher prevalence of LBW	7	20482	0.17 (0.11; 0.24)		0.13 (0.02; 0.25)	
At 3 months of age						
Lower prevalence of LBW	3	6847	0.13 (-0.00; 0.27)	0.58	0.13 (-0.00; 0.27)	0.58
Higher prevalence of LBW	4	17822	0.05 (-0.19; 0.30)		0.05 (-0.19; 0.30)	
At 6 months of age						
Lower prevalence of LBW	3	6999	0.02 (-0.11; 0.15)	0.22	0.02 (-0.11; 0.15)	0.22
Higher prevalence of LBW	6	11879	0.12 (0.03; 0.21)		0.12 (0.03; 0.21)	
At 12 months of age						
Lower prevalence of LBW	3	4226	0.03 (-0.17; 0.23)	0.78	0.03 (-0.17; 0.23)	0.81
High prevalence of LBW	4	12088	0.06 (-0.04; 0.16)		0.05 (-0.05; 0.16)	
At 18 months of age						
Lower prevalence of LBW	2	789	0.45 (0.04; 0.86)	0.15	0.45 (0.04; 0.86)	0.15
Higher prevalence of LBW	3	2148	0.09 (-0.19; 0.36)		0.09 (-0.19; 0.36)	
At 24 months of age						
Lower prevalence of LBW	0	0	N/A (no studies available)	N/A	N/A (no studies available)	N/A

Higher prevalence of LBW	4	8450	-0.06 (-0.20; 0.08)	-0.06 (-0.20; 0.08)

CI = Confidence interval; cm, centimetres; IFA = Iron and Folic Acid supplements; LBW = Low birthweight; MMS = Multiple Micronutrient supplements; N/A = Not applicable

The table shows the generic inverse variance weighted pooled mean differences with their corresponding 95% confidence intervals comparing MMS and IFA intervention groups.

Supplemental Table 2.2 – Effect of MMS vs IFA on head circumference (HC) stratified by supplementation in pregnancy vs pregnancy and postpartum, and by lower vs higher prevalence rates of low birthweight (LBW)

Effect of MMS vs IFA on infant HC (cm) at each age	n of studies	n of participants	Mean difference (95% CI) Fixed effects model	p value (subgroup differences)	Mean difference (95% CI) Random effects model	p value (subgroup differences)
A. Stratified by supplementation in pregnancy vs pregnancy and postpartum						
At 3 months of age						
Pregnancy	2	1515	-0.04 (-0.19, 0.10))	0.02	-0.04 (-0.20, 0.12)	0.09
Pregnancy and postpartum	4	23250	0.13 (0.10; 0.17)		0.12 (0.03; 0.20)	
At 6 months of age						
Pregnancy	3	1944	0.02 (-0.11, 0.15)	0.52	0.02 (-0.11, 0.15)	0.53
Pregnancy and postpartum	5	16815	0.07 (0.02; 0.11)		0.07 (0.00; 0.13)	
At 12 months of age						
Pregnancy	1	1539	0.10 (-0.05; 0.25)	0.44	0.10 (-0.05; 0.25)	0.44
Pregnancy and postpartum	5	14631	0.04 (-0.01; 0.09)		0.04 (-0.01; 0.09)	
At 18 months of age						
Pregnancy	1	260	0.00 (-0.34; 0.34)	0.59	0.00 (-0.34; 0.34)	0.58
Pregnancy and postpartum	3	1228	-0.10 (-0.26; 0.05)		-0.16 (-0.61; 0.29)	
At 24 months of age						
Pregnancy	0	0	N/A (no studies available)	N/A	N/A (no studies available)	N/A
Pregnancy and postpartum	2	7136	0.01 (-0.06; 0.08)		-0.03 (-0.22; 0.15)	
B. Stratified by lower (≤ 11.9%) and higher prevalence (>11.9%) rates of LBW						
At birth						
Lower prevalence of LBW	5	11169	0.03 (-0.04; 0.10)	0.001	0.03 (-0.06; 0.12)	0.09
Higher prevalence of LBW	7	20683	0.17 (0.13; 0.21)		0.14 (0.05; 0.22)	
At 3 months of age						
Lower prevalence of LBW	2	6701	0.06 (-0.01; 0.12)	0.03	0.10 (-0.07; 0.26)	0.84
Higher prevalence of LBW	4	18064	0.14 (0.10; 0.18)		0.07 (-0.06; 0.20)	
At 6 months of age						
Lower prevalence of LBW	2	6750	0.03 (-0.04; 0.10)	0.26	0.08 (-0.11; 0.27)	0.97
Higher prevalence of LBW	6	12009	0.08 (0.03; 0.13)		0.08 (0.03; 0.13)	
At 12 months of age						
Lower prevalence of LBW	2	3905	0.04 (-0.06; 0.13)	0.84	0.04 (-0.06; 0.13)	0.84
Higher prevalence of LBW	4	12265	0.05 (-0.00; 0.10)		0.05 (-0.00; 0.10)	
At 18 months of age						
Lower prevalence of LBW	2	786	0.08 (-0.10; 0.27)	0.007	0.08 (-0.10; 0.27)	0.20
Higher prevalence of LBW	2	702	-0.31 (-0.52; -0.09)		-0.26 (-0.75; 0.23)	
At 24 months of age						
Lower prevalence of LBW	0	0	N/A (no studies available)	N/A	N/A (no studies available)	N/A
Higher prevalence of LBW	2	7136	0.01 (-0.06; 0.08)		-0.03 (-0.22; 0.15)	

CI = Confidence interval; cm, centimetres; IFA = Iron and Folic Acid supplements; LBW = Low birthweight; MMS = Multiple Micronutrient supplements; N/A = Not applicable

The table shows the generic inverse variance weighted pooled mean differences with their corresponding 95% confidence intervals comparing MMS and IFA intervention groups.

Supplemental Table 2.3 – Effect of MMS vs IFA on weight stratified by supplementation in pregnancy vs pregnancy and postpartum, and by lower vs higher prevalence rates of low birthweight (LBW)

Effect of MMS vs IFA on infant/child weight (kg) at each age	n of studies	n of participants	Mean difference (95% CI) Fixed effects model	p value (subgroup differences)	Mean difference (95% CI) Random effects model	p value (subgroup differences)
A. Stratified by supplementation in pregnancy vs pregnancy and postpartum						
At 3 months of age						
Pregnancy	2	1523	-0.01 (-0.09; 0.07)	0.002	-0.01 (-0.09; 0.07)	0.05
Pregnancy and postpartum	5	23467	0.12 (0.10; 0.14)		0.13 (0.02; 0.24)	
At 6 months of age						
Pregnancy	3	1949	0.01 (-0.08; 0.10)	0.45	0.01 (-0.08; 0.10)	0.45
Pregnancy and postpartum	6	17114	0.04 (0.02; 0.07)		0.04 (0.02; 0.07)	
At 12 months of age						
Pregnancy	1	1539	0.07 (-0.05; 0.19)	0.43	0.07 (-0.05; 0.19)	0.43
Pregnancy and postpartum	6	14995	0.02 (-0.01; 0.05)		0.02 (-0.01; 0.05)	
At 18 months of age						
Pregnancy	2	1711	0.10 (-0.02; 0.22)	0.76	0.10 (-0.02; 0.22)	0.71
Pregnancy and postpartum	6	1230	0.07 (-0.07; 0.21)		0.05 (-0.14; 0.25)	
At 24 months of age						
Pregnancy	1	1454	0.01 (-0.13; 0.15)	0.96	0.01 (-0.13; 0.15)	0.96
Pregnancy and postpartum	3	7264	0.01 (-0.05; 0.06)		0.01 (-0.05; 0.06)	
B. Stratified by lower (≤ 11.9%) and higher prevalence (>11.9%) rates of LBW						
At birth						
Lower prevalence of LBW	9	38093	0.03 (0.02; 0.04)	0.06	0.03 (0.01; 0.06)	0.40
Higher prevalence of LBW	9	22286	0.05 (0.04; 0.06)		0.04 (0.03; 0.06)	
At 3 months of age						
Lower prevalence of LBW	3	7039	0.24 (0.20; 0.28)	<0.00001	0.16 (0.02; 0.30)	0.16
Higher prevalence of LBW	4	17951	0.07 (0.05; 0.09)		0.05 (0.01; 0.10)	
At 6 months of age						
Lower prevalence of LBW	3	7090	0.03 (-0.02; 0.08)	0.59	0.03 (-0.02; 0.08)	0.59
Higher prevalence of LBW	6	11973	0.04 (0.01; 0.08)		0.04 (0.01; 0.08)	
At 12 months of age						
Lower prevalence of LBW	3	4245	0.04 (-0.03; 0.11)	0.67	0.04 (-0.07; 0.16)	0.69
Higher prevalence of LBW	4	12289	0.02 (-0.01; 0.06)		0.02 (-0.01; 0.06)	
At 18 months of age						
Lower prevalence of LBW	3	788	0.16 (-0.01; 0.33)	0.31	0.16 (-0.01; 0.33)	0.27
Higher prevalence of LBW	5	2153	0.06 (-0.05; 0.16)		0.04 (-0.10; 0.18)	
At 24 months of age						
Lower prevalence of LBW	0	0	N/A (no studies available)	N/A	N/A (no studies available)	N/A
Higher prevalence of LBW	4	8718	0.01 (-0.04; 0.05)		0.01 (-0.04; 0.05)	

CI = Confidence interval; IFA = Iron and Folic Acid supplements; LBW = Low birthweight; kg, kilogram; MMS = Multiple Micronutrient supplements; N/A = Not applicable

The table shows the generic inverse variance weighted pooled mean differences with their corresponding 95% confidence intervals comparing MMS and IFA intervention groups.

Supplemental Table 2.4 – Effect of MMS vs IFA on mid-upper arm circumference (MUAC) stratified by supplementation in pregnancy vs pregnancy and postpartum, and by lower vs higher prevalence rates of low birthweight (LBW)

Effect of MMS vs IFA on infant/child MUAC (cm) at each age	n of studies	n of participants	Mean difference (95% CI) Fixed effects model	p value (subgroup differences)	Mean difference (95% CI) Random effects model	p value (subgroup differences)
A. Stratified by supplementation in pregnancy vs pregnancy and postpartum						
At 3 months of age						
Pregnancy	1	351	0.01 (-0.19, 0.21)	0.47	0.01 (-0.19, 0.21)	0.67
Pregnancy and postpartum	5	20586	0.08 (0.06; 0.11)		0.06 (-0.01; 0.12)	
At 6 months of age						
Pregnancy	2	603	0.00 (-0.17, 0.18)	0.86	0.00 (-0.17, 0.18)	0.86
Pregnancy and postpartum	6	14785	0.02 (-0.01; 0.06)		0.02 (-0.01; 0.06)	
At 12 months of age						
Pregnancy	0	0	N/A (no studies available)	N/A	N/A (no studies available)	N/A
Pregnancy and postpartum	6	14788	0.02 (-0.01; 0.06)		0.02 (-0.01; 0.06)	
At 18 months of age						
Pregnancy	1	260	0.10 (-0.18; 0.38)	0.75	0.10 (-0.18; 0.38)	0.75
Pregnancy and postpartum	3	1230	0.05 (-0.07; 0.17)		0.05 (-0.07; 0.17)	
At 24 months of age						
Pregnancy	1	1360	-0.16 (-0.48; 0.16)	0.35	-0.16 (-0.48; 0.16)	0.48
Pregnancy and postpartum	3	7299	-0.01 (-0.05; 0.04)		-0.04 (-0.15; 0.08)	
B. Stratified by lower (≤ 11.9%) and higher prevalence (>11.9%) rates of LBW						
At birth						
Lower prevalence of LBW	1	607	0.10 (-0.04; 0.24)	0.91	0.10 (-0.04; 0.24)	0.91
Higher prevalence of LBW	4	17174	0.11 (0.08; 0.13)		0.11 (0.08; 0.13)	
At 3 months of age						
Lower prevalence of LBW	3	4001	0.01 (-0.06; 0.08)	0.04	0.01 (-0.06; 0.08)	0.08
Higher prevalence of LBW	3	16936	0.09 (0.06; 0.12)		0.09 (0.04; 0.13)	
At 6 months of age						
Lower prevalence of LBW	3	4703	0.01 (-0.06; 0.08)	0.74	0.01 (-0.06; 0.08)	0.74
Higher prevalence of LBW	5	10685	0.02 (-0.02; 0.06)		0.02 (-0.02; 0.06)	
At 12 months of age						
Lower prevalence of LBW	3	3955	0.04 (-0.04; 0.11)	0.71	0.04 (-0.04; 0.11)	0.71
Higher prevalence of LBW	3	10833	0.02 (-0.02; 0.06)		0.02 (-0.02; 0.06)	
At 18 months of age						
Lower prevalence of LBW	2	788	0.08 (-0.07; 0.22)	0.74	0.05 (-0.19; 0.28)	0.95
Higher prevalence of LBW	2	702	0.04 (-0.13; 0.21)		0.04 (-0.13; 0.21)	
At 24 months of age						
Lower prevalence of LBW	0	0	N/A (no studies available)	N/A	N/A (no studies available)	N/A
Higher prevalence of LBW	3	7299	-0.01 (-0.05; 0.04)		-0.04 (-0.15; 0.08)	
LBW prevalence not known	1	1360	-0.16 (-0.48; 0.16)		-0.16 (-0.48; 0.16)	

CI = Confidence interval; cm, centimetres; IFA = Iron and Folic Acid supplements; LBW = Low birthweight; MMS = Multiple Micronutrient supplements; MUAC = Mid upper arm circumference; N/A = Not applicable

The table shows the generic inverse variance weighted pooled mean differences with their corresponding 95% confidence intervals comparing MMS and IFA intervention groups.

Supplemental Table 2.5 – Effect of MMS vs IFA on length-for-age Z-score (LAZ) stratified by supplementation in pregnancy vs pregnancy and postpartum, and by lower vs higher prevalence rates of low birthweight (LBW)

Effect of MMS vs IFA on infant/child LAZ at each age	n of studies	n of participants	Mean difference (95% CI) Fixed effects model	p value (subgroup differences)	Mean difference (95% CI) Random effects model	p value (subgroup differences)
A. Stratified by supplementation in pregnancy vs pregnancy and postpartum						
At 3 months of age						
Pregnancy	2	1505	-0.03 (-0.15; 0.08)	0.03	-0.03 (-0.15; 0.08)	0.03
Pregnancy and postpartum	5	23120	0.10 (0.07; 0.13)		0.10 (0.07; 0.13)	
At 6 months of age						
Pregnancy	3	1932	-0.03 (-0.13; 0.07)	0.01	-0.03 (-0.13; 0.07)	0.01
Pregnancy and postpartum	6	16918	0.05 (0.01; 0.08)		0.05 (0.01; 0.08)	
At 12 months of age						
Pregnancy	1	1525	-0.04 (-0.16; 0.08)	0.28	-0.04 (-0.16; 0.08)	0.28
Pregnancy and postpartum	6	12768	0.03 (-0.01; 0.07)		0.03 (-0.01; 0.07)	
At 18 months of age						
Pregnancy	2	1696	-0.08 (-0.19; 0.03)	0.01	-0.08 (-0.19; 0.03)	0.04
Pregnancy and postpartum	3	1231	0.12 (0.01; 0.24)		0.12 (-0.04; 0.29)	
At 24 months of age						
Pregnancy	2	1694	-0.07 (-0.18; 0.04)	0.40	-0.07 (-0.18; 0.04)	0.40
Pregnancy and postpartum	3	6999	-0.02 (-0.07; 0.04)		-0.02 (-0.07; 0.03)	
B. Stratified by lower (≤ 11.9%) and higher prevalence (>11.9%) rates of LBW						
At birth						
Lower prevalence of LBW	3	7492	0.05 (-0.03; 0.13)	0.34	0.05 (-0.03; 0.14)	0.39
Higher prevalence of LBW	5	18172	0.09 (0.06; 0.13)		0.09 (0.06; 0.13)	
At 3 months of age						
Lower prevalence of LBW	3	6809	0.07 (0.01; 0.13)	0.45	0.07 (-0.00; 0.15)	0.67
Higher prevalence of LBW	4	17816	0.10 (0.06; 0.13)		0.05 (-0.04; 0.14)	
At 6 months of age						
Lower prevalence of LBW	3	6979	0.02 (-0.03; 0.08)	0.47	0.02 (-0.03; 0.08)	0.47
Higher prevalence of LBW	6	11871	0.05 (0.01; 0.09)		0.05 (0.01; 0.09)	
At 12 months of age						
Lower prevalence of LBW	3	4212	0.04 (-0.04; 0.11)	0.64	0.04 (-0.04; 0.11)	0.64
Higher prevalence of LBW	4	10081	0.02 (-0.03; 0.06)		0.02 (-0.03; 0.06)	
At 18 months of age						
Lower prevalence of LBW	2	789	0.18 (0.04; 0.32)	0.004	0.18 (0.04; 0.32)	0.004
Higher prevalence of LBW	3	2138	-0.07 (-0.16; 0.03)		-0.07 (-0.16; 0.03)	
At 24 months of age						
Lower prevalence of LBW	0	0	N/A (no studies available)	N/A	N/A (no studies available)	N/A
Higher prevalence of LBW	4	8445	-0.03 (-0.07; 0.02)		-0.03 (-0.07; 0.02)	
LBW prevalence not known	1	248	-0.07 (-0.33; 0.19)		-0.07 (-0.33; 0.19)	

CI = Confidence interval; IFA = Iron and Folic Acid supplements; LAZ = Length-for-age Z-score; LBW = Low birthweight; MMS = Multiple Micronutrient supplements; N/A = Not applicable

The table shows the generic inverse variance weighted pooled mean differences with their corresponding 95% confidence intervals comparing MMS and IFA intervention groups.

Supplemental Table 2.6 – Effect of MMS vs IFA on weight-for-age Z-score (WAZ) stratified by supplementation in pregnancy vs pregnancy and postpartum, and by lower vs higher prevalence rates of low birthweight (LBW)

Effect of MMS vs IFA on infant/child WAZ at each age	n of studies	n of participants	Mean difference (95% CI) Fixed effects model	p value (subgroup differences)	Mean difference (95% CI) Random effects model	p value (subgroup differences)
A. Stratified by supplementation in pregnancy vs pregnancy and postpartum						
At 3 months of age						
Pregnancy	2	1517	0.03 (-0.09; 0.14)	0.20	0.03 (-0.09; 0.14)	0.35
Pregnancy and postpartum	5	23452	0.10 (0.08; 0.13)		0.09 (0.02; 0.17)	
At 6 months of age						
Pregnancy	3	1942	0.02 (-0.09; 0.12)	0.20	0.02 (-0.09; 0.12)	0.68
Pregnancy and postpartum	6	17099	0.04 (0.01; 0.07)		0.04 (0.01; 0.07)	
At 12 months of age						
Pregnancy	1	1532	0.06 (-0.06; 0.18)	0.54	0.06 (-0.06; 0.18)	0.55
Pregnancy and postpartum	6	14991	0.02 (-0.01; 0.05)		0.02 (-0.02; 0.07)	
At 18 months of age						
Pregnancy	2	1696	0.05 (-0.06; 0.16)	0.91	0.05 (-0.06; 0.16)	0.98
Pregnancy and postpartum	3	1230	0.06 (-0.06; 0.18)		0.05 (-0.14; 0.24)	
At 24 months of age						
Pregnancy	2	2809	-0.00 (-0.11; 0.10)	0.91	-0.00 (-0.11; 0.10)	0.91
Pregnancy and postpartum	3	7264	0.01 (-0.04; 0.05)		0.01 (-0.04; 0.05)	
B. Stratified by lower (≤ 11.9%) and higher prevalence (>11.9%) rates of LBW						
At birth						
Lower prevalence of LBW	3	9565	0.11 (0.06; 0.15)	0.66	0.09 (0.00; 0.18)	0.86
Higher prevalence of LBW	6	18879	0.12 (0.09; 0.15)		0.10 (0.05; 0.15)	
At 3 months of age						
Lower prevalence of LBW	3	7024	0.03 (-0.02; 0.08)	0.004	0.06 (-0.05; 0.17)	0.27
Higher prevalence of LBW	4	17945	0.12 (0.09; 0.15)		0.12 (0.09; 0.15)	
At 6 months of age						
Lower prevalence of LBW	3	7075	0.03 (-0.03; 0.08)	0.71	0.03 (-0.03; 0.08)	0.71
Higher prevalence of LBW	6	11966	0.04 (0.00; 0.08)		0.04 (0.00; 0.08)	
At 12 months of age						
Lower prevalence of LBW	3	4241	0.05 (-0.02; 0.11)	0.47	0.06 (-0.04; 0.16)	0.46
Higher prevalence of LBW	4	12282	0.02 (-0.02; 0.05)		0.02 (-0.02; 0.05)	
At 18 months of age						
Lower prevalence of LBW	2	788	0.15 (0.00; 0.29)	0.13	0.15 (0.00; 0.29)	0.13
Higher prevalence of LBW	3	2138	0.01 (-0.08; 0.11)		0.01 (-0.08; 0.11)	
At 24 months of age						
Lower prevalence of LBW	0	0	N/A (no studies available)	N/A	N/A (no studies available)	N/A
Higher prevalence of LBW	4	8713	0.01 (-0.03; 0.05)		0.01 (-0.03; 0.05)	
LBW prevalence not known	1	1360	-0.05 (-0.29; 0.19)		-0.05 (-0.29; 0.19)	

CI = Confidence interval; IFA = Iron and Folic Acid supplements; LBW = Low birthweight; MMS = Multiple Micronutrient supplements; WAZ = Weight-for-age Z-score; N/A = Not applicable

The table shows the generic inverse variance weighted pooled mean differences with their corresponding 95% confidence intervals comparing MMS and IFA intervention groups.

Supplemental Table 2.7 – Effect of MMS vs IFA on weight-for-age Z-score (WLZ) [or body mass index Z-score (BMI)] stratified by supplementation in pregnancy vs pregnancy and postpartum, and by lower vs higher prevalence rates of low birthweight (LBW)

Effect of MMS vs IFA on infant/child WLZ (or BMIZ at birth) at each age	n of studies	n of participants	Mean difference (95% CI) Fixed effects model	p value (subgroup differences)	Mean difference (95% CI) Random effects model	p value (subgroup differences)
A. Stratified by supplementation in pregnancy vs pregnancy and postpartum						
At 3 months of age						
Pregnancy	2	1502	0.04 (-0.08; 0.17)	0.83	0.04 (-0.08; 0.17)	0.80
Pregnancy and postpartum	5	23186	0.03 (-0.00; 0.06)		0.03 (-0.01; 0.07)	
At 6 months of age						
Pregnancy	3	1932	0.00 (-0.10; 0.11)	0.98	0.00 (-0.10; 0.11)	0.98
Pregnancy and postpartum	6	16936	0.00 (-0.03; 0.04)		0.00 (-0.03; 0.04)	
At 12 months of age						
Pregnancy	1	1525	0.10 (-0.02; 0.22)	0.20	0.10 (-0.02; 0.22)	0.23
Pregnancy and postpartum	6	14772	0.02 (-0.03; 0.06)		0.02 (-0.05; 0.08)	
At 18 months of age						
Pregnancy	2	1694	0.09 (-0.01; 0.20)	0.33	0.09 (-0.01; 0.20)	0.36
Pregnancy and postpartum	3	1228	0.02 (-0.10; 0.13)		0.06 (-0.03; 0.14)	
At 24 months of age						
Pregnancy	2	2806	0.03 (-0.07; 0.12)	0.52	0.03 (-0.07; 0.12)	0.52
Pregnancy and postpartum	3	7004	-0.01 (-0.05; 0.04)		-0.01 (-0.05; 0.04)	
B. Stratified by lower (≤ 11.9%) and higher prevalence (>11.9%) rates of LBW						
At birth						
Lower prevalence of LBW	3	7457	0.08 (0.00; 0.16)	0.92	0.10 (-0.02; 0.22)	0.64
Higher prevalence of LBW	5	15427	0.08 (0.05; 0.12)		0.07 (-0.00; 0.14)	
At 3 months of age						
Lower prevalence of LBW	3	6785	-0.01 (-0.07; 0.05)	0.10	-0.01 (-0.07; 0.05)	0.10
Higher prevalence of LBW	4	17903	0.04 (0.01; 0.08)		0.04 (0.01; 0.08)	
At 6 months of age						
Lower prevalence of LBW	3	6970	0.02 (-0.04; 0.08)	0.44	0.02 (-0.04; 0.08)	0.44
Higher prevalence of LBW	6	11898	-0.01 (-0.04; 0.03)		-0.01 (-0.04; 0.03)	
At 12 months of age						
Lower prevalence of LBW	3	4211	0.03 (-0.04; 0.11)	0.80	0.03 (-0.11; 0.17)	0.97
Higher prevalence of LBW	4	12086	0.02 (-0.03; 0.07)		0.03 (-0.04; 0.09)	
At 18 months of age						
Lower prevalence of LBW	2	786	0.09 (-0.05; 0.24)	0.58	0.09 (-0.05; 0.24)	0.49
Higher prevalence of LBW	3	2136	0.04 (-0.05; 0.14)		0.02 (-0.12; 0.16)	
At 24 months of age						
Lower prevalence of LBW	0	0	N/A (no studies available)	N/A	N/A (no studies available)	N/A
Higher prevalence of LBW	4	8450	0.00 (-0.04; 0.04)		0.00 (-0.04; 0.04)	
LBW prevalence not known	1	1360	-0.05 (-0.25; 0.15)		-0.05 (-0.25; 0.15)	

BMIZ = Body Mass Index Z-score; CI = Confidence interval; IFA = Iron and Folic Acid supplements; LBW = Low birthweight; MMS = Multiple Micronutrient supplements; N/A = Not applicable; WLZ = Weight-for-length Z-score

The table shows the generic inverse variance weighted pooled mean differences with their corresponding 95% confidence intervals comparing MMS and IFA intervention groups.

Supplemental Table 2.8 – Effect of MMS vs IFA on head circumference-for-age Z-score (HCAZ) stratified by supplementation in pregnancy vs pregnancy and postpartum, and by lower vs higher prevalence rates of low birthweight (LBW)

Effect of MMS vs IFA on infant/child HCAZ at each age	n of studies	n of participants	Mean difference (95% CI) Fixed effects model	p value (subgroup differences)	Mean difference (95% CI) Random effects model	p value (subgroup differences)
A. Stratified by supplementation in pregnancy vs pregnancy and postpartum						
At 3 months of age						
Pregnancy	2	1515	0.02 (-0.10; 0.14)	0.18	0.02 (-0.10; 0.14)	0.28
Pregnancy and postpartum	4	23205	0.10 (0.07; 0.13)		0.10 (0.02; 0.17)	
At 6 months of age						
Pregnancy	3	1944	0.04 (-0.05; 0.14)	0.96	0.04 (-0.05; 0.14)	0.94
Pregnancy and postpartum	5	16790	0.04 (0.01; 0.07)		0.04 (-0.00; 0.08)	
At 12 months of age						
Pregnancy	1	1539	0.00 (-0.11; 0.11)	0.67	0.00 (-0.11; 0.11)	0.67
Pregnancy and postpartum	5	14624	0.03 (-0.01; 0.06)		0.03 (-0.01; 0.06)	
At 18 months of age						
Pregnancy	1	260	0.01 (-0.22; 0.24)	0.78	0.01 (-0.22; 0.24)	0.78
Pregnancy and postpartum	3	1228	0.05 (-0.06; 0.15)		0.05 (-0.06; 0.15)	
At 24 months of age						
Pregnancy	0	0	N/A (no studies available)	N/A	N/A (no studies available)	N/A
Pregnancy and postpartum	2	7136	0.00 (-0.04; 0.05)		-0.03 (-0.16; 0.10)	
B. Stratified by lower (≤ 11.9%) and higher prevalence (>11.9%) rates of LBW						
At birth						
Lower prevalence of LBW	3	8570	0.04 (-0.03; 0.11)	0.01	0.04 (-0.03; 0.11)	0.01
Higher prevalence of LBW	4	17332	0.14 (0.11; 0.18)		0.14 (0.11; 0.18)	
At 3 months of age						
Lower prevalence of LBW	2	6656	0.04 (-0.02; 0.10)	0.03	0.09 (-0.09; 0.26)	0.79
Higher prevalence of LBW	4	18064	0.11 (0.08; 0.14)		0.11 (0.08; 0.14)	
At 6 months of age						
Lower prevalence of LBW	2	6725	0.01 (-0.04; 0.06)	0.14	0.04 (-0.08; 0.16)	0.79
Higher prevalence of LBW	6	12009	0.06 (0.02; 0.09)		0.06 (0.02; 0.09)	
At 12 months of age						
Lower prevalence of LBW	2	3898	0.04 (-0.03; 0.10)	0.61	0.04 (-0.03; 0.10)	0.61
Higher prevalence of LBW	4	12265	0.02 (-0.01; 0.05)		0.02 (-0.01; 0.05)	
At 18 months of age						
Lower prevalence of LBW	2	786	0.08 (-0.05; 0.21)	0.39	0.08 (-0.05; 0.21)	0.39
Higher prevalence of LBW	2	702	-0.01 (-0.15; 0.14)		-0.01 (-0.15; 0.14)	
At 24 months of age						
Lower prevalence of LBW	0	0	N/A (no studies available)	N/A	Not applicable (no studies available)	N/A
Higher prevalence of LBW	2	7136	0.00 (-0.04; 0.05)		-0.03 (-0.16; 0.10)	

CI = Confidence interval; IFA = Iron and Folic Acid supplements; HCAZ = Head circumference Z-score; LBW = Low birthweight; MMS = Multiple Micronutrient supplements; N/A = Not applicable
The table shows the generic inverse variance weighted pooled mean differences with their corresponding 95% confidence intervals comparing MMS and IFA intervention groups.

Supplemental Table 2.9 – Effect of MMS vs IFA on MUACZ stratified by supplementation in pregnancy vs pregnancy and postpartum, and by lower vs higher prevalence rates of low birthweight (LBW)

Effect of MMS vs IFA on infant/child MUACZ at each stage	n of studies	n of participants	Mean difference (95% CI)<br>Fixed effects model	p value (subgroup differences)	Mean difference (95% CI)<br>Random effects model	p value (subgroup differences)
A. Stratified by supplementation in pregnancy vs pregnancy and postpartum						
At 3 months of age						
Pregnancy	1	304	0.05 (-0.15, 0.24)	0.78	0.05 (-0.15, 0.24)	0.93
Pregnancy and postpartum	5	20429	0.07 (0.05; 0.10)		0.06 (-0.00; 0.12)	
At 6 months of age						
Pregnancy	2	603	0.04 (-0.12, 0.20)	0.84	0.04 (-0.12, 0.20)	0.85
Pregnancy and postpartum	6	14779	0.02 (-0.01; 0.05)		0.02 (-0.01; 0.06)	
At 12 months of age						
Pregnancy	0	0	N/A (no studies available)	N/A	N/A (no studies available)	N/A
Pregnancy and postpartum	6	14784	0.02 (-0.01; 0.05)		0.02 (-0.01; 0.05)	
At 18 months of age						
Pregnancy	1	260	0.09 (-0.15; 0.33)	0.82	0.09 (-0.15; 0.33)	0.81
Pregnancy and postpartum	3	1230	0.06 (-0.05; 0.17)		0.06 (-0.06; 0.17)	
At 24 months of age						
Pregnancy	0	0	N/A (no studies available)	N/A	N/A (no studies available)	N/A
Pregnancy and postpartum	3	7299	-0.01 (-0.05; 0.03)		-0.04 (-0.16; 0.08)	
B. Stratified by lower (≤ 11.9%) and higher prevalence (>11.9%) rates of LBW						
At birth						
Lower prevalence of LBW	N/A	N/A	N/A	N/A	N/A	N/A
Higher prevalence of LBW	N/A	N/A	N/A		N/A	
At 3 months of age						
Lower prevalence of LBW	3	3844	0.03 (-0.04; 0.09)	0.11	0.05 (-0.06; 0.16)	0.58
Higher prevalence of LBW	3	16889	0.08 (0.06; 0.11)		0.08 (0.06; 0.11)	
At 6 months of age						
Lower prevalence of LBW	3	4697	0.00 (-0.06; 0.06)	0.46	0.00 (-0.06; 0.06)	0.46
Higher prevalence of LBW	5	10685	0.03 (-0.01; 0.06)		0.03 (-0.01; 0.06)	
At 12 months of age						
Lower prevalence of LBW	3	3951	0.05 (-0.01; 0.12)	0.24	0.07 (-0.03; 0.16)	0.26
Higher prevalence of LBW	3	10833	0.01 (-0.03; 0.04)		0.01 (-0.03; 0.04)	
At 18 months of age						
Lower prevalence of LBW	2	788	0.10 (-0.03; 0.23)	0.44	0.09 (-0.06; 0.25)	0.52
Higher prevalence of LBW	2	702	0.02 (-0.13; 0.17)		0.02 (-0.13; 0.17)	
At 24 months of age						
Lower prevalence of LBW	0	0	N/A (no studies available)	N/A	N/A (no studies available)	N/A
Higher prevalence of LBW	3	7299	-0.01 (-0.05; 0.03)		-0.04 (-0.16; 0.08)	

CI = Confidence interval; IFA = Iron and Folic Acid supplements; LBW = Low birthweight; MMS = Multiple Micronutrient supplements; MUACZ = Mid upper arm circumference Z-score; N/A = Not applicable
The table shows the generic inverse variance weighted pooled mean differences with their corresponding 95% confidence intervals comparing MMS and IFA intervention groups.

Supplemental Table 2.10 – Effect of MMS vs IFA on stunting (LAZ < -2) stratified by supplementation in pregnancy vs pregnancy and postpartum, and by lower vs higher prevalence rates of low birthweight (LBW)

Effect of MMS vs IFA on infant/child stunting at each age	n of studies	n of participants	Risk Ratio (95% CI) Fixed effects model	p value (subgroup differences)	Risk Ratio (95% CI) Random effects model	p value (subgroup differences)
A. Stratified by supplementation in pregnancy vs pregnancy and postpartum						
At 3 months of age						
Pregnancy	2	1505	1.10 (0.91; 1.34)	0.01	1.18 (0.79; 1.76)	0.12
Pregnancy and postpartum	5	23120	0.85 (0.81; 0.89)		0.85 (0.81; 0.89)	
At 6 months of age						
Pregnancy	3	1933	1.19 (1.01; 1.40)	0.007	1.19 (1.01; 1.40)	0.007
Pregnancy and postpartum	6	16918	0.94 (0.88; 0.99)		0.94 (0.88; 0.99)	
At 12 months of age						
Pregnancy	1	1525	1.05 (0.91; 1.21)	0.58	1.05 (0.91; 1.21)	0.58
Pregnancy and postpartum	6	14771	1.01 (0.97; 1.06)		1.01 (0.97; 1.06)	
At 18 months of age						
Pregnancy	2	1695	1.00 (0.90; 1.11)	0.74	1.00 (0.90; 1.11)	0.53
Pregnancy and postpartum	3	1228	0.96 (0.78; 1.18)		0.89 (0.64; 1.25)	
At 24 months of age						
Pregnancy	2	2806	1.04 (0.95; 1.13)	0.78	1.04 (0.95; 1.13)	0.78
Pregnancy and postpartum	3	6999	1.02 (0.97; 1.07)		1.02 (0.97; 1.07)	
B. Stratified by lower (≤ 11.9%) and higher prevalence (>11.9%) rates of LBW						
At birth						
Lower prevalence of LBW	4	8098	0.95 (0.88; 1.02)	0.24	0.95 (0.88; 1.02)	0.31
Higher prevalence of LBW	5	18162	0.90 (0.86; 0.94)		0.90 (0.85; 0.95)	
At 3 months of age						
Lower prevalence of LBW	3	6809	0.90 (0.78; 1.05)	0.53	0.90 (0.78; 1.05)	0.62
Higher prevalence of LBW	4	17816	0.86 (0.82; 0.90)		0.96 (0.79; 1.18)	
At 6 months of age						
Lower prevalence of LBW	3	6979	0.94 (0.82; 1.07)	0.64	0.94 (0.78; 1.13)	0.51
Higher prevalence of LBW	6	11872	0.97 (0.91; 1.03)		1.02 (0.89; 1.16)	
At 12 months of age						
Lower prevalence of LBW	3	4215	1.07 (0.95; 1.21)	0.35	1.07 (0.95; 1.21)	0.35
Higher prevalence of LBW	4	12081	1.01 (0.96; 1.05)		1.01 (0.96; 1.05)	
At 18 months of age						
Lower prevalence of LBW	2	786	0.73 (0.52; 1.03)	0.07	0.73 (0.52; 1.03)	0.07
Higher prevalence of LBW	3	2137	1.01 (0.92; 1.12)		1.01 (0.92; 1.12)	
At 24 months of age						
Lower prevalence of LBW	0	0	N/A (no studies available)	N/A	N/A (no studies available)	N/A
Higher prevalence of LBW	4	8445	1.03 (0.98; 1.07)		1.03 (0.98; 1.07)	
LBW prevalence not known	1	1360	1.01 (0.88; 1.16)		1.01 (0.88; 1.16)	

CI = Confidence interval; IFA = Iron and Folic Acid supplements; LBW = Low birthweight; MMS = Multiple Micronutrient supplements; N/A = Not applicable
The table shows the generic inverse variance weighted pooled risk ratios with their corresponding 95% confidence intervals comparing MMS and IFA intervention groups.

Supplemental Table 2.11 – Effect of MMS vs IFA on underweight (WAZ < -2) stratified by supplementation in pregnancy vs pregnancy and postpartum, and by lower vs higher prevalence rates of low birthweight (LBW)

Effect of MMS vs IFA on infant/child underweight at each age	n of studies	n of participants	Risk Ratio (95% CI) Fixed effects model	p value (subgroup differences)	Risk Ratio (95% CI) Random effects model	p value (subgroup differences)
A. Stratified by supplementation in pregnancy vs pregnancy and postpartum						
At 3 months of age						
Pregnancy	2	1517	1.01 (0.81; 1.25)	0.14	1.01 (0.81; 1.25)	0.14
Pregnancy and postpartum	5	23452	0.85 (0.81; 0.89)		0.85 (0.81; 0.89)	
At 6 months of age						
Pregnancy	3	2032	1.07 (0.87; 1.31)	0.34	1.07 (0.87; 1.31)	0.34
Pregnancy and postpartum	6	17099	0.96 (0.90; 1.03)		0.96 (0.90; 1.03)	
At 12 months of age						
Pregnancy	1	1532	0.89 (0.75; 1.06)	0.32	0.89 (0.75; 1.06)	0.39
Pregnancy and postpartum	6	14991	0.97 (0.93; 1.02)		0.97 (0.88; 1.07)	
At 18 months of age						
Pregnancy	2	1695	0.90 (0.77; 1.04)	0.73	0.90 (0.77; 1.04)	0.91
Pregnancy and postpartum	3	1230	0.85 (0.63; 1.14)		0.93 (0.53; 1.62)	
At 24 months of age						
Pregnancy	2	2809	0.99 (0.87; 1.11)	0.71	0.99 (0.87; 1.11)	0.71
Pregnancy and postpartum	3	7264	1.01 (0.96; 1.06)		1.01 (0.96; 1.06)	
B. Stratified by lower (≤ 11.9%) and higher prevalence (>11.9%) rates of LBW						
At birth						
Lower prevalence of LBW	3	9565	0.85 (0.74; 0.98)	0.71	0.85 (0.74; 0.98)	0.71
Higher prevalence of LBW	6	18879	0.88 (0.84; 0.91)		0.88 (0.84; 0.91)	
At 3 months of age						
Lower prevalence of LBW	3	7024	0.78 (0.61; 0.99)	0.42	0.78 (0.61; 0.99)	0.40
Higher prevalence of LBW	4	17945	0.86 (0.82; 0.90)		0.87 (0.77; 0.99)	
At 6 months of age						
Lower prevalence of LBW	3	7075	0.95 (0.80; 1.13)	0.75	0.93 (0.71; 1.22)	0.71
Higher prevalence of LBW	6	12056	0.98 (0.91; 1.04)		0.98 (0.91; 1.04)	
At 12 months of age						
Lower prevalence of LBW	3	4241	0.94 (0.80; 1.11)	0.70	0.85 (0.58; 1.23)	0.48
Higher prevalence of LBW	4	12282	0.97 (0.93; 1.02)		0.97 (0.93; 1.02)	
At 18 months of age						
Lower prevalence of LBW	2	788	0.68 (0.47; 0.97)	0.11	0.70 (0.45; 1.08)	0.21
Higher prevalence of LBW	3	2137	0.93 (0.80; 1.07)		0.94 (0.79; 1.14)	
At 24 months of age						
Lower prevalence of LBW	0	0	N/A (no studies available)	N/A	N/A (no studies available)	N/A
Higher prevalence of LBW	4	8713	1.00 (0.96; 1.05)		1.00 (0.96; 1.05)	
LBW prevalence not known	1	1360	1.08 (0.86; 1.36)		1.08 (0.86; 1.36)	

CI = Confidence interval; IFA = Iron and Folic Acid supplements; LBW = Low birthweight; MMS = Multiple Micronutrient supplements; N/A = Not applicable

The table shows the generic inverse variance weighted pooled risk ratios with their corresponding 95% confidence intervals comparing MMS and IFA intervention groups.

Supplemental Table 2.12 – Effect of MMS vs IFA on wasting (WLZ < -2) stratified by supplementation in pregnancy vs pregnancy and postpartum, and by lower vs higher prevalence rates of low birthweight (LBW)

Effect of MMS vs IFA on infant/child wasting at each age	n of studies	n of participants	Risk Ratio (95% CI) Fixed effects model	p value (subgroup differences)	Risk Ratio (95% CI) Random effects model	p value (subgroup differences)
A. Stratified by supplementation in pregnancy vs pregnancy and postpartum						
At 3 months of age						
Pregnancy	2	1502	1.11 (0.76; 1.64)	0.37	1.11 (0.76; 1.64)	0.37
Pregnancy and postpartum	5	23186	0.93 (0.84; 1.03)		0.93 (0.84; 1.03)	
At 6 months of age						
Pregnancy	3	1932	0.91 (0.62; 1.32)	0.43	0.91 (0.62; 1.32)	0.43
Pregnancy and postpartum	6	16848	1.06 (0.95; 1.18)		1.06 (0.95; 1.18)	
At 12 months of age						
Pregnancy	1	1525	0.96 (0.72; 1.29)	0.97	0.96 (0.72; 1.29)	0.97
Pregnancy and postpartum	6	14772	0.97 (0.89; 1.05)		0.97 (0.89; 1.05)	
At 18 months of age						
Pregnancy	2	1694	1.01 (0.77; 1.34)	0.12	1.01 (0.77; 1.34)	0.12
Pregnancy and postpartum	3	1228	0.68 (0.44; 1.04)		0.68 (0.44; 1.04)	
At 24 months of age						
Pregnancy	2	2806	0.94 (0.73; 1.21)	0.58	0.94 (0.73; 1.21)	0.58
Pregnancy and postpartum	3	7004	1.01 (0.93; 1.11)		1.01 (0.93; 1.11)	
B. Stratified by lower (≤ 11.9%) and higher prevalence (>11.9%) rates of LBW						
At birth						
Lower prevalence of LBW	3	6228	0.89 (0.81; 0.99)	0.78	0.89 (0.81; 0.99)	0.79
Higher prevalence of LBW	5	15339	0.91 (0.84; 0.99)		0.91 (0.82; 1.02)	
At 3 months of age						
Lower prevalence of LBW	3	6785	0.97 (0.75; 1.26)	0.80	0.97 (0.75; 1.26)	0.80
Higher prevalence of LBW	4	17903	0.93 (0.84; 1.04)		0.93 (0.84; 1.04)	
At 6 months of age						
Lower prevalence of LBW	3	6851	1.01 (0.81; 1.26)	0.71	1.01 (0.81; 1.26)	0.71
Higher prevalence of LBW	6	11929	1.06 (0.93; 1.20)		1.06 (0.93; 1.20)	
At 12 months of age						
Lower prevalence of LBW	3	4211	0.88 (0.71; 1.11)	0.40	0.83 (0.57; 1.22)	0.41
Higher prevalence of LBW	4	12086	0.98 (0.90; 1.06)		0.98 (0.90; 1.06)	
At 18 months of age						
Lower prevalence of LBW	2	786	0.64 (0.40; 1.03)	0.11	0.64 (0.40; 1.03)	0.11
Higher prevalence of LBW	3	2136	1.00 (0.77; 1.31)		1.00 (0.77; 1.31)	
At 24 months of age						
Lower prevalence of LBW	0	0	N/A (no studies available)	N/A	N/A (no studies available)	N/A
Higher prevalence of LBW	4	8450	1.00 (0.92; 1.09)		1.00 (0.92; 1.09)	
LBW prevalence not known	1	1360	1.12 (0.71; 1.77)		1.12 (0.71; 1.77)	

CI = Confidence interval; IFA = Iron and Folic Acid supplements; LBW = Low birthweight; MMS = Multiple Micronutrient supplements; N/A = Not applicable

The table shows the generic inverse variance weighted pooled risk ratios with their corresponding 95% confidence intervals comparing MMS and IFA intervention groups.

Supplemental Table 2.13 – Effect of MMS vs IFA on small HC (HCAZ < -2) stratified by supplementation in pregnancy vs pregnancy and postpartum, and by lower vs higher prevalence rates of low birthweight (LBW)

Effect of MMS vs IFA on infant/child small HC at each age	n of studies	n of participants	Risk Ratio (95% CI) Fixed effects model	p value (subgroup differences)	Risk Ratio (95% CI) Random effects model	p value (subgroup differences)
A. Stratified by supplementation in pregnancy vs pregnancy and postpartum						
At 3 months of age						
Pregnancy	2	1515	1.02 (0.83; 1.25)	0.05	1.02 (0.83; 1.25)	0.30
Pregnancy and postpartum	4	23205	0.82 (0.77; 0.88)		0.84 (0.61; 1.14)	
At 6 months of age						
Pregnancy	3	1944	0.99 (0.85; 1.17)	0.88	0.99 (0.85; 1.17)	0.88
Pregnancy and postpartum	5	16790	0.98 (0.90; 1.07)		0.98 (0.90; 1.07)	
At 12 months of age						
Pregnancy	1	1538	1.03 (0.91; 1.16)	0.23	1.03 (0.91; 1.16)	0.23
Pregnancy and postpartum	5	14624	0.95 (0.89; 1.01)		0.95 (0.89; 1.01)	
At 18 months of age						
Pregnancy	1	259	0.79 (0.39; 1.63)	0.75	0.79 (0.39; 1.63)	0.75
Pregnancy and postpartum	3	1228	0.90 (0.67; 1.20)		0.90 (0.67; 1.20)	
At 24 months of age						
Pregnancy	0	0	N/A (no studies available)	N/A	N/A (no studies available)	N/A
Pregnancy and postpartum	2	7136	1.00 (0.93; 1.06)		1.00 (0.93; 1.06)	
B. Stratified by lower (≤ 11.9%) and higher prevalence (>11.9%) rates of LBW						
At birth						
Lower prevalence of LBW	3	8570	0.84 (0.72: 0.97)	0.51	0.84 (0.72: 0.97)	0.43
Higher prevalence of LBW	5	18377	0.88 (0.84; 0.93)		0.91 (0.78; 1.07)	
At 3 months of age						
Lower prevalence of LBW	2	6656	0.93 (0.68; 1.26)	0.51	0.82 (0.33; 2.05)	0.89
Higher prevalence of LBW	4	18064	0.84 (0.78; 0.89)		0.87 (0.76; 1.00)	
At 6 months of age						
Lower prevalence of LBW	2	6725	1.11 (0.85; 1.45)	0.35	1.08 (0.75; 1.56)	0.58
Higher prevalence of LBW	6	12009	0.97 (0.90; 1.05)		0.97 (0.90; 1.05)	
At 12 months of age						
Lower prevalence of LBW	2	3898	0.91 (0.70; 1.18)	0.67	0.91 (0.65; 1.27)	0.73
Higher prevalence of LBW	4	12264	0.97 (0.91; 1.02)		0.97 (0.91; 1.02)	
At 18 months of age						
Lower prevalence of LBW	2	786	0.80 (0.57; 1.12)	0.34	0.80 (0.57; 1.12)	0.34
Higher prevalence of LBW	2	701	1.05 (0.68; 1.62)		1.05 (0.68; 1.62)	
At 24 months of age						
Lower prevalence of LBW	0	0	N/A (no studies available)	N/A	N/A (no studies available)	N/A
Higher prevalence of LBW	2	7136	1.00 (0.93; 1.06)		1.00 (0.93; 1.06)	

CI = Confidence interval; HC = Head circumference; IFA = Iron and Folic Acid supplements; LBW = Low birthweight; MMS = Multiple Micronutrient supplements; N/A = Not applicable

The table shows the generic inverse variance weighted pooled risk ratios with their corresponding 95% confidence intervals comparing MMS and IFA intervention groups.

Supplemental Table 2.14 – Effect of MMS vs IFA on low MUAC (MUACZ < -2) stratified by supplementation in pregnancy vs pregnancy and postpartum, and by lower vs higher prevalence rates of low birthweight (LBW)

Effect of MMS vs IFA on infant/child low MUAC at each age	n of studies	n of participants	Risk Ratio (95% CI) Fixed effects model	p value (subgroup differences)	Risk Ratio (95% CI) Random effects model	p value (subgroup differences)
A. Stratified by supplementation in pregnancy vs pregnancy and postpartum						
At 3 months of age						
Pregnancy	1	304	1.03 (0.06, 16.27)	0.93	1.03 (0.06, 16.27)	0.93
Pregnancy and postpartum	5	20429	0.90 (0.82; 0.99)		0.90 (0.82; 0.99)	
At 6 months of age						
Pregnancy	2	603	0.79 (0.22, 2.86)	0.73	1.03 (0.09, 11.40)	0.98
Pregnancy and postpartum	6	14779	0.99 (0.88; 1.12)		0.99 (0.88; 1.12)	
At 12 months of age						
Pregnancy	0	0	N/A (no studies available)	N/A	N/A (no studies available)	N/A
Pregnancy and postpartum	6	14784	0.99 (0.90; 1.09)		0.94 (0.74; 1.21)	
At 18 months of age						
Pregnancy	1	259	0.99 (0.20; 4.83)	0.91	0.99 (0.20; 4.83)	0.91
Pregnancy and postpartum	3	1230	0.90 (0.50; 1.63)		0.90 (0.50; 1.63)	
At 24 months of age						
Pregnancy	0	0	N/A (no studies available)	N/A	N/A (no studies available)	N/A
Pregnancy and postpartum	3	7299	0.92 (0.83; 1.02)		0.92 (0.83; 1.02)	
B. Stratified by lower (≤ 11.9%) and higher prevalence (>11.9%) rates of LBW						
At birth						
Lower prevalence of LBW	N/A	N/A	N/A	N/A	N/A	N/A
Higher prevalence of LBW	N/A	N/A	N/A		N/A	
At 3 months of age						
Lower prevalence of LBW	3	3844	0.75 (0.50; 1.13)	0.38	0.75 (0.50; 1.13)	0.38
Higher prevalence of LBW	3	16889	0.91 (0.83; 1.00)		0.91 (0.83; 1.00)	
At 6 months of age						
Lower prevalence of LBW	3	4697	1.08 (0.80; 1.46)	0.53	1.08 (0.80; 1.46)	0.53
Higher prevalence of LBW	5	10685	0.97 (0.85; 1.11)		0.97 (0.85; 1.11)	
At 12 months of age						
Lower prevalence of LBW	3	3951	0.95 (0.73; 1.23)	0.75	0.78 (0.45; 1.37)	0.42
Higher prevalence of LBW	3	10833	1.00 (0.90; 1.10)		1.03 (0.71; 1.48)	
At 18 months of age						
Lower prevalence of LBW	2	788	0.70 (0.34; 1.44)	0.27	0.70 (0.34; 1.44)	0.27
Higher prevalence of LBW	2	701	1.32 (0.56; 3.11)		1.32 (0.56; 3.11)	
At 24 months of age						
Lower prevalence of LBW	0	0	N/A (no studies available)	N/A	N/A (no studies available)	N/A
Higher prevalence of LBW	3	7299	0.92 (0.83; 1.02)		0.92 (0.83; 1.02)	

CI = Confidence interval; IFA = Iron and Folic Acid supplements; LBW = Low birthweight; MMS = Multiple Micronutrient supplements; MUAC = Mid upper arm circumference; N/A = Not applicable

The table shows the generic inverse variance weighted pooled risk ratios with their corresponding 95% confidence intervals comparing MMS and IFA intervention groups.

Supplemental Tables 3 –Sensitivity analyses limited to the 16 studies included in the WHO analyses

Supplemental Table 3.1 – Sensitivity analyses for length, LAZ, stunting (fixed effects)

	Length (cm)		LAZ score		Stunting (LAZ < -2)	
**Effect of MMS vs IFA**	**n of studies**	**Mean difference (95% CI), Fixed effects**	**n of studies**	**Mean difference (95% CI), Fixed effects**	**n of studies**	**Risk ratio (95% CI), Fixed effects**
**Birth**	12	0.05 (0.02, 0.08)	7	0.09 (0.06, 0.12)	7	0.90 (0.86, 0.94)
**3 months**	6	0.20 (0.13, 0.27)	6	0.10 (0.07, 0.13)	6	0.86 (0.82, 0.90)
**6 months**	8	0.12 (0.03, 0.20)	8	0.05 (0.01, 0.08)	8	1.01 (0.89, 1.14)
**12 months**	6	0.06 (-0.03, 0.16)	6	0.02 (-0.02, 0.06)	6	1.01 (0.96, 1.05)
**18 months**	4	0.18 (-0.06, 0.41)	4	0.00 (-0.08, 0.08)	4	1.00 (0.91, 1.10)
**24 months**	4	-0.06 (-0.20, 0.08)	4	-0.03 (-0.07, 0.02)	4	1.03 (0.98, 1.07)

CI = Confidence interval; cm = centimetres; IFA = Iron and Folic Acid supplements; LAZ = Length-for-age Z-score; MMS = Multiple Micronutrient Supplements
The table shows the generic inverse variance weighted pooled mean differences or pooled risk ratios with their corresponding 95% confidence intervals comparing MMS and IFA intervention groups.

Supplemental Table 3.2 – Sensitivity analyses for HC, HCAZ, small HC (fixed effects)

	HC (cm)		HCAZ score		Small HC (HCAZ < -2)	
**Effect of MMS vs IFA**	**n of studies**	**Mean difference (95% CI), Fixed effects**	**n of studies**	**Mean difference (95% CI), Fixed effects**	**n of studies**	**Risk ratio (95% CI), Fixed effects**
**Birth**	11	0.14 (0.10, 0.18)	6	0.13 (0.09, 0.16)	7	0.88 (0.84, 0.92)
**3 months**	5	0.15 (0.11, 0.18)	5	0.12 (0.09, 0.15)	5	0.83 (0.78, 0.89)
**6 months**	7	0.09 (0.04, 0.13)	7	0.06 (0.03, 0.10)	7	0.97 (0.90, 1.05)
**12 months**	5	0.05 (0.00, 0.10)	5	0.02 (-0.01, 0.06)	5	0.96 (0.91, 1.01)
**18 months**	3	-0.09 (-0.23, 0.06)	3	0.04 (-0.06, 0.14)	3	0.88 (0.67, 1,15)
**24 months**	2	0.01 (-0.06, 0.08)	2	0.00 (-0.04, 0.05)	2	1.00 (0.93, 1.06)

CI = Confidence interval; cm = centimetres; IFA = Iron and Folic Acid supplements; HC = Head circumference; HCAZ = Head circumference Z-score; MMS = Multiple Micronutrient Supplements
The table shows the generic inverse variance weighted pooled mean differences or pooled risk ratios with their corresponding 95% confidence intervals comparing MMS and IFA intervention groups.

Supplemental Table 3.3 – Sensitivity analyses for weight, WAZ, underweight (fixed effects)

	Weight (Kg)		WAZ score		Underweight (WAZ < -2)	
**Effect of MMS vs IFA**	**n of studies**	**Mean difference (95% CI), Fixed effects**	**n of studies**	**Mean difference (95% CI), Fixed effects**	**n of studies**	**Risk ratio (95% CI), Fixed effects**
**Birth**	16	0.04 (0.03, 0.04)	8	0.11 (0.08, 0.14)	8	0.87 (0.84, 0.91)
**3 months**	6	0.07 (0.05, 0.10)	6	0.12 (0.09, 0.15)	6	0.86 (0.82, 0.90)
**6 months**	8	0.05 (0.02, 0.08)	8	0.04 (0.01, 0.08)	8	0.97 (0.91, 1.04)
**12 months**	6	0.02 (-0.01, 0.06)	6	0.02 (-0.01, 0.06)	6	0.97 (0.92, 1.01)
**18 months**	4	0.09 (-0.00, 0.18)	4	0.05 (-0.03, 0.13)	4	0.88 (0.77, 1.01)
**24 months**	4	0.01 (-0.04, 0.05)	4	0.01 (-0.03, 0.05)	4	1.00 (0.96, 1.05)

CI = Confidence interval; IFA = Iron and Folic Acid supplements; kg = kilogram; MMS = Multiple Micronutrient Supplements; WAZ = Weight-for-age Z-score
The table shows the generic inverse variance weighted pooled mean differences or pooled risk ratios with their corresponding 95% confidence intervals comparing MMS and IFA intervention groups.

Supplemental Table 3.4 – Sensitivity analyses for WLZ, wasting (fixed effects)

	WLZ score		Wasting (WLZ < -2)	
**Effect of MMS vs IFA**	**n of studies**	**Mean difference (95% CI), Fixed effects**	**n of studies**	**Risk ratio (95% CI), Fixed effects**
**Birth**[1]	7	0.08 (0.05, 0.11)	7	0.91 (0.84, 0.98)
**3 months**	6	0.04 (0.01, 0.08)	6	0.93 (0.84, 1.04)
**6 months**	8	0.00 (-0.04, 0.04)	8	1.06 (0.94, 1.19)
**12 months**	6	0.03 (-0.02, 0.07)	6	0.97 (0.89, 1.05)
**18 months**	4	0.05 (-0.05, 0.15)	4	0.92 (0.72, 1.16)
**24 months**	4	0.00 (-0.04, 0.04)	4	1.00 (0.92, 1.09)

[1] Body Mass Index Z-score (BMIZ) for 4 studies and Weight-for-length Z-score (WLZ) for 3 studies
CI = Confidence interval; IFA = Iron and Folic Acid supplements; MMS = Multiple Micronutrient Supplements; WLZ = Weight-for-length Z-score
The table shows the generic inverse variance weighted pooled mean differences or pooled risk ratios with their corresponding 95% confidence intervals comparing MMS and IFA intervention groups.

Supplemental Table 3.5 – Sensitivity analyses for MUAC, MUACZ, low MUAC (fixed effects)

	MUAC (cm)		MUACZ score		Low MUAC (MUACZ < -2)	
**Effect of MMS vs IFA**	**n of studies**	**Mean difference (95% CI), Fixed effects**	**n of studies**	**Mean difference (95% CI), Fixed effects**	**n of studies**	**Risk ratio (95% CI), Fixed effects**
**Birth**	5	0.11 (0.08, 0.13)	N/A	N/A	N/A	N/A
**3 months**	5	0.09 (0.07, 0.12)	5	0.09 (0.06, 0.11)	5	0.90 (0.82, 0.99)
**6 months**	7	0.03 (-0.01, 0.06)	7	0.03 (-0.00, 0.06)	7	0.97 (0.85, 1.10)
**12 months**	5	0.02 (-0.01, 0.06)	5	0.02 (-0.02, 0.05)	5	0.97 (0.89, 1.07)
**18 months**	3	0.07 (-0.04, 0.19)	3	0.07 (-0.03, 0.17)	3	0.93 (0.53, 1.64)
**24 months**	3	-0.01 (-0.05, 0.04)	3	-0.01 (-0.05, 0.03)	3	0.92 (0.83, 1.02)

CI = Confidence interval; cm=centimetres; IFA = Iron and Folic Acid supplements; MMS = Multiple Micronutrient Supplements; MUAC = Mid-upper arm circumference; MUACZ = Mid-upper arm circumference Z-score; N/A = Not applicable
The table shows the generic inverse variance weighted pooled mean differences or pooled risk ratios with their corresponding 95% confidence intervals comparing MMS and IFA intervention groups.

Supplemental Figures 3 – Leave-One-Out sensitivity analyses

Leave−One−Out Sensitivity Analysis

Length at Birth, Fixed

Study	Mean Difference	MD	95%−CI	P−value	Tau2	Tau	I2
Omitting Adu−Afarwuah 2015		0.05	[0.02; 0.08]	< 0.01	0.0116	0.1079	66%
Omitting Ashorn 2010		0.05	[0.02; 0.08]	< 0.01	0.0117	0.1080	66%
Omitting Bhutta 2009		0.05	[0.02; 0.08]	< 0.01	0.0117	0.1080	66%
Omitting Christian 2003		0.05	[0.02; 0.08]	< 0.01	0.0121	0.1101	67%
Omitting Fawzi 2007		0.05	[0.02; 0.08]	< 0.01	0.0116	0.1079	66%
Omitting Friis 2004		0.05	[0.02; 0.08]	< 0.01	0.0112	0.1059	65%
Omitting Liu 2013		0.15	[0.10; 0.21]	< 0.01	0.0045	0.0668	24%
Omitting Osrin 2005		0.05	[0.02; 0.08]	< 0.01	0.0111	0.1055	65%
Omitting Ramakrishnan 2003		0.05	[0.02; 0.08]	< 0.01	0.0119	0.1092	66%
Omitting Roberfroid 2008		0.05	[0.02; 0.08]	< 0.01	0.0106	0.1031	64%
Omitting Sunawang 2009		0.05	[0.02; 0.08]	< 0.01	0.0123	0.1111	67%
Omitting Tofail 2008		0.05	[0.02; 0.08]	< 0.01	0.0116	0.1078	66%
Omitting West 2014		0.01	[−0.02; 0.05]	0.44	0.0022	0.0471	15%
Omitting Zeng 2008		0.05	[0.02; 0.08]	< 0.01	0.0123	0.1107	67%
**Fixed effect model**		**0.05**	**[0.02; 0.08]**	**< 0.01**	**0.0113**	**0.1061**	**64%**

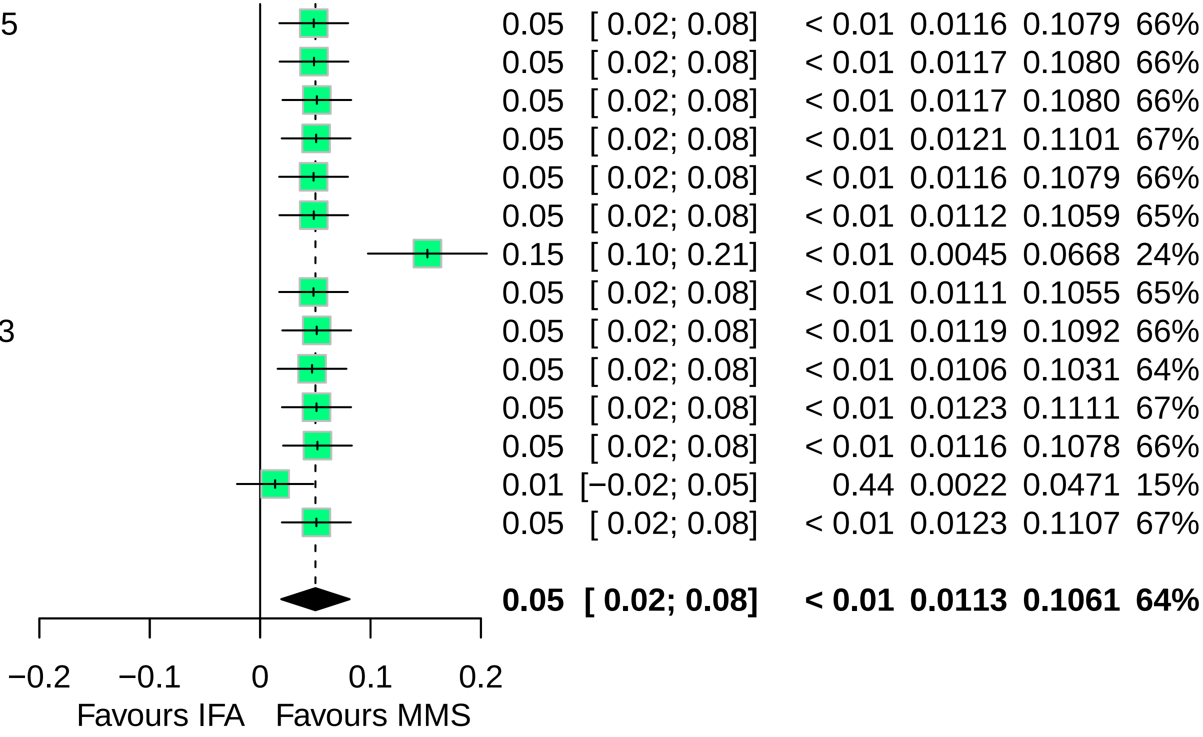

Leave−One−Out Sensitivity Analysis

Length at Birth, Random

Study	MD	95%−CI	P−value	Tau2	Tau	I2
Omitting Adu−Afarwuah 2015	0.08	[−0.00; 0.17]	0.06	0.0116	0.1079	66%
Omitting Ashorn 2010	0.09	[−0.00; 0.17]	0.06	0.0117	0.1080	66%
Omitting Bhutta 2009	0.10	[0.01; 0.19]	0.03	0.0117	0.1080	66%
Omitting Christian 2003	0.10	[0.01; 0.19]	0.04	0.0121	0.1101	67%
Omitting Fawzi 2007	0.08	[−0.01; 0.17]	0.07	0.0116	0.1079	66%
Omitting Friis 2004	0.08	[−0.00; 0.17]	0.06	0.0112	0.1059	65%
Omitting Liu 2013	0.12	[0.04; 0.20]	< 0.01	0.0045	0.0668	24%
Omitting Osrin 2005	0.08	[−0.01; 0.17]	0.07	0.0111	0.1055	65%
Omitting Ramakrishnan 2003	0.10	[0.01; 0.19]	0.03	0.0119	0.1092	66%
Omitting Roberfroid 2008	0.08	[−0.01; 0.16]	0.08	0.0106	0.1031	64%
Omitting Sunawang 2009	0.10	[0.01; 0.19]	0.04	0.0123	0.1111	67%
Omitting Tofail 2008	0.10	[0.01; 0.19]	0.02	0.0116	0.1078	66%
Omitting West 2014	0.05	[−0.02; 0.11]	0.17	0.0022	0.0471	15%
Omitting Zeng 2008	0.10	[0.01; 0.19]	0.04	0.0123	0.1107	67%
**Random effects model**	**0.09**	**[0.00; 0.18]**	**0.04**	**0.0113**	**0.1061**	**64%**

Mean Difference

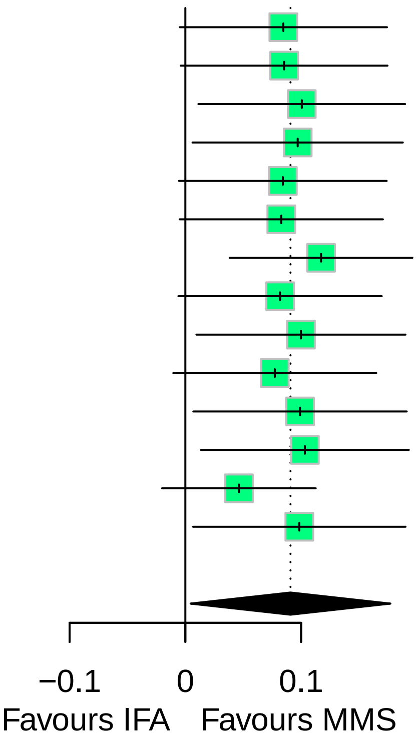

Leave−One−Out Sensitivity Analysis
Length at 3 Months, Fixed

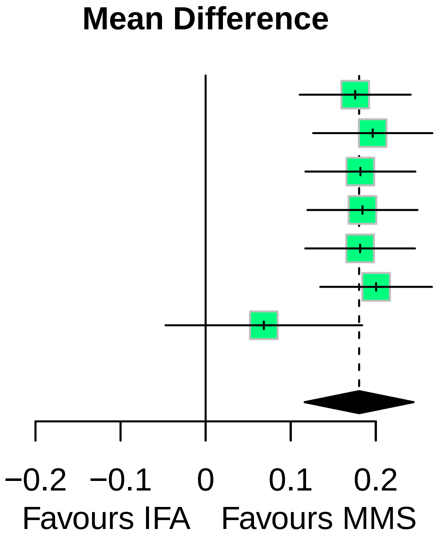

Study	MD	95%−CI	P−value	Tau2	Tau	I2
Omitting Adu−Afarwuah 2015	0.18	[0.11; 0.24]	< 0.01	0.0128	0.1132	51%
Omitting Fawzi 2007	0.20	[0.13; 0.27]	< 0.01	0.0190	0.1379	47%
Omitting Moore 2009	0.18	[0.12; 0.25]	< 0.01	0.0130	0.1139	53%
Omitting Roberfroid 2008	0.18	[0.12; 0.25]	< 0.01	0.0118	0.1086	50%
Omitting Sunawang 2009	0.18	[0.12; 0.25]	< 0.01	0.0129	0.1134	53%
Omitting Tofail 2008	0.20	[0.13; 0.27]	< 0.01	0	0	0%
Omitting West 2014	0.07	[−0.05; 0.18]	0.25	0.0026	0.0512	9%
**Fixed Effect Model**	**0.18**	**[0.12; 0.24]**	**< 0.01**	**0.0103**	**0.1015**	**44%**

Leave−One−Out Sensitivity Analysis
Length at 3 Months, Random

Study	MD	95%−CI	P−value	Tau2	Tau	I2
Omitting Adu−Afarwuah 2015	0.09	[−0.05; 0.23]	0.20	0.0128	0.1132	51%
Omitting Fawzi 2007	0.11	[−0.06; 0.28]	0.20	0.0190	0.1379	47%
Omitting Moore 2009	0.12	[−0.02; 0.26]	0.10	0.0130	0.1139	53%
Omitting Roberfroid 2008	0.13	[−0.01; 0.26]	0.07	0.0118	0.1086	50%
Omitting Sunawang 2009	0.12	[−0.02; 0.25]	0.09	0.0129	0.1134	53%
Omitting Tofail 2008	0.20	[0.13; 0.27]	< 0.01	0	0	0%
Omitting West 2014	0.07	[−0.06; 0.19]	0.32	0.0026	0.0512	9%
**Random effects model**	**0.12**	**[−0.01; 0.25]**	**0.06**	**0.0103**	**0.1015**	**44%**

Mean Difference

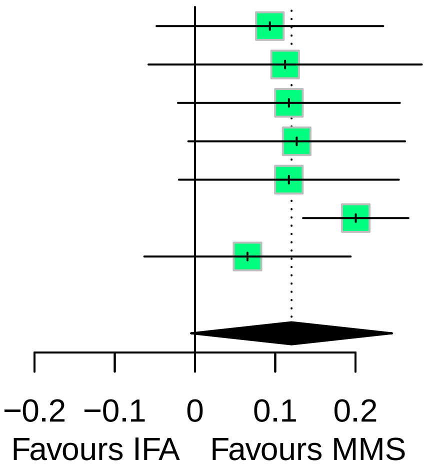

Leave−One−Out Sensitivity Analysis
Length at 6 Months, Fixed

Study	MD	95%−CI	P−value	Tau2	Tau	I2
Omitting Adu−Afarwuah 2015	0.08	[0.00; 0.15]	0.04	0	0	0%
Omitting Ashorn 2010 complete FU	0.08	[0.01; 0.16]	0.03	0	0	0%
Omitting Ashorn 2010 simplified FU	0.09	[0.02; 0.16]	0.02	0	0	0%
Omitting Fawzi 2007	0.12	[0.03; 0.20]	< 0.01	0	0	0%
Omitting Moore 2009	0.09	[0.01; 0.16]	0.03	0	0	0%
Omitting Roberfroid 2008	0.08	[0.00; 0.16]	0.04	0	0	0%
Omitting Sunawang 2009	0.09	[0.01; 0.16]	0.03	0	0	0%
Omitting Tofail 2008	0.09	[0.01; 0.17]	0.02	0	0	0%
Omitting West 2014	0.02	[−0.08; 0.13]	0.66	0	0	0%
**Fixed Effect Model**	**0.08**	**[0.01; 0.16]**	**0.03**	**0**	**0**	**0%**

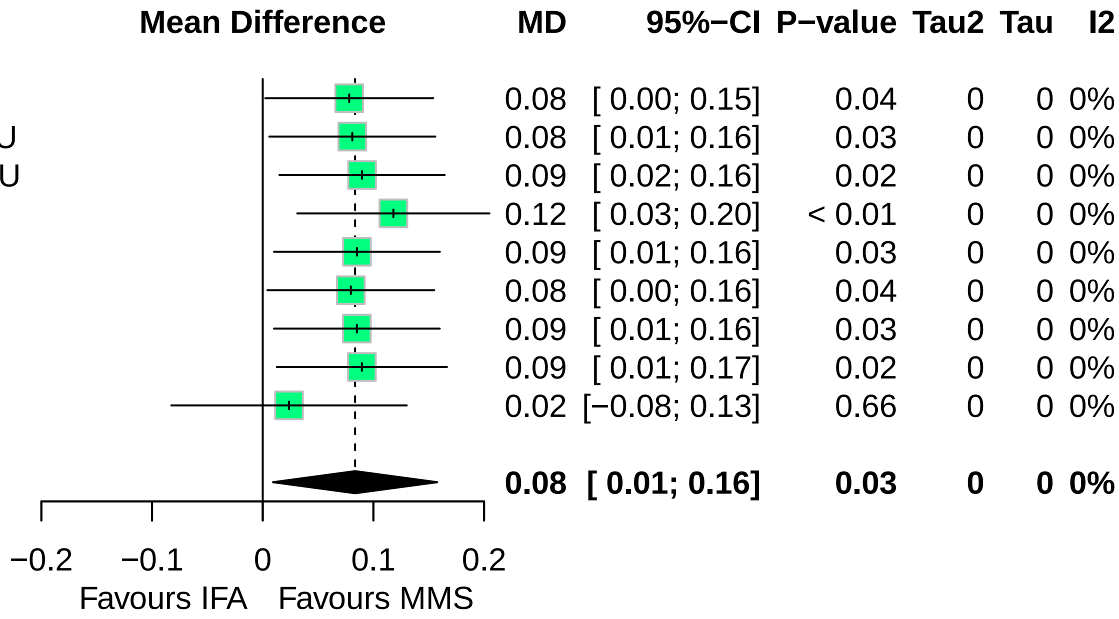

Leave−One−Out Sensitivity Analysis
Length at 6 Months, Random

Study	MD	95%−CI	P−value	Tau2	Tau	I2
Omitting Adu−Afarwuah 2015	0.08	[0.00; 0.15]	0.04	0	0	0%
Omitting Ashorn 2010 complete FU	0.08	[0.01; 0.16]	0.03	0	0	0%
Omitting Ashorn 2010 simplified FU	0.09	[0.02; 0.16]	0.02	0	0	0%
Omitting Fawzi 2007	0.12	[0.03; 0.20]	< 0.01	0	0	0%
Omitting Moore 2009	0.09	[0.01; 0.16]	0.03	0	0	0%
Omitting Roberfroid 2008	0.08	[0.00; 0.16]	0.04	0	0	0%
Omitting Sunawang 2009	0.09	[0.01; 0.16]	0.03	0	0	0%
Omitting Tofail 2008	0.09	[0.01; 0.17]	0.02	0	0	0%
Omitting West 2014	0.02	[−0.08; 0.13]	0.66	0	0	0%
**Random effects model**	**0.08**	**[0.01; 0.16]**	**0.03**	**0**	**0**	**0%**

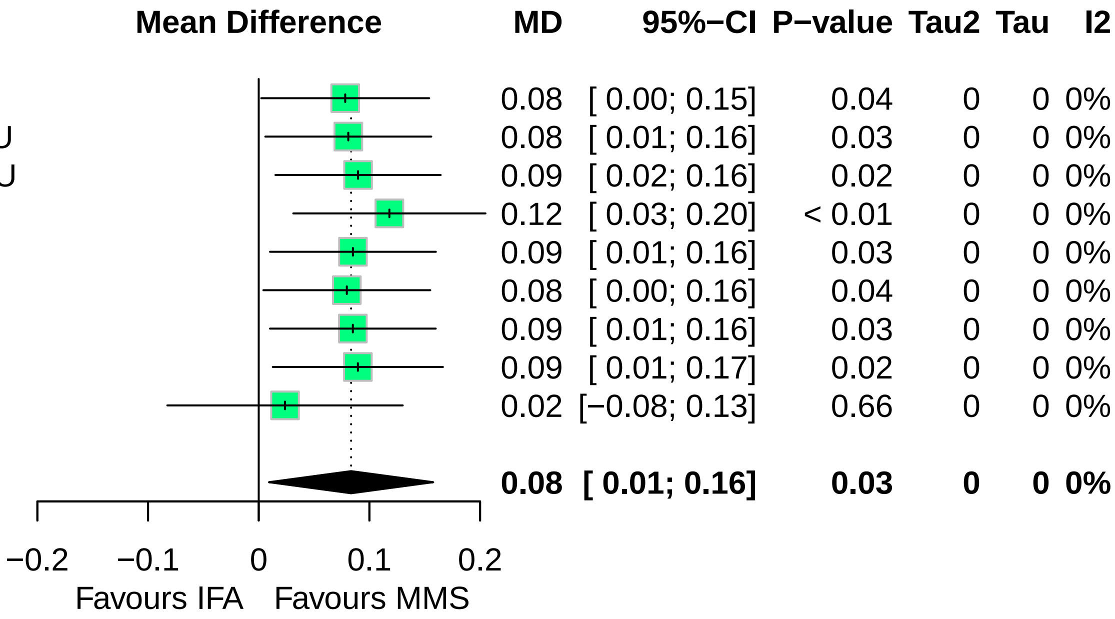

Leave−One−Out Sensitivity Analysis

Length at 12 Months, Fixed

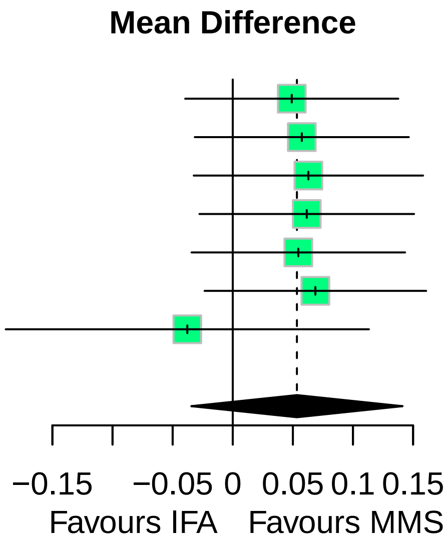

Study	MD	95%−CI	P−value	Tau2	Tau	I2
Omitting Adu−Afarwuah 2015	0.05	[−0.04; 0.14]	0.28	0	0	0%
Omitting Ashorn 2010 complete FU	0.06	[−0.03; 0.15]	0.21	0	0	0%
Omitting Fawzi 2007	0.06	[−0.03; 0.16]	0.20	0	0	0%
Omitting Roberfroid 2008	0.06	[−0.03; 0.15]	0.18	0	0	0%
Omitting Sunawang 2009	0.05	[−0.03; 0.14]	0.23	0	0	0%
Omitting Tofail 2008	0.07	[−0.02; 0.16]	0.14	0	0	0%
Omitting West 2014	−0.04	[−0.19; 0.11]	0.62	0	0	0%
**Fixed Effect Model**	**0.05**	**[−0.03; 0.14]**	**0.23**	**0**	**0**	**0%**

Leave−One−Out Sensitivity Analysis
Length at 12 Months, Random

Study	MD	95%−CI	P−value	Tau2	Tau	I2
Omitting Adu−Afarwuah 2015	0.05	[−0.04; 0.14]	0.28	0	0	0%
Omitting Ashorn 2010 complete FU	0.06	[−0.03; 0.15]	0.21	0	0	0%
Omitting Fawzi 2007	0.06	[−0.03; 0.16]	0.20	0	0	0%
Omitting Roberfroid 2008	0.06	[−0.03; 0.15]	0.18	0	0	0%
Omitting Sunawang 2009	0.05	[−0.03; 0.14]	0.23	0	0	0%
Omitting Tofail 2008	0.07	[−0.02; 0.16]	0.14	0	0	0%
Omitting West 2014	−0.04	[−0.19; 0.11]	0.62	0	0	0%
**Random effects model**	**0.05**	**[−0.03; 0.14]**	**0.23**	**0**	**0**	**0%**

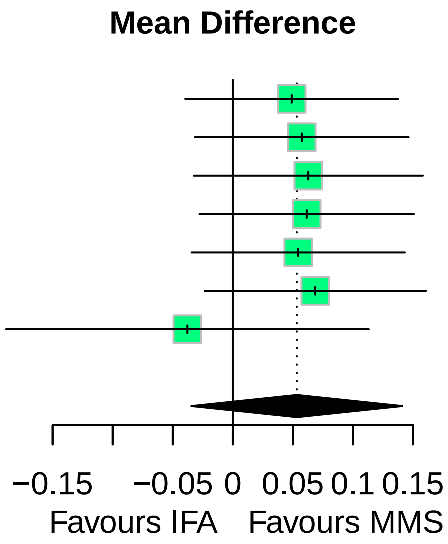

Leave−One−Out Sensitivity Analysis
Length at 18 Months, Fixed

Study	MD	95%−CI	P−value	Tau2	Tau	I2
Omitting Adu−Afarwuah 2015	0.12	[−0.15; 0.39]	0.38	0	0	0%
Omitting Ashorn 2010 complete FU	0.25	[0.00; 0.50]	0.05	0	0	0%
Omitting Ashorn 2010 simplified FU	0.24	[−0.00; 0.48]	0.05	0	0	0%
Omitting Fawzi 2007	0.18	[−0.06; 0.41]	0.14	0	0	0%
Omitting Tofail 2008	0.20	[−0.10; 0.50]	0.19	0.0201	0.1418	16%
**Fixed Effect Model**	**0.20**	**[−0.03; 0.43]**	**0.09**	**0**	**0**	**0%**

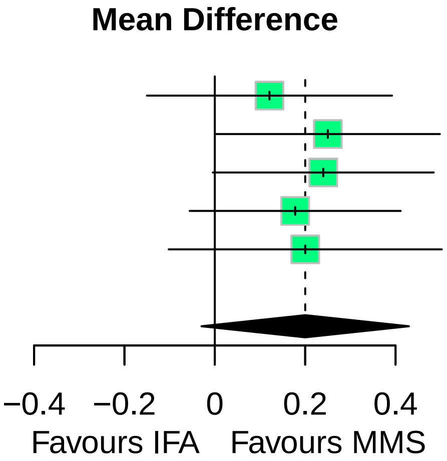

Leave−One−Out Sensitivity Analysis
Length at 18 Months, Random

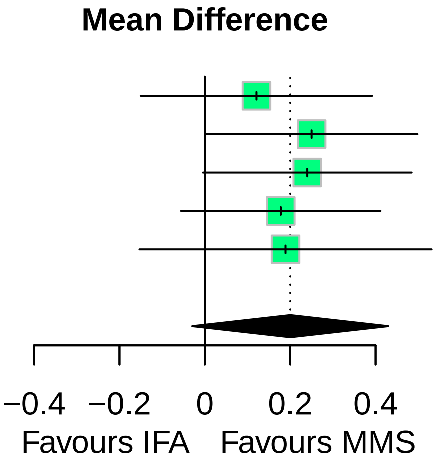

Study	MD	95%−CI	P−value	Tau2	Tau	I2
Omitting Adu−Afarwuah 2015	0.12	[−0.15; 0.39]	0.38	0	0	0%
Omitting Ashorn 2010 complete FU	0.25	[0.00; 0.50]	0.05	0	0	0%
Omitting Ashorn 2010 simplified FU	0.24	[−0.00; 0.48]	0.05	0	0	0%
Omitting Fawzi 2007	0.18	[−0.06; 0.41]	0.14	0	0	0%
Omitting Tofail 2008	0.19	[−0.15; 0.53]	0.28	0.0201	0.1418	16%
**Random effects model**	**0.20**	**[−0.03; 0.43]**	**0.09**	**0**	**0**	**0%**

Leave−One−Out Sensitivity Analysis
Length at 24 Months, Fixed

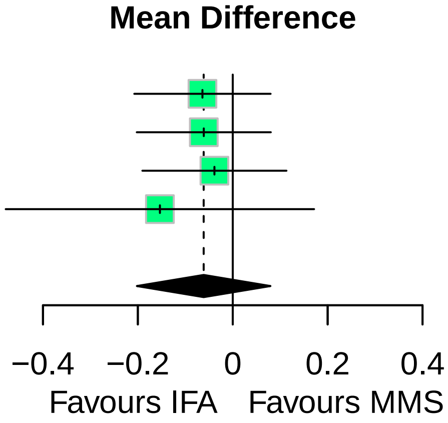

Study	MD	95%−CI	P−value	Tau2	Tau	I2
Omitting Ashorn 2010 complete FU	−0.06	[−0.21; 0.08]	0.38	0	0	0%
Omitting Roberfroid 2008	−0.06	[−0.20; 0.08]	0.40	0	0	0%
Omitting Tofail 2008	−0.04	[−0.19; 0.11]	0.62	0	0	0%
Omitting West 2014	−0.15	[−0.48; 0.17]	0.35	0	0	0%
**Fixed Effect Model**	**−0.06**	**[−0.20; 0.08]**	**0.39**	**0**	**0**	**0%**

Leave−One−Out Sensitivity Analysis
Length at 24 Months, Random

Study	MD	95%−CI	P−value	Tau2	Tau	I2
Omitting Ashorn 2010 complete FU	−0.06	[−0.21; 0.08]	0.38	0	0	0%
Omitting Roberfroid 2008	−0.06	[−0.20; 0.08]	0.40	0	0	0%
Omitting Tofail 2008	−0.04	[−0.19; 0.11]	0.62	0	0	0%
Omitting West 2014	−0.15	[−0.48; 0.17]	0.35	0	0	0%
**Random effects model**	**−0.06**	**[−0.20; 0.08]**	**0.39**	**0**	**0**	**0%**

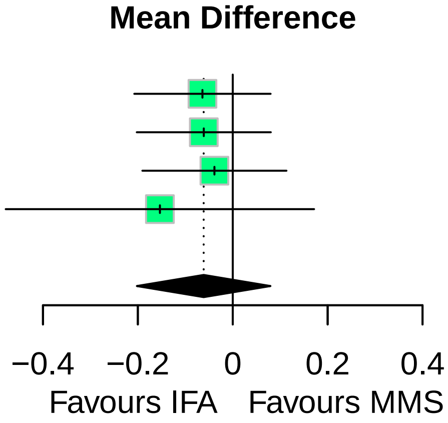

Leave−One−Out Sensitivity Analysis

HC at Birth, Fixed

Study	Mean Difference	MD	95%−CI	P−value	Tau2	Tau	I2
Omitting Adu−Afarwuah 2015		0.14	[0.10; 0.18]	< 0.01	0.0084	0.0915	60%
Omitting Ashorn 2010		0.14	[0.10; 0.17]	< 0.01	0.0093	0.0964	62%
Omitting Bhutta 2009		0.13	[0.10; 0.17]	< 0.01	0.0088	0.0939	62%
Omitting Christian 2003		0.14	[0.10; 0.18]	< 0.01	0.0081	0.0900	58%
Omitting Fawzi 2007		0.14	[0.10; 0.18]	< 0.01	0.0100	0.1000	61%
Omitting Friis 2004		0.13	[0.10; 0.17]	< 0.01	0.0090	0.0951	62%
Omitting Osrin 2005		0.13	[0.10; 0.17]	< 0.01	0.0089	0.0945	62%
Omitting Roberfroid 2008		0.14	[0.10; 0.18]	< 0.01	0.0082	0.0906	59%
Omitting Sunawang 2009		0.14	[0.11; 0.18]	< 0.01	0.0079	0.0887	57%
Omitting Tofail 2008		0.13	[0.09; 0.17]	< 0.01	0.0077	0.0876	58%
Omitting West 2014		0.06	[0.01; 0.12]	0.03	0.0044	0.0661	33%
Omitting Zeng 2008		0.15	[0.11; 0.18]	< 0.01	0.0048	0.0689	45%
**Fixed effect model**		**0.14**	**[0.10; 0.17]**	**< 0.01**	**0.0082**	**0.0905**	**58%**

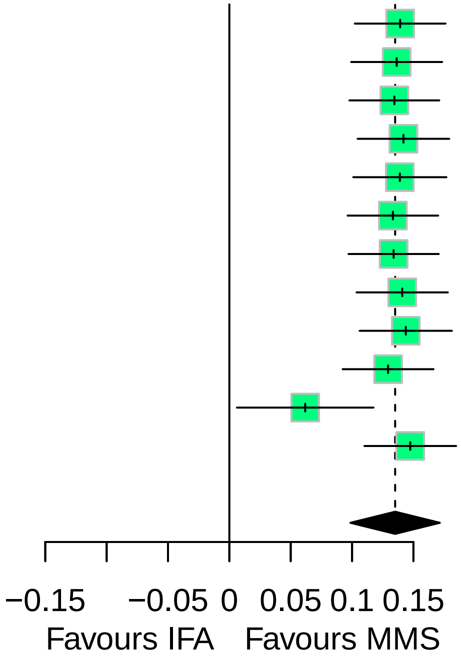

Leave−One−Out Sensitivity Analysis
HC at Birth, Random

Study	MD	95%−CI	P−value	Tau2	Tau	I2
Omitting Adu−Afarwuah 2015	0.10	[0.02; 0.17]	0.01	0.0084	0.0915	60%
Omitting Ashorn 2010	0.09	[0.01; 0.17]	0.03	0.0093	0.0964	62%
Omitting Bhutta 2009	0.09	[0.01; 0.16]	0.02	0.0088	0.0939	62%
Omitting Christian 2003	0.10	[0.02; 0.18]	0.01	0.0081	0.0900	58%
Omitting Fawzi 2007	0.09	[0.01; 0.17]	0.03	0.0100	0.1000	61%
Omitting Friis 2004	0.08	[0.00; 0.16]	0.04	0.0090	0.0951	62%
Omitting Osrin 2005	0.08	[0.01; 0.16]	0.03	0.0089	0.0945	62%
Omitting Roberfroid 2008	0.10	[0.02; 0.18]	0.01	0.0082	0.0906	59%
Omitting Sunawang 2009	0.10	[0.02; 0.18]	< 0.01	0.0079	0.0887	57%
Omitting Tofail 2008	0.07	[−0.00; 0.15]	0.05	0.0077	0.0876	58%
Omitting West 2014	0.07	[−0.00; 0.14]	0.05	0.0044	0.0661	33%
Omitting Zeng 2008	0.11	[0.05; 0.18]	< 0.01	0.0048	0.0689	45%
**Random effects model**	**0.09**	**[0.02; 0.16]**	**0.01**	**0.0082**	**0.0905**	**58%**

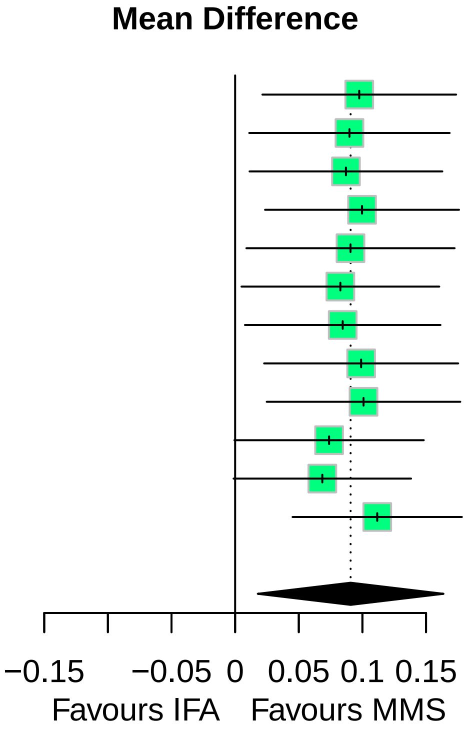

Leave−One−Out Sensitivity Analysis
HC at 3 Months, Fixed

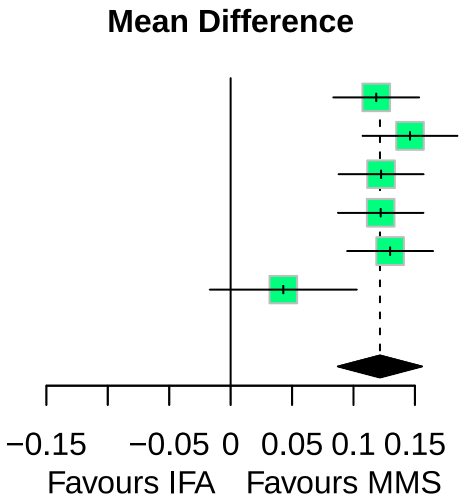

Study	MD	95%−CI	P−value	Tau2	Tau	I2
Omitting Adu−Afarwuah 2015	0.12	[0.08; 0.15]	< 0.01	0.0075	0.0867	73%
Omitting Fawzi 2007	0.15	[0.11; 0.18]	< 0.01	0.0064	0.0797	53%
Omitting Moore 2009	0.12	[0.09; 0.16]	< 0.01	0.0075	0.0864	74%
Omitting Roberfroid 2008	0.12	[0.09; 0.16]	< 0.01	0.0078	0.0881	75%
Omitting Tofail 2008	0.13	[0.09; 0.16]	< 0.01	0.0039	0.0627	59%
Omitting West 2014	0.04	[−0.02; 0.10]	0.16	0.0031	0.0558	32%
**Fixed Effect Model**	**0.12**	**[0.09; 0.16]**	**< 0.01**	**0.0066**	**0.0810**	**68%**

Leave−One−Out Sensitivity Analysis
HC at 3 Months, Random

Study	MD	95%−CI	P−value	Tau2	Tau	I2
Omitting Adu−Afarwuah 2015	0.06	[−0.03; 0.16]	0.20	0.0075	0.0867	73%
Omitting Fawzi 2007	0.10	[0.00; 0.20]	0.05	0.0064	0.0797	53%
Omitting Moore 2009	0.08	[−0.01; 0.18]	0.08	0.0075	0.0864	74%
Omitting Roberfroid 2008	0.08	[−0.02; 0.18]	0.11	0.0078	0.0881	75%
Omitting Tofail 2008	0.11	[0.03; 0.19]	< 0.01	0.0039	0.0627	59%
Omitting West 2014	0.05	[−0.04; 0.14]	0.25	0.0031	0.0558	32%
**Random effects model**	**0.08**	**[−0.00; 0.17]**	**0.06**	**0.0066**	**0.0810**	**68%**

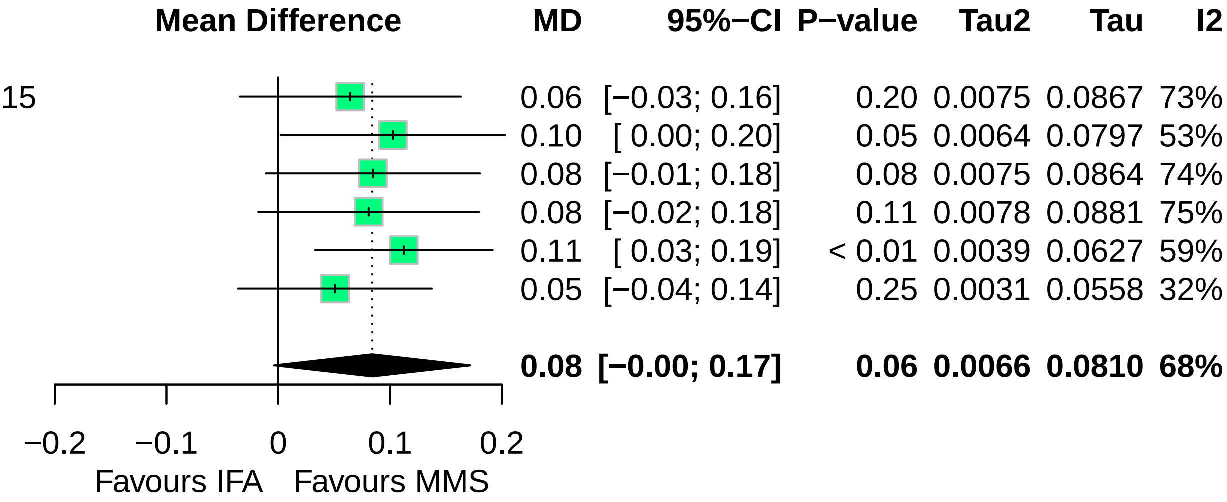

Leave−One−Out Sensitivity Analysis
HC at 6 Months, Fixed

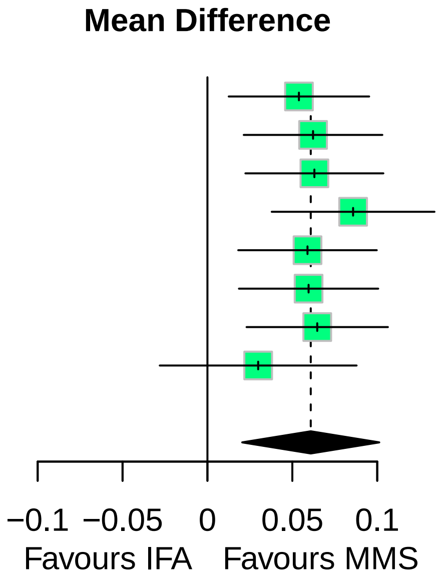

Study	MD	95%−CI	P−value	Tau2	Tau	I2
Omitting Adu−Afarwuah 2015	0.05	[0.01; 0.10]	0.01	0	0	0%
Omitting Ashorn 2010 complete FU	0.06	[0.02; 0.10]	< 0.01	0.0011	0.0329	21%
Omitting Ashorn 2010 simplified FU	0.06	[0.02; 0.10]	< 0.01	0.0006	0.0253	14%
Omitting Fawzi 2007	0.09	[0.04; 0.13]	< 0.01	0	0	0%
Omitting Moore 2009	0.06	[0.02; 0.10]	< 0.01	0.0009	0.0306	18%
Omitting Roberfroid 2008	0.06	[0.02; 0.10]	< 0.01	0.0012	0.0340	21%
Omitting Tofail 2008	0.06	[0.02; 0.11]	< 0.01	0.0009	0.0301	17%
Omitting West 2014	0.03	[−0.03; 0.09]	0.31	0	0	0%
**Fixed Effect Model**	**0.06**	**[0.02; 0.10]**	**< 0.01**	**0.0005**	**0.0224**	**10%**

Leave−One−Out Sensitivity Analysis
HC at 6 Months, Random

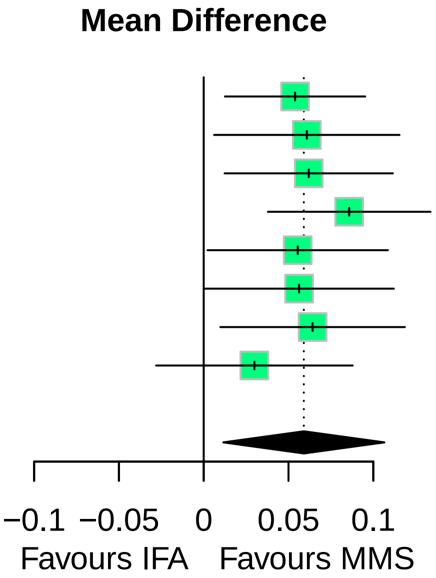

Study	MD	95%−CI	P−value	Tau2	Tau	I2
Omitting Adu−Afarwuah 2015	0.05	[0.01; 0.10]	0.01	0	0	0%
Omitting Ashorn 2010 complete FU	0.06	[0.01; 0.12]	0.03	0.0011	0.0329	21%
Omitting Ashorn 2010 simplified FU	0.06	[0.01; 0.11]	0.01	0.0006	0.0253	14%
Omitting Fawzi 2007	0.09	[0.04; 0.13]	< 0.01	0	0	0%
Omitting Moore 2009	0.06	[0.00; 0.11]	0.04	0.0009	0.0306	18%
Omitting Roberfroid 2008	0.06	[0.00; 0.11]	0.05	0.0012	0.0340	21%
Omitting Tofail 2008	0.06	[0.01; 0.12]	0.02	0.0009	0.0301	17%
Omitting West 2014	0.03	[−0.03; 0.09]	0.31	0	0	0%
**Random effects model**	**0.06**	**[0.01; 0.11]**	**0.01**	**0.0005**	**0.0224**	**10%**

Leave−One−Out Sensitivity Analysis
HC at 12 Months, Fixed

Study	Mean Difference	MD	95%−CI	P−value	Tau2	Tau	I2
Omitting Adu−Afarwuah 2015		0.04	[−0.00; 0.09]	0.06	0	0	0%
Omitting Ashorn 2010 complete FU		0.05	[0.00; 0.09]	0.04	0	0	0%
Omitting Fawzi 2007		0.05	[0.00; 0.10]	0.04	0	0	0%
Omitting Roberfroid 2008		0.04	[−0.00; 0.09]	0.05	0	0	0%
Omitting Tofail 2008		0.04	[−0.01; 0.09]	0.09	0	0	0%
Omitting West 2014		0.05	[−0.02; 0.13]	0.14	0	0	0%
**Fixed Effect Model**		**0.05**	**[0.00; 0.09]**	**0.04**	**0**	**0**	**0%**

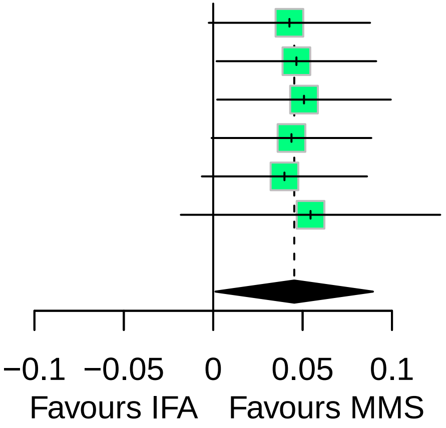

Leave−One−Out Sensitivity Analysis

HC at 12 Months, Random

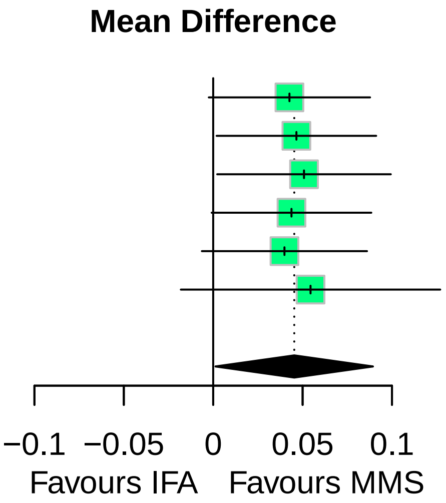

Study	MD	95%−CI	P−value	Tau2	Tau	I2
Omitting Adu−Afarwuah 2015	0.04	[−0.00; 0.09]	0.06	0	0	0%
Omitting Ashorn 2010 complete FU	0.05	[0.00; 0.09]	0.04	0	0	0%
Omitting Fawzi 2007	0.05	[0.00; 0.10]	0.04	0	0	0%
Omitting Roberfroid 2008	0.04	[−0.00; 0.09]	0.05	0	0	0%
Omitting Tofail 2008	0.04	[−0.01; 0.09]	0.09	0	0	0%
Omitting West 2014	0.05	[−0.02; 0.13]	0.14	0	0	0%
**Random effects model**	**0.05**	**[0.00; 0.09]**	**0.04**	**0**	**0**	**0%**

Leave−One−Out Sensitivity Analysis
HC at 18 Months, Fixed

Study	MD	95%−CI	P−value	Tau2	Tau	I2
Omitting Adu−Afarwuah 2015	−0.28	[−0.48; −0.08]	< 0.01	0.0659	0.2568	64%
Omitting Ashorn 2010 complete FU	0.07	[−0.10; 0.23]	0.43	0	0	0%
Omitting Ashorn 2010 simplified FU	−0.10	[−0.26; 0.05]	0.19	0.1252	0.3538	84%
Omitting Fawzi 2007	−0.09	[−0.23; 0.06]	0.24	0.0965	0.3107	84%
**Fixed Effect Model**	**−0.09**	**[−0.23; 0.05]**	**0.23**	**0.0783**	**0.2798**	**76%**

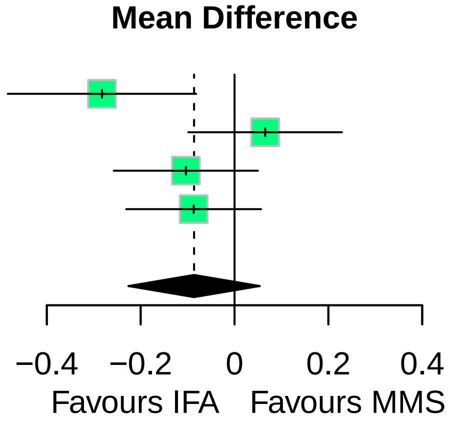

Leave−One−Out Sensitivity Analysis
HC at 18 Months, Random

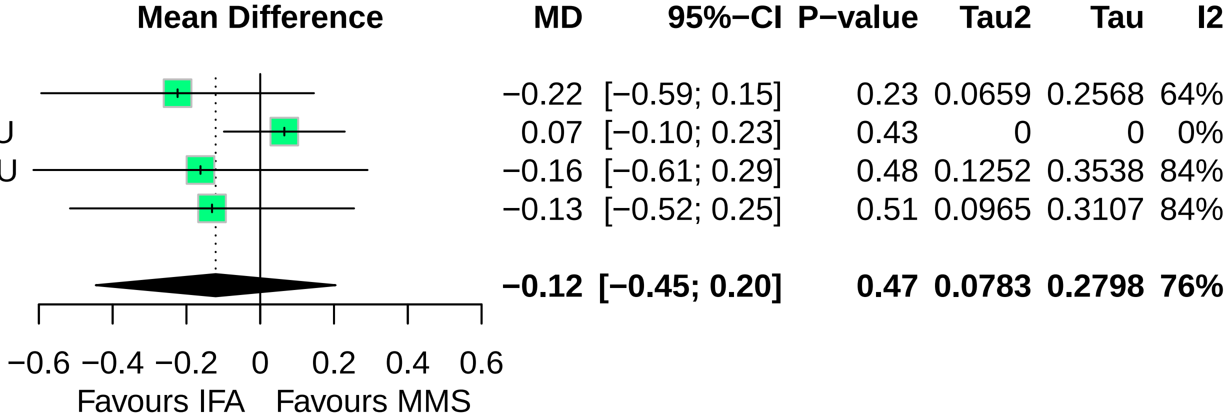

Study	MD	95%−CI	P−value	Tau2	Tau	I2
Omitting Adu−Afarwuah 2015	−0.22	[−0.59; 0.15]	0.23	0.0659	0.2568	64%
Omitting Ashorn 2010 complete FU	0.07	[−0.10; 0.23]	0.43	0	0	0%
Omitting Ashorn 2010 simplified FU	−0.16	[−0.61; 0.29]	0.48	0.1252	0.3538	84%
Omitting Fawzi 2007	−0.13	[−0.52; 0.25]	0.51	0.0965	0.3107	84%
**Random effects model**	**−0.12**	**[−0.45; 0.20]**	**0.47**	**0.0783**	**0.2798**	**76%**

Leave−One−Out Sensitivity Analysis
HC at 24 Months, Fixed

Study	Mean Difference	MD	95%−CI	P−value	Tau2	Tau	I2
Omitting Ashorn 2010 complete FU		0.02	[−0.05; 0.09]	0.56	.	.	.
Omitting West 2014		−0.20	[−0.51; 0.11]	0.21	.	.	.
**Fixed Effect Model**		**0.01**	**[−0.06; 0.08]**	**0.76**	**0.0107**	**0.1036**	**44%**

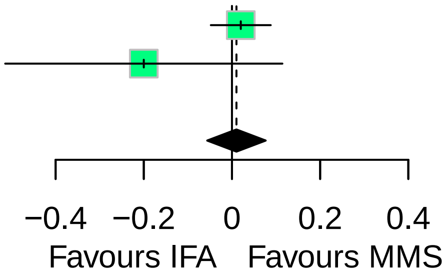

Leave−One−Out Sensitivity Analysis
HC at 24 Months, Random

Study	Mean Difference	MD	95%−CI	P−value	Tau2	Tau	I2
Omitting Ashorn 2010 complete FU		0.02	[−0.05; 0.09]	0.56	.	.	.
Omitting West 2014		−0.20	[−0.51; 0.11]	0.21	.	.	.
**Random effects model**		**−0.03**	**[−0.22; 0.15]**	**0.72**	**0.0107**	**0.1036**	**44%**

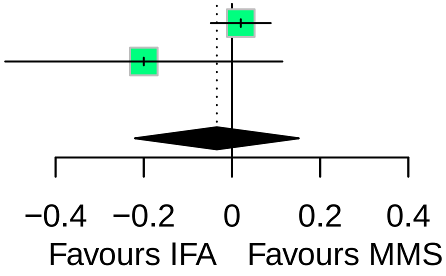

Leave−One−Out Sensitivity Analysis
Weight at Birth, Fixed

Study	MD	95%−CI	P−value	Tau2	Tau	I2
Omitting Adu−Afarwuah 2015	0.04	[0.03; 0.05]	< 0.01	0.0005	0.0232	69%
Omitting Ashorn 2010	0.04	[0.03; 0.05]	< 0.01	0.0005	0.0232	69%
Omitting Bhutta 2009	0.04	[0.03; 0.04]	< 0.01	0.0005	0.0230	69%
Omitting Christian 2003	0.04	[0.03; 0.05]	< 0.01	0.0005	0.0230	69%
Omitting Fawzi 2007	0.04	[0.03; 0.04]	< 0.01	0.0005	0.0233	68%
Omitting Friis 2004	0.04	[0.03; 0.05]	< 0.01	0.0005	0.0232	70%
Omitting Kaestel 2005	0.04	[0.03; 0.04]	< 0.01	0.0005	0.0229	69%
Omitting Liu 2013	0.05	[0.04; 0.06]	< 0.01	0.0002	0.0140	38%
Omitting Moore 2009	0.04	[0.03; 0.05]	< 0.01	0.0005	0.0230	69%
Omitting Osrin 2005	0.04	[0.03; 0.04]	< 0.01	0.0005	0.0227	68%
Omitting Ramakrishnan 2003	0.04	[0.03; 0.05]	< 0.01	0.0005	0.0229	69%
Omitting Roberfroid 2008	0.04	[0.03; 0.05]	< 0.01	0.0005	0.0234	69%
Omitting Summit 2008	0.04	[0.03; 0.05]	< 0.01	0.0006	0.0235	69%
Omitting Sunawang 2009	0.04	[0.03; 0.05]	< 0.01	0.0005	0.0227	68%
Omitting Tofail 2008	0.04	[0.03; 0.05]	< 0.01	0.0005	0.0232	69%
Omitting West 2014	0.03	[0.03; 0.04]	< 0.01	0.0006	0.0254	67%
Omitting Zagre 2007	0.03	[0.02; 0.04]	< 0.01	0.0004	0.0200	57%
Omitting Zeng 2008	0.04	[0.03; 0.05]	< 0.01	0.0006	0.0235	69%
**Fixed effect model**	**0.04**	**[0.03; 0.05]**	**< 0.01**	**0.0005**	**0.0228**	**68%**

Mean Difference

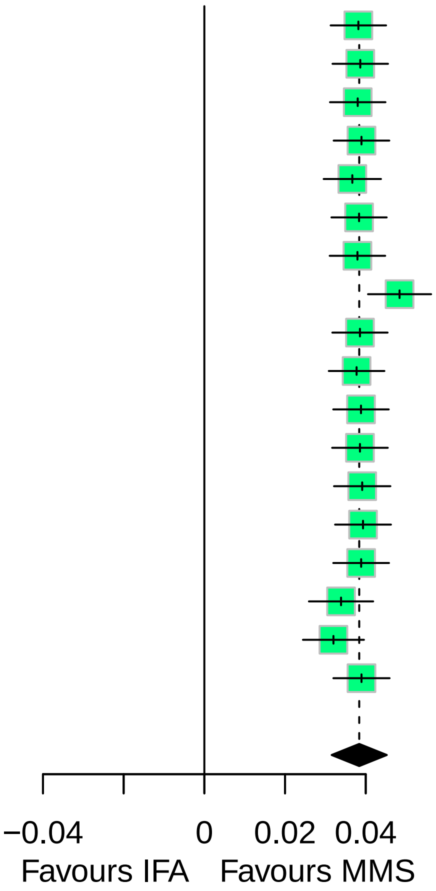

Leave−One−Out Sensitivity Analysis

Weight at Birth, Random

Study	MD	95%−CI	P−value	Tau2	Tau	I2
Omitting Adu−Afarwuah 2015	0.04	[0.02; 0.05]	< 0.01	0.0005	0.0232	69%
Omitting Ashorn 2010	0.04	[0.02; 0.05]	< 0.01	0.0005	0.0232	69%
Omitting Bhutta 2009	0.03	[0.02; 0.05]	< 0.01	0.0005	0.0230	69%
Omitting Christian 2003	0.04	[0.02; 0.05]	< 0.01	0.0005	0.0230	69%
Omitting Fawzi 2007	0.03	[0.02; 0.05]	< 0.01	0.0005	0.0233	68%
Omitting Friis 2004	0.04	[0.02; 0.05]	< 0.01	0.0005	0.0232	70%
Omitting Kaestel 2005	0.03	[0.02; 0.05]	< 0.01	0.0005	0.0229	69%
Omitting Liu 2013	0.04	[0.03; 0.05]	< 0.01	0.0002	0.0140	38%
Omitting Moore 2009	0.04	[0.02; 0.05]	< 0.01	0.0005	0.0230	69%
Omitting Osrin 2005	0.03	[0.02; 0.05]	< 0.01	0.0005	0.0227	68%
Omitting Ramakrishnan 2003	0.04	[0.02; 0.05]	< 0.01	0.0005	0.0229	69%
Omitting Roberfroid 2008	0.04	[0.02; 0.05]	< 0.01	0.0005	0.0234	69%
Omitting Summit 2008	0.04	[0.02; 0.05]	< 0.01	0.0006	0.0235	69%
Omitting Sunawang 2009	0.04	[0.02; 0.05]	< 0.01	0.0005	0.0227	68%
Omitting Tofail 2008	0.04	[0.02; 0.05]	< 0.01	0.0005	0.0232	69%
Omitting West 2014	0.03	[0.02; 0.05]	< 0.01	0.0006	0.0254	67%
Omitting Zagre 2007	0.03	[0.02; 0.05]	< 0.01	0.0004	0.0200	57%
Omitting Zeng 2008	0.04	[0.02; 0.05]	< 0.01	0.0006	0.0235	69%
**Random effects model**	**0.04**	**[0.02; 0.05]**	**< 0.01**	**0.0005**	**0.0228**	**68%**

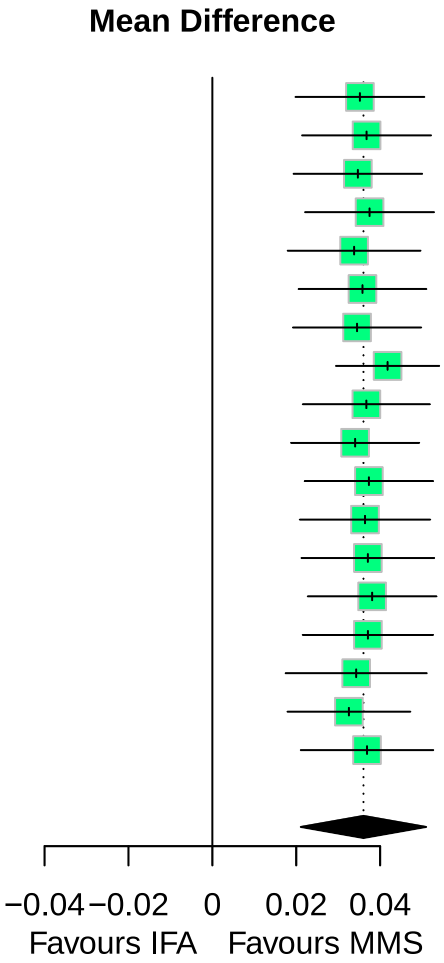

Leave−One−Out Sensitivity Analysis
Weight at 3 Months, Fixed

Study	MD	95%−CI	P−value	Tau2	Tau	I2
Omitting Adu−Afarwuah 2015	0.11	[0.09; 0.13]	< 0.01	0.0133	0.1152	93%
Omitting Fawzi 2007	0.07	[0.05; 0.10]	< 0.01	< 0.0001	0.0041	1%
Omitting Moore 2009	0.11	[0.09; 0.13]	< 0.01	0.0125	0.1117	93%
Omitting Roberfroid 2008	0.11	[0.09; 0.13]	< 0.01	0.0132	0.1147	93%
Omitting Sunawang 2009	0.11	[0.09; 0.13]	< 0.01	0.0127	0.1127	93%
Omitting Tofail 2008	0.12	[0.10; 0.14]	< 0.01	0.0126	0.1124	92%
Omitting West 2014	0.18	[0.15; 0.22]	< 0.01	0.0207	0.1437	89%
**Fixed Effect Model**	**0.11**	**[0.09; 0.13]**	**< 0.01**	**0.0121**	**0.1100**	**91%**

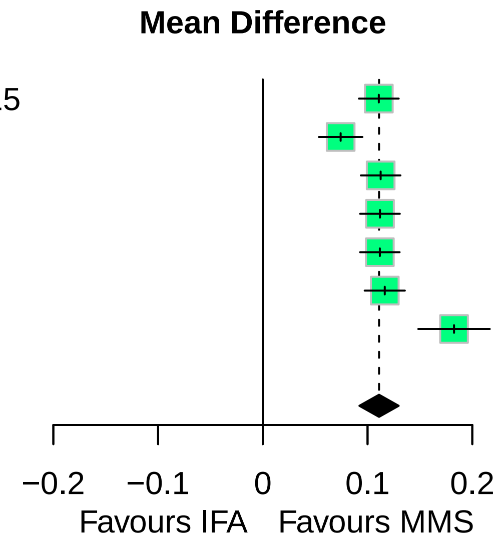

Leave−One−Out Sensitivity Analysis
Weight at 3 Months, Random

Study	MD	95%−CI	P−value	Tau2	Tau	I2
Omitting Adu−Afarwuah 2015	0.09	[−0.02; 0.19]	0.10	0.0133	0.1152	93%
Omitting Fawzi 2007	0.07	[0.05; 0.10]	< 0.01	< 0.0001	0.0041	1%
Omitting Moore 2009	0.10	[0.00; 0.20]	0.04	0.0125	0.1117	93%
Omitting Roberfroid 2008	0.09	[−0.01; 0.19]	0.07	0.0132	0.1147	93%
Omitting Sunawang 2009	0.10	[−0.00; 0.19]	0.06	0.0127	0.1127	93%
Omitting Tofail 2008	0.11	[0.01; 0.21]	0.04	0.0126	0.1124	92%
Omitting West 2014	0.09	[−0.04; 0.21]	0.16	0.0207	0.1437	89%
**Random effects model**	**0.09**	**[−0.00; 0.18]**	**0.05**	**0.0121**	**0.1100**	**91%**

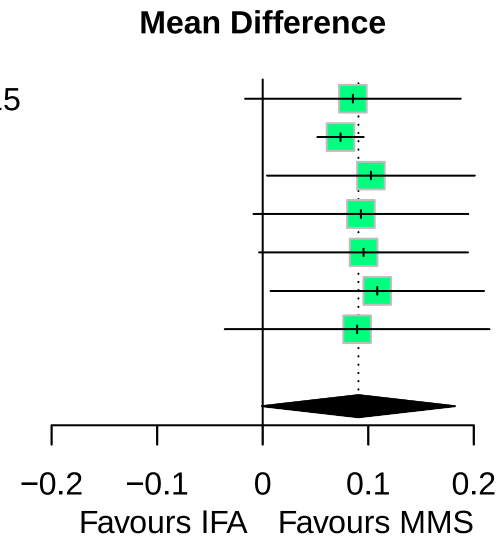

Leave−One−Out Sensitivity Analysis
Weight at 6 Months, Fixed

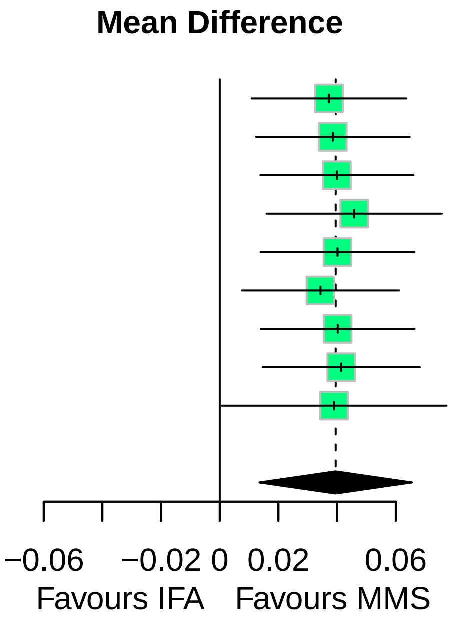

Study	MD	95%−CI	P−value	Tau2	Tau	I2
Omitting Adu−Afarwuah 2015	0.04	[0.01; 0.06]	< 0.01	0	0	0%
Omitting Ashorn 2010 complete FU	0.04	[0.01; 0.06]	< 0.01	0	0	0%
Omitting Ashorn 2010 simplified FU	0.04	[0.01; 0.07]	< 0.01	0	0	0%
Omitting Fawzi 2007	0.05	[0.02; 0.08]	< 0.01	0	0	0%
Omitting Moore 2009	0.04	[0.01; 0.07]	< 0.01	0	0	0%
Omitting Roberfroid 2008	0.03	[0.01; 0.06]	0.01	0	0	0%
Omitting Sunawang 2009	0.04	[0.01; 0.07]	< 0.01	0	0	0%
Omitting Tofail 2008	0.04	[0.01; 0.07]	< 0.01	0	0	0%
Omitting West 2014	0.04	[0.00; 0.08]	0.05	0	0	0%
**Fixed Effect Model**	**0.04**	**[0.01; 0.07]**	**< 0.01**	**0**	**0**	**0%**

Leave−One−Out Sensitivity Analysis
Weight at 6 Months, Random

Study	MD	95%−CI	P−value	Tau2	Tau	I2
Omitting Adu−Afarwuah 2015	0.04	[0.01; 0.06]	< 0.01	0	0	0%
Omitting Ashorn 2010 complete FU	0.04	[0.01; 0.06]	< 0.01	0	0	0%
Omitting Ashorn 2010 simplified FU	0.04	[0.01; 0.07]	< 0.01	0	0	0%
Omitting Fawzi 2007	0.05	[0.02; 0.08]	< 0.01	0	0	0%
Omitting Moore 2009	0.04	[0.01; 0.07]	< 0.01	0	0	0%
Omitting Roberfroid 2008	0.03	[0.01; 0.06]	0.01	0	0	0%
Omitting Sunawang 2009	0.04	[0.01; 0.07]	< 0.01	0	0	0%
Omitting Tofail 2008	0.04	[0.01; 0.07]	< 0.01	0	0	0%
Omitting West 2014	0.04	[0.00; 0.08]	0.05	0	0	0%
**Random effects model**	**0.04**	**[0.01; 0.07]**	**< 0.01**	**0**	**0**	**0%**

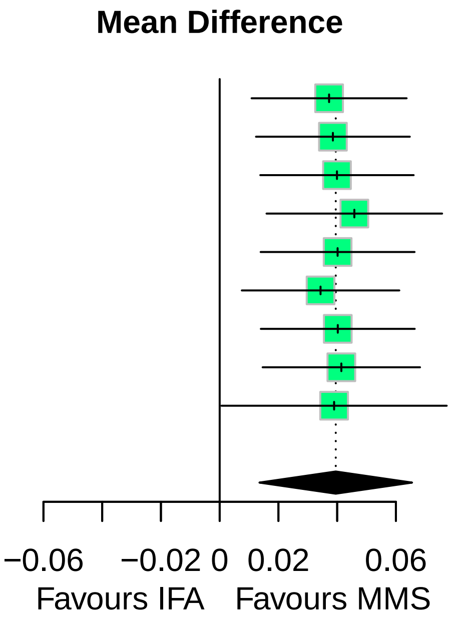

Leave−One−Out Sensitivity Analysis
Weight at 12 Months, Fixed

Study	Mean Difference	MD	95%−CI	P−value	Tau2	Tau	I2
Omitting Adu−Afarwuah 2015		0.02	[−0.01; 0.05]	0.26	0	0	0%
Omitting Ashorn 2010 complete FU		0.03	[−0.01; 0.06]	0.10	0	0	0%
Omitting Fawzi 2007		0.02	[−0.01; 0.06]	0.16	0.0004	0.0211	10%
Omitting Roberfroid 2008		0.02	[−0.01; 0.06]	0.13	0.0003	0.0160	8%
Omitting Sunawang 2009		0.03	[−0.01; 0.06]	0.11	0	0	0%
Omitting Tofail 2008		0.02	[−0.01; 0.05]	0.22	0	0	0%
Omitting West 2014		0.03	[−0.02; 0.09]	0.27	0.0005	0.0220	8%
**Fixed Effect Model**		**0.02**	**[−0.01; 0.05]**	**0.14**	**0**	**0**	**0%**

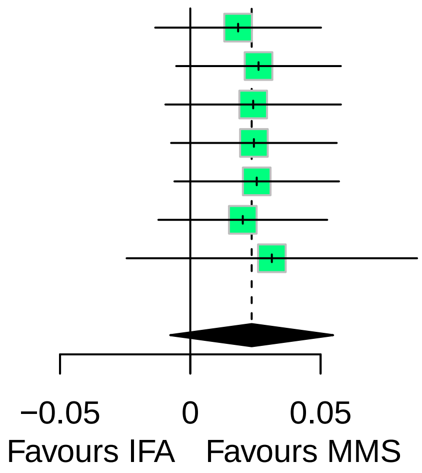

Leave−One−Out Sensitivity Analysis

Weight at 12 Months, Random

Study	MD	95%−CI	P−value	Tau2	Tau	I2
Omitting Adu−Afarwuah 2015	0.02	[−0.01; 0.05]	0.26	0	0	0%
Omitting Ashorn 2010 complete FU	0.03	[−0.01; 0.06]	0.10	0	0	0%
Omitting Fawzi 2007	0.03	[−0.02; 0.07]	0.25	0.0004	0.0211	10%
Omitting Roberfroid 2008	0.03	[−0.01; 0.06]	0.18	0.0003	0.0160	8%
Omitting Sunawang 2009	0.03	[−0.01; 0.06]	0.11	0	0	0%
Omitting Tofail 2008	0.02	[−0.01; 0.05]	0.22	0	0	0%
Omitting West 2014	0.03	[−0.03; 0.09]	0.31	0.0005	0.0220	8%
**Random effects model**	**0.02**	**[−0.01; 0.05]**	**0.14**	**0**	**0**	**0%**

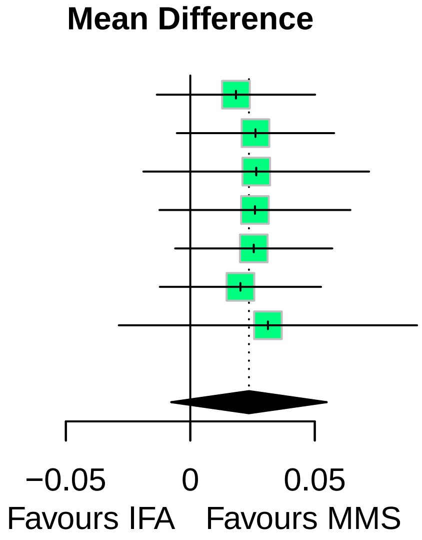

Leave−One−Out Sensitivity Analysis
Weight at 18 Months, Fixed

Study	Mean Difference	MD	95%−CI	P−value	Tau2	Tau	I2
Omitting Adu−Afarwuah 2015		0.06	[−0.05; 0.16]	0.27	0	0	0%
Omitting Ashorn 2010 complete FU		0.12	[0.02; 0.22]	0.02	0	0	0%
Omitting Ashorn 2010 simplified FU		0.10	[0.00; 0.19]	0.05	0.0019	0.0440	14%
Omitting Fawzi 2007		0.09	[−0.00; 0.18]	0.06	0.0029	0.0540	23%
Omitting Tofail 2008		0.06	[−0.07; 0.18]	0.36	0.0028	0.0526	13%
**Fixed Effect Model**		**0.09**	**[−0.00; 0.18]**	**0.06**	**0**	**0**	**0%**

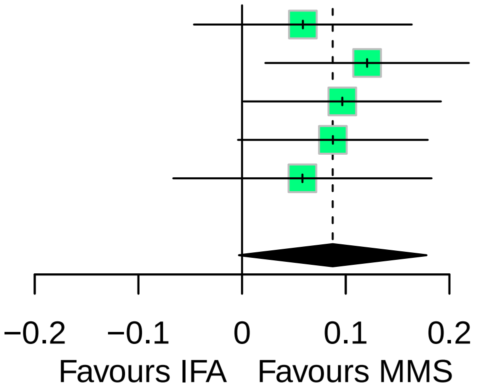

Leave−One−Out Sensitivity Analysis
Weight at 18 Months, Random

Study	MD	95%−CI	P−value	Tau2	Tau	I2
Omitting Adu−Afarwuah 2015	0.06	[−0.05; 0.16]	0.27	0	0	0%
Omitting Ashorn 2010 complete FU	0.12	[0.02; 0.22]	0.02	0	0	0%
Omitting Ashorn 2010 simplified FU	0.09	[−0.02; 0.20]	0.10	0.0019	0.0440	14%
Omitting Fawzi 2007	0.08	[−0.03; 0.19]	0.16	0.0029	0.0540	23%
Omitting Tofail 2008	0.05	[−0.09; 0.19]	0.46	0.0028	0.0526	13%
**Random effects model**	**0.09**	**[−0.00; 0.18]**	**0.06**	**0**	**0**	**0%**

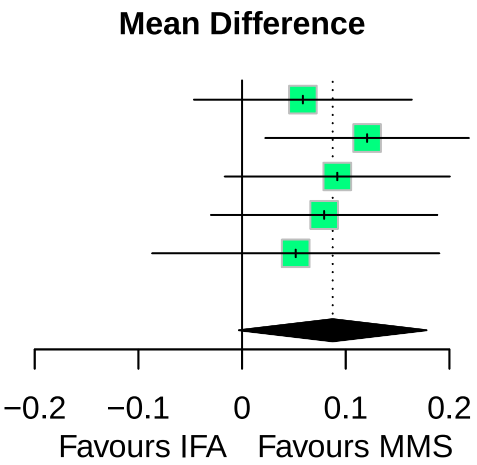

Leave−One−Out Sensitivity Analysis

Weight at 24 Months, Fixed

Study	MD	95%−CI	P−value	Tau2	Tau	I2
Omitting Ashorn 2010 complete FU	0.01	[−0.04; 0.06]	0.69	0	0	0%
Omitting Roberfroid 2008	0.01	[−0.04; 0.05]	0.80	0	0	0%
Omitting Tofail 2008	0.01	[−0.05; 0.06]	0.83	0	0	0%
Omitting West 2014	−0.01	[−0.13; 0.11]	0.83	0	0	0%
**Fixed Effect Model**	**0.01**	**[−0.04; 0.05]**	**0.80**	**0**	**0**	**0%**

Mean Difference

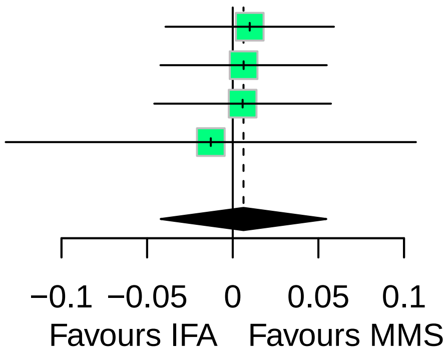

Leave−One−Out Sensitivity Analysis
Weight at 24 Months, Random

Study	MD	95%−CI	P−value	Tau2	Tau	I2
Omitting Ashorn 2010 complete FU	0.01	[−0.04; 0.06]	0.69	0	0	0%
Omitting Roberfroid 2008	0.01	[−0.04; 0.05]	0.80	0	0	0%
Omitting Tofail 2008	0.01	[−0.05; 0.06]	0.83	0	0	0%
Omitting West 2014	−0.01	[−0.13; 0.11]	0.83	0	0	0%
**Random effects model**	**0.01**	**[−0.04; 0.05]**	**0.80**	**0**	**0**	**0%**

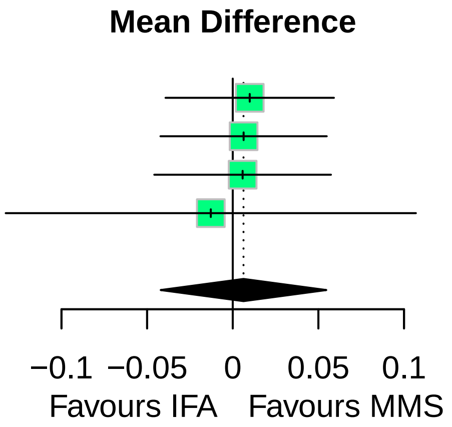

Leave−One−Out Sensitivity Analysis

MUAC at Birth, Fixed

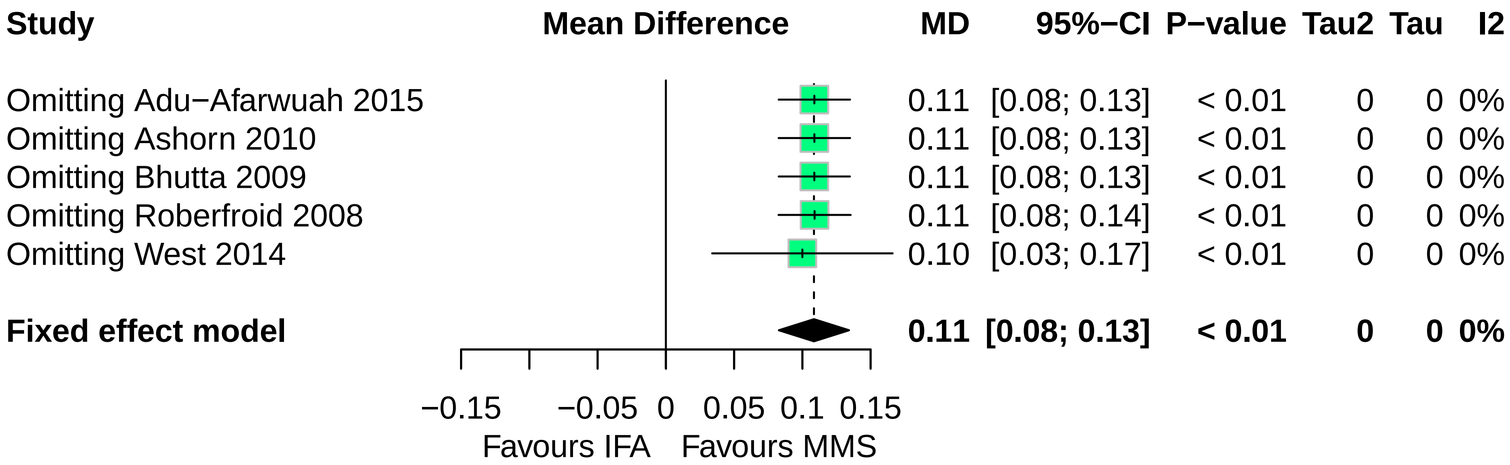

Leave−One−Out Sensitivity Analysis

MUAC at Birth, Random

Study	MD	95%−CI	P−value	Tau2	Tau	I2
Omitting Adu−Afarwuah 2015	0.11	[0.08; 0.13]	< 0.01	0	0	0%
Omitting Ashorn 2010	0.11	[0.08; 0.13]	< 0.01	0	0	0%
Omitting Bhutta 2009	0.11	[0.08; 0.13]	< 0.01	0	0	0%
Omitting Roberfroid 2008	0.11	[0.08; 0.14]	< 0.01	0	0	0%
Omitting West 2014	0.10	[0.03; 0.17]	< 0.01	0	0	0%
**Random effects model**	**0.11**	**[0.08; 0.13]**	**< 0.01**	**0**	**0**	**0%**

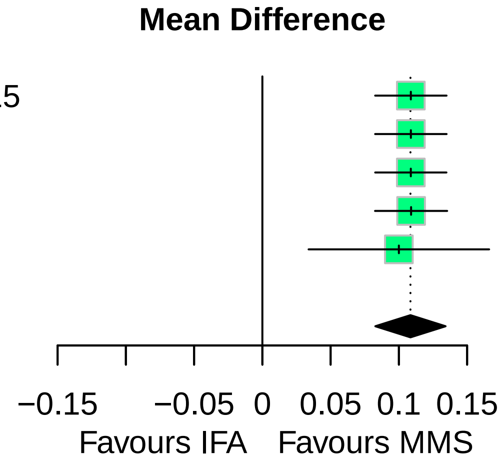

Leave−One−Out Sensitivity Analysis
MUAC at 3 Months, Fixed

Study	MD	95%−CI	P−value	Tau2	Tau	I2
Omitting Adu−Afarwuah 2015	0.08	[0.05; 0.11]	< 0.01	0.0026	0.0505	49%
Omitting Fawzi 2007	0.09	[0.07; 0.12]	< 0.01	0	0	0%
Omitting Moore 2009	0.08	[0.06; 0.11]	< 0.01	0.0021	0.0461	46%
Omitting Roberfroid 2008	0.08	[0.06; 0.11]	< 0.01	0.0019	0.0436	42%
Omitting Sunawang 2009	0.08	[0.06; 0.11]	< 0.01	0.0023	0.0477	49%
Omitting West 2014	0.01	[−0.05; 0.07]	0.73	0	0	0%
**Fixed Effect Model**	**0.08**	**[0.06; 0.11]**	**< 0.01**	**0.0017**	**0.0407**	**37%**

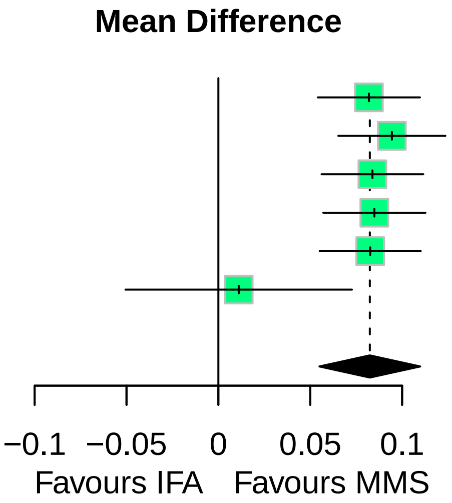

Leave−One−Out Sensitivity Analysis
MUAC at 3 Months, Random

Study	MD	95%−CI	P−value	Tau2	Tau	I2
Omitting Adu−Afarwuah 2015	0.04	[−0.02; 0.11]	0.21	0.0026	0.0505	49%
Omitting Fawzi 2007	0.09	[0.07; 0.12]	< 0.01	0	0	0%
Omitting Moore 2009	0.06	[−0.01; 0.12]	0.09	0.0021	0.0461	46%
Omitting Roberfroid 2008	0.06	[−0.00; 0.12]	0.06	0.0019	0.0436	42%
Omitting Sunawang 2009	0.05	[−0.01; 0.12]	0.11	0.0023	0.0477	49%
Omitting West 2014	0.01	[−0.05; 0.07]	0.73	0	0	0%
**Random effects model**	**0.05**	**[−0.00; 0.11]**	**0.06**	**0.0017**	**0.0407**	**37%**

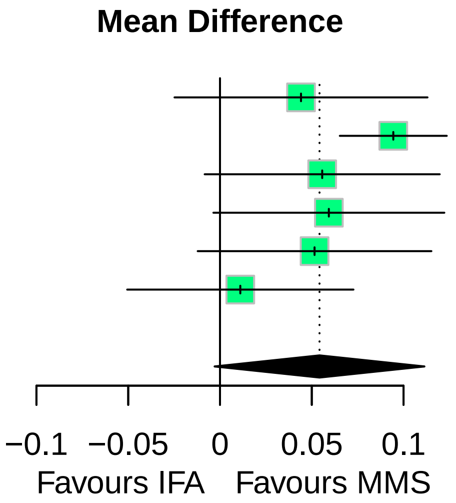

Leave−One−Out Sensitivity Analysis
MUAC at 6 Months, Fixed

Study	MD	95%−CI	P−value	Tau2	Tau	I2
Omitting Adu−Afarwuah 2015	0.02	[−0.02; 0.05]	0.36	0	0	0%
Omitting Ashorn 2010 complete FU	0.02	[−0.02; 0.05]	0.29	0	0	0%
Omitting Ashorn 2010 simplified FU	0.02	[−0.01; 0.06]	0.22	0	0	0%
Omitting Fawzi 2007	0.03	[−0.01; 0.06]	0.17	0	0	0%
Omitting Moore 2009	0.02	[−0.02; 0.05]	0.29	0	0	0%
Omitting Roberfroid 2008	0.02	[−0.02; 0.05]	0.30	0	0	0%
Omitting Sunawang 2009	0.02	[−0.01; 0.05]	0.25	0	0	0%
Omitting West 2014	0.02	[−0.04; 0.08]	0.53	0	0	0%
**Fixed Effect Model**	**0.02**	**[−0.01; 0.05]**	**0.25**	**0**	**0**	**0%**

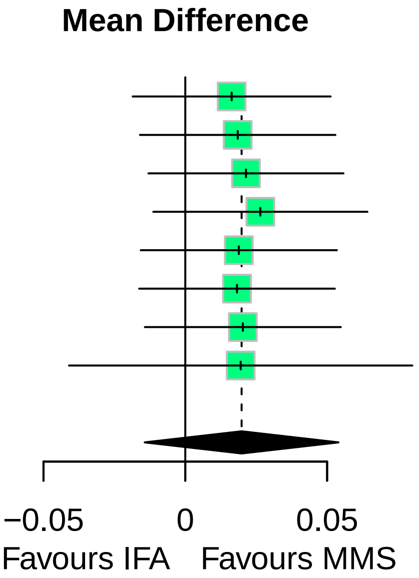

Leave−One−Out Sensitivity Analysis
MUAC at 6 Months, Random

Study	MD	95%−CI	P−value	Tau2	Tau	I2
Omitting Adu−Afarwuah 2015	0.02	[−0.02; 0.05]	0.36	0	0	0%
Omitting Ashorn 2010 complete FU	0.02	[−0.02; 0.05]	0.29	0	0	0%
Omitting Ashorn 2010 simplified FU	0.02	[−0.01; 0.06]	0.22	0	0	0%
Omitting Fawzi 2007	0.03	[−0.01; 0.06]	0.17	0	0	0%
Omitting Moore 2009	0.02	[−0.02; 0.05]	0.29	0	0	0%
Omitting Roberfroid 2008	0.02	[−0.02; 0.05]	0.30	0	0	0%
Omitting Sunawang 2009	0.02	[−0.01; 0.05]	0.25	0	0	0%
Omitting West 2014	0.02	[−0.04; 0.08]	0.53	0	0	0%
**Random effects model**	**0.02**	**[−0.01; 0.05]**	**0.25**	**0**	**0**	**0%**

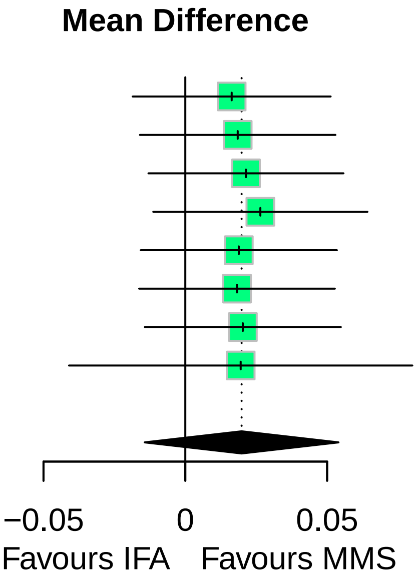

Leave−One−Out Sensitivity Analysis

MUAC at 12 Months, Fixed

Study	MD	95%−CI	P−value	Tau2	Tau	I2
Omitting Adu−Afarwuah 2015	0.02	[−0.02; 0.05]	0.29	0	0	0%
Omitting Ashorn 2010 complete FU	0.03	[−0.01; 0.06]	0.15	0	0	0%
Omitting Fawzi 2007	0.02	[−0.01; 0.06]	0.22	0	0	0%
Omitting Roberfroid 2008	0.02	[−0.01; 0.05]	0.25	0	0	0%
Omitting Sunawang 2009	0.02	[−0.01; 0.06]	0.19	0	0	0%
Omitting West 2014	0.03	[−0.04; 0.10]	0.40	0	0	0%
**Fixed Effect Model**	**0.02**	**[−0.01; 0.06]**	**0.20**	**0**	**0**	**0%**

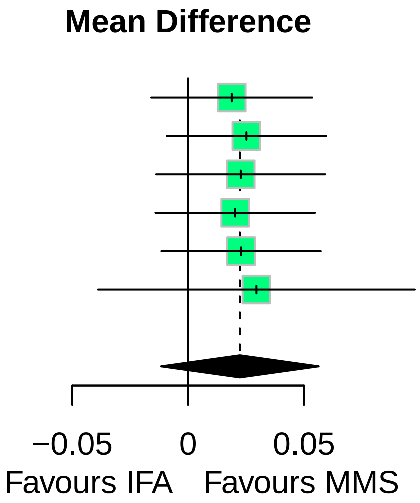

Leave−One−Out Sensitivity Analysis
MUAC at 12 Months, Random

Study	MD	95%−CI	P−value	Tau2	Tau	I2
Omitting Adu−Afarwuah 2015	0.02	[−0.02; 0.05]	0.29	0	0	0%
Omitting Ashorn 2010 complete FU	0.03	[−0.01; 0.06]	0.15	0	0	0%
Omitting Fawzi 2007	0.02	[−0.01; 0.06]	0.22	0	0	0%
Omitting Roberfroid 2008	0.02	[−0.01; 0.05]	0.25	0	0	0%
Omitting Sunawang 2009	0.02	[−0.01; 0.06]	0.19	0	0	0%
Omitting West 2014	0.03	[−0.04; 0.10]	0.40	0	0	0%
**Random effects model**	**0.02**	**[−0.01; 0.06]**	**0.20**	**0**	**0**	**0%**

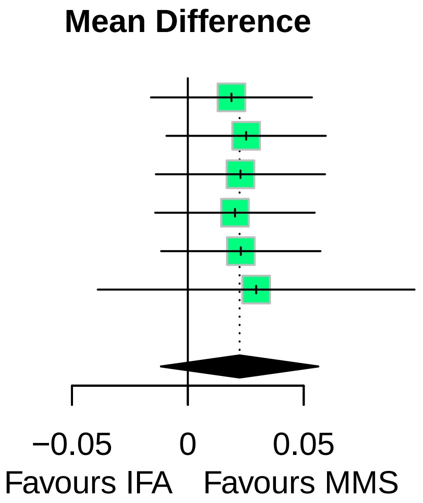

Leave−One−Out Sensitivity Analysis
MUAC at 18 Months, Fixed

Study	MD	95%−CI	P−value	Tau2	Tau	I2
Omitting Adu−Afarwuah 2015	0.01	[−0.15; 0.18]	0.87	0	0	0%
Omitting Ashorn 2010 complete FU	0.08	[−0.05; 0.21]	0.23	0	0	0%
Omitting Ashorn 2010 simplified FU	0.05	[−0.07; 0.17]	0.42	0	0	0%
Omitting Fawzi 2007	0.07	[−0.04; 0.19]	0.22	0	0	0%
**Fixed Effect Model**	**0.06**	**[−0.05; 0.17]**	**0.31**	**0**	**0**	**0%**

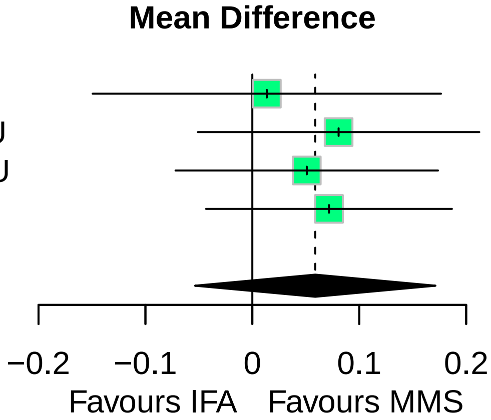

Leave−One−Out Sensitivity Analysis
MUAC at 18 Months, Random

Study	Mean Difference	MD	95%−CI	P−value	Tau2	Tau	I2
Omitting Adu−Afarwuah 2015		0.01	[−0.15; 0.18]	0.87	0	0	0%
Omitting Ashorn 2010 complete FU		0.08	[−0.05; 0.21]	0.23	0	0	0%
Omitting Ashorn 2010 simplified FU		0.05	[−0.07; 0.17]	0.42	0	0	0%
Omitting Fawzi 2007		0.07	[−0.04; 0.19]	0.22	0	0	0%
**Random effects model**		**0.06**	**[−0.05; 0.17]**	**0.31**	**0**	**0**	**0%**

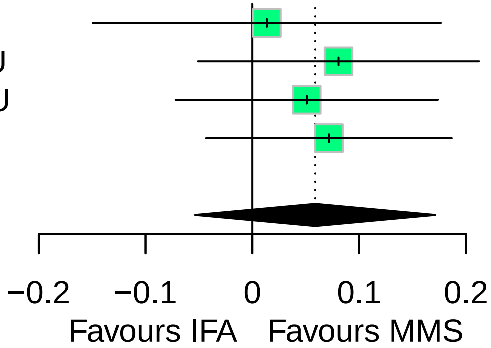

Leave−One−Out Sensitivity Analysis

MUAC at 24 Months, Fixed

Study	MD	95%−CI	P−value	Tau2	Tau	I2
Omitting Ashorn 2010 complete FU	−0.00	[−0.05; 0.04]	0.89	0	0	0%
Omitting Bliznashka 2021	−0.01	[−0.05; 0.04]	0.75	0.0042	0.0648	27%
Omitting Roberfroid 2008	−0.01	[−0.05; 0.03]	0.66	0.0076	0.0874	44%
Omitting West 2014	−0.16	[−0.33; 0.02]	0.07	0	0	0%
**Fixed Effect Model**	**−0.01**	**[−0.05; 0.03]**	**0.66**	**0.0024**	**0.0491**	**16%**

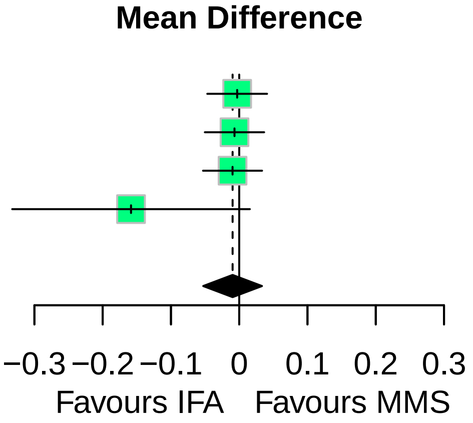

Leave−One−Out Sensitivity Analysis
MUAC at 24 Months, Random

Study	MD	95%−CI	P−value	Tau2	Tau	I2
Omitting Ashorn 2010 complete FU	−0.00	[−0.05; 0.04]	0.89	0	0	0%
Omitting Bliznashka 2021	−0.04	[−0.15; 0.08]	0.52	0.0042	0.0648	27%
Omitting Roberfroid 2008	−0.07	[−0.21; 0.07]	0.33	0.0076	0.0874	44%
Omitting West 2014	−0.16	[−0.33; 0.02]	0.07	0	0	0%
**Random effects model**	**−0.04**	**[−0.13; 0.05]**	**0.41**	**0.0024**	**0.0491**	**16%**

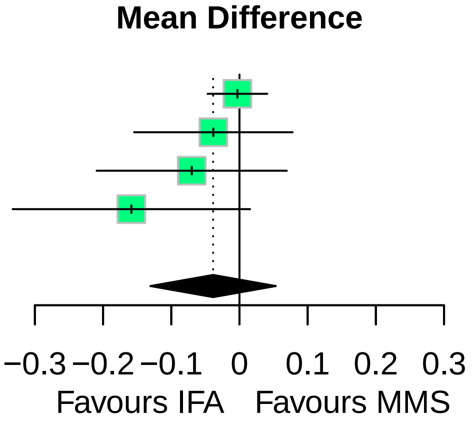

Leave−One−Out Sensitivity Analysis

LAZ at Birth, Fixed

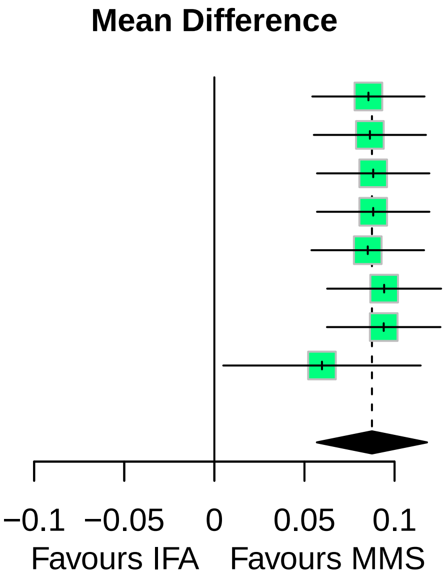

Study	MD	95%−CI	P−value	Tau2	Tau	I2
Omitting Adu−Afarwuah 2015	0.09	[0.05; 0.12]	< 0.01	0.0002	0.0147	6%
Omitting Ashorn 2010	0.09	[0.06; 0.12]	< 0.01	0.0004	0.0189	10%
Omitting Fawzi 2007	0.09	[0.06; 0.12]	< 0.01	0.0004	0.0204	12%
Omitting Osrin 2005	0.09	[0.06; 0.12]	< 0.01	0.0004	0.0204	12%
Omitting Roberfroid 2008	0.09	[0.05; 0.12]	< 0.01	0.0002	0.0134	5%
Omitting Sunawang 2009	0.09	[0.06; 0.13]	< 0.01	0	0	0%
Omitting Tofail 2008	0.09	[0.06; 0.13]	< 0.01	0	0	0%
Omitting West 2014	0.06	[0.00; 0.11]	0.03	0	0	0%
**Fixed effect model**	**0.09**	**[0.06; 0.12]**	**< 0.01**	**0**	**0**	**0%**

Leave−One−Out Sensitivity Analysis

LAZ at Birth, Random

Study	MD	95%−CI	P−value	Tau2	Tau	I2
Omitting Adu−Afarwuah 2015	0.08	[0.04; 0.12]	< 0.01	0.0002	0.0147	6%
Omitting Ashorn 2010	0.08	[0.04; 0.12]	< 0.01	0.0004	0.0189	10%
Omitting Fawzi 2007	0.08	[0.04; 0.12]	< 0.01	0.0004	0.0204	12%
Omitting Osrin 2005	0.08	[0.04; 0.12]	< 0.01	0.0004	0.0204	12%
Omitting Roberfroid 2008	0.08	[0.04; 0.12]	< 0.01	0.0002	0.0134	5%
Omitting Sunawang 2009	0.09	[0.06; 0.13]	< 0.01	0	0	0%
Omitting Tofail 2008	0.09	[0.06; 0.13]	< 0.01	0	0	0%
Omitting West 2014	0.06	[0.00; 0.11]	0.03	0	0	0%
**Random effects model**	**0.09**	**[0.06; 0.12]**	**< 0.01**	**0**	**0**	**0%**

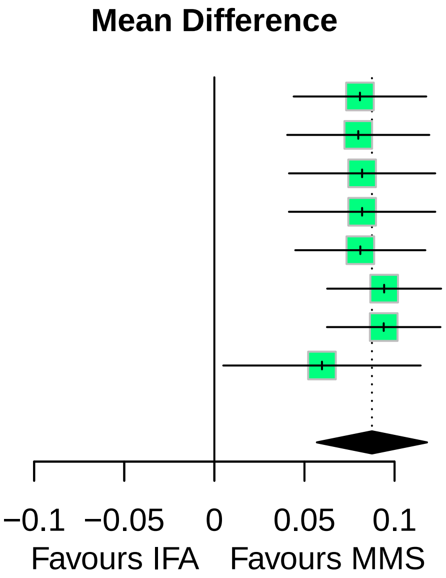

Leave−One−Out Sensitivity Analysis
LAZ at 3 Months, Fixed

Study	MD	95%−CI	P−value	Tau2	Tau	I2
Omitting Adu−Afarwuah 2015	0.09	[0.06; 0.12]	< 0.01	0.0015	0.0385	36%
Omitting Fawzi 2007	0.10	[0.07; 0.13]	< 0.01	0.0023	0.0478	34%
Omitting Moore 2009	0.09	[0.06; 0.12]	< 0.01	0.0017	0.0411	41%
Omitting Roberfroid 2008	0.09	[0.06; 0.12]	< 0.01	0.0018	0.0430	43%
Omitting Sunawang 2009	0.09	[0.06; 0.12]	< 0.01	0.0017	0.0409	41%
Omitting Tofail 2008	0.10	[0.07; 0.13]	< 0.01	0	0	0%
Omitting West 2014	0.04	[−0.01; 0.10]	0.10	0	0	0%
**Fixed Effect Model**	**0.09**	**[0.06; 0.12]**	**< 0.01**	**0.0014**	**0.0371**	**33%**

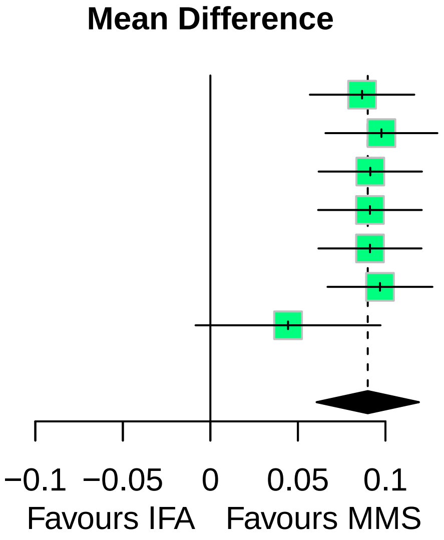

Leave−One−Out Sensitivity Analysis
LAZ at 3 Months, Random

Study	Mean Difference	MD	95%−CI	P−value	Tau2	Tau	I2
Omitting Adu−Afarwuah 2015		0.06	[0.00; 0.11]	0.04	0.0015	0.0385	36%
Omitting Fawzi 2007		0.07	[0.00; 0.14]	0.04	0.0023	0.0478	34%
Omitting Moore 2009		0.07	[0.02; 0.13]	0.01	0.0017	0.0411	41%
Omitting Roberfroid 2008		0.07	[0.01; 0.13]	0.02	0.0018	0.0430	43%
Omitting Sunawang 2009		0.07	[0.02; 0.13]	0.01	0.0017	0.0409	41%
Omitting Tofail 2008		0.10	[0.07; 0.13]	< 0.01	0	0	0%
Omitting West 2014		0.04	[−0.01; 0.10]	0.10	0	0	0%
**Random effects model**		**0.07**	**[0.02; 0.12]**	**< 0.01**	**0.0014**	**0.0371**	**33%**

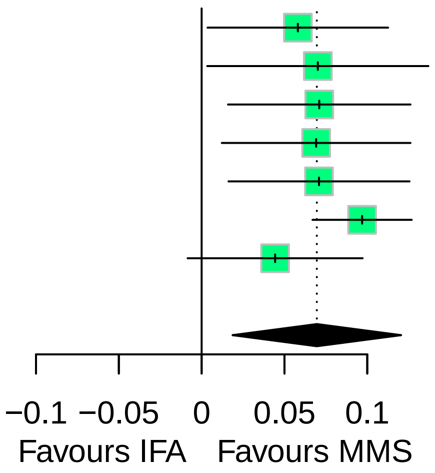

Leave−One−Out Sensitivity Analysis
LAZ at 6 Months, Fixed

Study	Mean Difference	MD	95%−CI	P−value	Tau2	Tau	I2
Omitting Adu−Afarwuah 2015		0.04	[0.01; 0.07]	0.02	0	0	0%
Omitting Ashorn 2010 complete FU		0.04	[0.01; 0.07]	0.02	0	0	0%
Omitting Ashorn 2010 simplified FU		0.04	[0.01; 0.07]	< 0.01	0	0	0%
Omitting Fawzi 2007		0.05	[0.01; 0.08]	0.01	0	0	0%
Omitting Moore 2009		0.04	[0.01; 0.07]	0.01	0	0	0%
Omitting Roberfroid 2008		0.04	[0.01; 0.07]	0.02	0	0	0%
Omitting Sunawang 2009		0.04	[0.01; 0.07]	< 0.01	0	0	0%
Omitting Tofail 2008		0.05	[0.01; 0.08]	< 0.01	0	0	0%
Omitting West 2014		0.02	[−0.03; 0.07]	0.42	0	0	0%
**Fixed Effect Model**		**0.04**	**[0.01; 0.07]**	**0.01**	**0**	**0**	**0%**

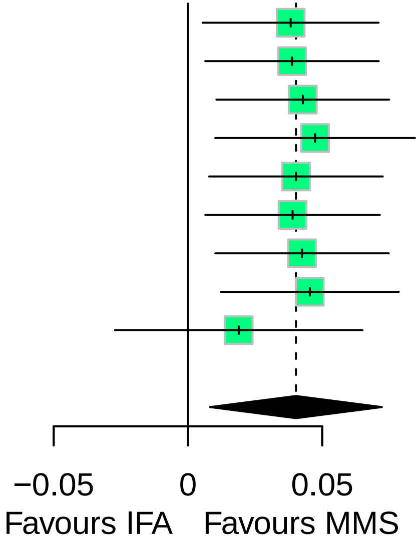

Leave−One−Out Sensitivity Analysis
LAZ at 6 Months, Random

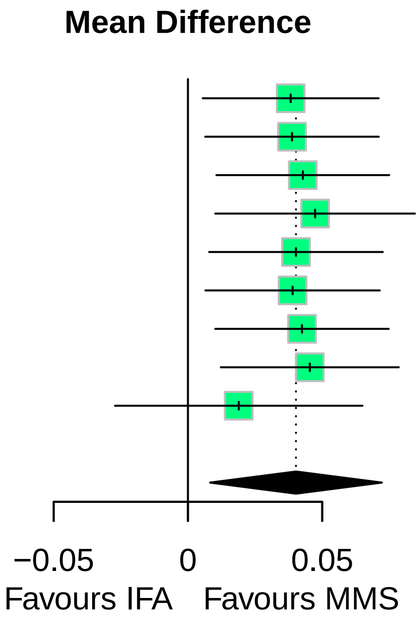

Study	MD	95%−CI	P−value	Tau2	Tau	I2
Omitting Adu−Afarwuah 2015	0.04	[0.01; 0.07]	0.02	0	0	0%
Omitting Ashorn 2010 complete FU	0.04	[0.01; 0.07]	0.02	0	0	0%
Omitting Ashorn 2010 simplified FU	0.04	[0.01; 0.07]	< 0.01	0	0	0%
Omitting Fawzi 2007	0.05	[0.01; 0.08]	0.01	0	0	0%
Omitting Moore 2009	0.04	[0.01; 0.07]	0.01	0	0	0%
Omitting Roberfroid 2008	0.04	[0.01; 0.07]	0.02	0	0	0%
Omitting Sunawang 2009	0.04	[0.01; 0.07]	< 0.01	0	0	0%
Omitting Tofail 2008	0.05	[0.01; 0.08]	< 0.01	0	0	0%
Omitting West 2014	0.02	[−0.03; 0.07]	0.42	0	0	0%
**Random effects model**	**0.04**	**[0.01; 0.07]**	**0.01**	**0**	**0**	**0%**

Leave−One−Out Sensitivity Analysis
LAZ at 12 Months, Fixed

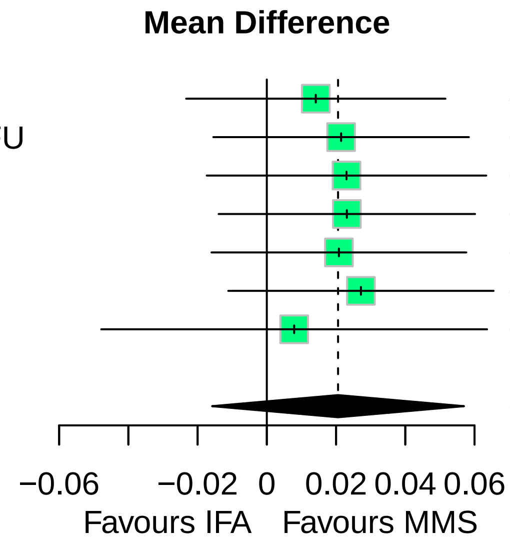

Study	MD	95%−CI	P−value	Tau2	Tau	I2
Omitting Adu−Afarwuah 2015	0.01	[−0.02; 0.05]	0.46	0	0	0%
Omitting Ashorn 2010 complete FU	0.02	[−0.02; 0.06]	0.25	0	0	0%
Omitting Fawzi 2007	0.02	[−0.02; 0.06]	0.26	0	0	0%
Omitting Roberfroid 2008	0.02	[−0.01; 0.06]	0.22	0	0	0%
Omitting Sunawang 2009	0.02	[−0.02; 0.06]	0.27	0	0	0%
Omitting Tofail 2008	0.03	[−0.01; 0.07]	0.16	0	0	0%
Omitting West 2014	0.01	[−0.05; 0.06]	0.78	0	0	0%
**Fixed Effect Model**	**0.02**	**[−0.02; 0.06]**	**0.27**	**0**	**0**	**0%**

Leave−One−Out Sensitivity Analysis

LAZ at 12 Months, Random

Study	MD	95%−CI	P−value	Tau2	Tau	I2
Omitting Adu−Afarwuah 2015	0.01	[−0.02; 0.05]	0.46	0	0	0%
Omitting Ashorn 2010 complete FU	0.02	[−0.02; 0.06]	0.25	0	0	0%
Omitting Fawzi 2007	0.02	[−0.02; 0.06]	0.26	0	0	0%
Omitting Roberfroid 2008	0.02	[−0.01; 0.06]	0.22	0	0	0%
Omitting Sunawang 2009	0.02	[−0.02; 0.06]	0.27	0	0	0%
Omitting Tofail 2008	0.03	[−0.01; 0.07]	0.16	0	0	0%
Omitting West 2014	0.01	[−0.05; 0.06]	0.78	0	0	0%
**Random effects model**	**0.02**	**[−0.02; 0.06]**	**0.27**	**0**	**0**	**0%**

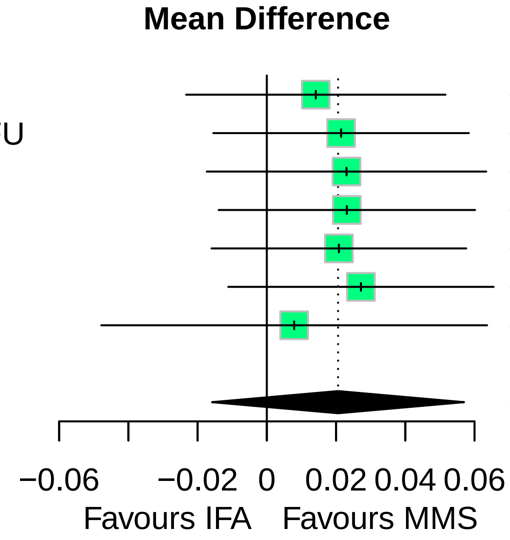

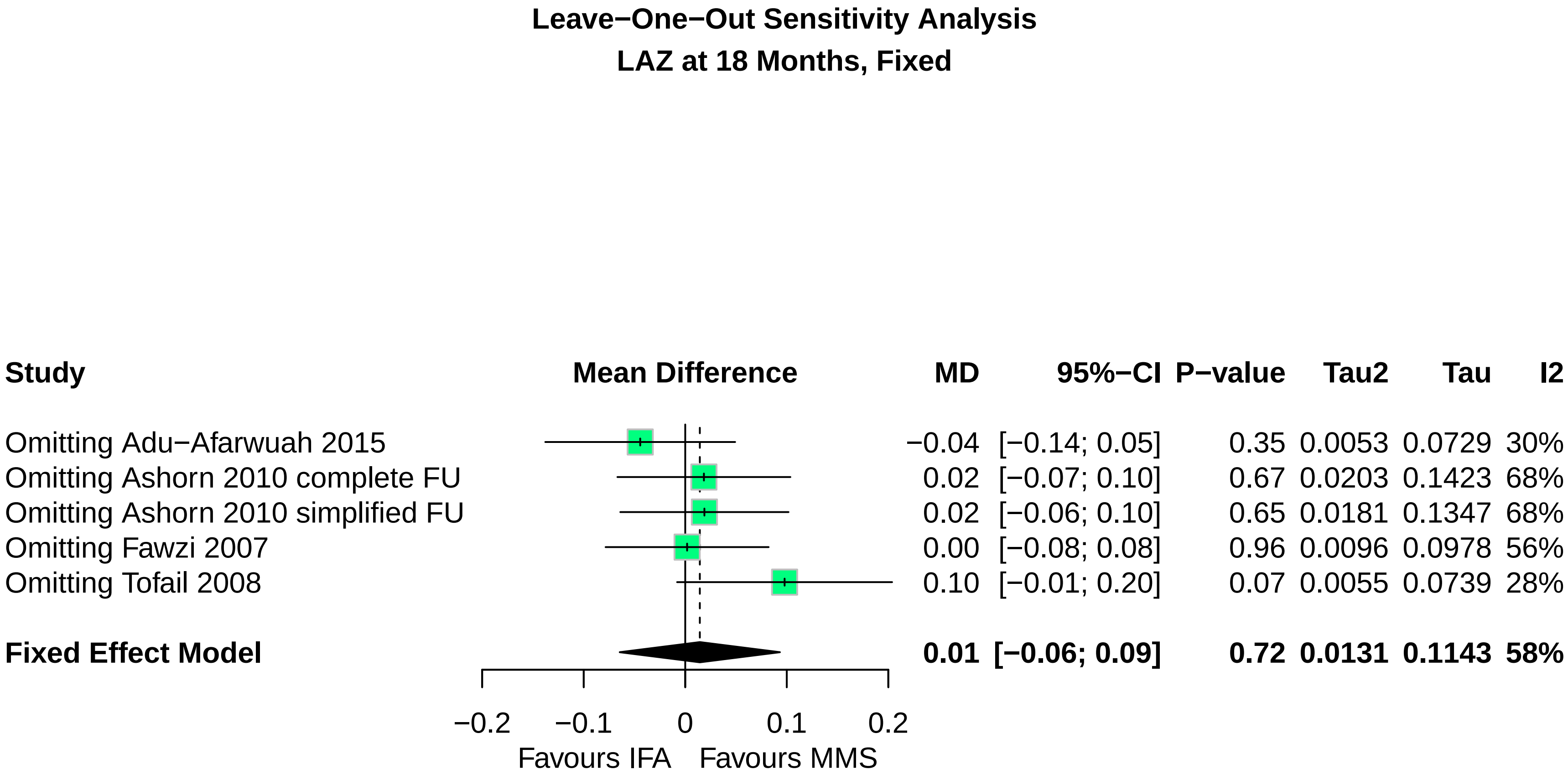
Leave−One−Out Sensitivity Analysis
LAZ at 18 Months, Fixed
Study
Mean Difference
MD
95%−CI
P−value
Tau2
Tau
I2
Omitting Adu−Afarwuah 2015 −0.04 [−0.14; 0.05] 0.35 0.0053 0.0729 30%
Omitting Ashorn 2010 complete FU 0.02 [−0.07; 0.10] 0.67 0.0203 0.1423 68%
Omitting Ashorn 2010 simplified FU 0.02 [−0.06; 0.10] 0.65 0.0181 0.1347 68%
Omitting Fawzi 2007 0.00 [−0.08; 0.08] 0.96 0.0096 0.0978 56%
Omitting Tofail 2008 0.10 [−0.01; 0.20] 0.07 0.0055 0.0739 28%
Fixed Effect Model 0.01 [−0.06; 0.09] 0.72 0.0131 0.1143 58%
−0.2
−0.1
0
0.1
0.2
Favours IFA
Favours MMS

Leave−One−Out Sensitivity Analysis
LAZ at 18 Months, Random

Study	MD	95%−CI	P−value	Tau2	Tau	I2
Omitting Adu−Afarwuah 2015	−0.02	[−0.15; 0.11]	0.75	0.0053	0.0729	30%
Omitting Ashorn 2010 complete FU	0.06	[−0.12; 0.24]	0.52	0.0203	0.1423	68%
Omitting Ashorn 2010 simplified FU	0.06	[−0.11; 0.23]	0.50	0.0181	0.1347	68%
Omitting Fawzi 2007	0.01	[−0.12; 0.14]	0.89	0.0096	0.0978	56%
Omitting Tofail 2008	0.09	[−0.04; 0.23]	0.18	0.0055	0.0739	28%
**Random effects model**	**0.04**	**[−0.10; 0.18]**	**0.58**	**0.0131**	**0.1143**	**58%**

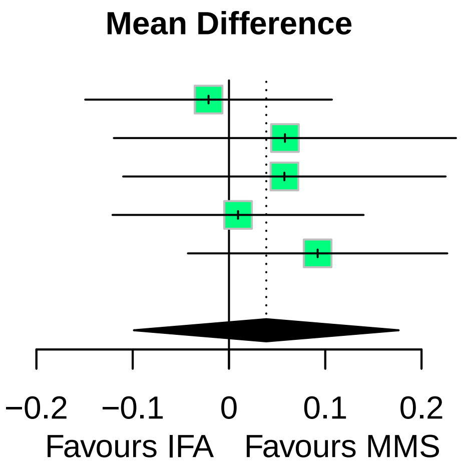

Leave−One−Out Sensitivity Analysis
LAZ at 24 Months, Fixed

Study	MD	95%−CI	P−value	Tau2	Tau	I2
Omitting Ashorn 2010 complete FU	−0.03	[−0.07; 0.02]	0.22	0	0	0%
Omitting Bliznashka 2021	−0.03	[−0.07; 0.02]	0.24	0	0	0%
Omitting Roberfroid 2008	−0.03	[−0.07; 0.02]	0.20	0	0	0%
Omitting Tofail 2008	−0.02	[−0.07; 0.02]	0.37	0	0	0%
Omitting West 2014	−0.06	[−0.15; 0.04]	0.25	0	0	0%
**Fixed Effect Model**	**−0.03**	**[−0.07; 0.02]**	**0.21**	**0**	**0**	**0%**

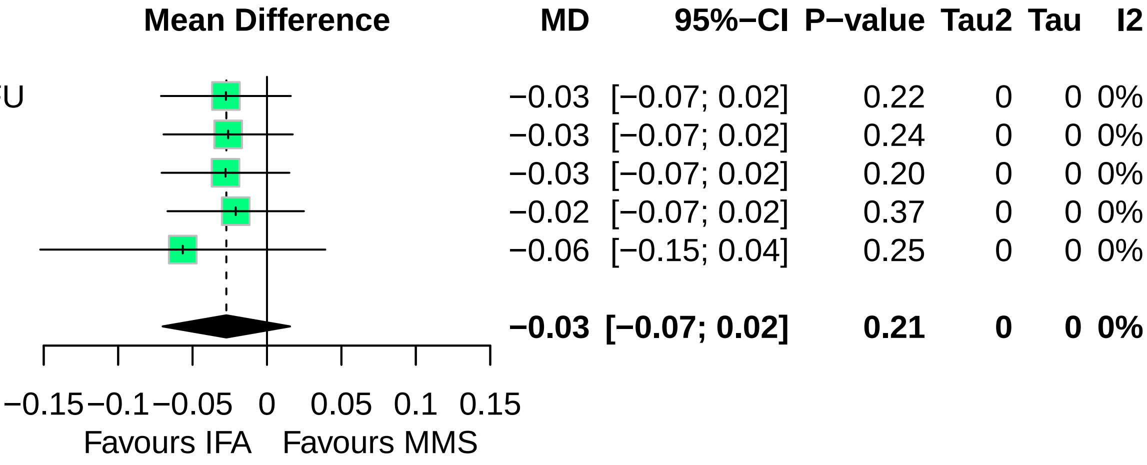

Leave−One−Out Sensitivity Analysis
LAZ at 24 Months, Random

Study	Mean Difference	MD	95%−CI	P−value	Tau2	Tau	I2
Omitting Ashorn 2010 complete FU		−0.03	[−0.07; 0.02]	0.22	0	0	0%
Omitting Bliznashka 2021		−0.03	[−0.07; 0.02]	0.24	0	0	0%
Omitting Roberfroid 2008		−0.03	[−0.07; 0.02]	0.20	0	0	0%
Omitting Tofail 2008		−0.02	[−0.07; 0.02]	0.37	0	0	0%
Omitting West 2014		−0.06	[−0.15; 0.04]	0.25	0	0	0%
**Random effects model**		**−0.03**	**[−0.07; 0.02]**	**0.21**	**0**	**0**	**0%**

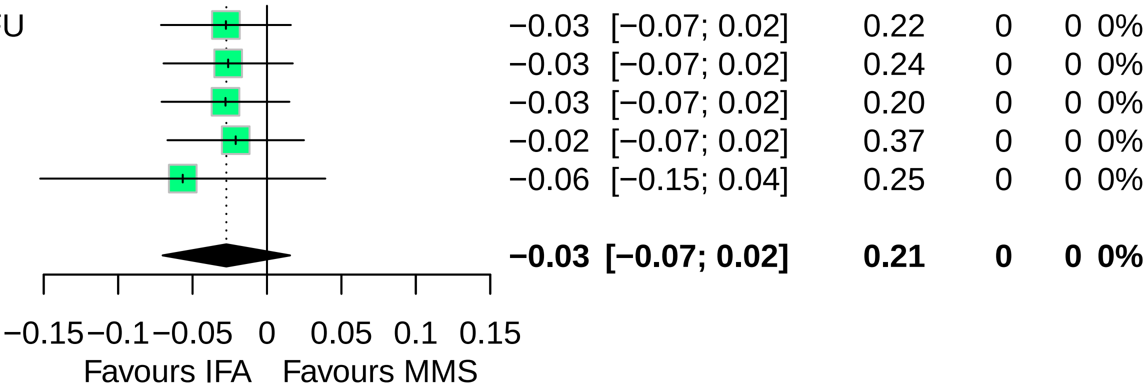

Leave−One−Out Sensitivity Analysis

WAZ at Birth, Fixed

Study	MD	95%−CI	P−value	Tau2	Tau	I2
Omitting Adu−Afarwuah 2015	0.11	[0.09; 0.14]	< 0.01	0.0015	0.0385	46%
Omitting Ashorn 2010	0.12	[0.09; 0.14]	< 0.01	0.0014	0.0378	45%
Omitting Fawzi 2007	0.11	[0.08; 0.14]	< 0.01	0.0022	0.0466	45%
Omitting Moore 2009	0.12	[0.09; 0.14]	< 0.01	0.0010	0.0321	38%
Omitting Osrin 2005	0.11	[0.09; 0.14]	< 0.01	0.0010	0.0315	36%
Omitting Roberfroid 2008	0.12	[0.09; 0.14]	< 0.01	0.0014	0.0368	43%
Omitting Sunawang 2009	0.12	[0.10; 0.15]	< 0.01	0.0003	0.0183	16%
Omitting Tofail 2008	0.12	[0.09; 0.14]	< 0.01	0.0010	0.0315	36%
Omitting West 2014	0.10	[0.06; 0.13]	< 0.01	0.0020	0.0450	39%
**Fixed effect model**	**0.12**	**[0.09; 0.14]**	**< 0.01**	**0.0012**	**0.0347**	**39%**

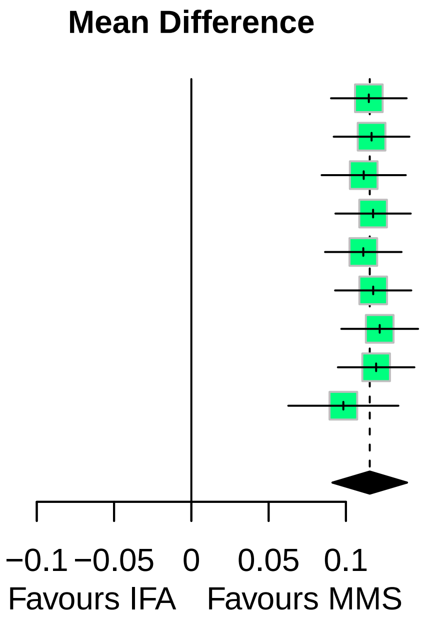

Leave−One−Out Sensitivity Analysis
WAZ at Birth, Random

Study	Mean Difference	MD	95%−CI	P−value	Tau2	Tau	I2
Omitting Adu−Afarwuah 2015		0.10	[0.05; 0.14]	< 0.01	0.0015	0.0385	46%
Omitting Ashorn 2010		0.10	[0.06; 0.14]	< 0.01	0.0014	0.0378	45%
Omitting Fawzi 2007		0.09	[0.04; 0.14]	< 0.01	0.0022	0.0466	45%
Omitting Moore 2009		0.11	[0.07; 0.14]	< 0.01	0.0010	0.0321	38%
Omitting Osrin 2005		0.09	[0.05; 0.13]	< 0.01	0.0010	0.0315	36%
Omitting Roberfroid 2008		0.10	[0.06; 0.15]	< 0.01	0.0014	0.0368	43%
Omitting Sunawang 2009		0.12	[0.09; 0.15]	< 0.01	0.0003	0.0183	16%
Omitting Tofail 2008		0.11	[0.07; 0.15]	< 0.01	0.0010	0.0315	36%
Omitting West 2014		0.09	[0.03; 0.14]	< 0.01	0.0020	0.0450	39%
**Random effects model**		**0.10**	**[0.06; 0.14]**	**< 0.01**	**0.0012**	**0.0347**	**39%**

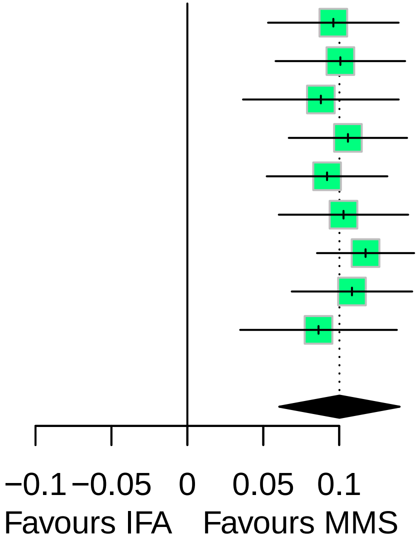

Leave−One−Out Sensitivity Analysis
WAZ at 3 Months, Fixed

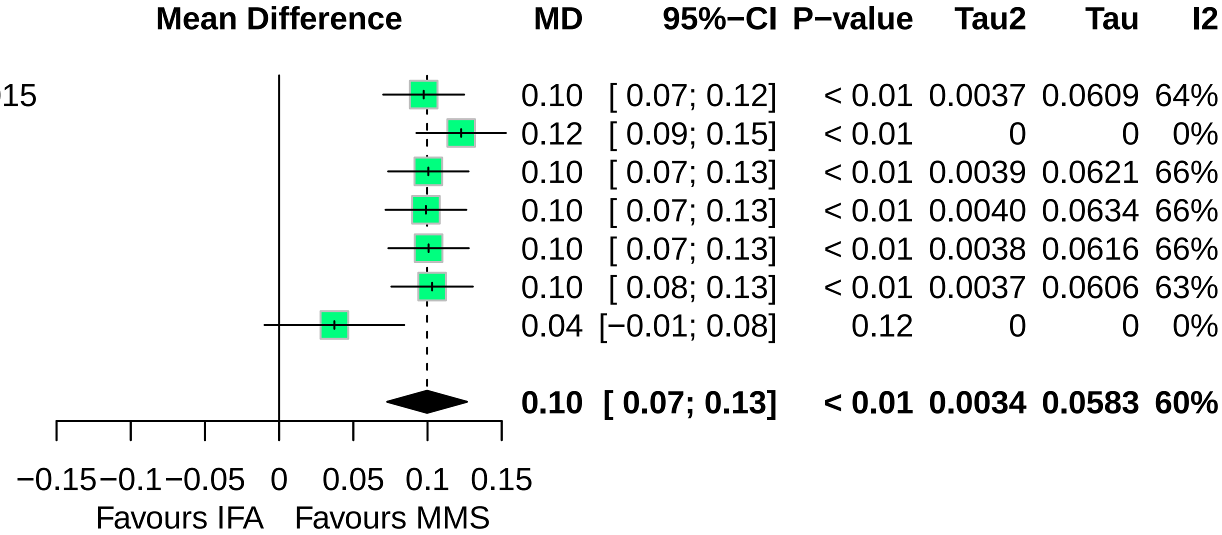

Study	MD	95%−CI	P−value	Tau2	Tau	I2
Omitting Adu−Afarwuah 2015	0.10	[0.07; 0.12]	< 0.01	0.0037	0.0609	64%
Omitting Fawzi 2007	0.12	[0.09; 0.15]	< 0.01	0	0	0%
Omitting Moore 2009	0.10	[0.07; 0.13]	< 0.01	0.0039	0.0621	66%
Omitting Roberfroid 2008	0.10	[0.07; 0.13]	< 0.01	0.0040	0.0634	66%
Omitting Sunawang 2009	0.10	[0.07; 0.13]	< 0.01	0.0038	0.0616	66%
Omitting Tofail 2008	0.10	[0.08; 0.13]	< 0.01	0.0037	0.0606	63%
Omitting West 2014	0.04	[−0.01; 0.08]	0.12	0	0	0%
**Fixed Effect Model**	**0.10**	**[0.07; 0.13]**	**< 0.01**	**0.0034**	**0.0583**	**60%**

Leave−One−Out Sensitivity Analysis
WAZ at 3 Months, Random

Study	MD	95%−CI	P−value	Tau2	Tau	I2
Omitting Adu−Afarwuah 2015	0.07	[−0.00; 0.14]	0.06	0.0037	0.0609	64%
Omitting Fawzi 2007	0.12	[0.09; 0.15]	< 0.01	0	0	0%
Omitting Moore 2009	0.08	[0.01; 0.15]	0.02	0.0039	0.0621	66%
Omitting Roberfroid 2008	0.07	[0.00; 0.14]	0.04	0.0040	0.0634	66%
Omitting Sunawang 2009	0.08	[0.01; 0.15]	0.02	0.0038	0.0616	66%
Omitting Tofail 2008	0.09	[0.02; 0.16]	0.01	0.0037	0.0606	63%
Omitting West 2014	0.04	[−0.01; 0.08]	0.12	0	0	0%
**Random effects model**	**0.08**	**[0.02; 0.14]**	**0.02**	**0.0034**	**0.0583**	**60%**

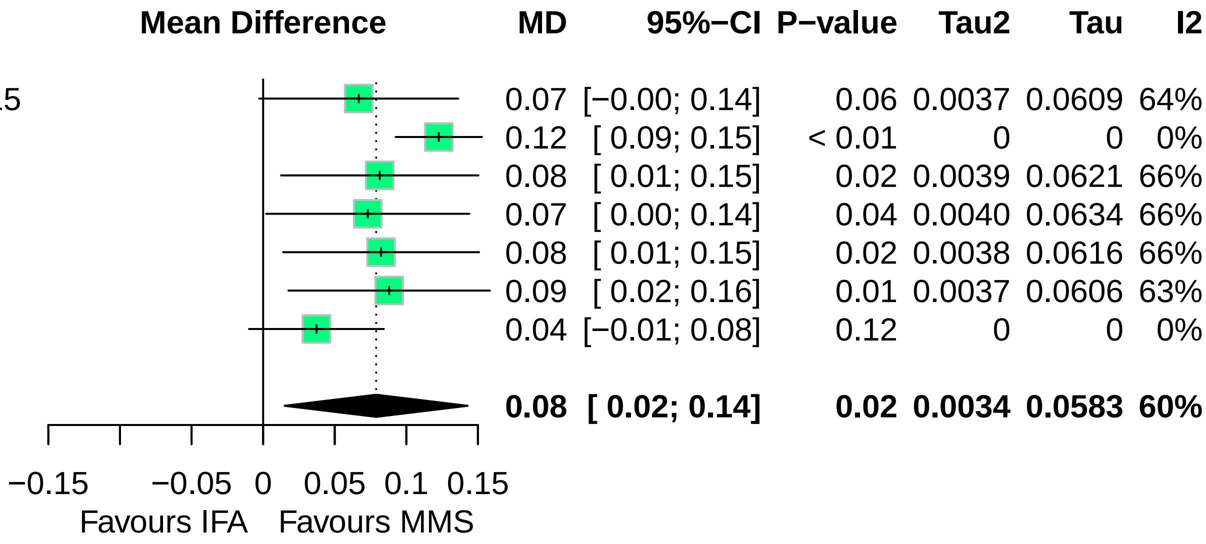

Leave−One−Out Sensitivity Analysis
WAZ at 6 Months, Fixed

Study	MD	95%−CI	P−value	Tau2	Tau	I2
Omitting Adu−Afarwuah 2015	0.03	[0.00; 0.06]	0.05	0	0	0%
Omitting Ashorn 2010 complete FU	0.04	[0.00; 0.07]	0.03	0	0	0%
Omitting Ashorn 2010 simplified FU	0.04	[0.00; 0.07]	0.02	0	0	0%
Omitting Fawzi 2007	0.04	[0.01; 0.08]	0.02	0	0	0%
Omitting Moore 2009	0.04	[0.00; 0.07]	0.03	0	0	0%
Omitting Roberfroid 2008	0.03	[0.00; 0.07]	0.04	0	0	0%
Omitting Sunawang 2009	0.04	[0.01; 0.07]	0.02	0	0	0%
Omitting Tofail 2008	0.04	[0.01; 0.07]	0.02	0	0	0%
Omitting West 2014	0.03	[−0.01; 0.08]	0.18	0	0	0%
**Fixed Effect Model**	**0.04**	**[0.00; 0.07]**	**0.02**	**0**	**0**	**0%**

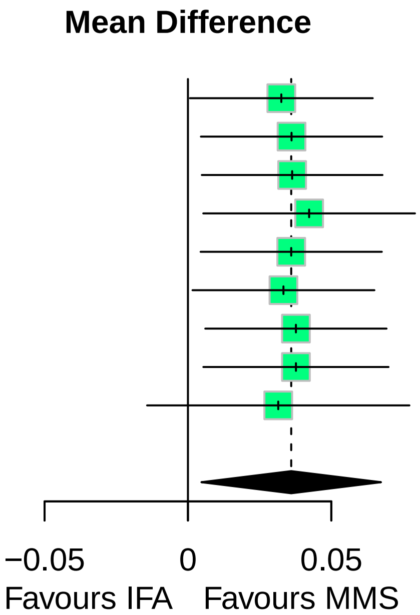

Leave−One−Out Sensitivity Analysis
WAZ at 6 Months, Random

Study	MD	95%−CI	P−value	Tau2	Tau	I2
Omitting Adu−Afarwuah 2015	0.03	[0.00; 0.06]	0.05	0	0	0%
Omitting Ashorn 2010 complete FU	0.04	[0.00; 0.07]	0.03	0	0	0%
Omitting Ashorn 2010 simplified FU	0.04	[0.00; 0.07]	0.02	0	0	0%
Omitting Fawzi 2007	0.04	[0.01; 0.08]	0.02	0	0	0%
Omitting Moore 2009	0.04	[0.00; 0.07]	0.03	0	0	0%
Omitting Roberfroid 2008	0.03	[0.00; 0.07]	0.04	0	0	0%
Omitting Sunawang 2009	0.04	[0.01; 0.07]	0.02	0	0	0%
Omitting Tofail 2008	0.04	[0.01; 0.07]	0.02	0	0	0%
Omitting West 2014	0.03	[−0.01; 0.08]	0.18	0	0	0%
**Random effects model**	**0.04**	**[0.00; 0.07]**	**0.02**	**0**	**0**	**0%**

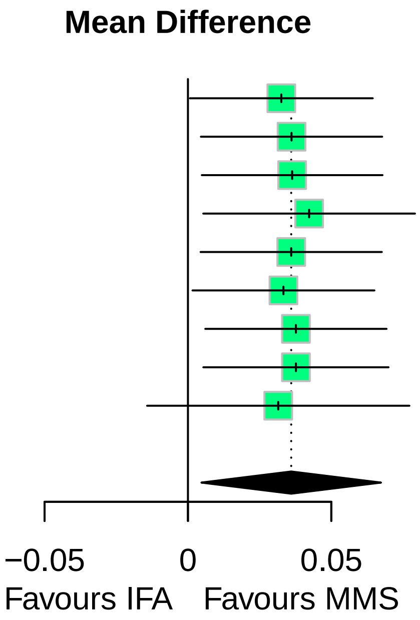

Leave−One−Out Sensitivity Analysis
WAZ at 12 Months, Fixed

Study	Mean Difference	MD	95%−CI	P−value	Tau2	Tau	I2
Omitting Adu−Afarwuah 2015		0.02	[−0.02; 0.05]	0.31	0	0	0%
Omitting Ashorn 2010 complete FU		0.03	[−0.01; 0.06]	0.11	0	0	0%
Omitting Fawzi 2007		0.02	[−0.01; 0.06]	0.18	0.0010	0.0317	19%
Omitting Roberfroid 2008		0.03	[−0.01; 0.06]	0.13	0.0003	0.0162	9%
Omitting Sunawang 2009		0.02	[−0.01; 0.06]	0.15	0.0006	0.0246	18%
Omitting Tofail 2008		0.02	[−0.01; 0.05]	0.23	0.0005	0.0226	14%
Omitting West 2014		0.03	[−0.03; 0.08]	0.30	0.0012	0.0352	18%
**Fixed Effect Model**		**0.02**	**[−0.01; 0.06]**	**0.16**	**< 0.0001**	**0.0097**	**3%**

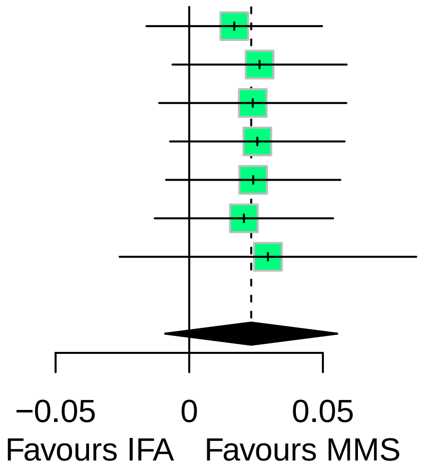

Leave−One−Out Sensitivity Analysis
WAZ at 12 Months, Random

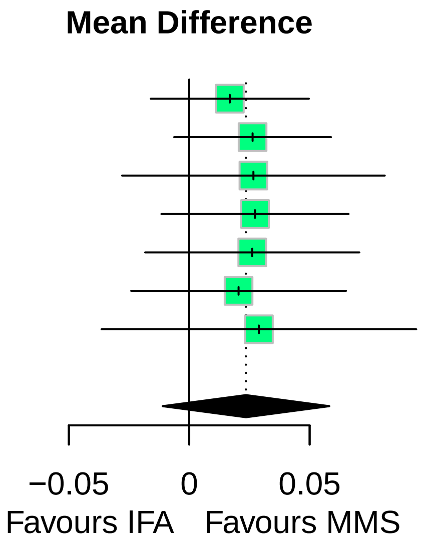

Study	MD	95%−CI	P−value	Tau2	Tau	I2
Omitting Adu−Afarwuah 2015	0.02	[−0.02; 0.05]	0.31	0	0	0%
Omitting Ashorn 2010 complete FU	0.03	[−0.01; 0.06]	0.11	0	0	0%
Omitting Fawzi 2007	0.03	[−0.03; 0.08]	0.34	0.0010	0.0317	19%
Omitting Roberfroid 2008	0.03	[−0.01; 0.07]	0.17	0.0003	0.0162	9%
Omitting Sunawang 2009	0.03	[−0.02; 0.07]	0.25	0.0006	0.0246	18%
Omitting Tofail 2008	0.02	[−0.02; 0.07]	0.37	0.0005	0.0226	14%
Omitting West 2014	0.03	[−0.04; 0.09]	0.39	0.0012	0.0352	18%
**Random effects model**	**0.02**	**[−0.01; 0.06]**	**0.18**	**< 0.0001**	**0.0097**	**3%**

Leave−One−Out Sensitivity Analysis
WAZ at 18 Months, Fixed

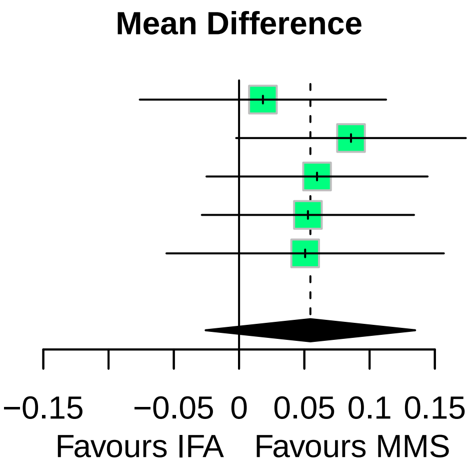

Study	MD	95%−CI	P−value	Tau2	Tau	I2
Omitting Adu−Afarwuah 2015	0.02	[−0.08; 0.11]	0.70	0	0	0%
Omitting Ashorn 2010 complete FU	0.09	[−0.00; 0.17]	0.06	0	0	0%
Omitting Ashorn 2010 simplified FU	0.06	[−0.02; 0.14]	0.17	0.0028	0.0529	24%
Omitting Fawzi 2007	0.05	[−0.03; 0.13]	0.20	0.0025	0.0504	25%
Omitting Tofail 2008	0.05	[−0.06; 0.16]	0.35	0.0049	0.0703	27%
**Fixed Effect Model**	**0.05**	**[−0.03; 0.13]**	**0.18**	**0.0002**	**0.0152**	**2%**

Leave−One−Out Sensitivity Analysis
WAZ at 18 Months, Random

Study	MD	95%−CI	P−value	Tau2	Tau	I2
Omitting Adu−Afarwuah 2015	0.02	[−0.08; 0.11]	0.70	0	0	0%
Omitting Ashorn 2010 complete FU	0.09	[−0.00; 0.17]	0.06	0	0	0%
Omitting Ashorn 2010 simplified FU	0.06	[−0.05; 0.16]	0.29	0.0028	0.0529	24%
Omitting Fawzi 2007	0.05	[−0.05; 0.15]	0.34	0.0025	0.0504	25%
Omitting Tofail 2008	0.04	[−0.09; 0.17]	0.53	0.0049	0.0703	27%
**Random effects model**	**0.05**	**[−0.03; 0.14]**	**0.19**	**0.0002**	**0.0152**	**2%**

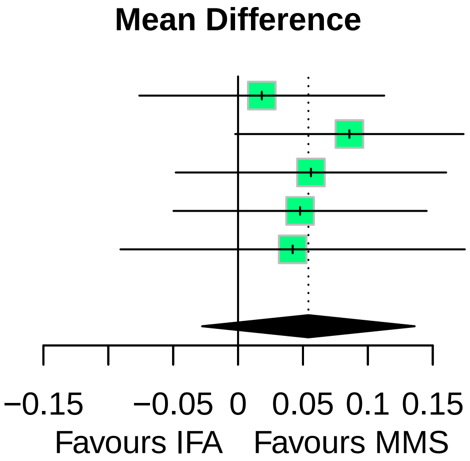

Leave−One−Out Sensitivity Analysis
WAZ at 24 Months, Fixed

Study	MD	95%−CI	P−value	Tau2	Tau	I2
Omitting Ashorn 2010 complete FU	0.01	[−0.03; 0.05]	0.66	0	0	0%
Omitting Bliznashka 2021	0.01	[−0.03; 0.05]	0.78	0	0	0%
Omitting Roberfroid 2008	0.00	[−0.04; 0.04]	0.87	0	0	0%
Omitting Tofail 2008	0.00	[−0.04; 0.05]	0.87	0	0	0%
Omitting West 2014	−0.02	[−0.11; 0.07]	0.69	0	0	0%
**Fixed Effect Model**	**0.00**	**[−0.04; 0.04]**	**0.83**	**0**	**0**	**0%**

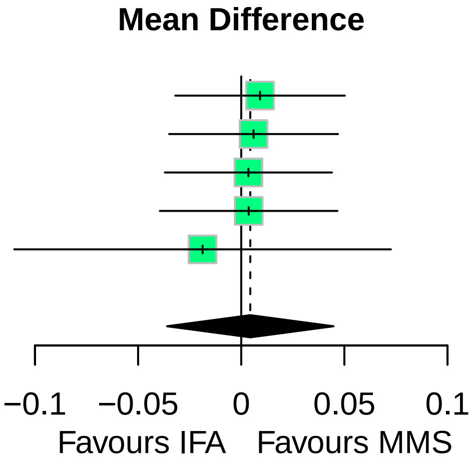

Leave−One−Out Sensitivity Analysis
WAZ at 24 Months, Random

Study	MD	95%−CI	P−value	Tau2	Tau	I2
Omitting Ashorn 2010 complete FU	0.01	[−0.03; 0.05]	0.66	0	0	0%
Omitting Bliznashka 2021	0.01	[−0.03; 0.05]	0.78	0	0	0%
Omitting Roberfroid 2008	0.00	[−0.04; 0.04]	0.87	0	0	0%
Omitting Tofail 2008	0.00	[−0.04; 0.05]	0.87	0	0	0%
Omitting West 2014	−0.02	[−0.11; 0.07]	0.69	0	0	0%
**Random effects model**	**0.00**	**[−0.04; 0.04]**	**0.83**	**0**	**0**	**0%**

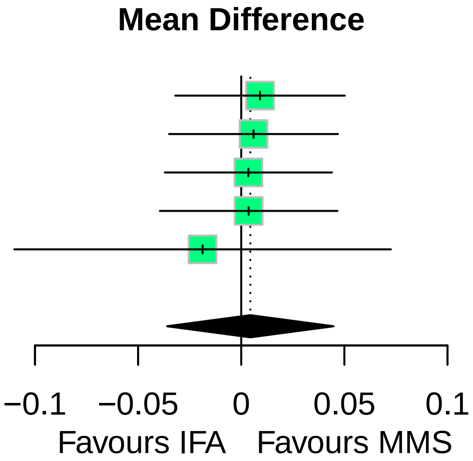

Leave−One−Out Sensitivity Analysis

WLAZ at Birth, Fixed

Study	MD	95%−CI	P−value	Tau2	Tau	I2
Omitting Adu−Afarwuah 2015	0.08	[0.05; 0.11]	< 0.01	0.0026	0.0506	46%
Omitting Ashorn 2010	0.09	[0.05; 0.12]	< 0.01	0.0022	0.0465	42%
Omitting Fawzi 2007	0.08	[0.05; 0.11]	< 0.01	0.0020	0.0452	41%
Omitting Osrin 2005	0.08	[0.05; 0.11]	< 0.01	0.0016	0.0397	34%
Omitting Roberfroid 2008	0.09	[0.06; 0.12]	< 0.01	0.0012	0.0340	27%
Omitting Sunawang 2009	0.09	[0.06; 0.12]	< 0.01	0.0022	0.0472	39%
Omitting Tofail 2008	0.08	[0.05; 0.11]	< 0.01	0.0032	0.0562	49%
Omitting West 2014	0.07	[0.01; 0.12]	0.01	0.0046	0.0680	47%
**Fixed effect model**	**0.08**	**[0.05; 0.11]**	**< 0.01**	**0.0022**	**0.0468**	**41%**

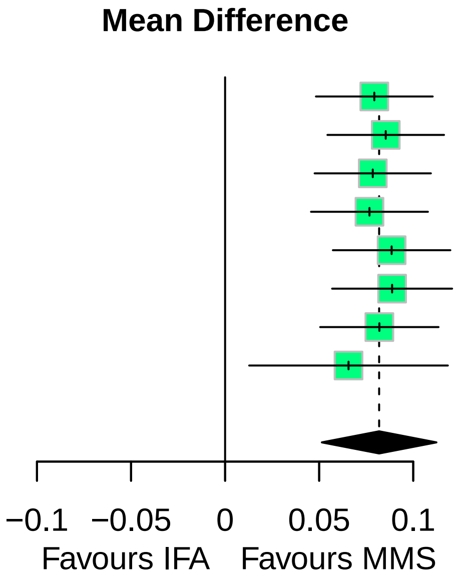

Leave−One−Out Sensitivity Analysis

WLAZ at Birth, Random

Study	MD	95%−CI	P−value	Tau2	Tau	I2
Omitting Adu−Afarwuah 2015	0.07	[0.01; 0.13]	0.02	0.0026	0.0506	46%
Omitting Ashorn 2010	0.09	[0.03; 0.14]	< 0.01	0.0022	0.0465	42%
Omitting Fawzi 2007	0.07	[0.01; 0.12]	0.02	0.0020	0.0452	41%
Omitting Osrin 2005	0.06	[0.01; 0.12]	0.02	0.0016	0.0397	34%
Omitting Roberfroid 2008	0.09	[0.04; 0.14]	< 0.01	0.0012	0.0340	27%
Omitting Sunawang 2009	0.09	[0.03; 0.15]	< 0.01	0.0022	0.0472	39%
Omitting Tofail 2008	0.08	[0.01; 0.14]	0.02	0.0032	0.0562	49%
Omitting West 2014	0.07	[−0.00; 0.15]	0.05	0.0046	0.0680	47%
**Random effects model**	**0.08**	**[0.02; 0.13]**	**< 0.01**	**0.0022**	**0.0468**	**41%**

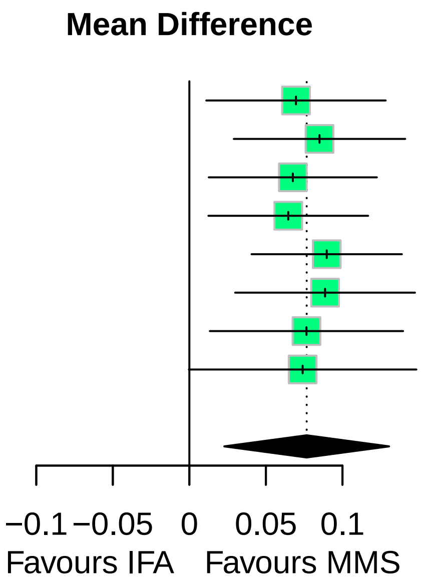

Leave−One−Out Sensitivity Analysis
WLAZ at 3 Months, Fixed

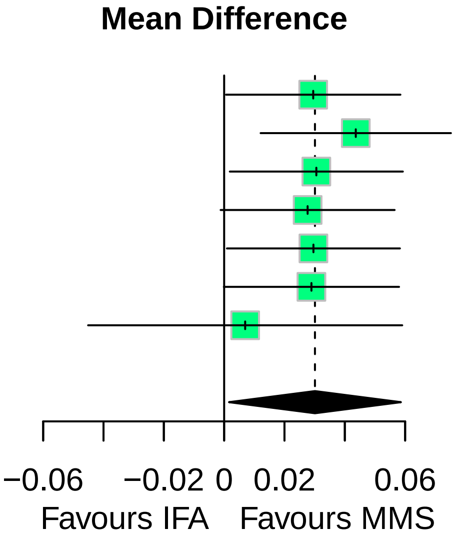

Study	MD	95%−CI	P−value	Tau2	Tau	I2
Omitting Adu−Afarwuah 2015	0.03	[0.00; 0.06]	0.05	0	0	0%
Omitting Fawzi 2007	0.04	[0.01; 0.08]	< 0.01	0	0	0%
Omitting Moore 2009	0.03	[0.00; 0.06]	0.04	0	0	0%
Omitting Roberfroid 2008	0.03	[−0.00; 0.06]	0.06	0	0	0%
Omitting Sunawang 2009	0.03	[0.00; 0.06]	0.04	0	0	0%
Omitting Tofail 2008	0.03	[−0.00; 0.06]	0.05	0	0	0%
Omitting West 2014	0.01	[−0.05; 0.06]	0.79	0	0	0%
**Fixed Effect Model**	**0.03**	**[0.00; 0.06]**	**0.04**	**0**	**0**	**0%**

Leave−One−Out Sensitivity Analysis
WLAZ at 3 Months, Random

Study	MD	95%−CI	P−value	Tau2	Tau	I2
Omitting Adu−Afarwuah 2015	0.03	[0.00; 0.06]	0.05	0	0	0%
Omitting Fawzi 2007	0.04	[0.01; 0.08]	< 0.01	0	0	0%
Omitting Moore 2009	0.03	[0.00; 0.06]	0.04	0	0	0%
Omitting Roberfroid 2008	0.03	[−0.00; 0.06]	0.06	0	0	0%
Omitting Sunawang 2009	0.03	[0.00; 0.06]	0.04	0	0	0%
Omitting Tofail 2008	0.03	[−0.00; 0.06]	0.05	0	0	0%
Omitting West 2014	0.01	[−0.05; 0.06]	0.79	0	0	0%
**Random effects model**	**0.03**	**[0.00; 0.06]**	**0.04**	**0**	**0**	**0%**

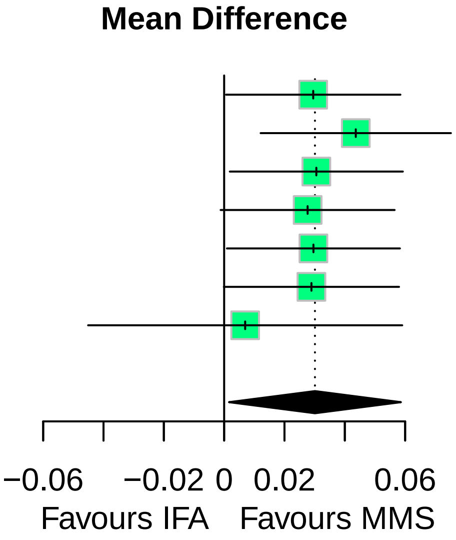

Leave−One−Out Sensitivity Analysis
WLAZ at 6 Months, Fixed

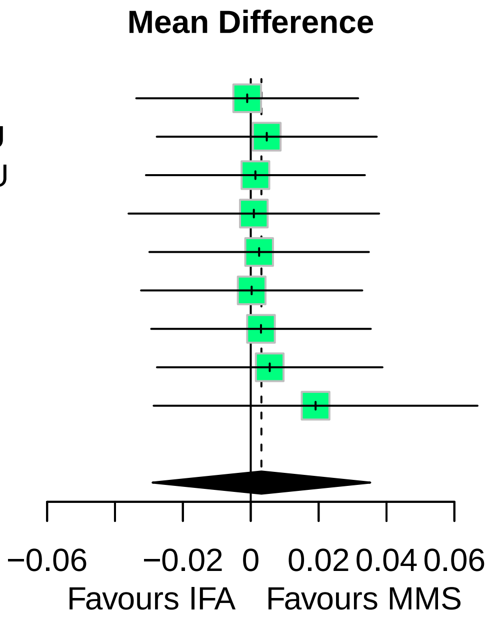

Study	MD	95%−CI	P−value	Tau2	Tau	I2
Omitting Adu−Afarwuah 2015	−0.00	[−0.03; 0.03]	0.95	0	0	0%
Omitting Ashorn 2010 complete FU	0.00	[−0.03; 0.04]	0.78	0	0	0%
Omitting Ashorn 2010 simplified FU	0.00	[−0.03; 0.03]	0.93	0	0	0%
Omitting Fawzi 2007	0.00	[−0.04; 0.04]	0.96	0	0	0%
Omitting Moore 2009	0.00	[−0.03; 0.03]	0.88	0	0	0%
Omitting Roberfroid 2008	0.00	[−0.03; 0.03]	0.99	0	0	0%
Omitting Sunawang 2009	0.00	[−0.03; 0.04]	0.85	0	0	0%
Omitting Tofail 2008	0.01	[−0.03; 0.04]	0.74	0	0	0%
Omitting West 2014	0.02	[−0.03; 0.07]	0.43	0	0	0%
**Fixed Effect Model**	**0.00**	**[−0.03; 0.04]**	**0.85**	**0**	**0**	**0%**

Leave−One−Out Sensitivity Analysis
WLAZ at 6 Months, Random

Study	MD	95%−CI	P−value	Tau2	Tau	I2
Omitting Adu−Afarwuah 2015	−0.00	[−0.03; 0.03]	0.95	0	0	0%
Omitting Ashorn 2010 complete FU	0.00	[−0.03; 0.04]	0.78	0	0	0%
Omitting Ashorn 2010 simplified FU	0.00	[−0.03; 0.03]	0.93	0	0	0%
Omitting Fawzi 2007	0.00	[−0.04; 0.04]	0.96	0	0	0%
Omitting Moore 2009	0.00	[−0.03; 0.03]	0.88	0	0	0%
Omitting Roberfroid 2008	0.00	[−0.03; 0.03]	0.99	0	0	0%
Omitting Sunawang 2009	0.00	[−0.03; 0.04]	0.85	0	0	0%
Omitting Tofail 2008	0.01	[−0.03; 0.04]	0.74	0	0	0%
Omitting West 2014	0.02	[−0.03; 0.07]	0.43	0	0	0%
**Random effects model**	**0.00**	**[−0.03; 0.04]**	**0.85**	**0**	**0**	**0%**

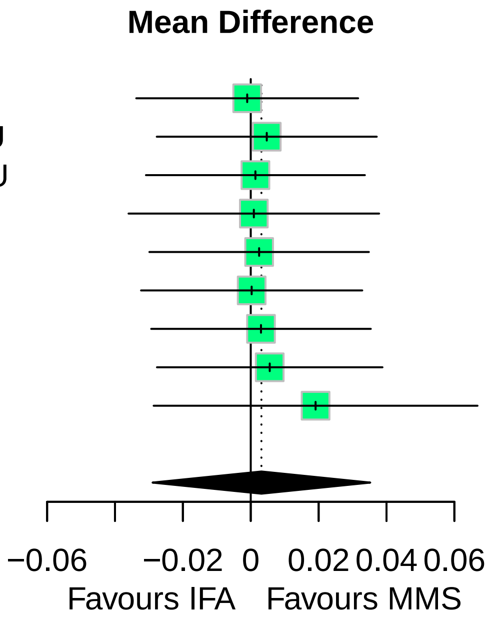

Leave−One−Out Sensitivity Analysis
WLAZ at 12 Months, Fixed

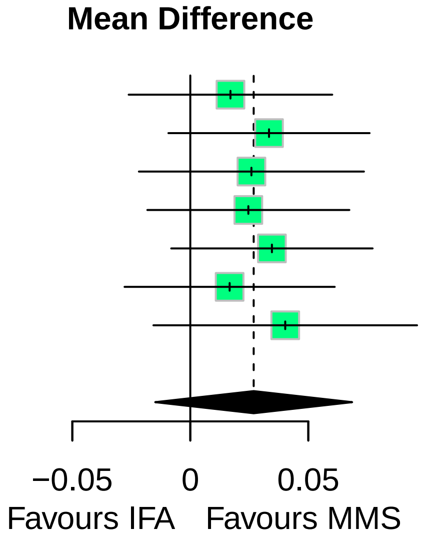

Study	MD	95%−CI	P−value	Tau2	Tau	I2
Omitting Adu−Afarwuah 2015	0.02	[−0.03; 0.06]	0.44	0.0010	0.0321	23%
Omitting Ashorn 2010 complete FU	0.03	[−0.01; 0.08]	0.13	0.0017	0.0408	33%
Omitting Fawzi 2007	0.03	[−0.02; 0.07]	0.29	0.0043	0.0659	48%
Omitting Roberfroid 2008	0.02	[−0.02; 0.07]	0.26	0.0030	0.0546	47%
Omitting Sunawang 2009	0.03	[−0.01; 0.08]	0.11	0.0012	0.0342	26%
Omitting Tofail 2008	0.02	[−0.03; 0.06]	0.46	0.0023	0.0478	37%
Omitting West 2014	0.04	[−0.02; 0.10]	0.16	0.0045	0.0668	45%
**Fixed Effect Model**	**0.03**	**[−0.01; 0.07]**	**0.21**	**0.0022**	**0.0473**	**37%**

Leave−One−Out Sensitivity Analysis
WLAZ at 12 Months, Random

Study	MD	95%−CI	P−value	Tau2	Tau	I2
Omitting Adu−Afarwuah 2015	0.02	[−0.04; 0.07]	0.57	0.0010	0.0321	23%
Omitting Ashorn 2010 complete FU	0.04	[−0.02; 0.10]	0.18	0.0017	0.0408	33%
Omitting Fawzi 2007	0.03	[−0.05; 0.11]	0.49	0.0043	0.0659	48%
Omitting Roberfroid 2008	0.02	[−0.04; 0.09]	0.47	0.0030	0.0546	47%
Omitting Sunawang 2009	0.04	[−0.01; 0.10]	0.15	0.0012	0.0342	26%
Omitting Tofail 2008	0.02	[−0.05; 0.08]	0.64	0.0023	0.0478	37%
Omitting West 2014	0.03	[−0.05; 0.12]	0.42	0.0045	0.0668	45%
**Random effects model**	**0.03**	**[−0.03; 0.09]**	**0.34**	**0.0022**	**0.0473**	**37%**

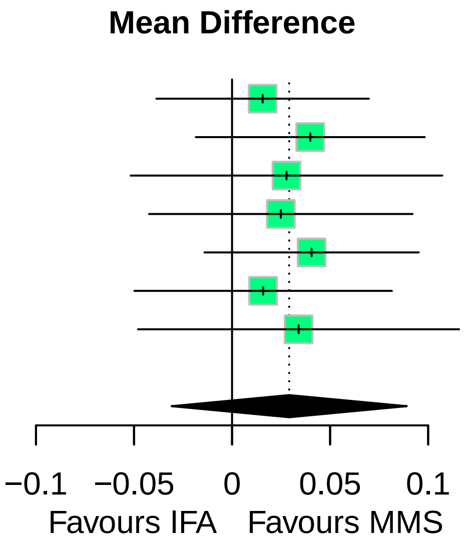

Leave−One−Out Sensitivity Analysis
WLAZ at 18 Months, Fixed

Study	Mean Difference	MD	95%−CI	P−value	Tau2	Tau	I2
Omitting Adu−Afarwuah 2015		0.04	[−0.05; 0.13]	0.35	0.0035	0.0589	23%
Omitting Ashorn 2010 complete FU		0.09	[0.01; 0.18]	0.03	0	0	0%
Omitting Ashorn 2010 simplified FU		0.06	[−0.02; 0.14]	0.13	0.0033	0.0573	29%
Omitting Fawzi 2007		0.06	[−0.02; 0.14]	0.14	0.0030	0.0548	30%
Omitting Tofail 2008		0.02	[−0.09; 0.12]	0.75	0	0	0%
**Fixed Effect Model**		**0.06**	**[−0.02; 0.14]**	**0.14**	**0.0007**	**0.0260**	**7%**

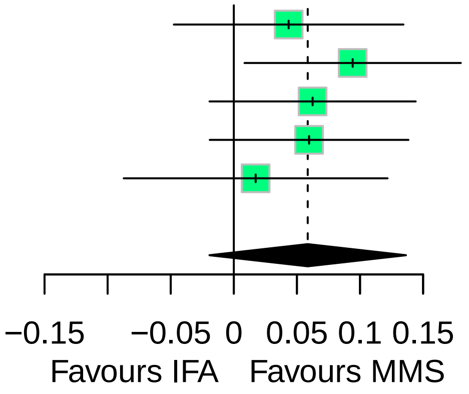

Leave−One−Out Sensitivity Analysis
WLAZ at 18 Months, Random

Study	MD	95%−CI	P−value	Tau2	Tau	I2
Omitting Adu−Afarwuah 2015	0.03	[−0.09; 0.14]	0.64	0.0035	0.0589	23%
Omitting Ashorn 2010 complete FU	0.09	[0.01; 0.18]	0.03	0	0	0%
Omitting Ashorn 2010 simplified FU	0.05	[−0.05; 0.16]	0.33	0.0033	0.0573	29%
Omitting Fawzi 2007	0.05	[−0.05; 0.15]	0.32	0.0030	0.0548	30%
Omitting Tofail 2008	0.02	[−0.09; 0.12]	0.75	0	0	0%
**Random effects model**	**0.06**	**[−0.03; 0.14]**	**0.19**	**0.0007**	**0.0260**	**7%**

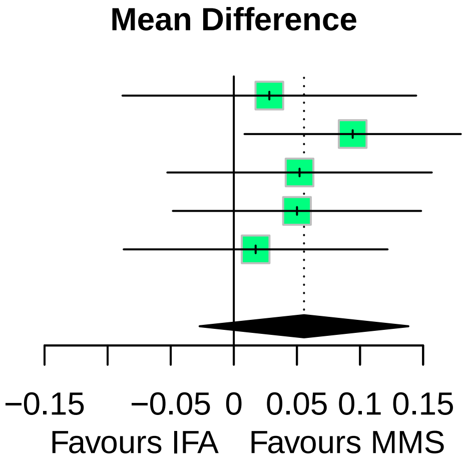

Leave−One−Out Sensitivity Analysis
WLAZ at 24 Months, Fixed

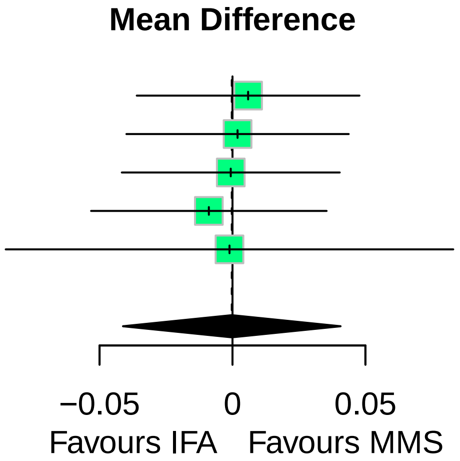

Study	MD	95%−CI	P−value	Tau2	Tau	I2
Omitting Ashorn 2010 complete FU	0.01	[−0.04; 0.05]	0.78	0	0	0%
Omitting Bliznashka 2021	0.00	[−0.04; 0.04]	0.93	0	0	0%
Omitting Roberfroid 2008	−0.00	[−0.04; 0.04]	0.98	0	0	0%
Omitting Tofail 2008	−0.01	[−0.05; 0.04]	0.69	0	0	0%
Omitting West 2014	−0.00	[−0.09; 0.08]	0.98	0	0	0%
**Fixed Effect Model**	**−0.00**	**[−0.04; 0.04]**	**0.99**	**0**	**0**	**0%**

Leave−One−Out Sensitivity Analysis
WLAZ at 24 Months, Random

Study	MD	95%−CI	P−value	Tau2	Tau	I2
Omitting Ashorn 2010 complete FU	0.01	[−0.04; 0.05]	0.78	0	0	0%
Omitting Bliznashka 2021	0.00	[−0.04; 0.04]	0.93	0	0	0%
Omitting Roberfroid 2008	−0.00	[−0.04; 0.04]	0.98	0	0	0%
Omitting Tofail 2008	−0.01	[−0.05; 0.04]	0.69	0	0	0%
Omitting West 2014	−0.00	[−0.09; 0.08]	0.98	0	0	0%
**Random effects model**	**−0.00**	**[−0.04; 0.04]**	**0.99**	**0**	**0**	**0%**

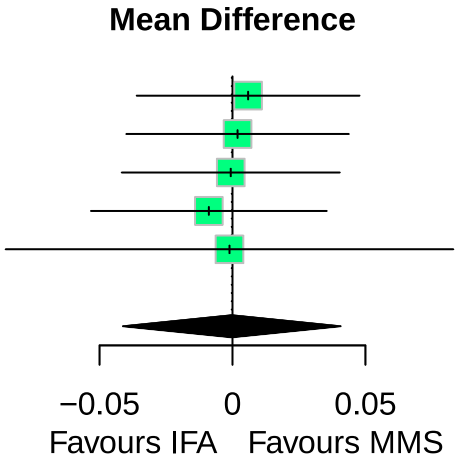

Leave−One−Out Sensitivity Analysis

HCAZ at Birth, Fixed

Study	MD	95%−CI	P−value	Tau2	Tau	I2
Omitting Adu−Afarwuah 2015	0.13	[0.09; 0.16]	< 0.01	0.0018	0.0421	37%
Omitting Ashorn 2010	0.12	[0.09; 0.16]	< 0.01	0.0023	0.0480	43%
Omitting Fawzi 2007	0.13	[0.09; 0.16]	< 0.01	0.0017	0.0418	32%
Omitting Roberfroid 2008	0.13	[0.09; 0.16]	< 0.01	0.0018	0.0420	35%
Omitting Sunawang 2009	0.13	[0.10; 0.16]	< 0.01	0.0005	0.0221	12%
Omitting Tofail 2008	0.12	[0.09; 0.15]	< 0.01	0.0017	0.0415	38%
Omitting West 2014	0.06	[0.00; 0.12]	0.05	0	0	0%
**Fixed effect model**	**0.12**	**[0.09; 0.15]**	**< 0.01**	**0.0015**	**0.0390**	**32%**

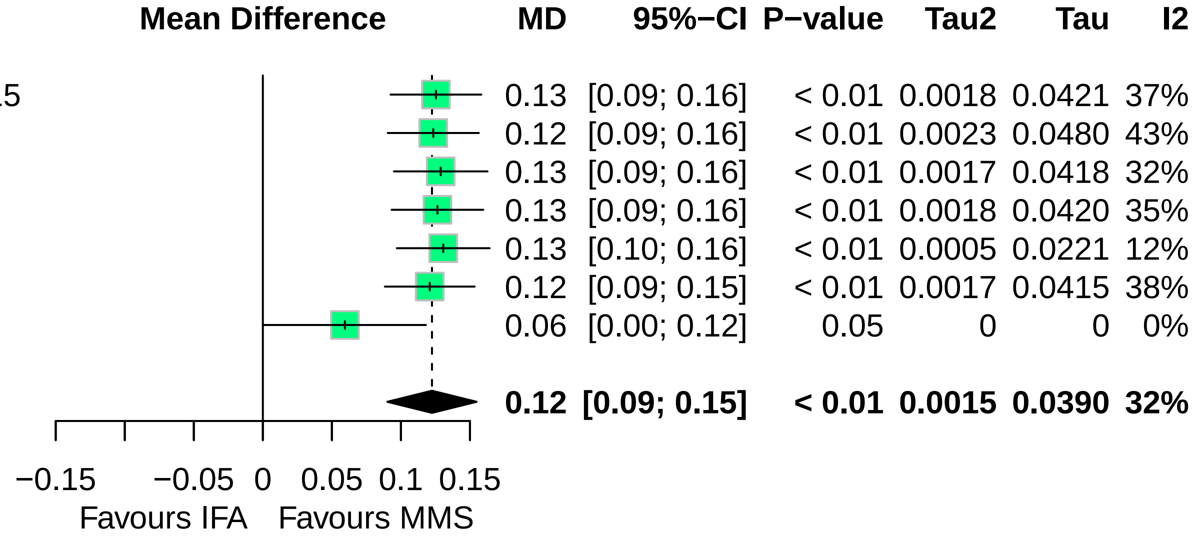

Leave−One−Out Sensitivity Analysis
HCAZ at Birth, Random

Study	MD	95%−CI	P−value	Tau2	Tau	I2
Omitting Adu−Afarwuah 2015	0.10	[0.04; 0.16]	< 0.01	0.0018	0.0421	37%
Omitting Ashorn 2010	0.09	[0.03; 0.15]	< 0.01	0.0023	0.0480	43%
Omitting Fawzi 2007	0.10	[0.04; 0.16]	< 0.01	0.0017	0.0418	32%
Omitting Roberfroid 2008	0.10	[0.04; 0.16]	< 0.01	0.0018	0.0420	35%
Omitting Sunawang 2009	0.12	[0.07; 0.16]	< 0.01	0.0005	0.0221	12%
Omitting Tofail 2008	0.09	[0.03; 0.15]	< 0.01	0.0017	0.0415	38%
Omitting West 2014	0.06	[0.00; 0.12]	0.05	0	0	0%
**Random effects model**	**0.10**	**[0.04; 0.15]**	**< 0.01**	**0.0015**	**0.0390**	**32%**

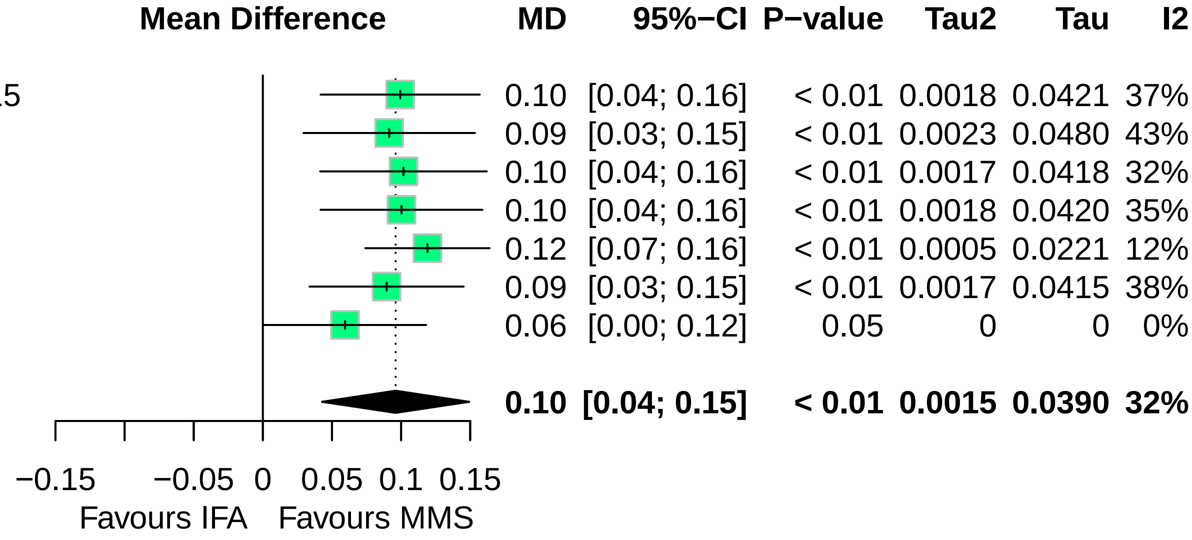

Leave−One−Out Sensitivity Analysis
HCAZ at 3 Months, Fixed

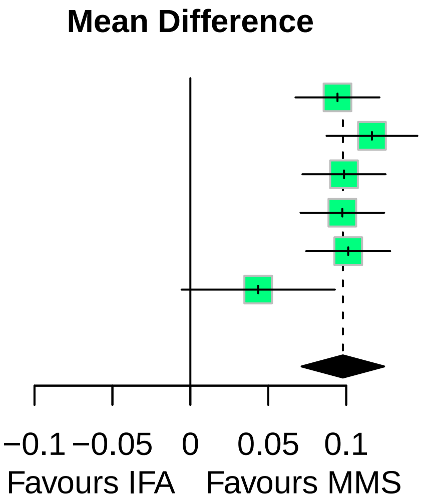

Study	MD	95%−CI	P−value	Tau2	Tau	I2
Omitting Adu−Afarwuah 2015	0.09	[0.07; 0.12]	< 0.01	0.0032	0.0569	64%
Omitting Fawzi 2007	0.12	[0.09; 0.15]	< 0.01	0	0	0%
Omitting Moore 2009	0.10	[0.07; 0.13]	< 0.01	0.0037	0.0607	69%
Omitting Roberfroid 2008	0.10	[0.07; 0.12]	< 0.01	0.0039	0.0624	69%
Omitting Tofail 2008	0.10	[0.07; 0.13]	< 0.01	0.0032	0.0563	64%
Omitting West 2014	0.04	[−0.01; 0.09]	0.08	0.0025	0.0502	37%
**Fixed Effect Model**	**0.10**	**[0.07; 0.12]**	**< 0.01**	**0.0031**	**0.0561**	**61%**

Leave−One−Out Sensitivity Analysis
HCAZ at 3 Months, Random

Study	MD	95%−CI	P−value	Tau2	Tau	I2
Omitting Adu−Afarwuah 2015	0.06	[−0.00; 0.13]	0.07	0.0032	0.0569	64%
Omitting Fawzi 2007	0.12	[0.09; 0.15]	< 0.01	0	0	0%
Omitting Moore 2009	0.08	[0.01; 0.15]	0.02	0.0037	0.0607	69%
Omitting Roberfroid 2008	0.08	[0.00; 0.15]	0.04	0.0039	0.0624	69%
Omitting Tofail 2008	0.09	[0.02; 0.16]	< 0.01	0.0032	0.0563	64%
Omitting West 2014	0.06	[−0.01; 0.13]	0.10	0.0025	0.0502	37%
**Random effects model**	**0.08**	**[0.02; 0.14]**	**0.01**	**0.0031**	**0.0561**	**61%**

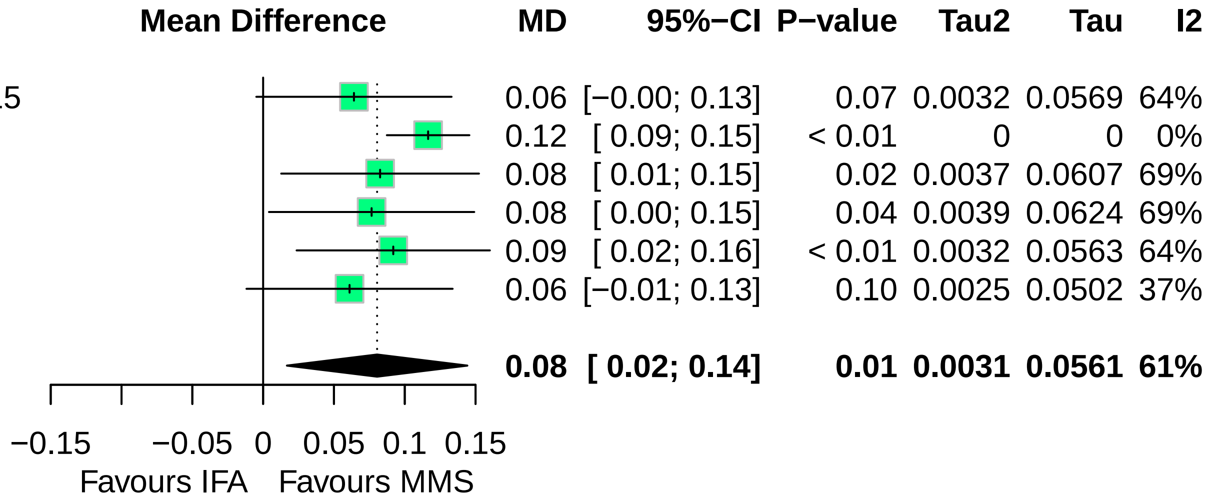

Leave−One−Out Sensitivity Analysis
HCAZ at 6 Months, Fixed

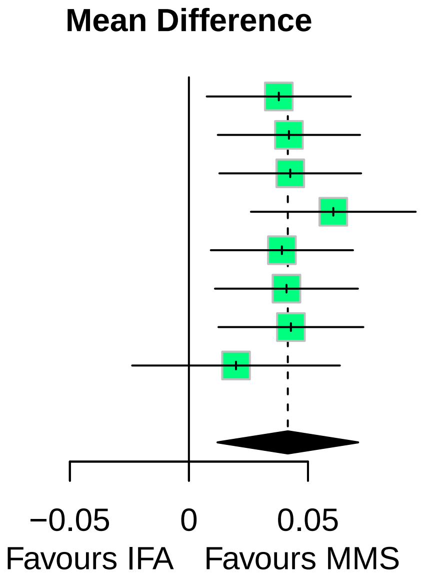

Study	MD	95%−CI	P−value	Tau2	Tau	I2
Omitting Adu−Afarwuah 2015	0.04	[0.01; 0.07]	0.01	0	0	0%
Omitting Ashorn 2010 complete FU	0.04	[0.01; 0.07]	< 0.01	0.0004	0.0194	14%
Omitting Ashorn 2010 simplified FU	0.04	[0.01; 0.07]	< 0.01	0.0003	0.0163	11%
Omitting Fawzi 2007	0.06	[0.03; 0.10]	< 0.01	0	0	0%
Omitting Moore 2009	0.04	[0.01; 0.07]	0.01	0	0	0%
Omitting Roberfroid 2008	0.04	[0.01; 0.07]	< 0.01	0.0004	0.0195	14%
Omitting Tofail 2008	0.04	[0.01; 0.07]	< 0.01	0.0004	0.0194	13%
Omitting West 2014	0.02	[−0.02; 0.06]	0.37	0	0	0%
**Fixed Effect Model**	**0.04**	**[0.01; 0.07]**	**< 0.01**	**< 0.0001**	**0.0033**	**0%**

Leave−One−Out Sensitivity Analysis
HCAZ at 6 Months, Random

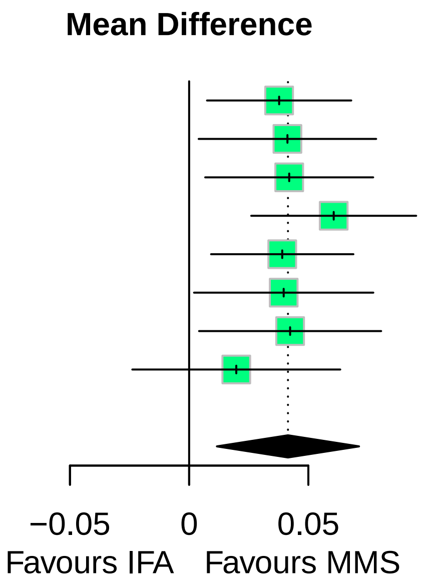

Study	MD	95%−CI	P−value	Tau2	Tau	I2
Omitting Adu−Afarwuah 2015	0.04	[0.01; 0.07]	0.01	0	0	0%
Omitting Ashorn 2010 complete FU	0.04	[0.00; 0.08]	0.03	0.0004	0.0194	14%
Omitting Ashorn 2010 simplified FU	0.04	[0.01; 0.08]	0.02	0.0003	0.0163	11%
Omitting Fawzi 2007	0.06	[0.03; 0.10]	< 0.01	0	0	0%
Omitting Moore 2009	0.04	[0.01; 0.07]	0.01	0	0	0%
Omitting Roberfroid 2008	0.04	[0.00; 0.08]	0.04	0.0004	0.0195	14%
Omitting Tofail 2008	0.04	[0.00; 0.08]	0.03	0.0004	0.0194	13%
Omitting West 2014	0.02	[−0.02; 0.06]	0.37	0	0	0%
**Random effects model**	**0.04**	**[0.01; 0.07]**	**< 0.01**	**< 0.0001**	**0.0033**	**0%**

Leave−One−Out Sensitivity Analysis

HCAZ at 12 Months, Fixed

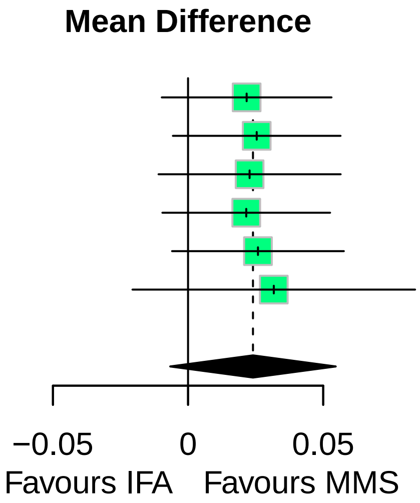

Study	MD	95%−CI	P−value	Tau2	Tau	I2
Omitting Adu−Afarwuah 2015	0.02	[−0.01; 0.05]	0.18	0	0	0%
Omitting Ashorn 2010 complete FU	0.03	[−0.01; 0.06]	0.11	0	0	0%
Omitting Fawzi 2007	0.02	[−0.01; 0.06]	0.19	0	0	0%
Omitting Roberfroid 2008	0.02	[−0.01; 0.05]	0.17	0	0	0%
Omitting Tofail 2008	0.03	[−0.01; 0.06]	0.11	0	0	0%
Omitting West 2014	0.03	[−0.02; 0.08]	0.23	0	0	0%
**Fixed Effect Model**	**0.02**	**[−0.01; 0.05]**	**0.12**	**0**	**0**	**0%**

Leave−One−Out Sensitivity Analysis
HCAZ at 12 Months, Random

Study	MD	95%−CI	P−value	Tau2	Tau	I2
Omitting Adu−Afarwuah 2015	0.02	[−0.01; 0.05]	0.18	0	0	0%
Omitting Ashorn 2010 complete FU	0.03	[−0.01; 0.06]	0.11	0	0	0%
Omitting Fawzi 2007	0.02	[−0.01; 0.06]	0.19	0	0	0%
Omitting Roberfroid 2008	0.02	[−0.01; 0.05]	0.17	0	0	0%
Omitting Tofail 2008	0.03	[−0.01; 0.06]	0.11	0	0	0%
Omitting West 2014	0.03	[−0.02; 0.08]	0.23	0	0	0%
**Random effects model**	**0.02**	**[−0.01; 0.05]**	**0.12**	**0**	**0**	**0%**

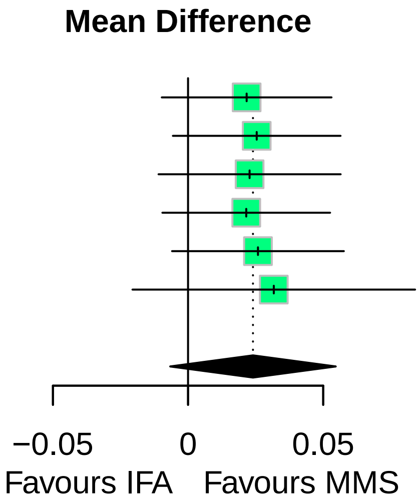

Leave−One−Out Sensitivity Analysis
HCAZ at 18 Months, Fixed

Study	MD	95%−CI	P−value	Tau2	Tau	I2
Omitting Adu−Afarwuah 2015	−0.00	[−0.14; 0.14]	0.97	0	0	0%
Omitting Ashorn 2010 complete FU	0.06	[−0.05; 0.17]	0.28	0	0	0%
Omitting Ashorn 2010 simplified FU	0.05	[−0.06; 0.15]	0.39	0	0	0%
Omitting Fawzi 2007	0.04	[−0.06; 0.14]	0.43	0	0	0%
**Fixed Effect Model**	**0.04**	**[−0.06; 0.14]**	**0.42**	**0**	**0**	**0%**

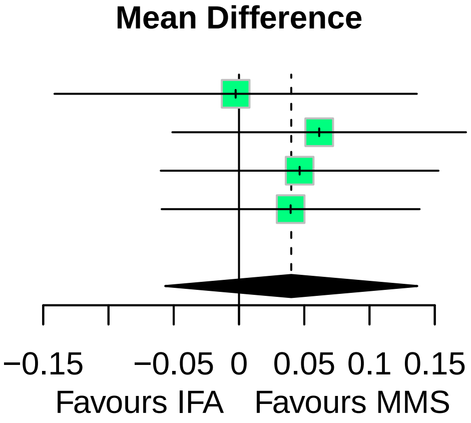

Leave−One−Out Sensitivity Analysis
HCAZ at 18 Months, Random

Study	Mean Difference	MD	95%−CI	P−value	Tau2	Tau	I2
Omitting Adu−Afarwuah 2015		−0.00	[−0.14; 0.14]	0.97	0	0	0%
Omitting Ashorn 2010 complete FU		0.06	[−0.05; 0.17]	0.28	0	0	0%
Omitting Ashorn 2010 simplified FU		0.05	[−0.06; 0.15]	0.39	0	0	0%
Omitting Fawzi 2007		0.04	[−0.06; 0.14]	0.43	0	0	0%
**Random effects model**		**0.04**	**[−0.06; 0.14]**	**0.42**	**0**	**0**	**0%**

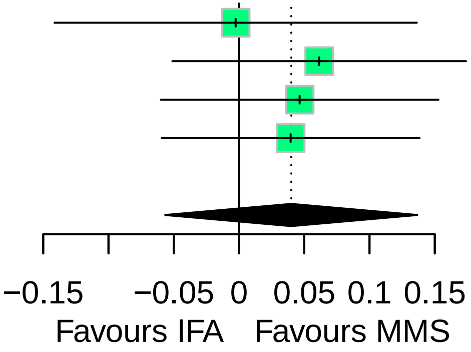

Leave−One−Out Sensitivity Analysis
HCAZ at 24 Months, Fixed

Study	MD	95%−CI	P−value	Tau2	Tau	I2
Omitting Ashorn 2010 complete FU	0.01	[−0.03; 0.05]	0.66	.	.	.
Omitting West 2014	−0.14	[−0.35; 0.07]	0.20	.	.	.
**Fixed Effect Model**	**0.00**	**[−0.04; 0.05]**	**0.87**	**0.0051**	**0.0716**	**46%**

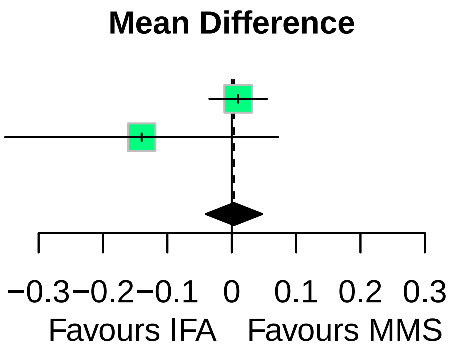

Leave−One−Out Sensitivity Analysis
HCAZ at 24 Months, Random

Study	Mean Difference	MD	95%−CI	P−value	Tau2	Tau	I2
Omitting Ashorn 2010 complete FU		0.01	[−0.03; 0.05]	0.66	.	.	.
Omitting West 2014		−0.14	[−0.35; 0.07]	0.20	.	.	.
**Random effects model**		**−0.03**	**[−0.16; 0.10]**	**0.67**	**0.0051**	**0.0716**	**46%**

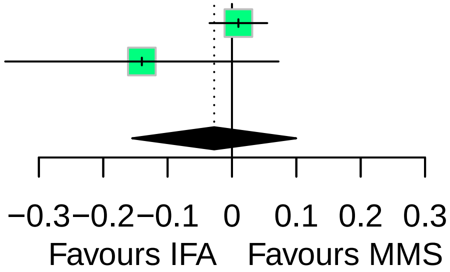

Leave−One−Out Sensitivity Analysis
MUACZ at 3 Months, Fixed

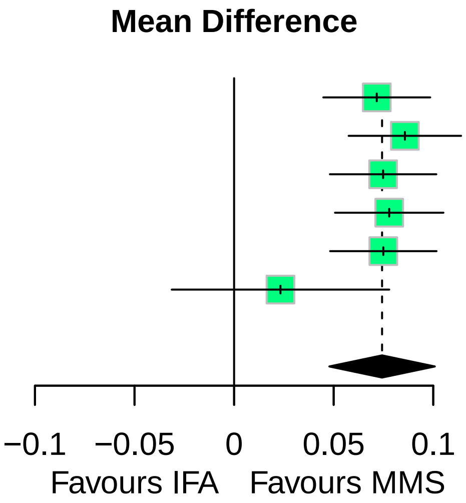

Study	MD	95%−CI	P−value	Tau2	Tau	I2
Omitting Adu−Afarwuah 2015	0.07	[0.04; 0.10]	< 0.01	0.0016	0.0396	43%
Omitting Fawzi 2007	0.09	[0.06; 0.11]	< 0.01	0	0	0%
Omitting Moore 2009	0.07	[0.05; 0.10]	< 0.01	0.0022	0.0465	52%
Omitting Roberfroid 2008	0.08	[0.05; 0.11]	< 0.01	0.0018	0.0430	44%
Omitting Sunawang 2009	0.07	[0.05; 0.10]	< 0.01	0.0021	0.0463	52%
Omitting West 2014	0.02	[−0.03; 0.08]	0.40	< 0.0001	0.0076	1%
**Fixed Effect Model**	**0.07**	**[0.05; 0.10]**	**< 0.01**	**0.0016**	**0.0398**	**41%**

Leave−One−Out Sensitivity Analysis

MUACZ at 3 Months, Random

Study	Mean Difference	MD	95%−CI	P−value	Tau2	Tau	I2
Omitting Adu−Afarwuah 2015		0.04	[−0.01; 0.10]	0.11	0.0016	0.0396	43%
Omitting Fawzi 2007		0.09	[0.06; 0.11]	< 0.01	0	0	0%
Omitting Moore 2009		0.06	[−0.00; 0.12]	0.07	0.0022	0.0465	52%
Omitting Roberfroid 2008		0.06	[0.00; 0.12]	0.04	0.0018	0.0430	44%
Omitting Sunawang 2009		0.06	[−0.00; 0.12]	0.07	0.0021	0.0463	52%
Omitting West 2014		0.02	[−0.03; 0.08]	0.40	< 0.0001	0.0076	1%
**Random effects model**		**0.06**	**[0.00; 0.11]**	**0.04**	**0.0016**	**0.0398**	**41%**

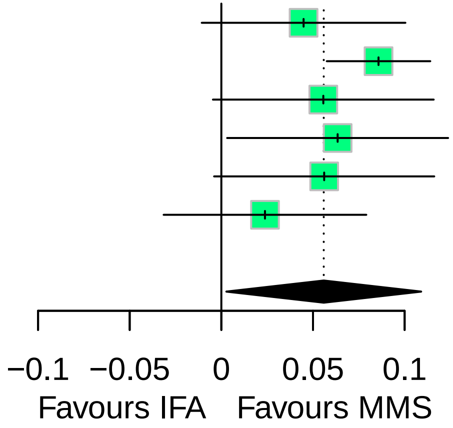

Leave−One−Out Sensitivity Analysis

MUACZ at 6 Months, Fixed

Study	MD	95%−CI	P−value	Tau2	Tau	I2
Omitting Adu−Afarwuah 2015	0.02	[−0.01; 0.05]	0.23	0	0	0%
Omitting Ashorn 2010 complete FU	0.02	[−0.01; 0.05]	0.20	0	0	0%
Omitting Ashorn 2010 simplified FU	0.02	[−0.01; 0.05]	0.15	0	0	0%
Omitting Fawzi 2007	0.03	[−0.00; 0.06]	0.09	0	0	0%
Omitting Moore 2009	0.02	[−0.01; 0.05]	0.19	0	0	0%
Omitting Roberfroid 2008	0.02	[−0.01; 0.05]	0.26	0	0	0%
Omitting Sunawang 2009	0.02	[−0.01; 0.06]	0.13	0	0	0%
Omitting West 2014	0.03	[−0.03; 0.08]	0.32	0	0	0%
**Fixed Effect Model**	**0.02**	**[−0.01; 0.05]**	**0.16**	**0**	**0**	**0%**

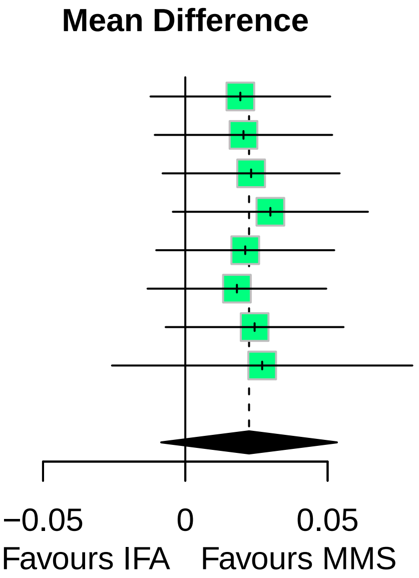

Leave−One−Out Sensitivity Analysis
MUACZ at 6 Months, Random

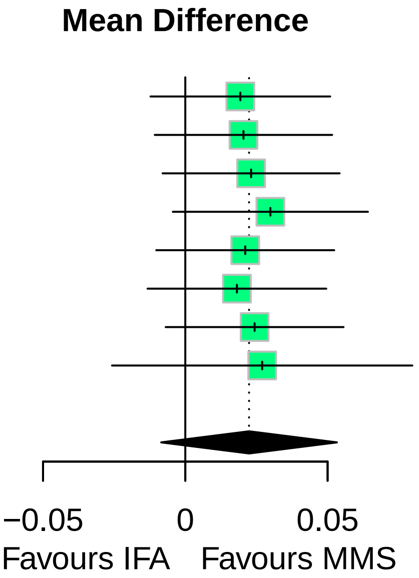

Study	MD	95%−CI	P−value	Tau2	Tau	I2
Omitting Adu−Afarwuah 2015	0.02	[−0.01; 0.05]	0.23	0	0	0%
Omitting Ashorn 2010 complete FU	0.02	[−0.01; 0.05]	0.20	0	0	0%
Omitting Ashorn 2010 simplified FU	0.02	[−0.01; 0.05]	0.15	0	0	0%
Omitting Fawzi 2007	0.03	[−0.00; 0.06]	0.09	0	0	0%
Omitting Moore 2009	0.02	[−0.01; 0.05]	0.19	0	0	0%
Omitting Roberfroid 2008	0.02	[−0.01; 0.05]	0.26	0	0	0%
Omitting Sunawang 2009	0.02	[−0.01; 0.06]	0.13	0	0	0%
Omitting West 2014	0.03	[−0.03; 0.08]	0.32	0	0	0%
**Random effects model**	**0.02**	**[−0.01; 0.05]**	**0.16**	**0**	**0**	**0%**

Leave−One−Out Sensitivity Analysis
MUACZ at 12 Months, Fixed

Study	MD	95%−CI	P−value	Tau2	Tau	I2
Omitting Adu−Afarwuah 2015	0.01	[−0.02; 0.04]	0.50	0	0	0%
Omitting Ashorn 2010 complete FU	0.02	[−0.01; 0.05]	0.20	0.0001	0.0116	5%
Omitting Fawzi 2007	0.02	[−0.02; 0.05]	0.30	0.0012	0.0344	20%
Omitting Roberfroid 2008	0.02	[−0.01; 0.05]	0.27	0.0006	0.0246	19%
Omitting Sunawang 2009	0.02	[−0.01; 0.05]	0.27	0.0006	0.0244	20%
Omitting West 2014	0.04	[−0.02; 0.10]	0.19	0.0004	0.0203	6%
**Fixed Effect Model**	**0.02**	**[−0.01; 0.05]**	**0.25**	**< 0.0001**	**0.0020**	**0%**

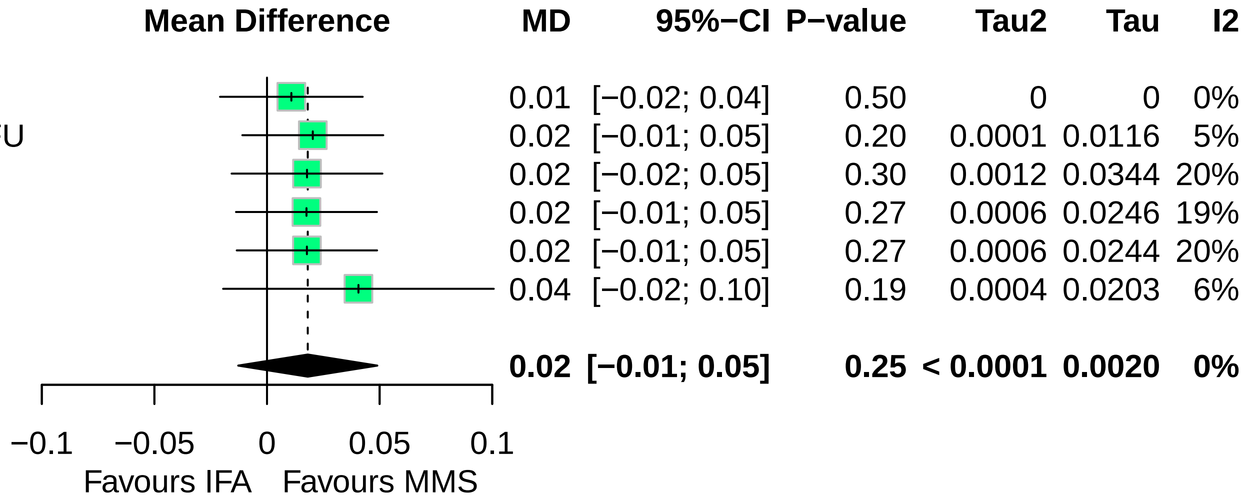

Leave−One−Out Sensitivity Analysis
MUACZ at 12 Months, Random

Study	Mean Difference	MD	95%−CI	P−value	Tau2	Tau	I2
Omitting Adu−Afarwuah 2015		0.01	[−0.02; 0.04]	0.50	0	0	0%
Omitting Ashorn 2010 complete FU		0.02	[−0.01; 0.06]	0.21	0.0001	0.0116	5%
Omitting Fawzi 2007		0.03	[−0.03; 0.09]	0.33	0.0012	0.0344	20%
Omitting Roberfroid 2008		0.02	[−0.02; 0.07]	0.31	0.0006	0.0246	19%
Omitting Sunawang 2009		0.02	[−0.02; 0.07]	0.30	0.0006	0.0244	20%
Omitting West 2014		0.04	[−0.02; 0.11]	0.20	0.0004	0.0203	6%
**Random effects model**		**0.02**	**[−0.01; 0.05]**	**0.25**	**< 0.0001**	**0.0020**	**0%**

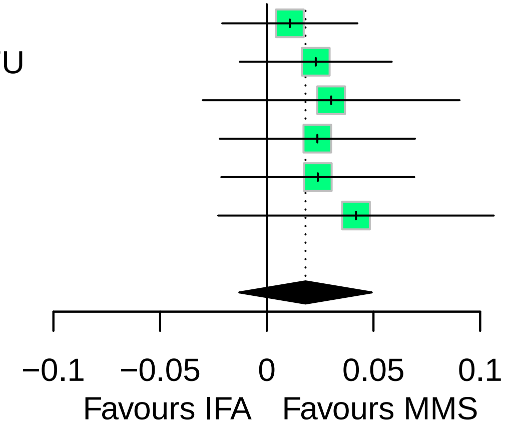

Leave−One−Out Sensitivity Analysis
MUACZ at 18 Months, Fixed

Study	Mean Difference	MD	95%−CI	P−value	Tau2	Tau	I2
Omitting Adu−Afarwuah 2015		0.01	[−0.13; 0.15]	0.92	0	0	0%
Omitting Ashorn 2010 complete FU		0.10	[−0.02; 0.21]	0.10	0	0	0%
Omitting Ashorn 2010 simplified FU		0.06	[−0.05; 0.17]	0.27	0.0007	0.0269	6%
Omitting Fawzi 2007		0.07	[−0.03; 0.17]	0.15	0	0	0%
**Fixed Effect Model**		**0.06**	**[−0.03; 0.16]**	**0.19**	**0**	**0**	**0%**

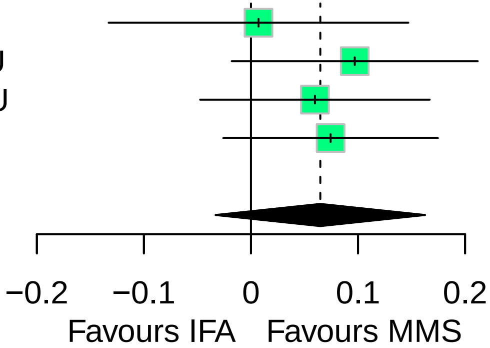

Leave−One−Out Sensitivity Analysis
MUACZ at 18 Months, Random

Study	MD	95%−CI	P−value	Tau2	Tau	I2
Omitting Adu−Afarwuah 2015	0.01	[−0.13; 0.15]	0.92	0	0	0%
Omitting Ashorn 2010 complete FU	0.10	[−0.02; 0.21]	0.10	0	0	0%
Omitting Ashorn 2010 simplified FU	0.06	[−0.06; 0.17]	0.33	0.0007	0.0269	6%
Omitting Fawzi 2007	0.07	[−0.03; 0.17]	0.15	0	0	0%
**Random effects model**	**0.06**	**[−0.03; 0.16]**	**0.19**	**0**	**0**	**0%**

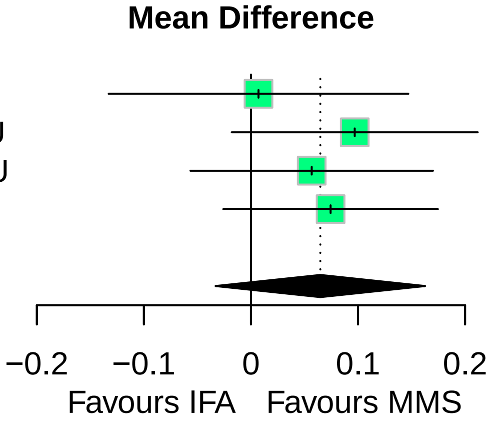

Leave−One−Out Sensitivity Analysis

MUACZ at 24 Months, Fixed

Study	MD	95%−CI	P−value	Tau2	Tau	I2
Omitting Ashorn 2010 complete FU	0.00	[−0.04; 0.04]	0.98	0	0	0%
Omitting Roberfroid 2008	−0.01	[−0.05; 0.03]	0.72	0.0110	0.1047	68%
Omitting West 2014	−0.14	[−0.31; 0.04]	0.13	0	0	0%
**Fixed Effect Model**	**−0.01**	**[−0.05; 0.03]**	**0.73**	**0.0049**	**0.0698**	**36%**

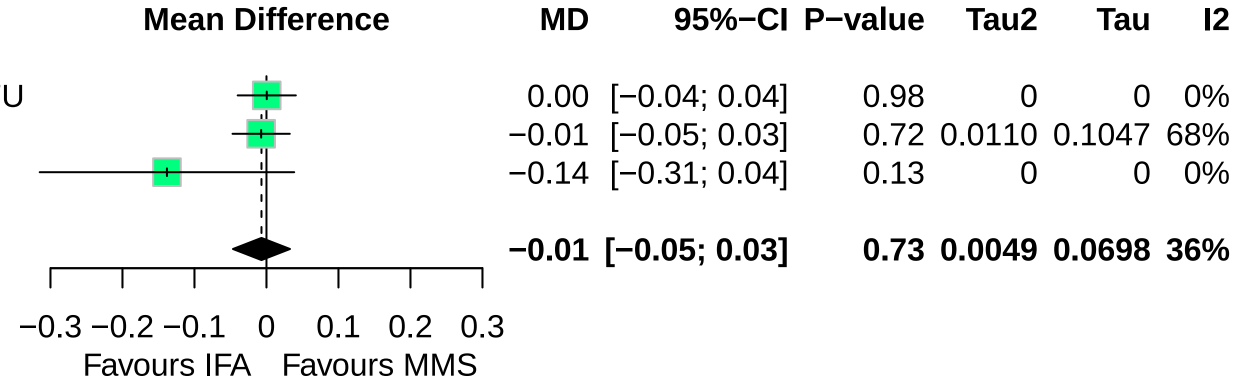

Leave−One−Out Sensitivity Analysis
MUACZ at 24 Months, Random

Study	MD	95%−CI	P−value	Tau2	Tau	I2
Omitting Ashorn 2010 complete FU	0.00	[−0.04; 0.04]	0.98	0	0	0%
Omitting Roberfroid 2008	−0.06	[−0.23; 0.11]	0.46	0.0110	0.1047	68%
Omitting West 2014	−0.14	[−0.31; 0.04]	0.13	0	0	0%
**Random effects model**	**−0.04**	**[−0.16; 0.08]**	**0.50**	**0.0049**	**0.0698**	**36%**

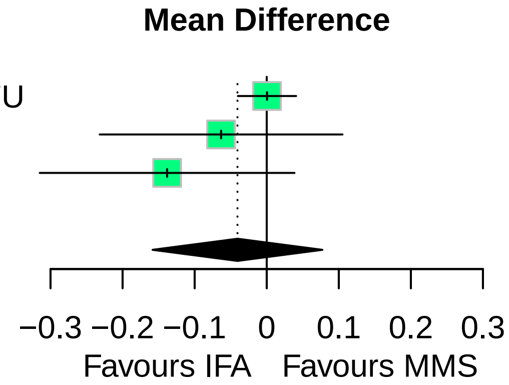

Leave−One−Out Sensitivity Analysis
Stunting at Birth, Fixed

Study	Risk Ratio	RR	95%−CI	P−value	Tau2	Tau	I2
Omitting Adu−Afarwuah 2015		0.91	[0.88; 0.95]	< 0.01	0	0	0%
Omitting Ashorn 2010		0.91	[0.88; 0.95]	< 0.01	0	0	0%
Omitting Fawzi 2007		0.90	[0.87; 0.94]	< 0.01	0	0	0%
Omitting Osrin 2005		0.91	[0.88; 0.95]	< 0.01	0.0001	0.0121	3%
Omitting Ramakrishnan 2003		0.91	[0.88; 0.95]	< 0.01	0.0001	0.0101	2%
Omitting Roberfroid 2008		0.91	[0.88; 0.95]	< 0.01	0.0001	0.0122	3%
Omitting Sunawang 2009		0.91	[0.87; 0.94]	< 0.01	0	0	0%
Omitting Tofail 2008		0.90	[0.87; 0.94]	< 0.01	0	0	0%
Omitting West 2014		0.95	[0.88; 1.01]	0.10	0	0	0%
**Fixed Effect Model**		**0.91**	**[0.88; 0.95]**	**< 0.01**	**0**	**0**	**0%**

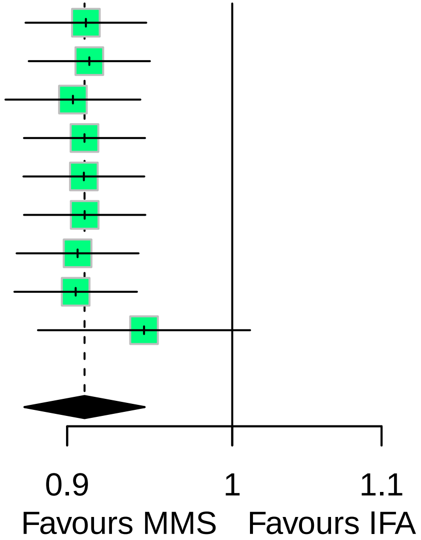

Leave−One−Out Sensitivity Analysis

Stunting at Birth, Random

Study	RR	95%−CI	P−value	Tau2	Tau	I2
Omitting Adu−Afarwuah 2015	0.91	[0.88; 0.95]	< 0.01	0	0	0%
Omitting Ashorn 2010	0.91	[0.88; 0.95]	< 0.01	0	0	0%
Omitting Fawzi 2007	0.90	[0.87; 0.94]	< 0.01	0	0	0%
Omitting Osrin 2005	0.91	[0.87; 0.95]	< 0.01	0.0001	0.0121	3%
Omitting Ramakrishnan 2003	0.91	[0.87; 0.95]	< 0.01	0.0001	0.0101	2%
Omitting Roberfroid 2008	0.91	[0.87; 0.95]	< 0.01	0.0001	0.0122	3%
Omitting Sunawang 2009	0.91	[0.87; 0.94]	< 0.01	0	0	0%
Omitting Tofail 2008	0.90	[0.87; 0.94]	< 0.01	0	0	0%
Omitting West 2014	0.95	[0.88; 1.01]	0.10	0	0	0%
**Random effects model**	**0.91**	**[0.88; 0.95]**	**< 0.01**	**0**	**0**	**0%**

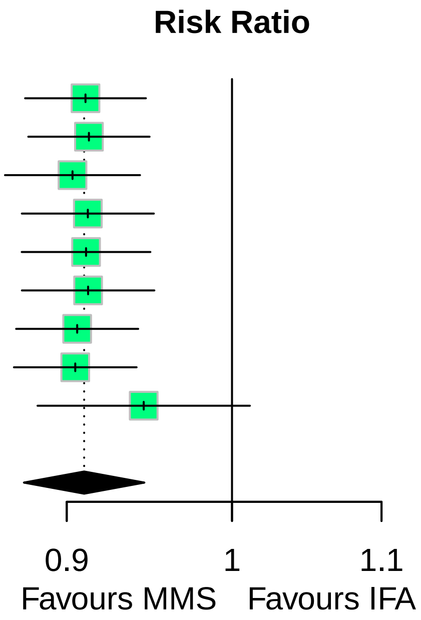

Leave−One−Out Sensitivity Analysis
Stunting at 3 Months, Fixed

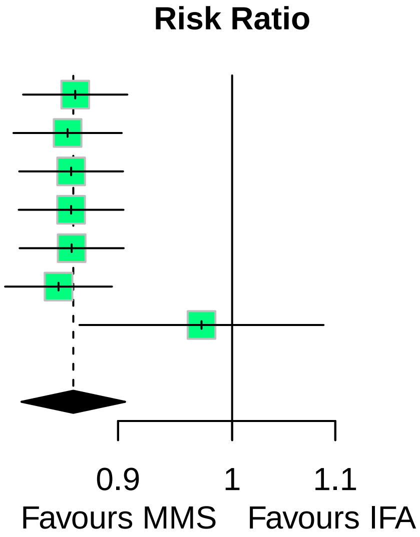

Study	RR	95%−CI	P−value	Tau2	Tau	I2
Omitting Adu−Afarwuah 2015	0.87	[0.82; 0.91]	< 0.01	0.0094	0.0971	49%
Omitting Fawzi 2007	0.86	[0.82; 0.90]	< 0.01	0.0155	0.1246	48%
Omitting Moore 2009	0.86	[0.82; 0.90]	< 0.01	0.0049	0.0700	34%
Omitting Roberfroid 2008	0.86	[0.82; 0.90]	< 0.01	0.0103	0.1013	49%
Omitting Sunawang 2009	0.86	[0.82; 0.90]	< 0.01	0.0090	0.0950	48%
Omitting Tofail 2008	0.85	[0.81; 0.89]	< 0.01	0.0004	0.0203	3%
Omitting West 2014	0.97	[0.87; 1.09]	0.62	0.0002	0.0151	1%
**Fixed Effect Model**	**0.86**	**[0.82; 0.91]**	**< 0.01**	**0.0079**	**0.0891**	**41%**

Leave−One−Out Sensitivity Analysis

Stunting at 3 Months, Random

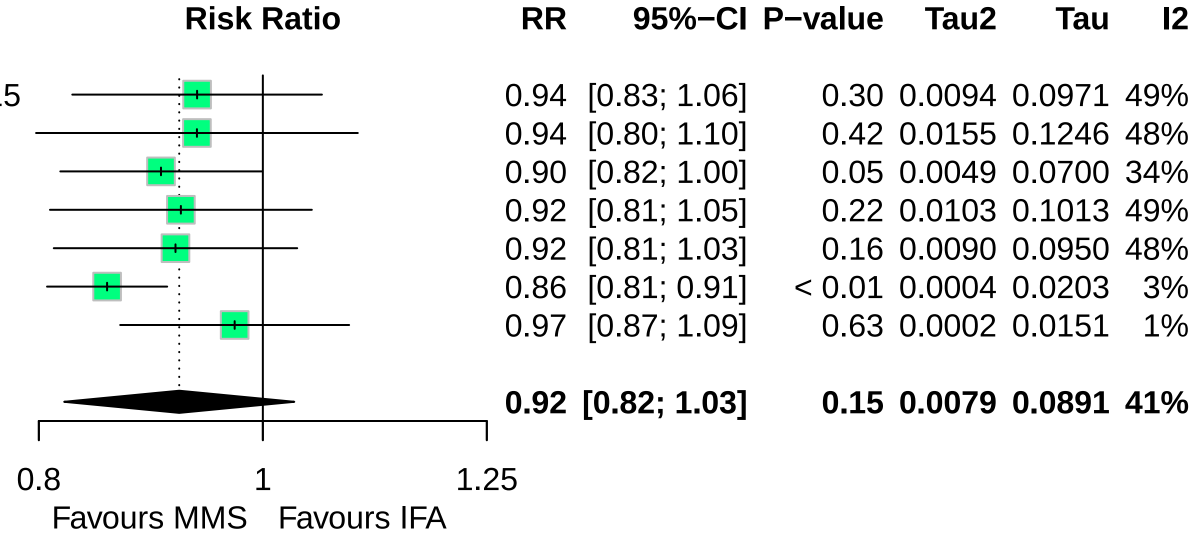

Study	RR	95%−CI	P−value	Tau2	Tau	I2
Omitting Adu−Afarwuah 2015	0.94	[0.83; 1.06]	0.30	0.0094	0.0971	49%
Omitting Fawzi 2007	0.94	[0.80; 1.10]	0.42	0.0155	0.1246	48%
Omitting Moore 2009	0.90	[0.82; 1.00]	0.05	0.0049	0.0700	34%
Omitting Roberfroid 2008	0.92	[0.81; 1.05]	0.22	0.0103	0.1013	49%
Omitting Sunawang 2009	0.92	[0.81; 1.03]	0.16	0.0090	0.0950	48%
Omitting Tofail 2008	0.86	[0.81; 0.91]	< 0.01	0.0004	0.0203	3%
Omitting West 2014	0.97	[0.87; 1.09]	0.63	0.0002	0.0151	1%
**Random effects model**	**0.92**	**[0.82; 1.03]**	**0.15**	**0.0079**	**0.0891**	**41%**

Leave−One−Out Sensitivity Analysis
Stunting at 6 Months, Fixed

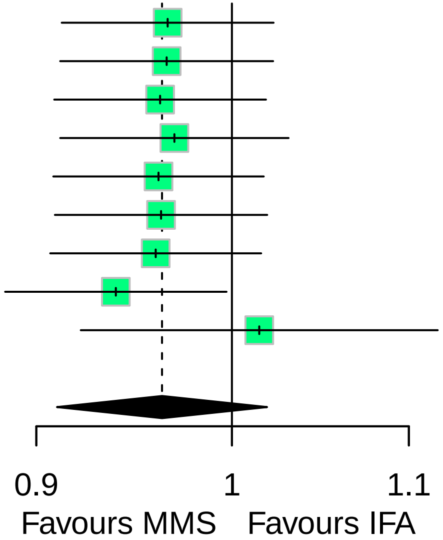

Study	RR	95%−CI	P−value	Tau2	Tau	I2
Omitting Adu−Afarwuah 2015	0.97	[0.91; 1.02]	0.23	0.0049	0.0697	30%
Omitting Ashorn 2010 complete FU	0.97	[0.91; 1.02]	0.23	0.0056	0.0751	33%
Omitting Ashorn 2010 simplified FU	0.96	[0.91; 1.02]	0.18	0.0058	0.0760	34%
Omitting Fawzi 2007	0.97	[0.91; 1.03]	0.32	0.0085	0.0923	32%
Omitting Moore 2009	0.96	[0.91; 1.02]	0.17	0.0039	0.0626	26%
Omitting Roberfroid 2008	0.96	[0.91; 1.02]	0.19	0.0060	0.0772	34%
Omitting Sunawang 2009	0.96	[0.91; 1.02]	0.16	0.0032	0.0564	22%
Omitting Tofail 2008	0.94	[0.89; 1.00]	0.04	0	0	0%
Omitting West 2014	1.01	[0.92; 1.12]	0.76	0.0065	0.0804	21%
**Fixed Effect Model**	**0.96**	**[0.91; 1.02]**	**0.19**	**0.0041**	**0.0640**	**25%**

Leave−One−Out Sensitivity Analysis
Stunting at 6 Months, Random

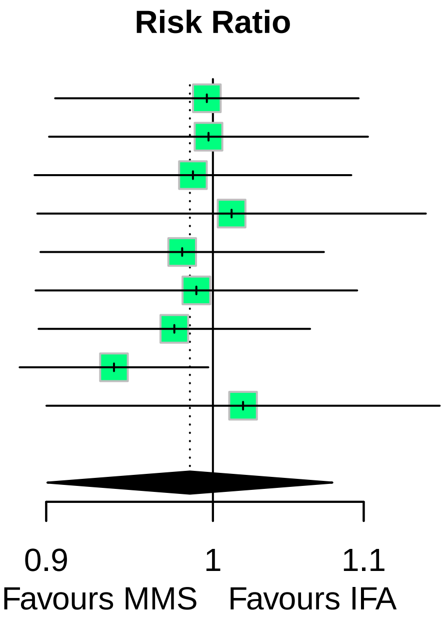

Study	RR	95%−CI	P−value	Tau2	Tau	I2
Omitting Adu−Afarwuah 2015	1.00	[0.91; 1.10]	0.94	0.0049	0.0697	30%
Omitting Ashorn 2010 complete FU	1.00	[0.90; 1.10]	0.96	0.0056	0.0751	33%
Omitting Ashorn 2010 simplified FU	0.99	[0.89; 1.09]	0.80	0.0058	0.0760	34%
Omitting Fawzi 2007	1.01	[0.89; 1.14]	0.85	0.0085	0.0923	32%
Omitting Moore 2009	0.98	[0.90; 1.07]	0.67	0.0039	0.0626	26%
Omitting Roberfroid 2008	0.99	[0.89; 1.10]	0.84	0.0060	0.0772	34%
Omitting Sunawang 2009	0.98	[0.90; 1.06]	0.58	0.0032	0.0564	22%
Omitting Tofail 2008	0.94	[0.89; 1.00]	0.04	0	0	0%
Omitting West 2014	1.02	[0.90; 1.15]	0.76	0.0065	0.0804	21%
**Random effects model**	**0.99**	**[0.90; 1.08]**	**0.75**	**0.0041**	**0.0640**	**25%**

Leave−One−Out Sensitivity Analysis
Stunting at 12 Months, Fixed

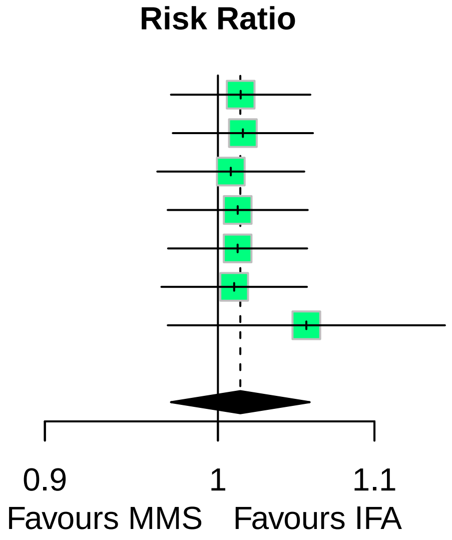

Study	RR	95%−CI	P−value	Tau2	Tau	I2
Omitting Adu−Afarwuah 2015	1.01	[0.97; 1.06]	0.52	0	0	0%
Omitting Ashorn 2010 complete FU	1.02	[0.97; 1.06]	0.49	0	0	0%
Omitting Fawzi 2007	1.01	[0.96; 1.05]	0.73	0	0	0%
Omitting Roberfroid 2008	1.01	[0.97; 1.06]	0.58	0	0	0%
Omitting Sunawang 2009	1.01	[0.97; 1.06]	0.58	0	0	0%
Omitting Tofail 2008	1.01	[0.97; 1.06]	0.66	0	0	0%
Omitting West 2014	1.06	[0.97; 1.15]	0.21	0	0	0%
**Fixed Effect Model**	**1.01**	**[0.97; 1.06]**	**0.53**	**0**	**0**	**0%**

Leave−One−Out Sensitivity Analysis
Stunting at 12 Months, Random

Study	RR	95%−CI	P−value	Tau2	Tau	I2
Omitting Adu−Afarwuah 2015	1.01	[0.97; 1.06]	0.52	0	0	0%
Omitting Ashorn 2010 complete FU	1.02	[0.97; 1.06]	0.49	0	0	0%
Omitting Fawzi 2007	1.01	[0.96; 1.05]	0.73	0	0	0%
Omitting Roberfroid 2008	1.01	[0.97; 1.06]	0.58	0	0	0%
Omitting Sunawang 2009	1.01	[0.97; 1.06]	0.58	0	0	0%
Omitting Tofail 2008	1.01	[0.97; 1.06]	0.66	0	0	0%
Omitting West 2014	1.06	[0.97; 1.15]	0.21	0	0	0%
**Random effects model**	**1.01**	**[0.97; 1.06]**	**0.53**	**0**	**0**	**0%**

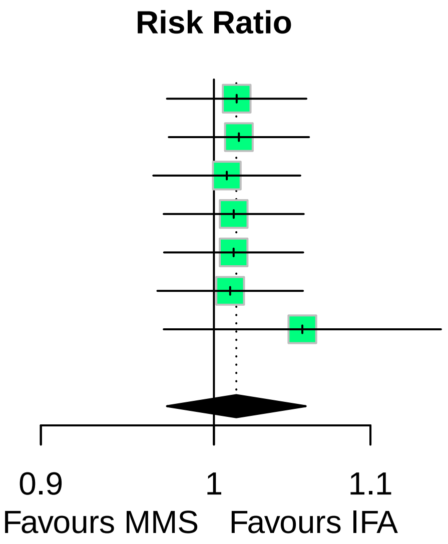

Leave−One−Out Sensitivity Analysis
Stunting at 18 Months, Fixed

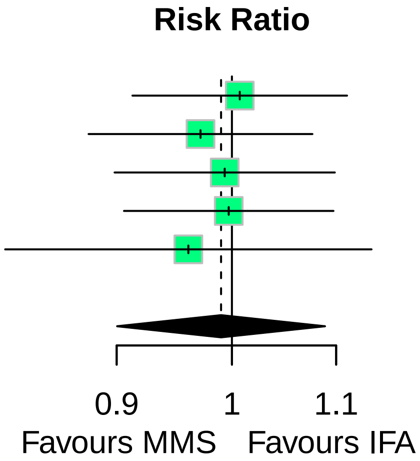

Study	RR	95%−CI	P−value	Tau2	Tau	I2
Omitting Adu−Afarwuah 2015	1.01	[0.91; 1.11]	0.89	0	0	0%
Omitting Ashorn 2010 complete FU	0.97	[0.88; 1.08]	0.58	0.0009	0.0303	4%
Omitting Ashorn 2010 simplified FU	0.99	[0.90; 1.10]	0.90	0.0069	0.0833	26%
Omitting Fawzi 2007	1.00	[0.91; 1.10]	0.95	0	0	0%
Omitting Tofail 2008	0.96	[0.81; 1.14]	0.64	0.0097	0.0987	23%
**Fixed Effect Model**	**0.99**	**[0.90; 1.09]**	**0.84**	**0.0003**	**0.0180**	**2%**

Leave−One−Out Sensitivity Analysis
Stunting at 18 Months, Random

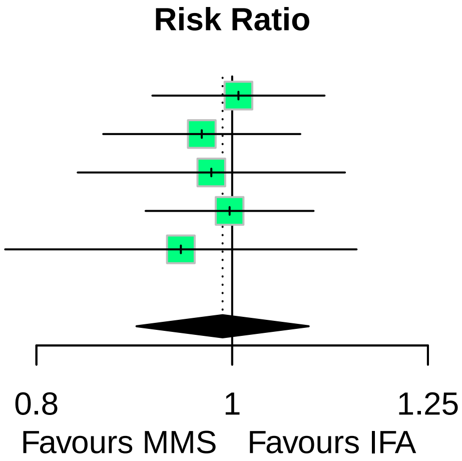

Study	RR	95%−CI	P−value	Tau2	Tau	I2
Omitting Adu−Afarwuah 2015	1.01	[0.91; 1.11]	0.89	0	0	0%
Omitting Ashorn 2010 complete FU	0.97	[0.86; 1.08]	0.55	0.0009	0.0303	4%
Omitting Ashorn 2010 simplified FU	0.98	[0.84; 1.14]	0.76	0.0069	0.0833	26%
Omitting Fawzi 2007	1.00	[0.91; 1.10]	0.95	0	0	0%
Omitting Tofail 2008	0.94	[0.77; 1.15]	0.57	0.0097	0.0987	23%
**Random effects model**	**0.99**	**[0.90; 1.09]**	**0.83**	**0.0003**	**0.0180**	**2%**

Leave−One−Out Sensitivity Analysis
Stunting at 24 Months, Fixed

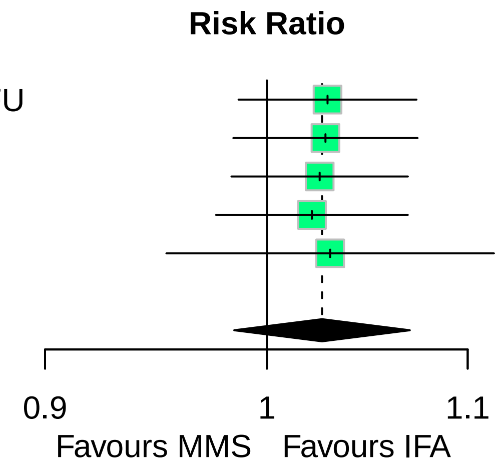

Study	RR	95%−CI	P−value	Tau2	Tau	I2
Omitting Ashorn 2010 complete FU	1.03	[0.99; 1.07]	0.18	0	0	0%
Omitting Bliznashka 2021	1.03	[0.98; 1.07]	0.21	0	0	0%
Omitting Roberfroid 2008	1.03	[0.98; 1.07]	0.24	0	0	0%
Omitting Tofail 2008	1.02	[0.98; 1.07]	0.36	0	0	0%
Omitting West 2014	1.03	[0.95; 1.11]	0.45	0	0	0%
**Fixed Effect Model**	**1.03**	**[0.98; 1.07]**	**0.22**	**0**	**0**	**0%**

Leave−One−Out Sensitivity Analysis
Stunting at 24 Months, Random

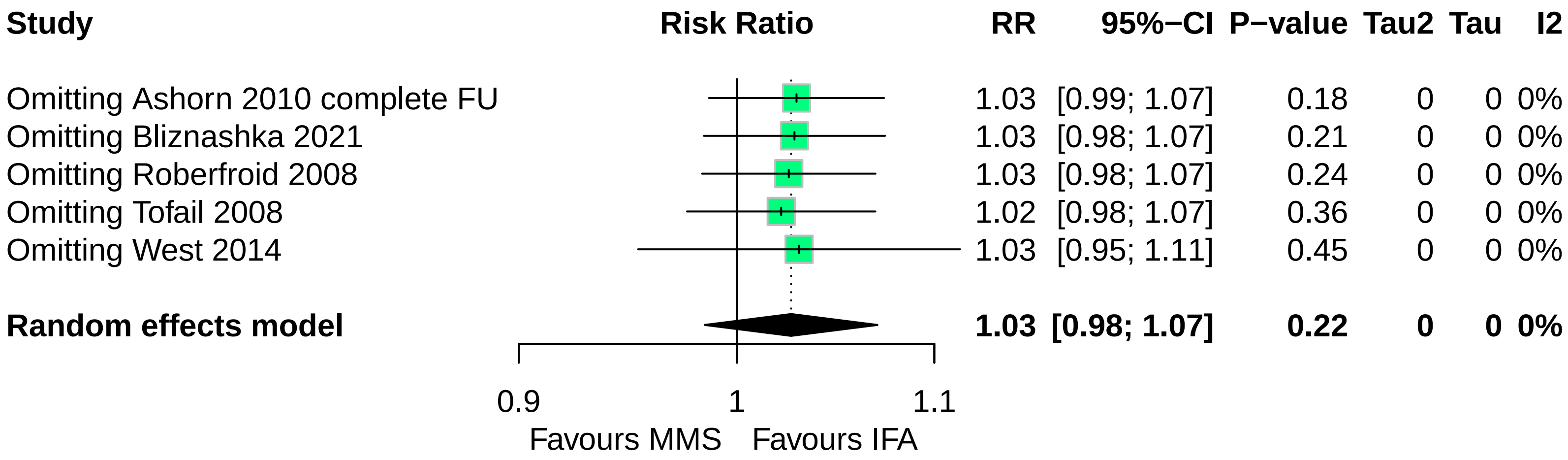

Leave−One−Out Sensitivity Analysis
Underweight at Birth, Fixed

Study	RR	95%−CI	P−value	Tau2	Tau	I2
Omitting Adu−Afarwuah 2015	0.87	[0.84; 0.91]	< 0.01	0	0	0%
Omitting Ashorn 2010	0.87	[0.84; 0.91]	< 0.01	0	0	0%
Omitting Fawzi 2007	0.87	[0.84; 0.91]	< 0.01	0	0	0%
Omitting Moore 2009	0.87	[0.84; 0.91]	< 0.01	0	0	0%
Omitting Osrin 2005	0.88	[0.84; 0.91]	< 0.01	0	0	0%
Omitting Roberfroid 2008	0.87	[0.84; 0.91]	< 0.01	0	0	0%
Omitting Sunawang 2009	0.87	[0.84; 0.91]	< 0.01	0	0	0%
Omitting Tofail 2008	0.87	[0.83; 0.91]	< 0.01	0	0	0%
Omitting West 2014	0.88	[0.80; 0.96]	< 0.01	0	0	0%
**Fixed Effect Model**	**0.87**	**[0.84; 0.91]**	**< 0.01**	**0**	**0**	**0%**

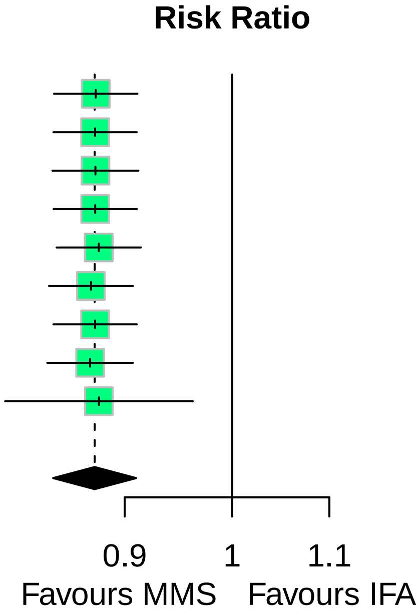

Leave−One−Out Sensitivity Analysis

Underweight at Birth, Random

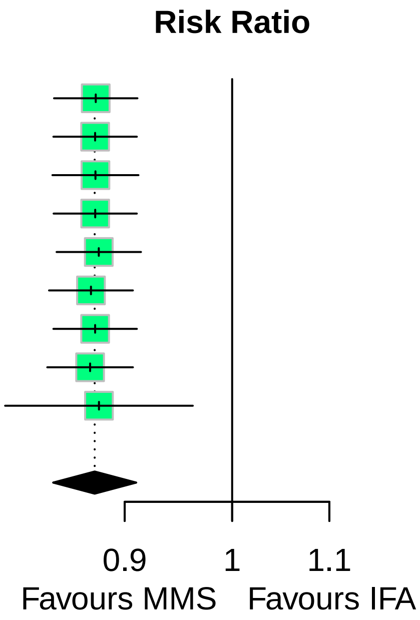

Study	RR	95%−CI	P−value	Tau2	Tau	I2
Omitting Adu−Afarwuah 2015	0.87	[0.84; 0.91]	< 0.01	0	0	0%
Omitting Ashorn 2010	0.87	[0.84; 0.91]	< 0.01	0	0	0%
Omitting Fawzi 2007	0.87	[0.84; 0.91]	< 0.01	0	0	0%
Omitting Moore 2009	0.87	[0.84; 0.91]	< 0.01	0	0	0%
Omitting Osrin 2005	0.88	[0.84; 0.91]	< 0.01	0	0	0%
Omitting Roberfroid 2008	0.87	[0.84; 0.91]	< 0.01	0	0	0%
Omitting Sunawang 2009	0.87	[0.84; 0.91]	< 0.01	0	0	0%
Omitting Tofail 2008	0.87	[0.83; 0.91]	< 0.01	0	0	0%
Omitting West 2014	0.88	[0.80; 0.96]	< 0.01	0	0	0%
**Random effects model**	**0.87**	**[0.84; 0.91]**	**< 0.01**	**0**	**0**	**0%**

Leave−One−Out Sensitivity Analysis

Underweight at 3 Months, Fixed

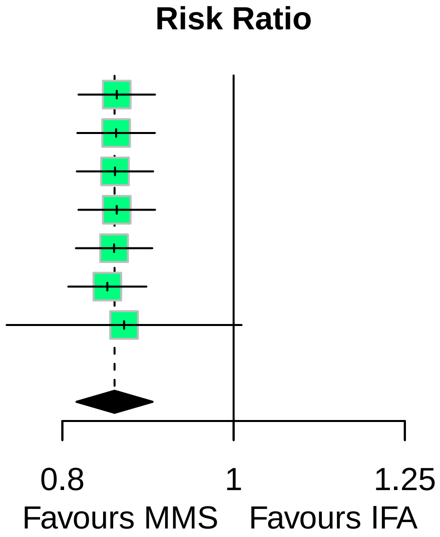

Study	RR	95%−CI	P−value	Tau2	Tau	I2
Omitting Adu−Afarwuah 2015	0.86	[0.82; 0.90]	< 0.01	0	0	0%
Omitting Fawzi 2007	0.86	[0.82; 0.90]	< 0.01	0.0065	0.0807	22%
Omitting Moore 2009	0.86	[0.82; 0.90]	< 0.01	0.0048	0.0694	22%
Omitting Roberfroid 2008	0.86	[0.82; 0.90]	< 0.01	0.0010	0.0322	5%
Omitting Sunawang 2009	0.86	[0.81; 0.90]	< 0.01	0.0049	0.0699	22%
Omitting Tofail 2008	0.85	[0.81; 0.89]	< 0.01	0	0	0%
Omitting West 2014	0.87	[0.74; 1.01]	0.07	0.0137	0.1172	23%
**Fixed Effect Model**	**0.86**	**[0.81; 0.90]**	**< 0.01**	**0.0018**	**0.0427**	**8%**

Leave−One−Out Sensitivity Analysis
Underweight at 3 Months, Random

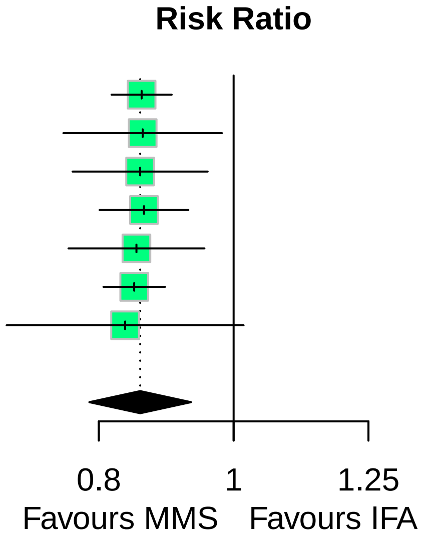

Study	RR	95%−CI	P−value	Tau2	Tau	I2
Omitting Adu−Afarwuah 2015	0.86	[0.82; 0.90]	< 0.01	0	0	0%
Omitting Fawzi 2007	0.86	[0.75; 0.98]	0.02	0.0065	0.0807	22%
Omitting Moore 2009	0.86	[0.77; 0.96]	< 0.01	0.0048	0.0694	22%
Omitting Roberfroid 2008	0.86	[0.80; 0.93]	< 0.01	0.0010	0.0322	5%
Omitting Sunawang 2009	0.85	[0.76; 0.95]	< 0.01	0.0049	0.0699	22%
Omitting Tofail 2008	0.85	[0.81; 0.89]	< 0.01	0	0	0%
Omitting West 2014	0.84	[0.69; 1.02]	0.07	0.0137	0.1172	23%
**Random effects model**	**0.86**	**[0.79; 0.93]**	**< 0.01**	**0.0018**	**0.0427**	**8%**

Leave−One−Out Sensitivity Analysis

Underweight at 6 Months, Fixed

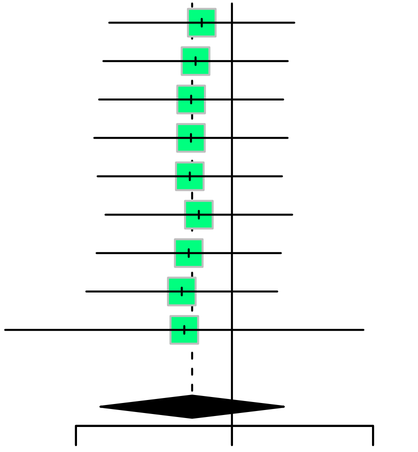

Study	RR	95%−CI	P−value	Tau2	Tau	I2
Omitting Adu−Afarwuah 2015	0.98	[0.92; 1.04]	0.52	0	0	0%
Omitting Ashorn 2010 complete FU	0.98	[0.92; 1.04]	0.44	0	0	0%
Omitting Ashorn 2010 simplified FU	0.97	[0.91; 1.04]	0.38	0	0	0%
Omitting Fawzi 2007	0.97	[0.91; 1.04]	0.41	0	0	0%
Omitting Moore 2009	0.97	[0.91; 1.03]	0.37	0	0	0%
Omitting Roberfroid 2008	0.98	[0.92; 1.04]	0.49	0	0	0%
Omitting Sunawang 2009	0.97	[0.91; 1.03]	0.36	0	0	0%
Omitting Tofail 2008	0.97	[0.91; 1.03]	0.30	0	0	0%
Omitting West 2014	0.97	[0.86; 1.09]	0.60	0	0	0%
**Fixed Effect Model**	**0.97**	**[0.91; 1.04]**	**0.40**	**0**	**0**	**0%**

Leave−One−Out Sensitivity Analysis
Underweight at 6 Months, Random

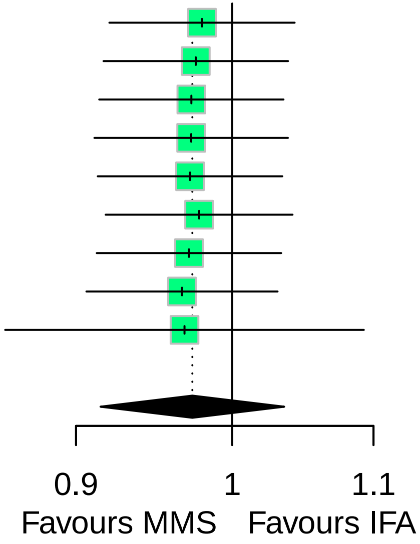

Study	RR	95%−CI	P−value	Tau2	Tau	I2
Omitting Adu−Afarwuah 2015	0.98	[0.92; 1.04]	0.52	0	0	0%
Omitting Ashorn 2010 complete FU	0.98	[0.92; 1.04]	0.44	0	0	0%
Omitting Ashorn 2010 simplified FU	0.97	[0.91; 1.04]	0.38	0	0	0%
Omitting Fawzi 2007	0.97	[0.91; 1.04]	0.41	0	0	0%
Omitting Moore 2009	0.97	[0.91; 1.03]	0.37	0	0	0%
Omitting Roberfroid 2008	0.98	[0.92; 1.04]	0.49	0	0	0%
Omitting Sunawang 2009	0.97	[0.91; 1.03]	0.36	0	0	0%
Omitting Tofail 2008	0.97	[0.91; 1.03]	0.30	0	0	0%
Omitting West 2014	0.97	[0.86; 1.09]	0.60	0	0	0%
**Random effects model**	**0.97**	**[0.91; 1.04]**	**0.40**	**0**	**0**	**0%**

Leave−One−Out Sensitivity Analysis

Underweight at 12 Months, Fixed

Study	RR	95%−CI	P−value	Tau2	Tau	I2
Omitting Adu−Afarwuah 2015	0.97	[0.93; 1.02]	0.24	0	0	0%
Omitting Ashorn 2010 complete FU	0.97	[0.92; 1.01]	0.13	0.0008	0.0287	9%
Omitting Fawzi 2007	0.97	[0.92; 1.01]	0.14	0.0053	0.0728	30%
Omitting Roberfroid 2008	0.97	[0.92; 1.01]	0.17	0.0047	0.0683	32%
Omitting Sunawang 2009	0.97	[0.92; 1.01]	0.16	0.0040	0.0631	32%
Omitting Tofail 2008	0.97	[0.93; 1.02]	0.27	0.0036	0.0597	22%
Omitting West 2014	0.94	[0.85; 1.05]	0.25	0.0084	0.0914	29%
**Fixed Effect Model**	**0.97**	**[0.92; 1.01]**	**0.16**	**0.0023**	**0.0477**	**19%**

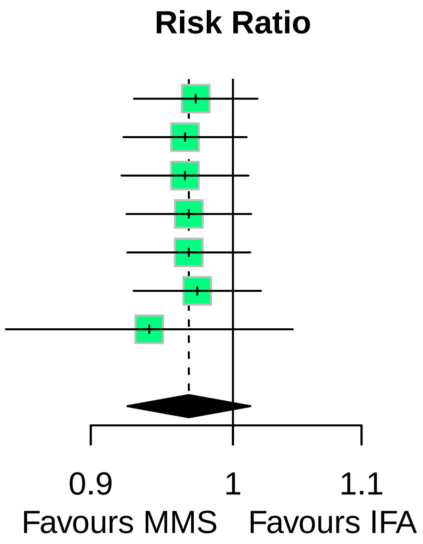

Leave−One−Out Sensitivity Analysis
Underweight at 12 Months, Random

Study	RR	95%−CI	P−value	Tau2	Tau	I2
Omitting Adu−Afarwuah 2015	0.97	[0.93; 1.02]	0.24	0	0	0%
Omitting Ashorn 2010 complete FU	0.96	[0.90; 1.02]	0.18	0.0008	0.0287	9%
Omitting Fawzi 2007	0.94	[0.84; 1.05]	0.28	0.0053	0.0728	30%
Omitting Roberfroid 2008	0.95	[0.86; 1.05]	0.35	0.0047	0.0683	32%
Omitting Sunawang 2009	0.96	[0.87; 1.05]	0.32	0.0040	0.0631	32%
Omitting Tofail 2008	0.97	[0.88; 1.07]	0.55	0.0036	0.0597	22%
Omitting West 2014	0.94	[0.81; 1.08]	0.37	0.0084	0.0914	29%
**Random effects model**	**0.96**	**[0.89; 1.04]**	**0.29**	**0.0023**	**0.0477**	**19%**

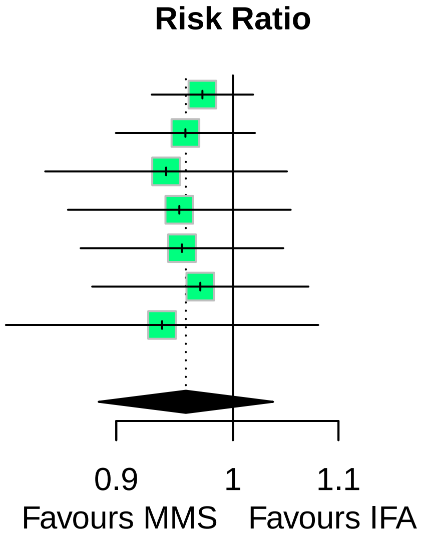

Leave−One−Out Sensitivity Analysis
Underweight at 18 Months, Fixed

Study	RR	95%−CI	P−value	Tau2	Tau	I2
Omitting Adu−Afarwuah 2015	0.93	[0.81; 1.07]	0.33	0	0	0%
Omitting Ashorn 2010 complete FU	0.86	[0.75; 0.99]	0.04	0.0034	0.0584	7%
Omitting Ashorn 2010 simplified FU	0.88	[0.77; 1.02]	0.08	0.0395	0.1987	49%
Omitting Fawzi 2007	0.88	[0.77; 1.01]	0.08	0.0330	0.1818	48%
Omitting Tofail 2008	0.86	[0.66; 1.13]	0.28	0.0769	0.2774	49%
**Fixed Effect Model**	**0.89**	**[0.78; 1.02]**	**0.08**	**0.0213**	**0.1460**	**33%**

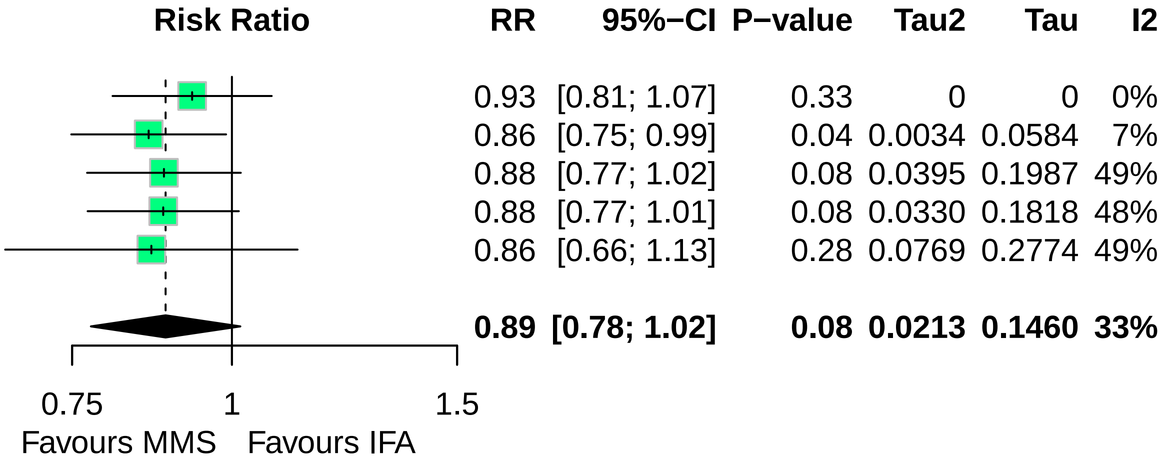

Leave−One−Out Sensitivity Analysis

Underweight at 18 Months, Random

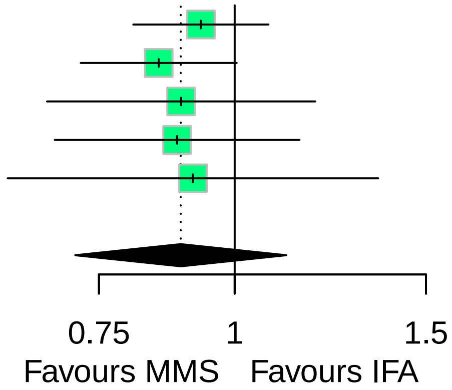

Study	RR	95%−CI	P−value	Tau2	Tau	I2
Omitting Adu−Afarwuah 2015	0.93	[0.81; 1.07]	0.33	0	0	0%
Omitting Ashorn 2010 complete FU	0.85	[0.72; 1.00]	0.06	0.0034	0.0584	7%
Omitting Ashorn 2010 simplified FU	0.89	[0.67; 1.19]	0.43	0.0395	0.1987	49%
Omitting Fawzi 2007	0.89	[0.68; 1.15]	0.36	0.0330	0.1818	48%
Omitting Tofail 2008	0.92	[0.62; 1.36]	0.66	0.0769	0.2774	49%
**Random effects model**	**0.89**	**[0.71; 1.12]**	**0.32**	**0.0213**	**0.1460**	**33%**

Leave−One−Out Sensitivity Analysis
Underweight at 24 Months, Fixed

Study	RR	95%−CI	P−value	Tau2	Tau	I2
Omitting Ashorn 2010 complete FU	1.01	[0.96; 1.05]	0.82	0	0	0%
Omitting Bliznashka 2021	1.00	[0.96; 1.05]	0.84	0	0	0%
Omitting Roberfroid 2008	1.01	[0.96; 1.06]	0.69	0	0	0%
Omitting Tofail 2008	1.01	[0.97; 1.06]	0.57	0	0	0%
Omitting West 2014	0.99	[0.89; 1.11]	0.91	0	0	0%
Omitting	1.01	[0.96; 1.05]	0.74	0	0	0%
Omitting	1.01	[0.96; 1.05]	0.74	0	0	0%
Omitting	1.01	[0.96; 1.05]	0.74	0	0	0%
Omitting	1.01	[0.96; 1.05]	0.74	0	0	0%
Omitting	1.01	[0.96; 1.05]	0.74	0	0	0%
Omitting	1.01	[0.96; 1.05]	0.74	0	0	0%
**Fixed Effect Model**	**1.01**	**[0.96; 1.05]**	**0.74**	**0**	**0**	**0%**

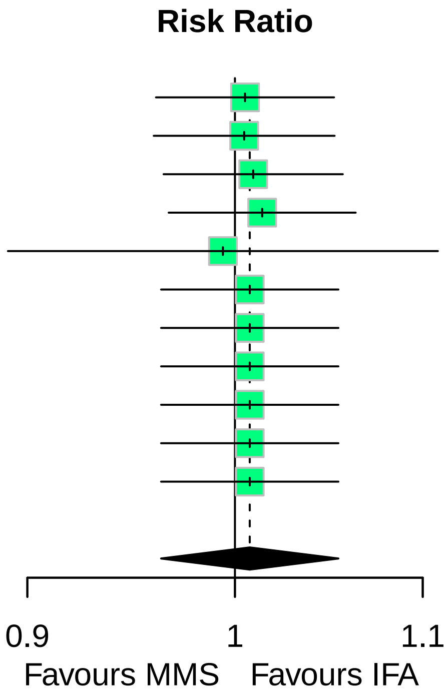

Leave−One−Out Sensitivity Analysis
Underweight at 24 Months, Random

Study	RR	95%−CI	P−value	Tau2	Tau	I2
Omitting Ashorn 2010 complete FU	1.01	[0.96; 1.05]	0.82	0	0	0%
Omitting Bliznashka 2021	1.00	[0.96; 1.05]	0.84	0	0	0%
Omitting Roberfroid 2008	1.01	[0.96; 1.06]	0.69	0	0	0%
Omitting Tofail 2008	1.01	[0.97; 1.06]	0.57	0	0	0%
Omitting West 2014	0.99	[0.89; 1.11]	0.91	0	0	0%
Omitting	1.01	[0.96; 1.05]	0.74	0	0	0%
Omitting	1.01	[0.96; 1.05]	0.74	0	0	0%
Omitting	1.01	[0.96; 1.05]	0.74	0	0	0%
Omitting	1.01	[0.96; 1.05]	0.74	0	0	0%
Omitting	1.01	[0.96; 1.05]	0.74	0	0	0%
Omitting	1.01	[0.96; 1.05]	0.74	0	0	0%
**Random effects model**	**1.01**	**[0.96; 1.05]**	**0.74**	**0**	**0**	**0%**

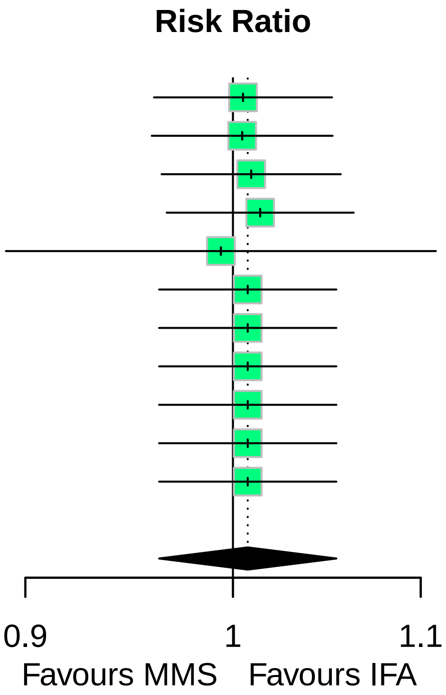

Leave−One−Out Sensitivity Analysis

Wasting at Birth, Fixed

Study	RR	95%−CI	P−value	Tau2	Tau	I2
Omitting Adu−Afarwuah 2015	0.90	[0.85; 0.96]	< 0.01	< 0.0001	0.0055	0%
Omitting Ashorn 2010	0.90	[0.85; 0.96]	< 0.01	< 0.0001	0.0049	0%
Omitting Fawzi 2007	0.91	[0.84; 0.98]	0.02	0	0	0%
Omitting Osrin 2005	0.92	[0.86; 0.99]	0.02	0	0	0%
Omitting Roberfroid 2008	0.89	[0.84; 0.95]	< 0.01	0	0	0%
Omitting Sunawang 2009	0.90	[0.85; 0.96]	< 0.01	0	0	0%
Omitting Tofail 2008	0.90	[0.85; 0.97]	< 0.01	0	0	0%
Omitting West 2014	0.89	[0.82; 0.96]	< 0.01	0	0	0%
**Fixed Effect Model**	**0.90**	**[0.85; 0.96]**	**< 0.01**	**0**	**0**	**0%**

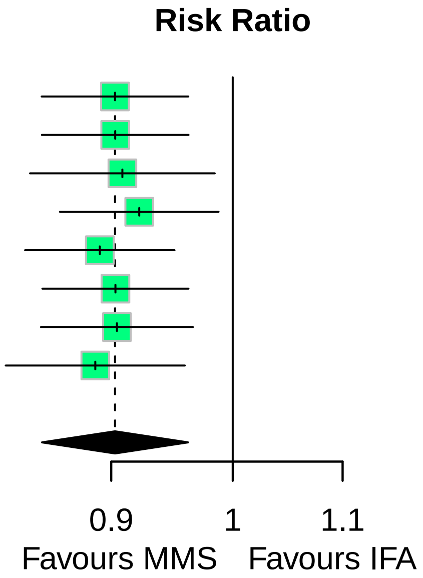

Leave−One−Out Sensitivity Analysis

Wasting at Birth, Random

Study	Risk Ratio	RR	95%−CI	P−value	Tau2	Tau	I2
Omitting Adu−Afarwuah 2015		0.90	[0.85; 0.96]	< 0.01	< 0.0001	0.0055	0%
Omitting Ashorn 2010		0.90	[0.85; 0.96]	< 0.01	< 0.0001	0.0049	0%
Omitting Fawzi 2007		0.91	[0.84; 0.98]	0.02	0	0	0%
Omitting Osrin 2005		0.92	[0.86; 0.99]	0.02	0	0	0%
Omitting Roberfroid 2008		0.89	[0.84; 0.95]	< 0.01	0	0	0%
Omitting Sunawang 2009		0.90	[0.85; 0.96]	< 0.01	0	0	0%
Omitting Tofail 2008		0.90	[0.85; 0.97]	< 0.01	0	0	0%
Omitting West 2014		0.89	[0.82; 0.96]	< 0.01	0	0	0%
**Random effects model**		**0.90**	**[0.85; 0.96]**	**< 0.01**	**0**	**0**	**0%**

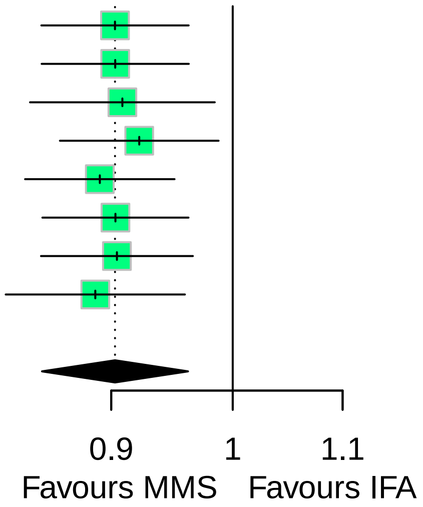

Leave−One−Out Sensitivity Analysis
Wasting at 3 Months, Fixed

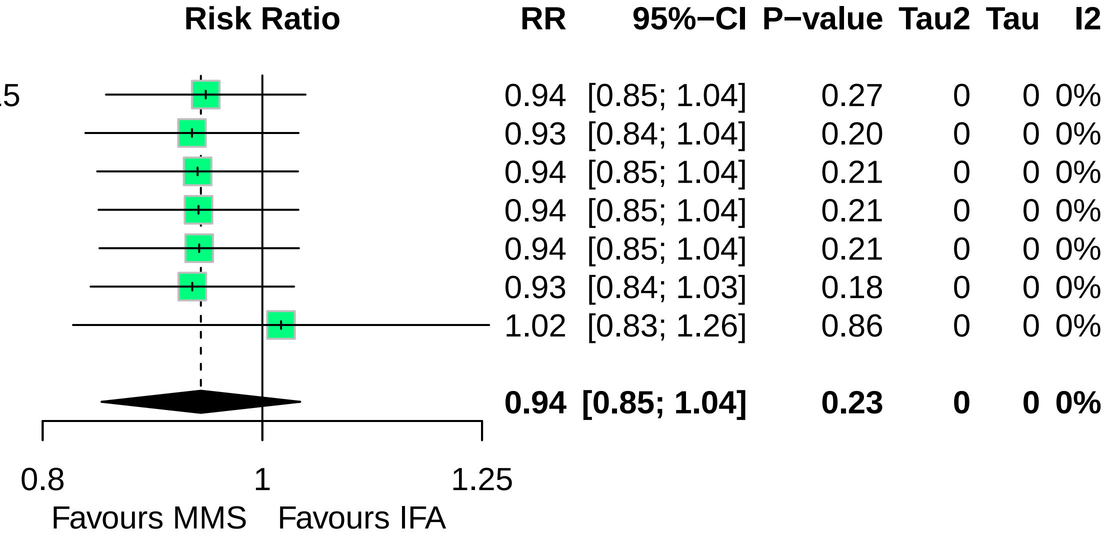

Study	RR	95%−CI	P−value	Tau2	Tau	I2
Omitting Adu−Afarwuah 2015	0.94	[0.85; 1.04]	0.27	0	0	0%
Omitting Fawzi 2007	0.93	[0.84; 1.04]	0.20	0	0	0%
Omitting Moore 2009	0.94	[0.85; 1.04]	0.21	0	0	0%
Omitting Roberfroid 2008	0.94	[0.85; 1.04]	0.21	0	0	0%
Omitting Sunawang 2009	0.94	[0.85; 1.04]	0.21	0	0	0%
Omitting Tofail 2008	0.93	[0.84; 1.03]	0.18	0	0	0%
Omitting West 2014	1.02	[0.83; 1.26]	0.86	0	0	0%
**Fixed Effect Model**	**0.94**	**[0.85; 1.04]**	**0.23**	**0**	**0**	**0%**

Leave−One−Out Sensitivity Analysis
Wasting at 3 Months, Random

Study	RR	95%−CI	P−value	Tau2	Tau	I2
Omitting Adu−Afarwuah 2015	0.94	[0.85; 1.04]	0.27	0	0	0%
Omitting Fawzi 2007	0.93	[0.84; 1.04]	0.20	0	0	0%
Omitting Moore 2009	0.94	[0.85; 1.04]	0.21	0	0	0%
Omitting Roberfroid 2008	0.94	[0.85; 1.04]	0.21	0	0	0%
Omitting Sunawang 2009	0.94	[0.85; 1.04]	0.21	0	0	0%
Omitting Tofail 2008	0.93	[0.84; 1.03]	0.18	0	0	0%
Omitting West 2014	1.02	[0.83; 1.26]	0.86	0	0	0%
**Random effects model**	**0.94**	**[0.85; 1.04]**	**0.23**	**0**	**0**	**0%**

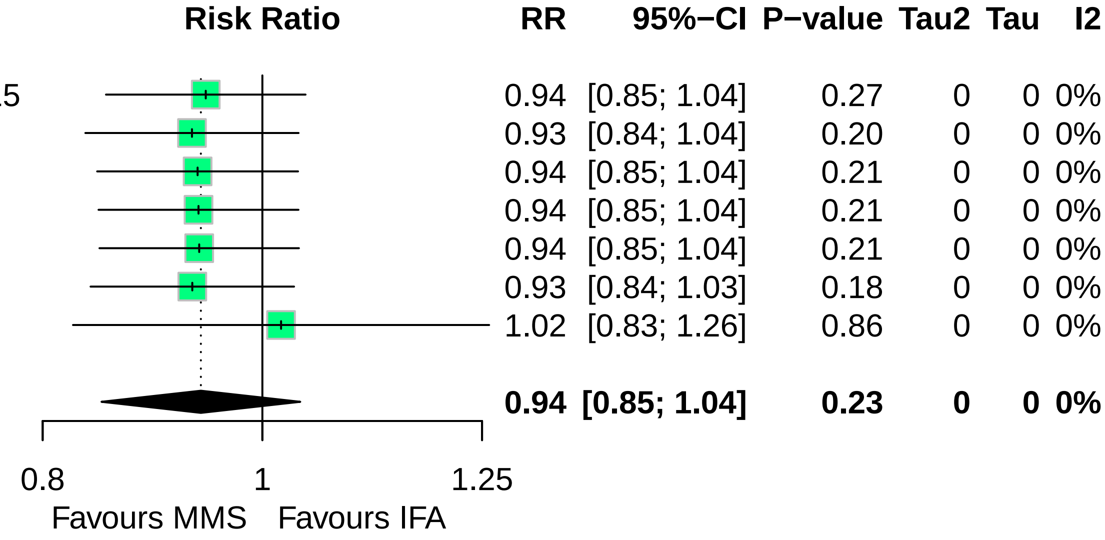

Leave−One−Out Sensitivity Analysis
Wasting at 6 Months, Fixed

Study	RR	95%−CI	P−value	Tau2	Tau	I2
Omitting Adu−Afarwuah 2015	1.05	[0.94; 1.17]	0.43	0	0	0%
Omitting Ashorn 2010 complete FU	1.04	[0.93; 1.16]	0.49	0	0	0%
Omitting Ashorn 2010 simplified FU	1.04	[0.94; 1.16]	0.43	0	0	0%
Omitting Fawzi 2007	1.06	[0.94; 1.19]	0.37	0	0	0%
Omitting Moore 2009	1.04	[0.94; 1.16]	0.45	0	0	0%
Omitting Roberfroid 2008	1.04	[0.93; 1.16]	0.48	0	0	0%
Omitting Sunawang 2009	1.05	[0.94; 1.17]	0.41	0	0	0%
Omitting Tofail 2008	1.06	[0.95; 1.19]	0.28	0	0	0%
Omitting West 2014	1.02	[0.86; 1.22]	0.82	0	0	0%
**Fixed Effect Model**	**1.05**	**[0.94; 1.16]**	**0.42**	**0**	**0**	**0%**

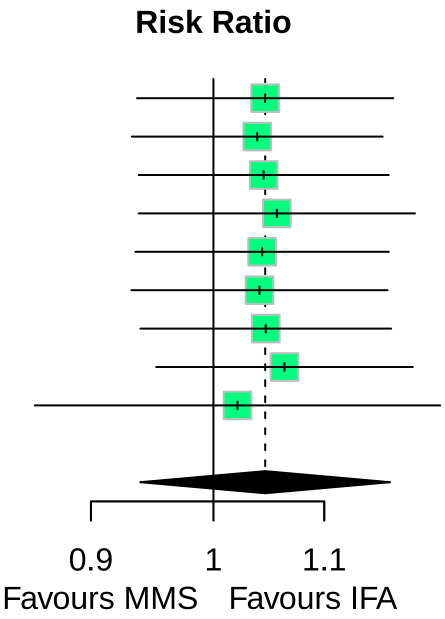

Leave−One−Out Sensitivity Analysis
Wasting at 6 Months, Random

Study	RR	95%−CI	P−value	Tau2	Tau	I2
Omitting Adu−Afarwuah 2015	1.05	[0.94; 1.17]	0.43	0	0	0%
Omitting Ashorn 2010 complete FU	1.04	[0.93; 1.16]	0.49	0	0	0%
Omitting Ashorn 2010 simplified FU	1.04	[0.94; 1.16]	0.43	0	0	0%
Omitting Fawzi 2007	1.06	[0.94; 1.19]	0.37	0	0	0%
Omitting Moore 2009	1.04	[0.94; 1.16]	0.45	0	0	0%
Omitting Roberfroid 2008	1.04	[0.93; 1.16]	0.48	0	0	0%
Omitting Sunawang 2009	1.05	[0.94; 1.17]	0.41	0	0	0%
Omitting Tofail 2008	1.06	[0.95; 1.19]	0.28	0	0	0%
Omitting West 2014	1.02	[0.86; 1.22]	0.82	0	0	0%
**Random effects model**	**1.05**	**[0.94; 1.16]**	**0.42**	**0**	**0**	**0%**

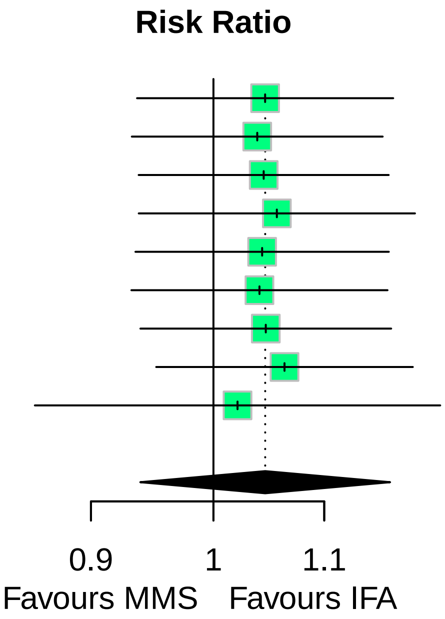

Leave−One−Out Sensitivity Analysis

Wasting at 12 Months, Fixed

Study	RR	95%−CI	P−value	Tau2	Tau	I2
Omitting Adu−Afarwuah 2015	0.98	[0.90; 1.05]	0.54	0	0	0%
Omitting Ashorn 2010 complete FU	0.97	[0.90; 1.04]	0.36	0	0	0%
Omitting Fawzi 2007	0.97	[0.89; 1.05]	0.41	0	0	0%
Omitting Roberfroid 2008	0.97	[0.89; 1.04]	0.39	0	0	0%
Omitting Sunawang 2009	0.97	[0.90; 1.04]	0.38	0	0	0%
Omitting Tofail 2008	0.97	[0.89; 1.05]	0.40	0	0	0%
Omitting West 2014	0.93	[0.80; 1.09]	0.39	0	0	0%
**Fixed Effect Model**	**0.97**	**[0.90; 1.04]**	**0.38**	**0**	**0**	**0%**

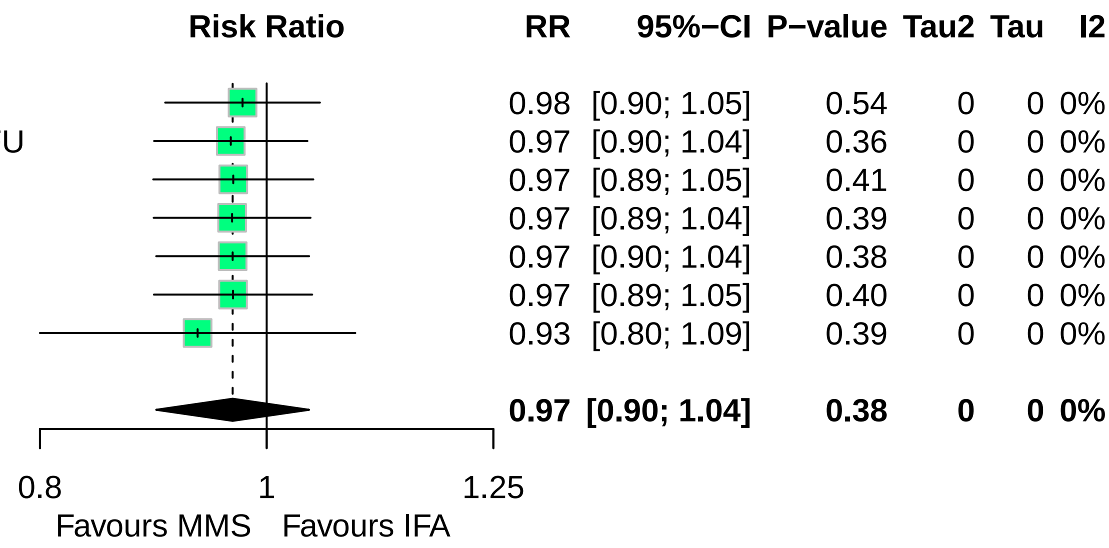

Leave−One−Out Sensitivity Analysis
Wasting at 12 Months, Random

Study	RR	95%−CI	P−value	Tau2	Tau	I2
Omitting Adu−Afarwuah 2015	0.98	[0.90; 1.05]	0.54	0	0	0%
Omitting Ashorn 2010 complete FU	0.97	[0.90; 1.04]	0.36	0	0	0%
Omitting Fawzi 2007	0.97	[0.89; 1.05]	0.41	0	0	0%
Omitting Roberfroid 2008	0.97	[0.89; 1.04]	0.39	0	0	0%
Omitting Sunawang 2009	0.97	[0.90; 1.04]	0.38	0	0	0%
Omitting Tofail 2008	0.97	[0.89; 1.05]	0.40	0	0	0%
Omitting West 2014	0.93	[0.80; 1.09]	0.39	0	0	0%
**Random effects model**	**0.97**	**[0.90; 1.04]**	**0.38**	**0**	**0**	**0%**

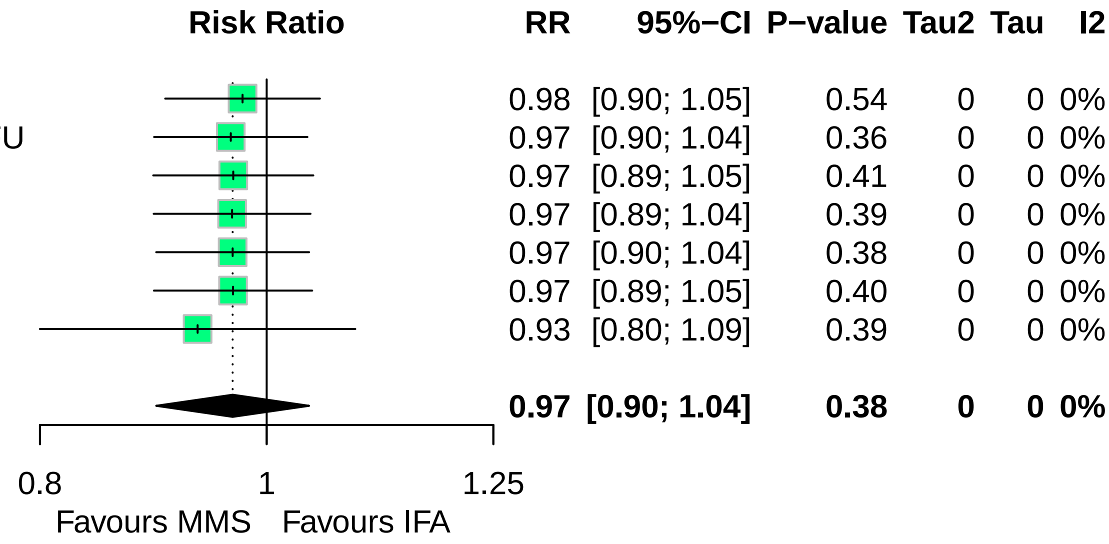

Leave−One−Out Sensitivity Analysis
Wasting at 18 Months, Fixed

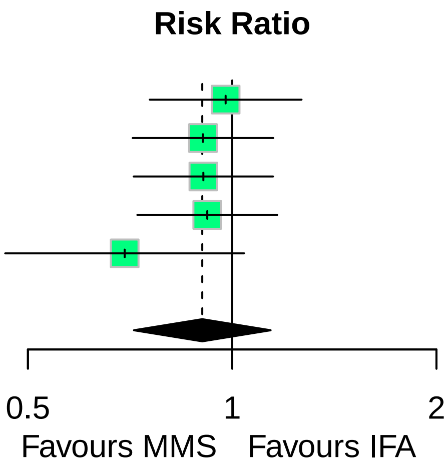

Study	RR	95%−CI	P−value	Tau2	Tau	I2
Omitting Adu−Afarwuah 2015	0.98	[0.76; 1.27]	0.87	0	0	0%
Omitting Ashorn 2010 complete FU	0.91	[0.71; 1.15]	0.42	0	0	0%
Omitting Ashorn 2010 simplified FU	0.91	[0.72; 1.15]	0.42	0	0	0%
Omitting Fawzi 2007	0.92	[0.72; 1.16]	0.48	0	0	0%
Omitting Tofail 2008	0.69	[0.46; 1.04]	0.08	0	0	0%
**Fixed Effect Model**	**0.90**	**[0.72; 1.14]**	**0.39**	**0**	**0**	**0%**

Leave−One−Out Sensitivity Analysis
Wasting at 18 Months, Random

Study	RR	95%−CI	P−value	Tau2	Tau	I2
Omitting Adu−Afarwuah 2015	0.98	[0.76; 1.27]	0.87	0	0	0%
Omitting Ashorn 2010 complete FU	0.91	[0.71; 1.15]	0.42	0	0	0%
Omitting Ashorn 2010 simplified FU	0.91	[0.72; 1.15]	0.42	0	0	0%
Omitting Fawzi 2007	0.92	[0.72; 1.16]	0.48	0	0	0%
Omitting Tofail 2008	0.69	[0.46; 1.04]	0.08	0	0	0%
**Random effects model**	**0.90**	**[0.72; 1.14]**	**0.39**	**0**	**0**	**0%**

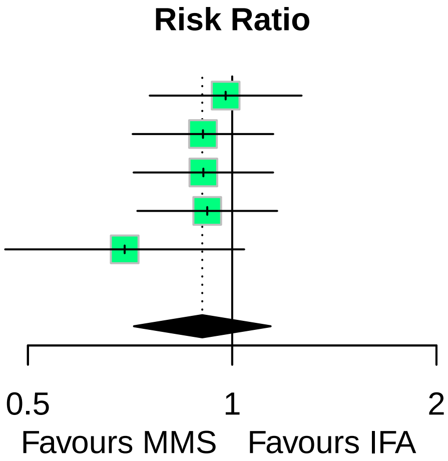

Leave−One−Out Sensitivity Analysis
Wasting at 24 Months, Fixed

Study	RR	95%−CI	P−value	Tau2	Tau	I2
Omitting Ashorn 2010 complete FU	1.00	[0.92; 1.09]	0.94	0	0	0%
Omitting Bliznashka 2021	1.00	[0.92; 1.09]	0.97	0	0	0%
Omitting Roberfroid 2008	1.00	[0.92; 1.09]	0.97	0	0	0%
Omitting Tofail 2008	1.02	[0.93; 1.11]	0.70	0	0	0%
Omitting West 2014	1.01	[0.83; 1.23]	0.91	0	0	0%
**Fixed Effect Model**	**1.01**	**[0.92; 1.09]**	**0.90**	**0**	**0**	**0%**

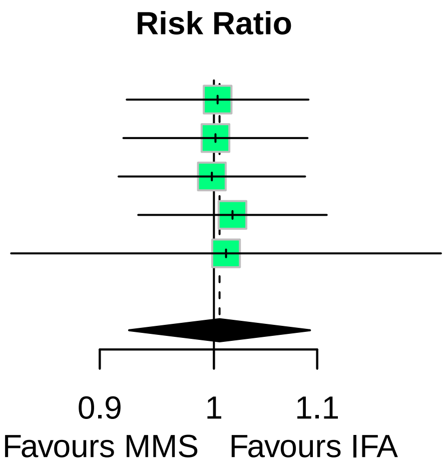

Leave−One−Out Sensitivity Analysis
Wasting at 24 Months, Random

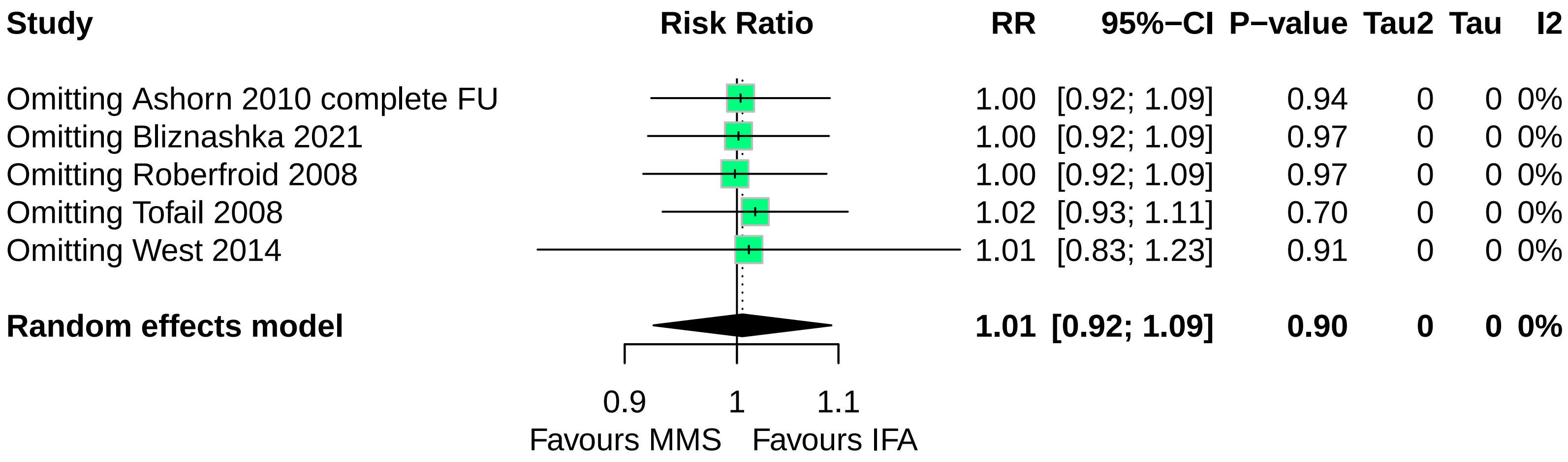

Leave−One−Out Sensitivity Analysis

Small HC at Birth, Fixed

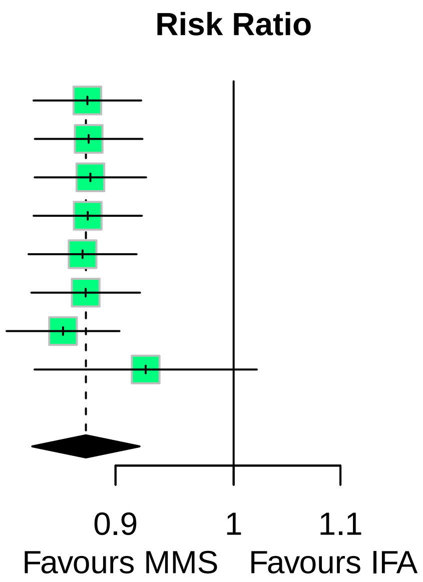

Study	RR	95%−CI	P−value	Tau2	Tau	I2
Omitting Adu−Afarwuah 2015	0.88	[0.84; 0.92]	< 0.01	0.0065	0.0804	41%
Omitting Ashorn 2010	0.88	[0.84; 0.92]	< 0.01	0.0037	0.0606	29%
Omitting Fawzi 2007	0.88	[0.84; 0.92]	< 0.01	0.0091	0.0954	42%
Omitting Osrin 2005	0.88	[0.84; 0.92]	< 0.01	0.0071	0.0843	42%
Omitting Roberfroid 2008	0.87	[0.83; 0.92]	< 0.01	0.0057	0.0752	37%
Omitting Sunawang 2009	0.88	[0.83; 0.92]	< 0.01	0.0078	0.0882	44%
Omitting Tofail 2008	0.86	[0.82; 0.90]	< 0.01	0	0	0%
Omitting West 2014	0.92	[0.84; 1.02]	0.12	0.0115	0.1073	35%
**Fixed Effect Model**	**0.88**	**[0.84; 0.92]**	**< 0.01**	**0.0055**	**0.0742**	**34%**

Leave−One−Out Sensitivity Analysis

Small HC at Birth, Random

Study	RR	95%−CI	P−value	Tau2	Tau	I2
Omitting Adu−Afarwuah 2015	0.90	[0.81; 0.99]	0.03	0.0065	0.0804	41%
Omitting Ashorn 2010	0.90	[0.82; 0.98]	0.02	0.0037	0.0606	29%
Omitting Fawzi 2007	0.90	[0.80; 1.02]	0.10	0.0091	0.0954	42%
Omitting Osrin 2005	0.90	[0.81; 1.00]	0.05	0.0071	0.0843	42%
Omitting Roberfroid 2008	0.88	[0.80; 0.97]	0.01	0.0057	0.0752	37%
Omitting Sunawang 2009	0.89	[0.80; 0.99]	0.04	0.0078	0.0882	44%
Omitting Tofail 2008	0.86	[0.82; 0.90]	< 0.01	0	0	0%
Omitting West 2014	0.90	[0.78; 1.03]	0.14	0.0115	0.1073	35%
**Random effects model**	**0.89**	**[0.81; 0.98]**	**0.02**	**0.0055**	**0.0742**	**34%**

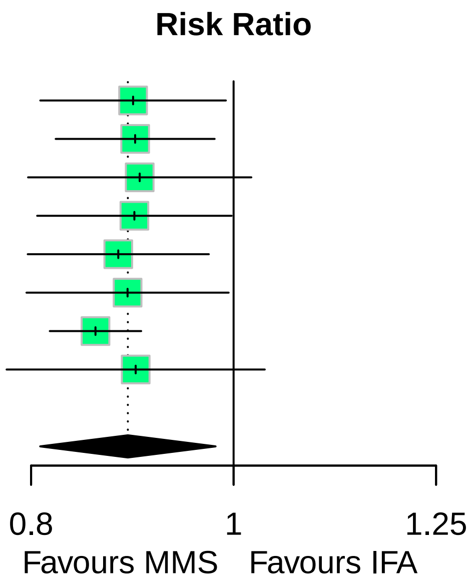

Leave−One−Out Sensitivity Analysis
Small HC at 3 Months, Fixed

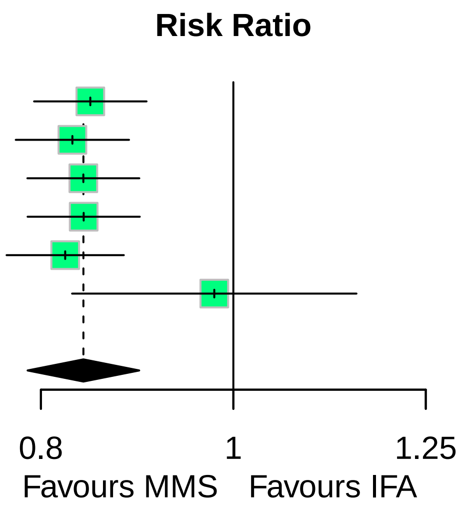

Study	RR	95%−CI	P−value	Tau2	Tau	I2
Omitting Adu−Afarwuah 2015	0.85	[0.79; 0.90]	< 0.01	0.0217	0.1474	55%
Omitting Fawzi 2007	0.83	[0.78; 0.89]	< 0.01	0.0181	0.1347	48%
Omitting Moore 2009	0.84	[0.79; 0.90]	< 0.01	0.0358	0.1891	68%
Omitting Roberfroid 2008	0.84	[0.79; 0.90]	< 0.01	0.0368	0.1919	68%
Omitting Tofail 2008	0.82	[0.77; 0.88]	< 0.01	0.0449	0.2120	54%
Omitting West 2014	0.98	[0.83; 1.15]	0.79	0.0594	0.2437	55%
**Fixed Effect Model**	**0.84**	**[0.79; 0.90]**	**< 0.01**	**0.0306**	**0.1748**	**61%**

Leave−One−Out Sensitivity Analysis
Small HC at 3 Months, Random

Study	RR	95%−CI	P−value	Tau2	Tau	I2
Omitting Adu−Afarwuah 2015	0.94	[0.78; 1.15]	0.56	0.0217	0.1474	55%
Omitting Fawzi 2007	0.84	[0.69; 1.01]	0.06	0.0181	0.1347	48%
Omitting Moore 2009	0.89	[0.71; 1.11]	0.30	0.0358	0.1891	68%
Omitting Roberfroid 2008	0.89	[0.71; 1.12]	0.33	0.0368	0.1919	68%
Omitting Tofail 2008	0.84	[0.64; 1.10]	0.20	0.0449	0.2120	54%
Omitting West 2014	0.91	[0.67; 1.23]	0.53	0.0594	0.2437	55%
**Random effects model**	**0.89**	**[0.72; 1.09]**	**0.25**	**0.0306**	**0.1748**	**61%**

Risk Ratio

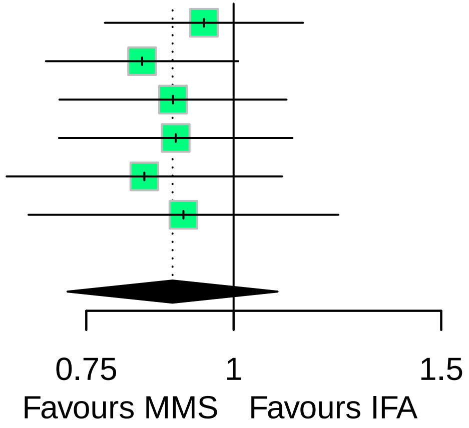

Leave−One−Out Sensitivity Analysis
Small HC at 6 Months, Fixed

Study	RR	95%−CI	P−value	Tau2	Tau	I2
Omitting Adu−Afarwuah 2015	0.99	[0.92; 1.06]	0.73	0	0	0%
Omitting Ashorn 2010 complete FU	0.98	[0.91; 1.06]	0.61	0	0	0%
Omitting Ashorn 2010 simplified FU	0.98	[0.91; 1.06]	0.60	0	0	0%
Omitting Fawzi 2007	0.97	[0.90; 1.05]	0.44	0	0	0%
Omitting Moore 2009	0.99	[0.92; 1.06]	0.71	0	0	0%
Omitting Roberfroid 2008	0.98	[0.91; 1.06]	0.63	0	0	0%
Omitting Tofail 2008	0.98	[0.90; 1.07]	0.65	0	0	0%
Omitting West 2014	1.03	[0.91; 1.18]	0.63	0	0	0%
**Fixed Effect Model**	**0.98**	**[0.91; 1.06]**	**0.66**	**0**	**0**	**0%**

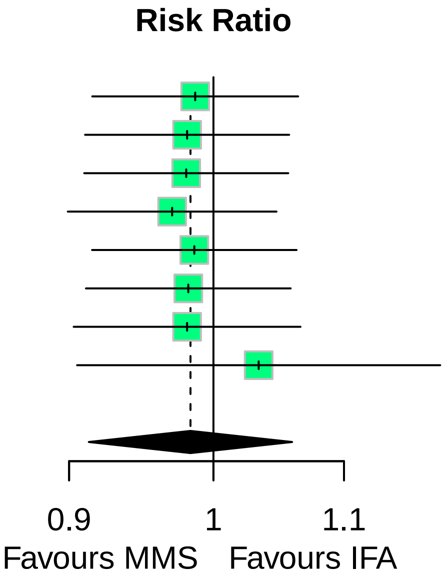

Leave−One−Out Sensitivity Analysis

Small HC at 6 Months, Random

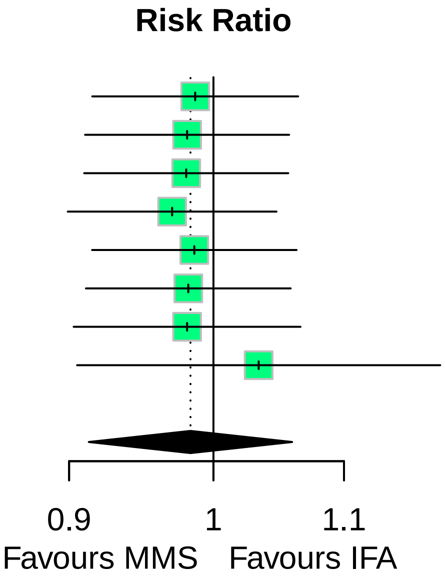

Study	RR	95%−CI	P−value	Tau2	Tau	I2
Omitting Adu−Afarwuah 2015	0.99	[0.92; 1.06]	0.73	0	0	0%
Omitting Ashorn 2010 complete FU	0.98	[0.91; 1.06]	0.61	0	0	0%
Omitting Ashorn 2010 simplified FU	0.98	[0.91; 1.06]	0.60	0	0	0%
Omitting Fawzi 2007	0.97	[0.90; 1.05]	0.44	0	0	0%
Omitting Moore 2009	0.99	[0.92; 1.06]	0.71	0	0	0%
Omitting Roberfroid 2008	0.98	[0.91; 1.06]	0.63	0	0	0%
Omitting Tofail 2008	0.98	[0.90; 1.07]	0.65	0	0	0%
Omitting West 2014	1.03	[0.91; 1.18]	0.63	0	0	0%
**Random effects model**	**0.98**	**[0.91; 1.06]**	**0.66**	**0**	**0**	**0%**

Leave−One−Out Sensitivity Analysis
Small HC at 12 Months, Fixed

Study	RR	95%−CI	P−value	Tau2	Tau	I2
Omitting Adu−Afarwuah 2015	0.97	[0.92; 1.02]	0.25	0	0	0%
Omitting Ashorn 2010 complete FU	0.96	[0.91; 1.01]	0.15	0	0	0%
Omitting Fawzi 2007	0.96	[0.91; 1.01]	0.15	0.0010	0.0310	11%
Omitting Roberfroid 2008	0.96	[0.91; 1.02]	0.20	0.0012	0.0347	14%
Omitting Tofail 2008	0.95	[0.89; 1.01]	0.08	0	0	0%
Omitting West 2014	1.01	[0.91; 1.12]	0.90	0	0	0%
**Fixed Effect Model**	**0.96**	**[0.91; 1.02]**	**0.18**	**0**	**0**	**0%**

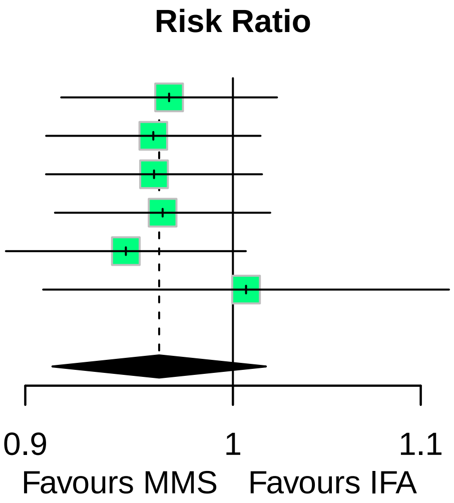

Leave−One−Out Sensitivity Analysis
Small HC at 12 Months, Random

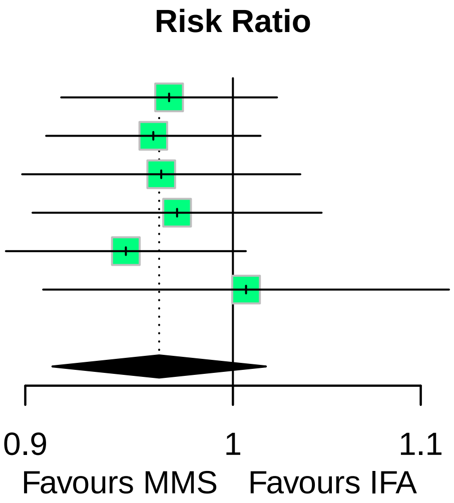

Study	RR	95%−CI	P−value	Tau2	Tau	I2
Omitting Adu−Afarwuah 2015	0.97	[0.92; 1.02]	0.25	0	0	0%
Omitting Ashorn 2010 complete FU	0.96	[0.91; 1.01]	0.15	0	0	0%
Omitting Fawzi 2007	0.96	[0.90; 1.03]	0.31	0.0010	0.0310	11%
Omitting Roberfroid 2008	0.97	[0.90; 1.05]	0.45	0.0012	0.0347	14%
Omitting Tofail 2008	0.95	[0.89; 1.01]	0.08	0	0	0%
Omitting West 2014	1.01	[0.91; 1.12]	0.90	0	0	0%
**Random effects model**	**0.96**	**[0.91; 1.02]**	**0.18**	**0**	**0**	**0%**

Leave−One−Out Sensitivity Analysis
Small HC at 18 Months, Fixed

Study	RR	95%−CI	P−value	Tau2	Tau	I2
Omitting Adu−Afarwuah 2015	1.05	[0.69; 1.62]	0.81	0	0	0%
Omitting Ashorn 2010 complete FU	0.80	[0.59; 1.09]	0.15	0	0	0%
Omitting Ashorn 2010 simplified FU	0.90	[0.67; 1.20]	0.48	0	0	0%
Omitting Fawzi 2007	0.88	[0.67; 1.15]	0.36	0	0	0%
**Fixed Effect Model**	**0.89**	**[0.68; 1.16]**	**0.38**	**0**	**0**	**0%**

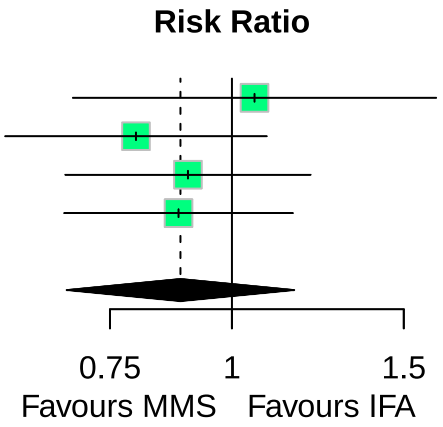

Leave−One−Out Sensitivity Analysis
Small HC at 18 Months, Random

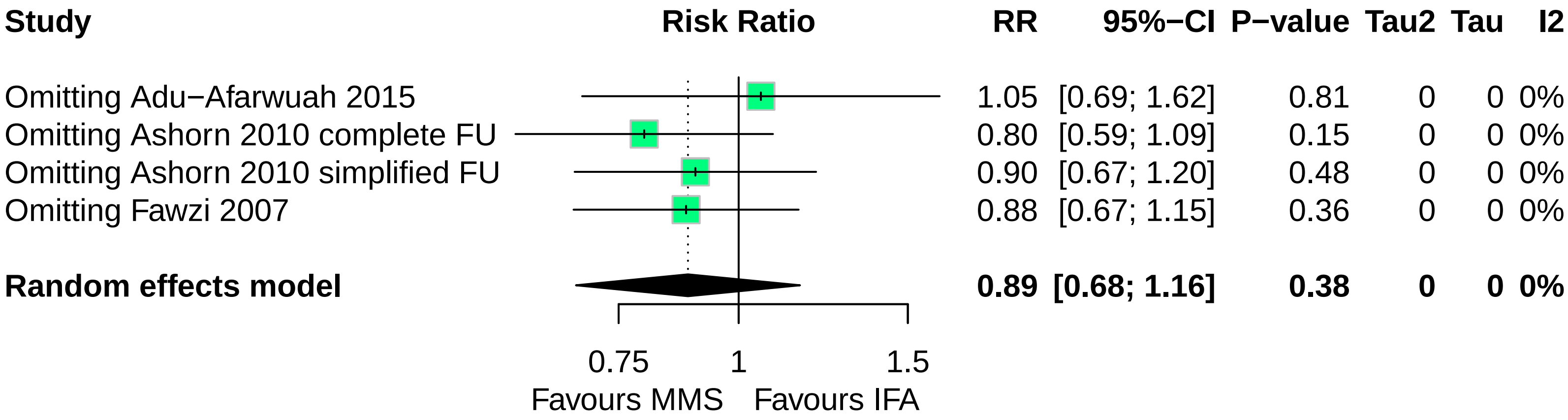

Leave−One−Out Sensitivity Analysis
Small HC at 24 Months, Fixed

Study	RR	95%−CI	P−value	Tau2	Tau	I2
Omitting Ashorn 2010 complete FU	0.99	[0.93; 1.06]	0.86	.	.	.
Omitting West 2014	1.19	[0.72; 1.94]	0.50	.	.	.
**Fixed Effect Model**	**1.00**	**[0.93; 1.06]**	**0.93**	**0**	**0**	**0%**

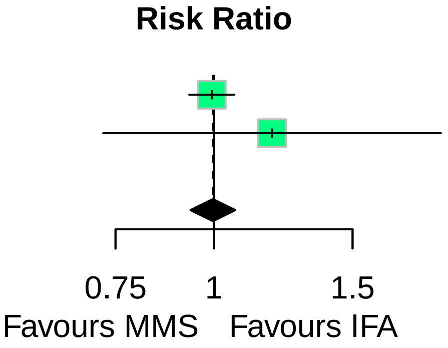

Leave−One−Out Sensitivity Analysis
Small HC at 24 Months, Random

Study	Risk Ratio	RR	95%−CI	P−value	Tau2	Tau	I2
Omitting Ashorn 2010 complete FU		0.99	[0.93; 1.06]	0.86	.	.	.
Omitting West 2014		1.19	[0.72; 1.94]	0.50	.	.	.
**Random effects model**		**1.00**	**[0.93; 1.06]**	**0.93**	**0**	**0**	**0%**

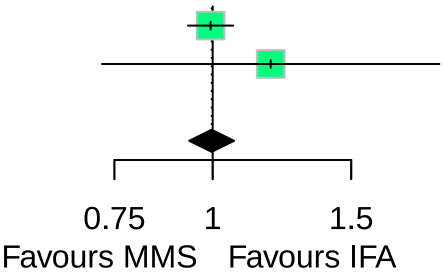

Leave−One−Out Sensitivity Analysis
Small MUAC at 3 Months, Fixed

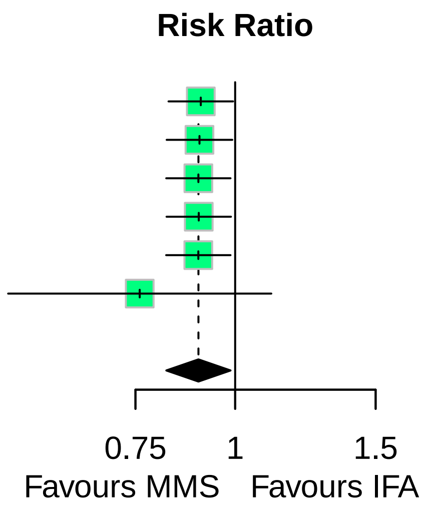

Study	RR	95%−CI	P−value	Tau2	Tau	I2
Omitting Adu−Afarwuah 2015	0.91	[0.83; 0.99]	0.04	0	0	0%
Omitting Fawzi 2007	0.90	[0.82; 0.99]	0.03	0	0	0%
Omitting Moore 2009	0.90	[0.82; 0.99]	0.03	0	0	0%
Omitting Roberfroid 2008	0.90	[0.82; 0.99]	0.03	0	0	0%
Omitting Sunawang 2009	0.90	[0.82; 0.99]	0.03	0	0	0%
Omitting West 2014	0.76	[0.52; 1.11]	0.16	0	0	0%
**Fixed Effect Model**	**0.90**	**[0.82; 0.99]**	**0.03**	**0**	**0**	**0%**

Leave−One−Out Sensitivity Analysis
Small MUAC at 3 Months, Random

Study	RR	95%−CI	P−value	Tau2	Tau	I2
Omitting Adu−Afarwuah 2015	0.91	[0.83; 0.99]	0.04	0	0	0%
Omitting Fawzi 2007	0.90	[0.82; 0.99]	0.03	0	0	0%
Omitting Moore 2009	0.90	[0.82; 0.99]	0.03	0	0	0%
Omitting Roberfroid 2008	0.90	[0.82; 0.99]	0.03	0	0	0%
Omitting Sunawang 2009	0.90	[0.82; 0.99]	0.03	0	0	0%
Omitting West 2014	0.76	[0.52; 1.11]	0.16	0	0	0%
**Random effects model**	**0.90**	**[0.82; 0.99]**	**0.03**	**0**	**0**	**0%**

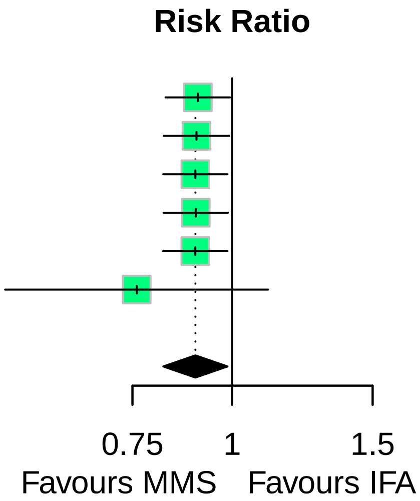

Leave−One−Out Sensitivity Analysis
Small MUAC at 6 Months, Fixed

Study	RR	95%−CI	P−value	Tau2	Tau	I2
Omitting Adu−Afarwuah 2015	0.99	[0.88; 1.12]	0.91	0	0	0%
Omitting Ashorn 2010 complete FU	0.98	[0.87; 1.11]	0.80	0	0	0%
Omitting Ashorn 2010 simplified FU	0.98	[0.87; 1.11]	0.80	0	0	0%
Omitting Fawzi 2007	0.97	[0.85; 1.10]	0.63	0	0	0%
Omitting Moore 2009	1.00	[0.88; 1.12]	0.94	0	0	0%
Omitting Roberfroid 2008	0.99	[0.87; 1.11]	0.83	0	0	0%
Omitting Sunawang 2009	0.99	[0.88; 1.12]	0.86	0	0	0%
Omitting West 2014	1.08	[0.83; 1.40]	0.56	0	0	0%
**Fixed Effect Model**	**0.99**	**[0.88; 1.11]**	**0.86**	**0**	**0**	**0%**

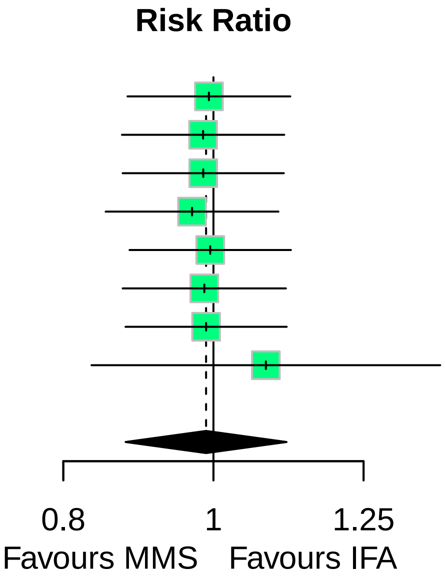

Leave−One−Out Sensitivity Analysis
Small MUAC at 6 Months, Random

Study	RR	95%−CI	P−value	Tau2	Tau	I2
Omitting Adu−Afarwuah 2015	0.99	[0.88; 1.12]	0.91	0	0	0%
Omitting Ashorn 2010 complete FU	0.98	[0.87; 1.11]	0.80	0	0	0%
Omitting Ashorn 2010 simplified FU	0.98	[0.87; 1.11]	0.80	0	0	0%
Omitting Fawzi 2007	0.97	[0.85; 1.10]	0.63	0	0	0%
Omitting Moore 2009	1.00	[0.88; 1.12]	0.94	0	0	0%
Omitting Roberfroid 2008	0.99	[0.87; 1.11]	0.83	0	0	0%
Omitting Sunawang 2009	0.99	[0.88; 1.12]	0.86	0	0	0%
Omitting West 2014	1.08	[0.83; 1.40]	0.56	0	0	0%
**Random effects model**	**0.99**	**[0.88; 1.11]**	**0.86**	**0**	**0**	**0%**

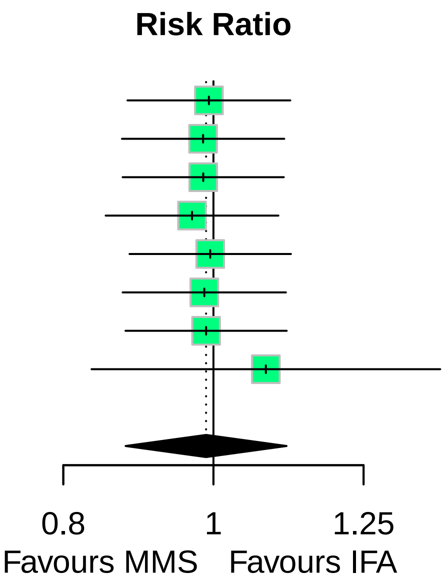

Leave−One−Out Sensitivity Analysis
Small MUAC at 12 Months, Fixed

Study	RR	95%−CI	P−value	Tau2	Tau	I2
Omitting Adu−Afarwuah 2015	1.00	[0.91; 1.10]	0.97	0.0135	0.1162	29%
Omitting Ashorn 2010 complete FU	0.99	[0.90; 1.08]	0.78	0.0194	0.1393	39%
Omitting Fawzi 2007	0.97	[0.89; 1.07]	0.60	0.0758	0.2753	55%
Omitting Roberfroid 2008	0.99	[0.90; 1.09]	0.85	0.0583	0.2415	60%
Omitting Sunawang 2009	1.00	[0.91; 1.09]	0.96	0.0447	0.2114	55%
Omitting West 2014	0.97	[0.77; 1.22]	0.80	0.1294	0.3597	60%
**Fixed Effect Model**	**0.99**	**[0.90; 1.09]**	**0.83**	**0.0387**	**0.1967**	**50%**

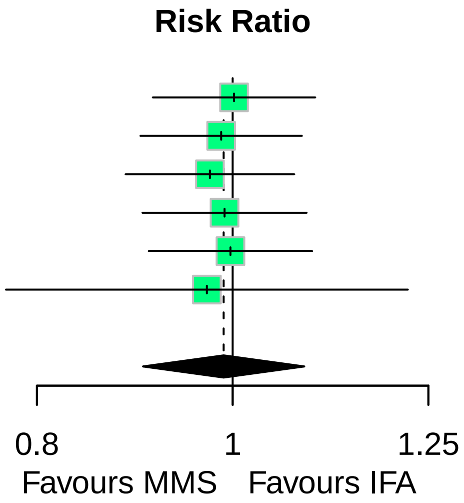

Leave−One−Out Sensitivity Analysis
Small MUAC at 12 Months, Random

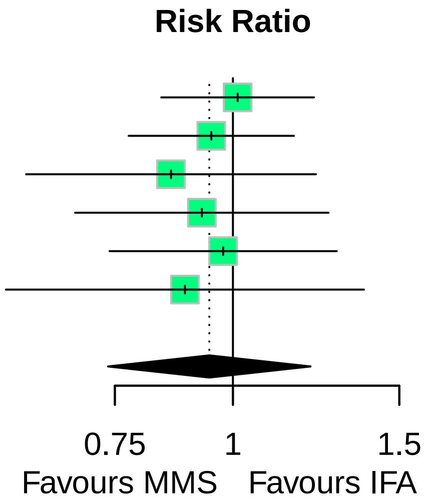

Study	RR	95%−CI	P−value	Tau2	Tau	I2
Omitting Adu−Afarwuah 2015	1.01	[0.84; 1.22]	0.90	0.0135	0.1162	29%
Omitting Ashorn 2010 complete FU	0.95	[0.78; 1.16]	0.61	0.0194	0.1393	39%
Omitting Fawzi 2007	0.86	[0.60; 1.22]	0.40	0.0758	0.2753	55%
Omitting Roberfroid 2008	0.93	[0.68; 1.26]	0.63	0.0583	0.2415	60%
Omitting Sunawang 2009	0.98	[0.74; 1.29]	0.87	0.0447	0.2114	55%
Omitting West 2014	0.89	[0.58; 1.38]	0.60	0.1294	0.3597	60%
**Random effects model**	**0.94**	**[0.74; 1.21]**	**0.65**	**0.0387**	**0.1967**	**50%**

Leave−One−Out Sensitivity Analysis
Small MUAC at 18 Months, Fixed

Study	Risk Ratio	RR	95%−CI	P−value	Tau2	Tau	I2
Omitting Adu−Afarwuah 2015		1.22	[0.55; 2.73]	0.62	0	0	0%
Omitting Ashorn 2010 complete FU		0.74	[0.38; 1.44]	0.38	0	0	0%
Omitting Ashorn 2010 simplified FU		0.90	[0.50; 1.63]	0.73	0	0	0%
Omitting Fawzi 2007		0.93	[0.53; 1.64]	0.80	0	0	0%
**Fixed Effect Model**		**0.91**	**[0.52; 1.59]**	**0.75**	**0**	**0**	**0%**

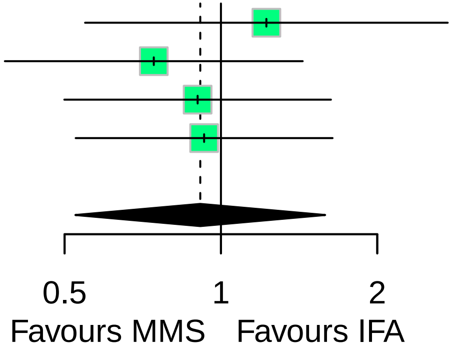

Leave−One−Out Sensitivity Analysis
Small MUAC at 18 Months, Random

Study	RR	95%−CI	P−value	Tau2	Tau	I2
Omitting Adu−Afarwuah 2015	1.22	[0.55; 2.73]	0.62	0	0	0%
Omitting Ashorn 2010 complete FU	0.74	[0.38; 1.44]	0.38	0	0	0%
Omitting Ashorn 2010 simplified FU	0.90	[0.50; 1.63]	0.73	0	0	0%
Omitting Fawzi 2007	0.93	[0.53; 1.64]	0.80	0	0	0%
**Random effects model**	**0.91**	**[0.52; 1.59]**	**0.75**	**0**	**0**	**0%**

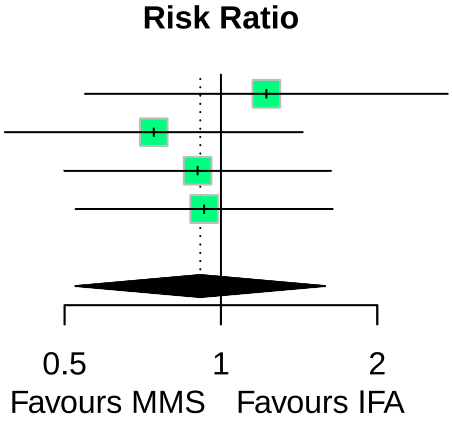

Leave−One−Out Sensitivity Analysis
Small MUAC at 24 Months, Fixed

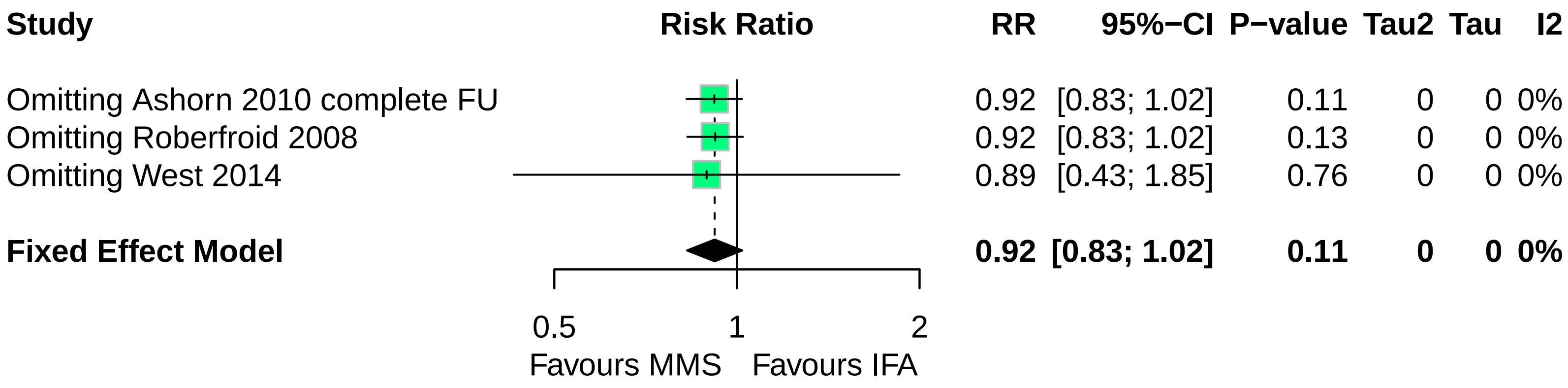

Leave−One−Out Sensitivity Analysis
Small MUAC at 24 Months, Random

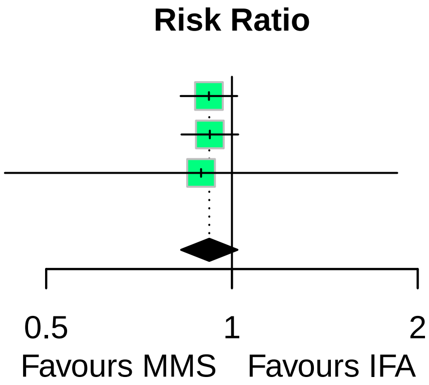

Study	RR	95%−CI	P−value	Tau2	Tau	I2
Omitting Ashorn 2010 complete FU	0.92	[0.83; 1.02]	0.11	0	0	0%
Omitting Roberfroid 2008	0.92	[0.83; 1.02]	0.13	0	0	0%
Omitting West 2014	0.89	[0.43; 1.85]	0.76	0	0	0%
**Random effects model**	**0.92**	**[0.83; 1.02]**	**0.11**	**0**	**0**	**0%**

Supplemental Figures 4 – Risk of Bias assessment

Supplemental Figure 4.1: Individually randomized controlled trials risk of bias for individual trials

Study	D1	D2	D3	D4	D5	Overall
Adu-Afarwuah, 2015	+	+	+	+	+	+
Ashorn, 2015	+	+	+	+	+	+
Fawzi, 2007	+	+	+	+	+	+
Friis, 2007	+	+	+	+	+	+
Kaestel, 2005	+	+	+	+	+	+
Liu, 2013	+	+	+	+	+	+
Moore, 2009	+	+	+	+	+	+
Osrin, 2005	+	+	+	+	+	+
Tofail, 2008	+	+	+	+	+	+
Ramakrishnan, 2003	+	+	+	+	+	+
Roberfoid, 2008	+	+	+	+	+	+

\+ Low risk
! Some concerns
\- High risk

D1 Randomisation process
D2 Deviations from the intended interventions
D3 Missing outcome data
D4 Measurement of the outcome
D5 Selection of the reported result

Supplemental Figure 4.2: Cluster randomized controlled trials risk of bias for individual trials

Study	D1a	D1b	D2	D3	D4	D5	Overall
Bhutta, 2009	+	+	+	+	+	+	+
Bliznashka, 2021	+	!	!	!	+	+	!
Christian, 2003	+	+	+	!	+	+	!
Shankar, 2008	+	+	+	+	+	+	+
Sunawang, 2009	+	+	+	+	+	+	+
West, 2014	+	+	+	+	+	+	+
Zagre, 2007	+	+	+	!	+	+	!
Zeng, 2008	+	+	+	+	+	+	+

\+ Low risk
! Some concerns
\- High risk

D1a Randomisation process
D1b Timing of identification or recruitment of participants
D2 Deviations from the intended interventions
D3 Missing outcome data
D4 Measurement of the outcome
D5 Selection of the reported result

Supplemental Figure 4.3: Individually randomized controlled trials risk of bias summary plot

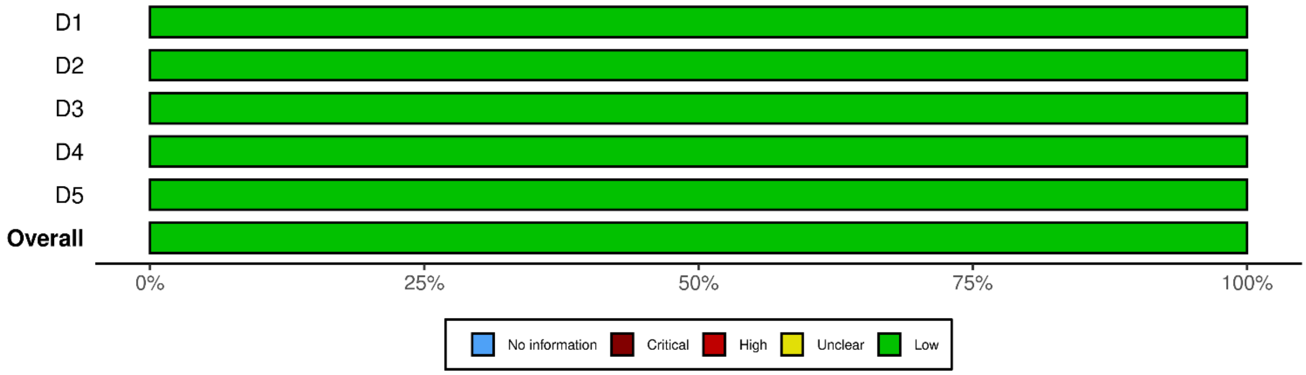

D1a=randomization process; D2=deviations from the intended interventions; D3=missing outcome data; D4=measurement of the outcome; D5=selection of the reported result

Supplemental Figure 4.4: Cluster randomized controlled trials risk of bias summary plot

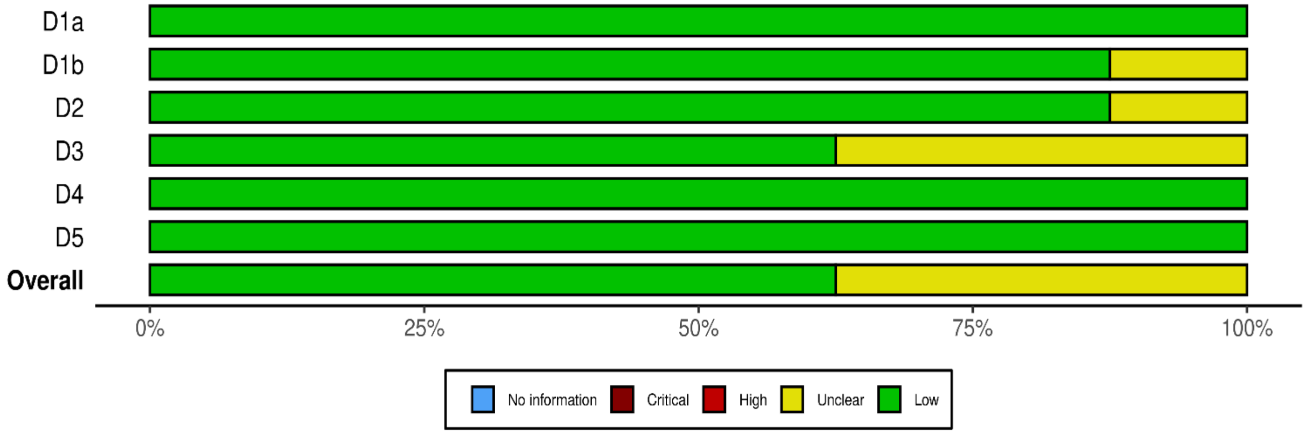

D1a=randomization process; D1b=timing and identification or recruitment of participants; D2=deviations from the intended interventions; D3=missing outcome data; D4=measurement of the outcome; D5=selection of the reported result

Supplemental Figures 5 – Publication bias assessment

Supplemental Figure 5.1: Contour-enhanced funnel plot for length at birth

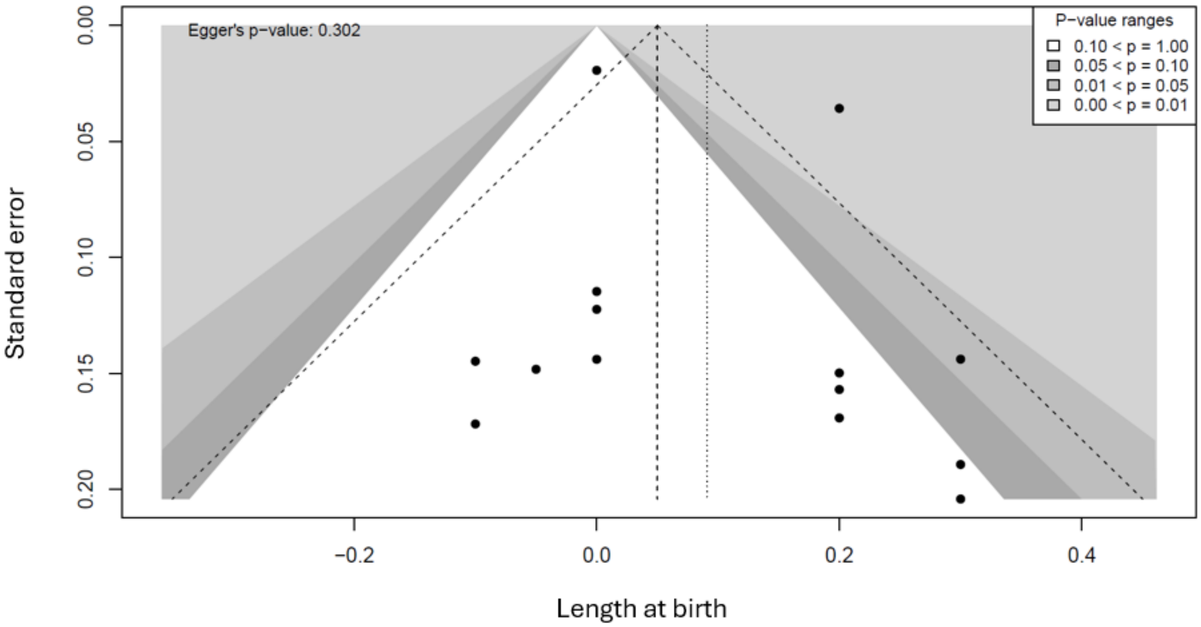

Supplemental Figure 5.2: Contour-enhanced funnel plot for head circumference (HC) at birth

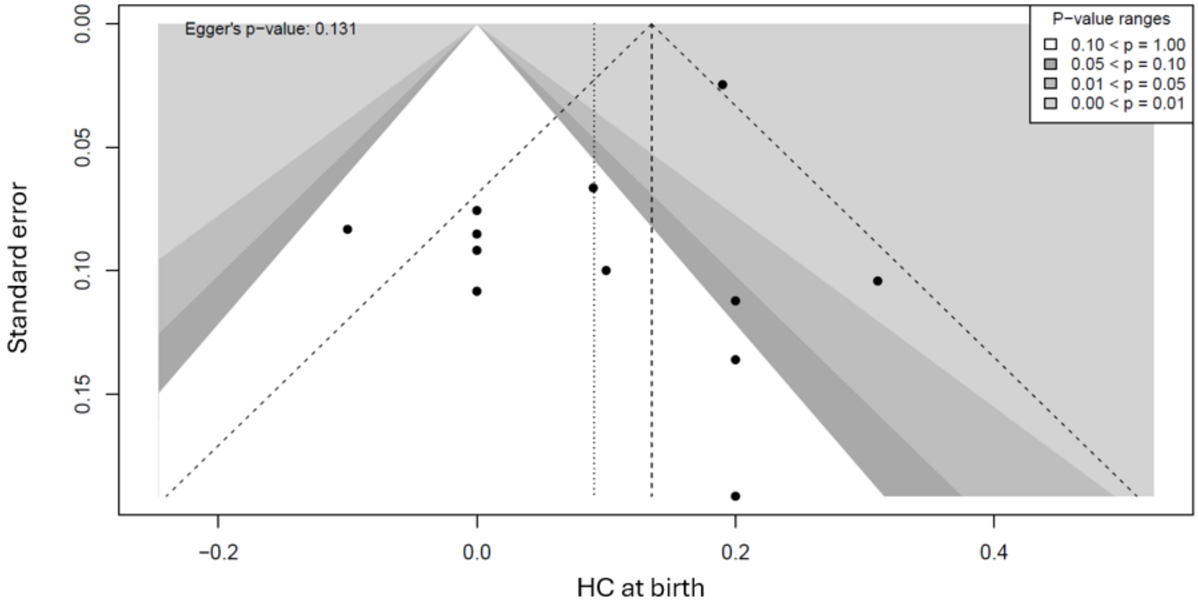

Supplemental Figure 5.3: Contour-enhanced funnel plot for weight at birth

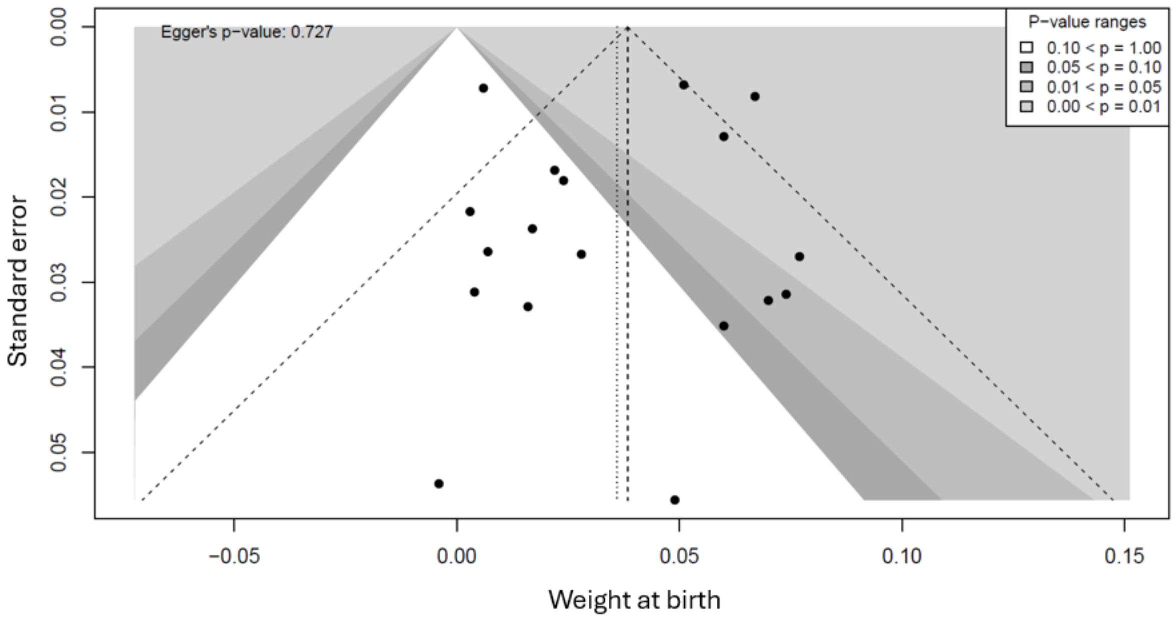

Supplement: Multimedia component 1 [file mmc1.pdf]
